# Exploring latitudinal gradients and environmental drivers of amphipod biodiversity patterns regarding depth and habitat variations

**DOI:** 10.1038/s41598-024-83314-6

**Published:** 2024-12-18

**Authors:** Farzaneh Momtazi, Hanieh Saeedi

**Affiliations:** 1 Department of Marine Zoology, Biodiversity Information Section, Senckenberg Research Institute and Natural History Museum, Senckenberganlage 25, 60325 Frankfurt am Main, Germany; 2 Marine Bioscience Department, Iranian National Institute for Oceanography and Atmospheric Science (INIOAS), Tehran, Iran; 3 Department 15 - Life Sciences, Institute for Ecology, Evolution and Diversity, Goethe University Frankfurt, Max-von-Laue-Straße 13, 60438 Frankfurt am Main, Germany

**Keywords:** Amphipoda, biodiversity hotspots, environmental variables, temperature, bimodality, Biodiversity, Biogeography, Marine biology

## Abstract

Amphipods are known as umbrella species in conservation biology that their protection indirectly protects other species. Recent hypotheses suggest a bimodal latitudinal global species richness pattern for amphipods, irrespective of species’ depth or habitat type. Phylogeographic hypotheses suggested two distinct procedures for amphipod diversification: ecological radiation and Pangea fragmentation. This study aimed to revisit the bimodal latitudinal pattern of species richness for amphipods, investigate similarities in species composition and main environmental amphipod distribution drivers regarding their depth and habitat variations. The comprehensive database was collected from open-access data and a personal sampling database from the Persian Gulf and the Gulf of Oman. After rigorous data quality controls, the final dataset comprised 1,142,416 distribution records of 6,424 accepted marine amphipod species distributed from 0 to 10,900 m depth. All the species were grouped into pelagic and benthic, shallow-water (> 200 m), and deep-sea (< 200 m). Alpha and gamma species richness and the expected number of species per 50 random samples (ES50) were calculated. Our findings showed that species richness patterns in amphipods are shaped not only by habitat types but also by depth, and they are not significantly bimodal in all groups. Also, the Beta diversity of benthic species revealed distinct diversification patterns between benthic and pelagic species. The species similarity clusters revealed a fragmentation between Gondwana and Laurasia for shallow benthic species. However, the similarity between pelagic amphipod communities is more compatible with ecological parameters. Generalized Additive Models (GAMs) highlighted that environmental variables play a significant role in species distributions, for example, temperature and chlorophyll levels were the main predictors of species richness in shallow waters. However, a complete effect of multiple environmental variables is responsible for deep-sea species gradients. These findings are crucial information to be considered when managing the species richness and establishing conservation priorities for their habitats.

**Supplementary Information:**

The online version contains supplementary material available at 10.1038/s41598-024-83314-6.

## Introduction

A recent report showed that 60% of marine biodiversity is threatened, and over 40% of the world’s oceans need protection to preserve their biodiversity^1^. To determine the position of areas that need a higher level of protection, we need to map the global distribution of marine organisms to discover hot and low spots of species richness^2^. Amphipods are a dominant group in marine ecosystems with a key role in energy flux, food webs, and detecting marine pollution^3–7^. Amphipods live in both pelagic and benthic habitats. The suborder Hyperiidea, a main group of pelagic amphipods, is found from the surface to the abyssal depths, and even in the hadal zone up to 6,000 m^8,9^. Similarly, benthic amphipods are reported from a wide range of bathymetric distributions (0–10,000 m)^10^. Understanding amphipods’ distribution patterns helps protect the health and resilience of marine ecosystems^11^.

Three main hypotheses explain the distribution patterns of marine organisms, focusing on dispersal, vicariance (where distribution patterns are shaped by historical events), and ecological models that emphasize present-day environmental factors^12^. The historical global distribution of amphipods has been studied using the cladistic morphological relationship and molecular phylogenetic analysis (Ref^13,14^). Those studies proposed two geological times of diversifications for amphipods. The cladistic analysis based on morphologic data^13^ and some molecular analysis^15^ suggested that the current diversity of amphipods was created by the end of the Palaeozoic or Early Mesozoic^16^ due to the fragmentation of Pangea. In contrast, some molecular analyses promoted the diversification time to the post-Jurassic period due to global changes and the existence of suitable ecological conditions for amphipods^14,17,18^. Despite numerous historical studies on amphipod diversity, surviving their present global distribution patterns have been largely overlooked.

Only, Arfianti & Costello^19^ studied present amphipod occurrence data (approximately 400,000 records) and proposed a globally bimodal pattern for amphipod species richness distribution; they also supported the paleozoic time for diversification amphipods. The existence of a bimodal pattern for global species richness distribution with latitude in correlation with water temperature was accepted in several studies for different marine organisms^20–22^. However, Arfianti & Costello have not considered the depth and ecological habitats (e.g., benthic or pelagic) in amphipods that affect the distribution patterns in marine environments^23–25^. This classification of the species into benthic and shallow is crucial as, for example, the pelagic species richness is correlated with water characteristics such as sea surface temperature, tide height, and mixed layer depth (the depth at which the surface temperature cools by 0.2 °C)^26–29^, but not the benthic species. Additional factors such as sedimentology, wave dynamics, and tidal dynamics^30,31^ were considered in benthic species distribution. Key questions persist: Does the proposed bimodal pattern and the geological timing of amphipod diversification hold true when considering depth and ecological habitats? Furthermore, which environmental variables significantly shape global amphipod species richness, particularly when comparing shallow-water and deep-sea communities?

We thus designated extensive analysis to investigate the global latitudinal and bathymetrical diversity gradients of amphipod species. To do this, we compiled a comprehensive database containing 1,740,216 occurrence records from different sources, including the author’s personal databases from the Persian Gulf and the Gulf of Oman, literature reviews, and museum collection databases, in addition to open access databases such as OBIS and GBIF. The regional personal dataset was composed of 1,509 records belonging to 117 species which 52 species were endemic to the Persian Gulf and the Gulf of Oman. This personal dataset significantly strengthened the final dataset in tropical and subtropical regions where data were deficient. We hypothesized that there are different species composition and distribution patterns for habitat types and depth groups (shallow vs. deep and benthic vs. pelagic) and distinct environmental predictors explain the species richness patterns in shallow and deep-sea as well as benthic versus pelagic fauna.

## Materials and methods

### Data extraction and cleaning

**Fig. 1:**
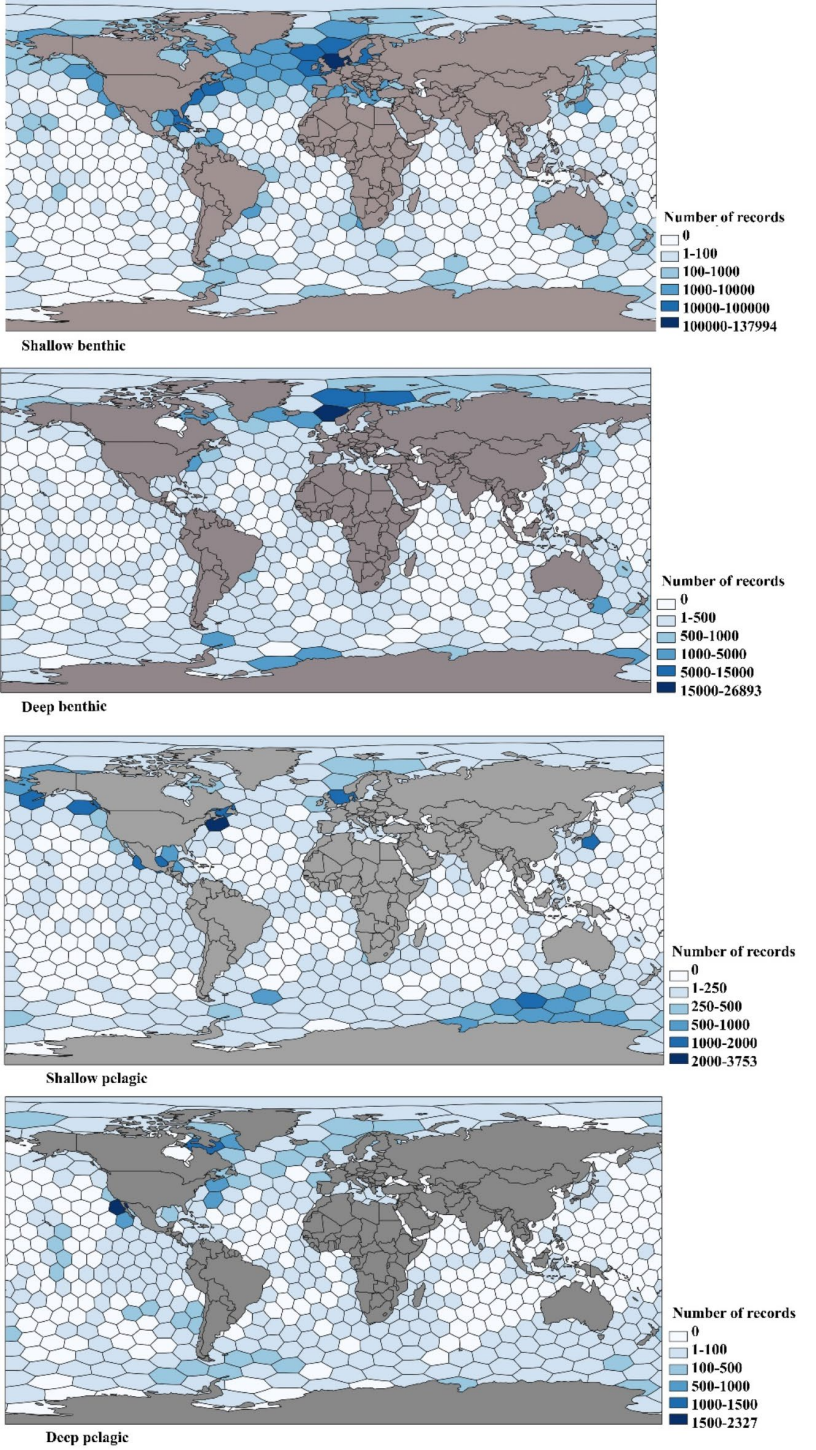
The number of occurrence records per c. 50,000 km^2^ hexagonal cells for benthic and pelagic groups in shallow waters and the deep sea. QGIS 3.28.10 was used to create the figure.

The final dataset contained all distribution data available in OBIS and GBIF, along with 1,509 records of 117 species from depths 0 to 1055 m obtained from literature, museum data, and personal data of sampling during 10 years in the Persian Gulf and the Gulf of Oman (https://ipt.iobis.org/obis-deepsea/resource?r=amphipod_persiangulf). The personal dataset contained 51 endemic species from unsampled and unprotected areas in the subtropical region (Fig. 1 S2). The citations of the used datasets can be found in SI (Tables 1 and 2 S1). The dataset, which includes 1,740,216 records, underwent quality control following the methodology outlined in Saeedi et al.^32^. All species names were matched against the World Register of Marine Species and synonyms were reconciled by “obistools”^33^. Invalid records such as those without geographic coordinates, duplicated entries, fossil records, coordinate uncertainties exceeding 100 km, or data located on land or without depth were removed using R 4.1.1^34^, and the packages “tidyverse ”^35^ and “scrubr”^36^. The dataset was then divided into pelagic and benthic groups and shallow-water (> 200 m) and deep-sea (< 200 m).

Environmental benthic variables including average bottom temperature (°C), dissolved oxygen (mol.m − 3), primary productivity (g.m − 3.d − 1), chlorophyll (mg.m − 3), current velocity (m − 1), phosphate (PO4) (mol.m − 3), silicate (SiO4) (mol.m − 3), depth (m) salinity (PSS), and nitrate (NO3) (mol.m − 3) were extracted from Bio-ORACLE^37,38^ with 5 arcmins (c. 9.2 km) spatial resolution using R packages of “sdmpredictors” and “leaflet”. Calculating the mean of each variable per hexagon was carried out by QGIS^39^.

### Data analysis

The R packages “tidyverse”, “openxlsx”^40^, “MASS”^41^, “ggplot2”^42^, and “sf”^43^ were used for importing, manipulating, and plotting refined data. The distribution pattern of amphipods was calculated using alpha, beta, and gamma diversity. The global distribution patterns of the total number of occurrence records (frequency), number of species (alpha diversity), and the estimated species for 50 records using rarefaction (ES50) per hexagon (as shown in Fig. 1) were calculated. The results were then plotted using QGIS 3.16 software^39^. Gamma diversity of amphipods (number of species per 5° latitudinal band) and ES50 per 5° latitudinal band were measured using the R package “vegan”^44^. Kernel curves, Anderson-Darling normality test^45^, and Hartigans’ dip test^46^ were used to investigate significant bimodality patterns in species distribution graphs (Table 8 S2 and Fig. 7 S2). To define the biogeographic pattern of amphipod communities, the beta diversity was analyzed using cluster analysis within realms defined by Spalding et al.^47^, for benthic and pelagic species groups with less than 200 m depth. The used marine realms were taken from the Marine Ecoregions of the World (MEOW) polygon dataset^47^. To perform the cluster analysis, the “vegan” package in R^44^ was used, with Euclidean distance and group-average linkage, which is recommended for analyzing species composition. To obtain the Bootstrap support, ordinary bootstrap resampling and multiscale bootstrap resampling (AU)^48^ were used, following the R package Pvclust^49^.

It was not feasible to extract environmental factors for pelagic species due to their widespread distribution and the lack of a specific depth. Therefore, we only analyzed benthic species to establish a connection between environmental factors and their distribution. We have applied principal component analysis (PCA), Generalized additive models (GAMs), and General Linear models (GLM) to map the correlation between the species richness and environmental variables. The R packages “mgcv”, “ggplot2” and “ggcorrplot” were utilized for this purpose. The PCA analysis was employed to understand the variation in shallow-water and deep-sea benthic groups, as well as the variation of variables within each group. GAMs analysis was used to identify the main variables that drive distribution patterns in hexagons. General Linear models (GLM) were then employed to identify these variables at 5° latitudinal bands, following the methodology established by Saeedi et al.^32^.

The study aimed to identify the most appropriate model for analyzing the distribution of species numbers and ES50 in both deep and shallow waters. Four types of Generalized Additive Models (GAMs) were employed in the analysis: an intercept-only model, individual models for each environmental predictor, a model that considered the combined effects of all environmental predictors, and a spatial autocorrelation model. The intercept model served as the null hypothesis, indicating that environmental factors or spatial autocorrelation did not influence response variables. The AIC analysis was used to compare models for goodness of fit while penalizing for overparameterization in benthic amphipod assemblage and estimate the relative support for each model when compared with others.

## Results

### Biodiversity analysis

The final dataset comprised a total of 1,142,416 distribution records belonging to 6,424 marine amphipod species distributed from 0 to 10,900 m depth. Among the genera, *Liljeborgia* Spence Bate, 1862^50^ had the broadest distribution range in all 5° latitudinal bands, except at latitude 0° and 80°-90°. *Corophium volutator*^51^ was the most frequent benthic species with 26,701 occurrence records. Amphipods’ shallow benthic occurrence records were more numerous than for other groups, with a maximum of 137,994 records per 50,000 km^2^ hexagon cell (Fig. 1).

**Fig. 2:**
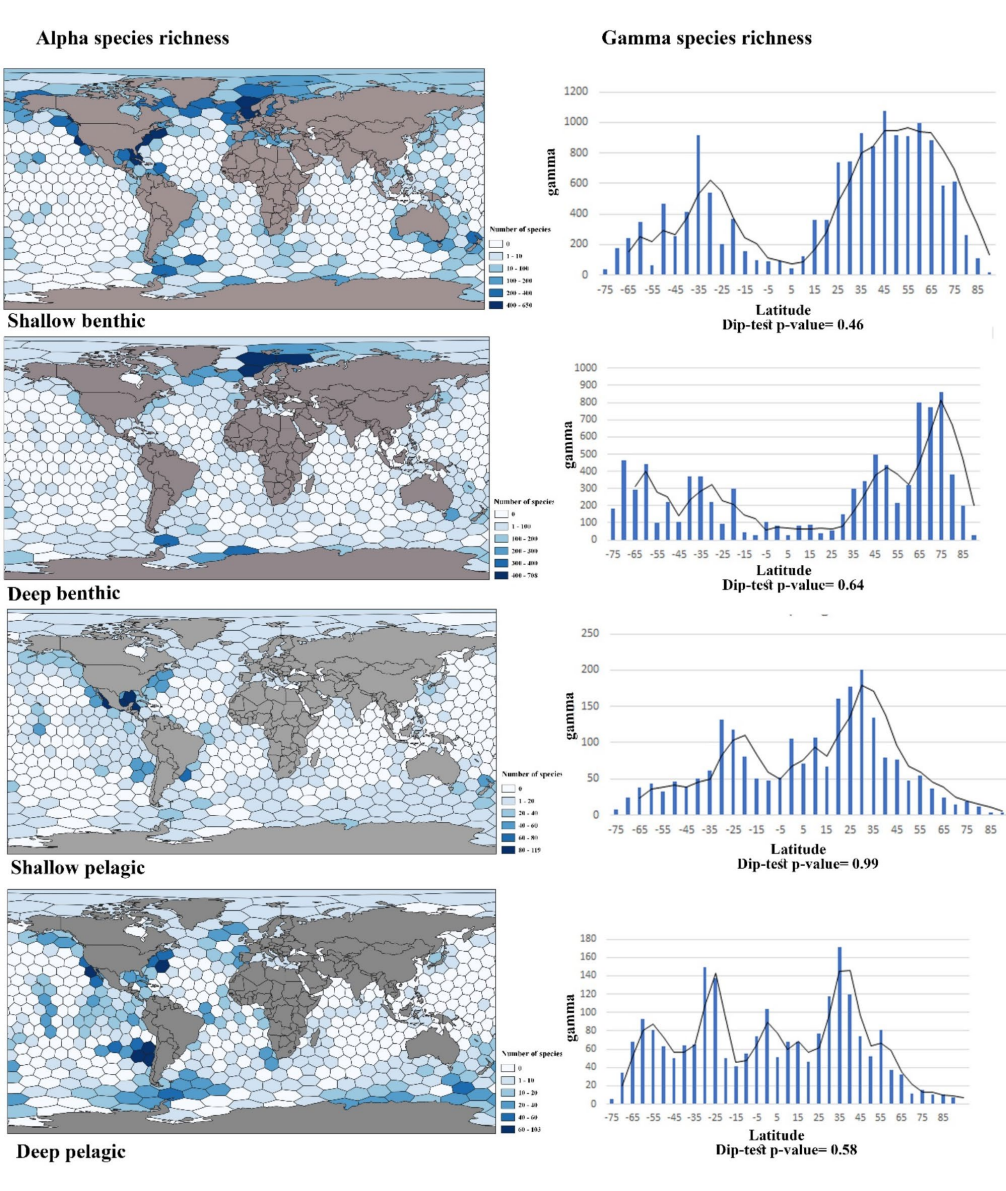
The left plots show the number of species per c. 50,000 km^2^ hexagonal cells (alpha diversity). The right graphs show the gamma species richness (number of species per 5° latitudinal bands) of benthic and pelagic groups in the shallow and deep-sea over a 5° latitude band. Dip-test p-value higher than 0.05 rejects the null hypothesis. The figure was created using QGIS 3.28.10.

**Fig. 3:**
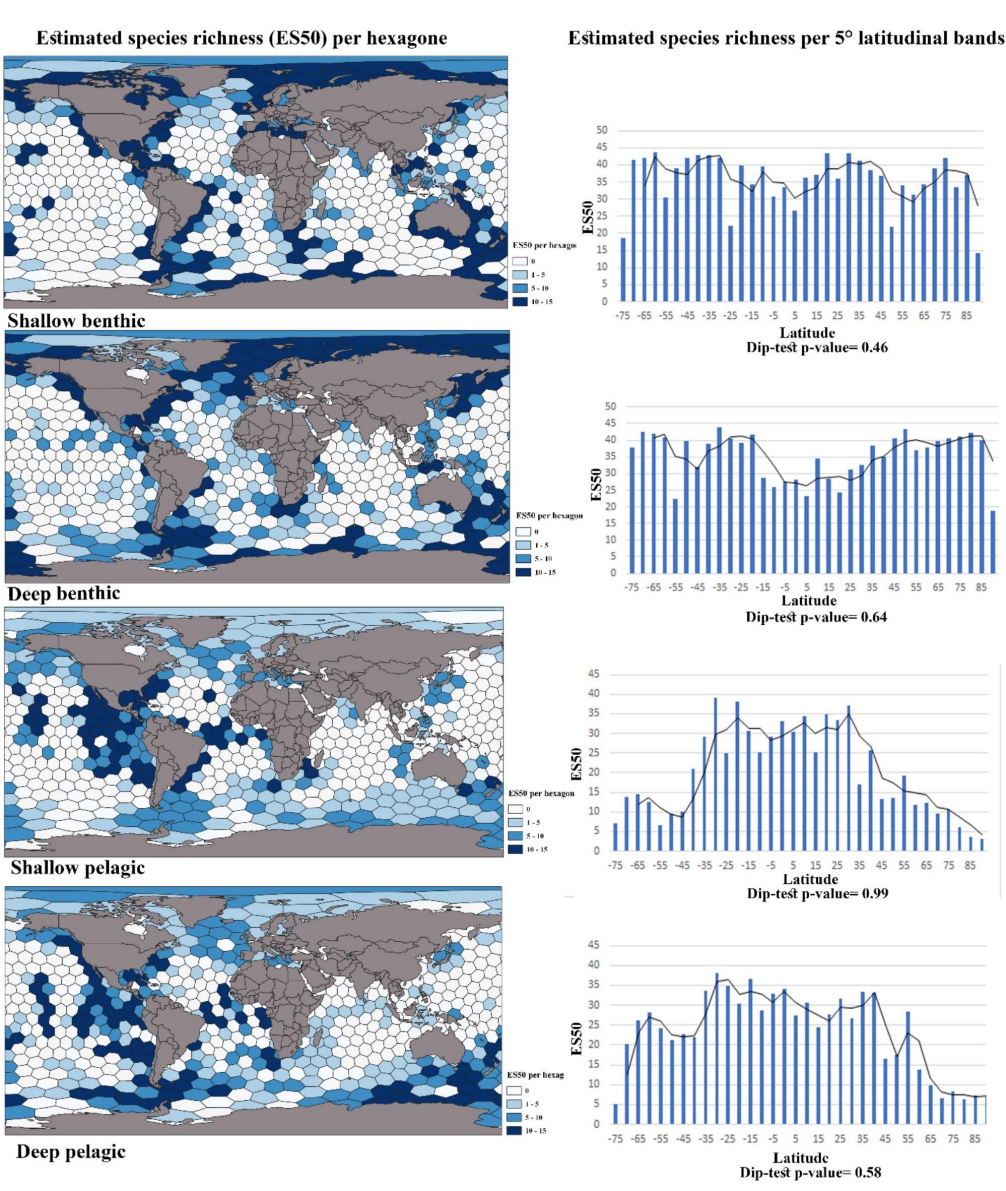
The right column shows the global pattern of ES50 (estimated species for 50 records) calculated per c. 50,000 km^2^ hexagonal cells and the left column shows ES50 per 5° latitude band, for benthic and pelagic groups in shallow and deep-sea. Dip-test p-value bigger than 0.05 rejects the null hypothesis.QGIS 3.28.10 was used to create the figure.

**Fig. 4:**
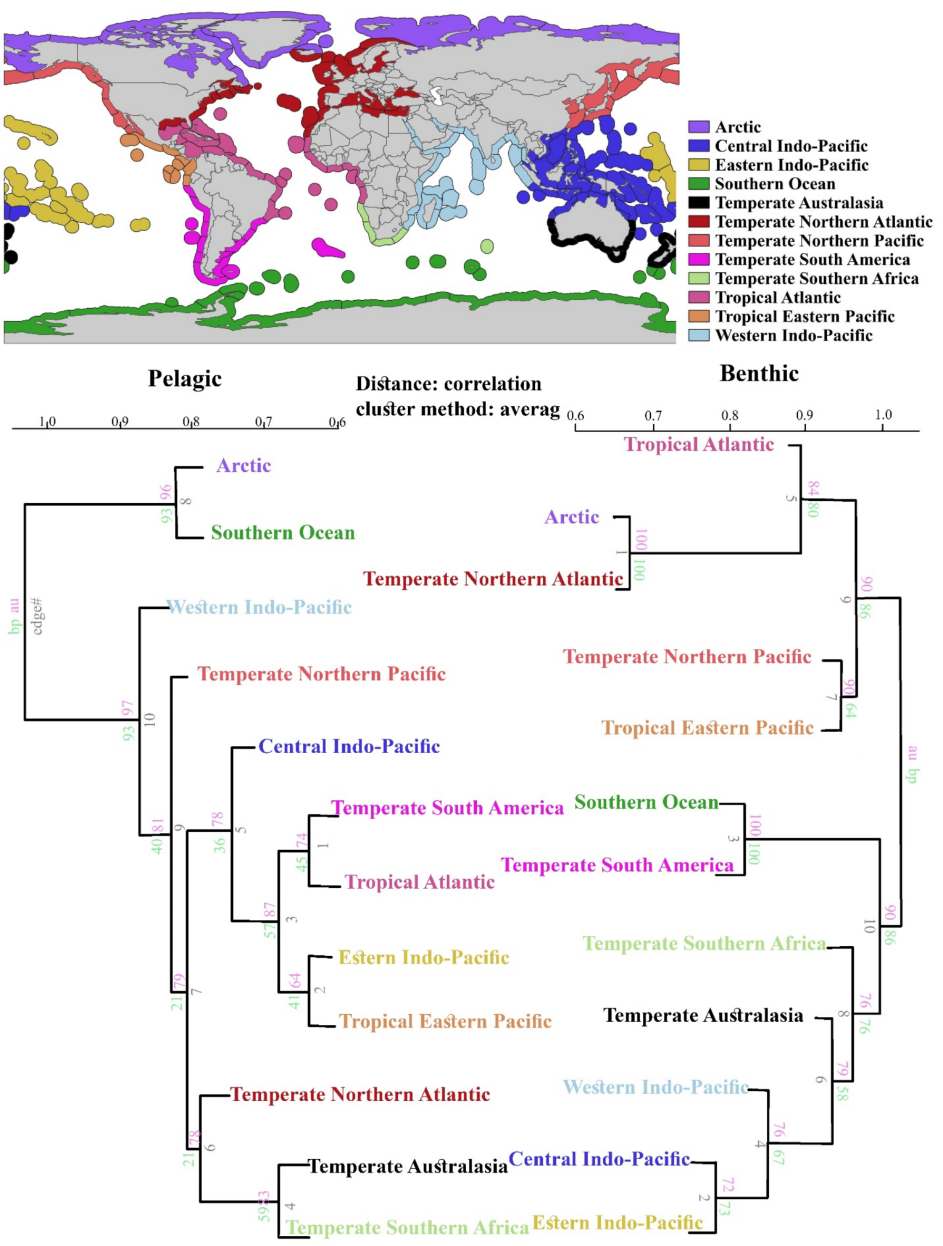
The cluster dendrogram plot represents the beta diversity (amphipod species richness (presence/absence) between different marine realms). The polygon shapefile was extracted from the Marine Ecoregions of the World (MEOW)^47^. The species are divided into benthic (on the right) and pelagic species (on the left). The numbers above each edge in the clusters represent the probability of nodes below that edge occurring as a cluster in resampled trees. The significant level comparisons were done using ordinary bootstrap resampling (BP, shown in green) and multiscale bootstrap resampling (AU, shown in red). The black numbers represent the realm cell IDs.

**Fig. 5:**
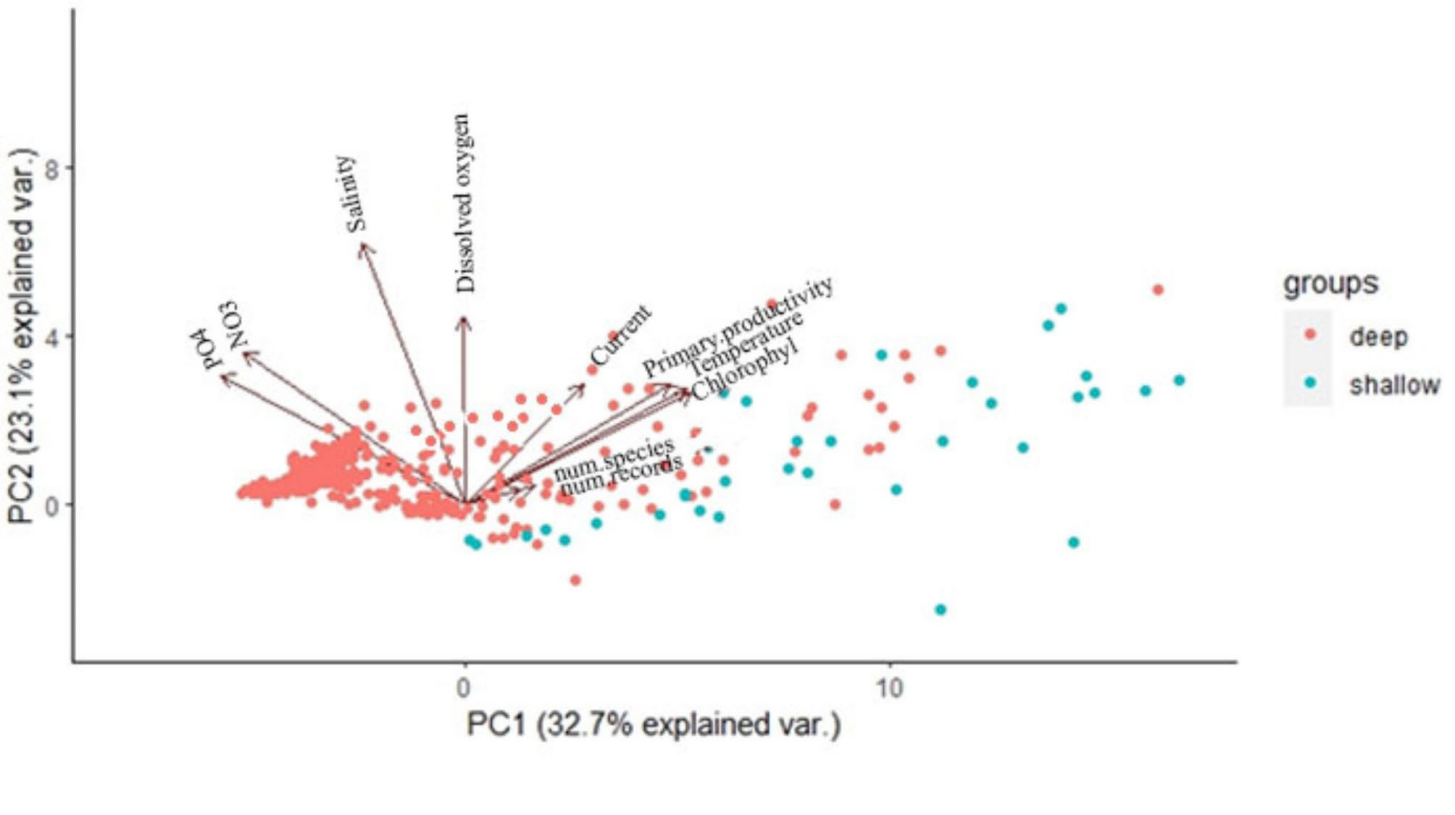
The PCA analysis of the average environmental factors within each hexagon, relative to the number of benthic amphipod species and their records. The hexagons were classified as deep (with over 200 m depth) and shallow (with less than 200 m depth).

To explore the global species richness patterns of amphipods, the number of records and number of species were calculated in each hexagone. All the distribution points of the amphipods are shown in Fig. 2 S2 and extracted maps for the pelagic and benthic groups without regarding depth were embedded in Figs. 3–6 S2. Pelagic species were predominantly found in the Caribbean Sea and Temperate northern Atlantic realm regions. However, they were less distributed in the western, eastern, and central Indo-Pacific realms. The highest number of shallow pelagic species was recorded in Nova Scotia with 3,753 occurrence records per 50,000 km^2^ hexagon cell. In contrast, the deep pelagic taxa were recorded mostly from the Gulf of California. Shallow benthic fauna was mostly recorded from the northern temperate Atlantic; whereas, deep benthic fauna were more abundant in the Arctic realm and the Norwegian Sea.

This study found differences in species richness patterns between benthic and pelagic species in shallow-water and deep-sea. The shallow waters in the Norwegian Sea, North Sea, coastal lines of the USA in the Atlantic, and Pacific Oceans were hotspots for benthic amphipod species richness; however, the deep benthic species hotspot was only limited to the Norwegian Sea and Barents Sea. The tropical Atlantic, especially the Gulf of Mexico, was recorded as a shallow pelagic hotspot of amphipod species richness, while the highest number of deep pelagic species were reported from the temperate Pacific South America and the northern temperate Atlantic and Pacific realms. The number of species per hexagon was higher in benthic fauna compared to the pelagic ones, and the highest number of species per hexagon was recorded for deep benthic species (708 species) (see Fig. 2).

The global gamma diversity pattern of amphipods was calculated by measuring the total number of species per 5° latitudinal bands. ES50 was calculated based on the number of expected species per 5° latitudinal bands. The results showed that gamma diversity was affected by depth and habitat types. The kernel curve and normality test (Fig. 7 S2) showed that the distribution of all groups was not either normal (Anderson-Darling normality test) nor significantly different from an unimodal pattern (Hartigans’ dip test) (Fig. 2, and 3), the significant bimodal pattern was not confirmed for them. The null hypothesis in the dip-test shows an unimodal distribution pattern. The p-value higher than 0.05 accepts the null hypothesis. Latitudinal species richness pattern in benthic species recorded below 200 m depth showed the highest frequencies in latitudes − 55°, -10° in the southern hemisphere, and latitudes 5° and 55° in the northern hemisphere. Shallow pelagic species had the highest gamma diversity in subtropical regions as well as temperate regions. The gamma diversity of deep pelagic species had the same pattern as the shallow water, with an additional peak in the subarctic region (-60°) (see Figs. 2 and 6 S2).

The number of expected species per hexagon, also known as rarefied ES50, was also different between pelagic and benthic species in shallow-water and deep-sea (Fig. 3). The hotspots of ES50 species richness in both shallow and deep benthic species differed in the Mediterranean Sea and Japan Sea, where shallow benthic species were more prevalent. The highest number of ES50 in pelagic species was found in the tropical Atlantic, tropical eastern Pacific, and temperate southwest Atlantic realms (Fig. 3).

The cluster analysis of shallow amphipod species revealed distinct clusters for benthic and pelagic groups. The benthic group was divided into two main branches (Fig. 4, right cluster), with the first branch consisting of realms from Gondwana lands, including the Indian Ocean, Australasia, South and temperate South America, and Africa; while the second branch included Laurasia realms of tropical eastern Pacific, temperate northern Pacific, Arctic, tropical and temperate North Atlantic. The Arctic and the temperate North Atlantic Ocean had the highest degree of similarity in benthic amphipod assemblages. In addition, a branch of different realms of the Indian Ocean was identical and the high similarity between the Southern Ocean and the Temperate Southern Atlantic was obtained. In the pelagic group, the polar region, including the Arctic and Southern Ocean realms, formed a distinct group from other realms. The Western Indo-Pacific was separated from the remaining realms, and it was then possible to identify the realms belonging to temperate and tropical regions (Fig. 4, left cluster). The temperate Australasia, temperate southern Africa, and temperate northern Atlantic made a group as well as a group of tropical Atlantic, temperate southern America, eastern Indo-Pacific, and tropical eastern Pacific.

### Environmental variables analysis

The Pearson correlation and PCA analyses were used to show the relation between environmental factors and the number of benthic species and records. Due to the correlation between depth and other environmental variables and to avoid its additional effect on depth groups, it was removed from the variable set in the PCA analysis. The PCA results showed that the first PCA component (32.7% of variance) partially separated deep-sea and shallow benthic amphipod assemblages (Fig. 5, Table 1 S2). Temperature, chlorophyll, and primary productivity variations had the main positive effect on the first component, and the amount of nitrate and phosphate were the variables with the highest negative impact on it. The shallow hexagons were characterized by positive values and deep hexagons were mostly identified by negative values on the first component. Therefore, shallow hexagons showed stronger correlations with temperature and chlorophyll levels. However, amphipod communities in the deep sea encountered a broader range of environments, leading to their distribution along the first component including from lower to higher degrees of variation.

The Pearson coefficient analysis supported the PCA results as key environmental factors shaping amphipod biodiversity (Fig. 7 S2). In shallow assemblages, phosphate (PO4) and depth exhibited a negative correlation with the number of species records and richness, while temperature and chlorophyll showed a positive correlation. Notably, in the deep-sea, only dissolved oxygen exhibited a positive correlation with species richness and the number of records but depth and PO4 had a negative correlation with biodiversity metrics.

GAM analyses were performed to find which variables could predict the distribution of species richness and ES50 of amphipods for benthic communities in each hexagon and GLM analysis for each 5° latitudinal band. Due to the significant correlation observed between PO4, SiO4, and NO3 (Fig. 7 S2), as well as the correlation between primary productivity and chlorophyll, only PO4 and chlorophyll were selected from these sets for the GLM and GAM analyses. Other factors used in GLM and GAM were dissolved oxygen, salinity, current, temperature, depth, and pH. The GAMs models indicated that the most important environmental variables for species richness and ES50 patterns in shallow waters were chlorophyll (deltaAIC = 0.00 and 67.9% of the variation) and temperature (deltaAIC = 0.00 and 32.8% of the variation), respectively (Table 2 S2). Following temperature and chlorophyll, the model including all environmental variables with delta AIC = 0.07 and 67.9% deviance explained was the best-fitted model for species richness. The dissolved oxygen was the most important variable after the temperature in creating the ES50 distribution models with deltaAIC = 0.24 and 32.6% deviance. Despite slightly higher deltaAIC values for depth (deltaAIC = 0.60 and 32.4% of the variation) and pH (deltaAIC = 0.67 and 32.5% of the variation) models, they remained statistically significant in ES50 species richness (Table 3 S2 ).

The results for deep-sea communities demonstrated that the model based on the combination of environmental factors was the best-fitted model responsible for creating both species (deltaAIC = 0.00 and 89.3% of the variation) and ES50 (deltaAIC = 0.00 and 46.2% of the variation) richness patterns. In the next rank, depth ((deltaAIC = 6.06 and 89.1% of the variation), temperature (deltaAIC = 6.22 and 89% of the variation), and pH (deltaAIC = 6.97 and 89% of the variation) models were the most important variable to shape species richness. (Tables 4 and 5 S2).

GLM analysis selected the best-fitted model for all amphipod assemblages within each 5° latitudinal band. The findings highlighted spatial autocorrelation (deltaAIC = 0.00) as a significant predictor of species richness and ES50 within the 5-degree latitudinal band. Respectively, the chlorophyll (deltaAIC = 35.91) and salinity (deltaAIC = 3.36) models were the most responsible variables on species richness and ES50 patterns in 5°latitude bands (Tables 6 and 7 S2).

## Discussion

In recent decades, various studies have explored the distribution patterns of living organisms across latitudes and proposed bimodal patterns of latitudinal species richness at the global level on various taxa^21,52^. Similarly, Arfianti and Costello^19^ suggested a bimodal pattern for ES50, alpha, and gamma amphipod species richness against 5° latitudinal bands. However, these studies overlooked significant ecological and depth habitat variations, potentially influencing marine organism distributions and diversity. Here, we have categorized the data by shallow-water and deep-sea, as well as benthic and pelagic, to document the variations in species richness patterns against latitude among different depths and habitat types. Although a marginal bimodality pattern could be seen for the shallow benthic group, the results of the dip-test did not detect any significant bimodality in the species richness patterns. It seems that the bimodal pattern in previous studies was mostly affected by the size of the database^53^ and the sampling bias in equatorial regions^54^. Our dataset was three times bigger than the previous study^19^ by having 1,548 species and 1,300,216 occurrence records (76% of the present database) and we had sampling records from 178 new localities that never discovered in the previous studies in the Persian Gulf and the Gulf of Oman. We found that *Liljeborgia* Spence Bate, 1862 as the most widespread amphipod instead of *Themisto gaudichaudii* as reported by Arfianti and Costello^19^. The most dominant species was *Corophium volutator* in our dataset in contrast to*Monoporeia affinis* recorded by Arfianti and Costello^19^. The present study highlights the tropical regions, where results revealed significant alpha diversity among shallow pelagic species, particularly in the Caribbean Sea. The ES50 patterns further confirmed high biodiversity in several tropical areas. However, these findings contrast with Ritter and Bourne^11^ who mentioned temperate regions as the primary hotspots for amphipod biodiversity. In the earlier study, Arfianti and Costello^19^ did not highlight the tropical region as a hotspot for amphipods, but they mentioned that by adding data from the tropical and subtropical regions, it is expected to find new endemism regions. In the present study, data from the Persian Gulf and Gulf of Oman in addition to new tropical regions’ data submitted in GBIF and OBIS in recent years strengthened our tropical data. Recent studies support the high biodiversity in the tropical regions^7,19,32,55^. Higher potential of speciation, decreasing extinction rate, specialized niche^56,57^, and the existence of topographic complexity, large semi-enclosed seas, and islands^32^ were suggested as reasons for the high biodiversity in tropical regions.

The results of the present study showed that classifying data based on depth and ecology results in different diversity patterns, both in the hexagonal grid and 5° latitude bands. The role of depth in the distribution of marine organisms differs in epipelagic and deeper parts. In the epipelagic zone temperature, primary productivity, and global climate change correlate with depth^58^, however, the effect of depth in the mesopelagic and deeper zones is not very sensible due to decreased correlation with other environmental variables^59^. Results of the GAM, PCA, and Pearson analysis confirmed that different environmental variables drive diversity in shallow and deep-sea habitats. In shallow waters, temperature, chlorophyll, and primary productivity were the main drivers. The role of temperature in shaping biodiversity patterns in shallow waters was mentioned by Saeedi et al.^22^, in razor clams, Wang et al.^60^, in Bacteria, and Hirai et al.^61^, in copepods. In contrast, GAMs results showed the combinations of all environmental models, followed by salinity and dissolved oxygen shaped amphipod diversity in the deep sea, certainly due to a stable environment and complicated topology in the deep-sea realm^62^. A combination of variables such as sediment structure, oceanic currents, and carbon fluxes was reported by Danovaro et al.^63^, and Saeedi et al.^32^, as main drivers for shaping deep-sea diversity. In the northern North Atlantic and Nordic seas, Lörz et al.^64^, found depth, temperature, and salinity as one of the main environmental drivers of deep-sea benthic amphipod distribution. In other peracids, the amount of chlorophyll, silt, and total organic matter was reported as the main environmental drivers in the mesopelagic zone^59^. Our findings suggest that amphipod biodiversity decreases with rising temperatures and diminishing oxygen levels. However, it is crucial to account for species-specific adaptations, as certain amphipod species are known to thrive in environments characterized by low oxygen availability^65^ and elevated temperatures^66^.

We also found different diversity patterns across various ecological groups. The highest benthic biodiversity was reported from the continental shelf while the greatest pelagic amphipods were observed in tropical regions. Burridge et al.^67^, similarly reported a higher biodiversity of hyperid amphipods in the subtropical region and noticed that upwelling areas in the tropical zone caused higher pelagic diversity. The revealed different patterns for ecological groups reflect distinct drivers for them. Based on the energy-richness theory, areas with upwelling experience increased species richness of crustacean plankton, including pelagic amphipods. In contrast, the distribution of benthic species is affected by depth^24^, temperature, and water characteristics such as sedimentology, wave dynamics, and tidal dynamics^30,31,68^. This variation in effective environmental conditions between pelagic and benthic habitats highlights the importance of considering ecological context when studying marine species diversity.

Traditionally, the distinction between the ecological groups was not considered in amphipod phylogeographic studies. Two hypotheses on amphipod diversification considered distinct processes and origin time. The paleozoic origin hypothesis suggests that the limited distribution range of benthic amphipods, due to their direct development, was shaped by historical climatic changes (e.g., temperature shifts) and geographic events (e.g., plate tectonics)^13,69,70^. In an alternative scenario, the Cenozoic origin hypothesis proposes that the global ecological changes during the Cenozoic era created favorable conditions for diversification. Decreasing the ocean temperature and rising oxygen in the marine environment at that time provided proper conditions for the diversification of sensitive amphipods to temperature and oxygen^17^. The present study focused on the current species richness patterns without direct historical evidence to test these hypotheses. However, the similarity of amphipod assemblages in the defined realm by Spalding et al.^47^, in coastal areas could present a new perspective on amphipod biogeographic patterns. In general view, the cluster of pelagic species is more compatible with climate regions on the earth and supports the importance of ecological conditions in shaping present amphipod distribution. The cluster of benthic species supported the Paleozoic origin hypothesis by dividing benthic assemblages into Gondwana and Laurasia lands. However, previous studies on amphipods proposed Australasian coasts as a unique region^13,19^, our result confirmed Costello et al.^71^, to divide Australasian coasts into two distinct realms including; Temperate Australia and the center Indo-Pacific Ocean. Data from the Persian Gulf and the Gulf of Oman and other regional data created a distinct clade of the Western Indo-Pacific region separated from other Indo-Pacific realms. Characterization of Western Indopacific supports the effect of tectonic events on amphipod diversification which was mentioned by Myers and Lowry^13^ for this region.

In conclusion, our findings did not approve a bimodal pattern for global latitudinal amphipod species richness pattern however detected different patterns for ecological and depth groups. We identified temperature, chlorophyll, and primary productivity as key drivers in the shallow waters, not the deep sea. In contrast, the deep sea is influenced by a combination of environmental variables, including dissolved oxygen as we hypothesized. Additionally, our study revealed differences in species composition patterns between pelagic and benthic groups, with notable biodiversity hotspots identified in the tropical regions. 99% of the sampling database that was used in the current project belonged to unprotected areas and provided information about amphipod biodiversity from unknown areas (Fig. 1 S2). By understanding these distribution patterns of amphipods as umbrella species, policymakers can prioritize conservation efforts and develop effective management strategies to protect marine ecosystems, especially in the face of global warming.

## Electronic Supplementary Material

Below is the link to the electronic supplementary material.

Supplementary Material 1

Supplementary Material 2

## Data Availability

The data used in this study are available from open databases OBIS, GBIF, and sampling data is accessible from https://ipt.iobis.org/obis-deepsea/resource? r=amphipod_persiangulf .
