# Supplementary material for: Exploring latitudinal gradients and environmental drivers of amphipod biodiversity patterns regarding depth and habitat variations
Source: Sci Rep. 2024 Dec 18;14:30547. doi: 10.1038/s41598-024-83314-6 (PMC11655948; doi:10.1038/s41598-024-83314-6)
Supplement: Supplementary file 1 — Supplementary Material 1 [file 41598_2024_83314_MOESM1_ESM.docx]

**Table S1.** Datasets used in this study were extracted from OBIS.

| **id** | **citation** |
| --- | --- |
| 0c1cb7e9-c7d1-4643-b245-daa8839a183f | Marine Biological Association of the UK (MBA); (2016): DASSH: The UK Archive for Marine Species and Habitats Data |
| e2c4a839-89b5-484f-a63c-df89c2f9f974 | NA |
| d52444db-3aee-4ac3-8181-b8952fe53821 | The archive for marine species and habitats data (DASSH) (2021): Marine Recorder Snapshot extract of surveys entered by The archive for marine species and habitats data (DASSH). v2.1. Marine Biological Association. Dataset/Samplingevent. https://doi.org/10.17031/myrqac |
| e668dcb3-24cd-4d87-8835-e14d1961cccd | Hummel H. 2004: BIS dataset of the south-western part of Netherlands (1985-2004). Netherlands Institute of Ecology; Centre for Estuarine and Marine Ecology, Netherlands. Metadata available at http://mda.nioo.knaw.nl/imis.php?module=dataset&amp;dasid=599 |
| d2916eda-91c1-4c15-8dab-51da044595a4 | Galbraith M (2021): Zooplankton data from central and northern Strait of Georgia. v1.4. Fisheries and Oceans Canada. Dataset/Samplingevent. http://ipt.iobis.org/obiscanada/resource?r=zoopl_sofg&amp;v=1.4 |
| a9a3bdc6-209f-4c66-aafd-ce5271cb63b3 | Cooper et al. (2017). RSMP Baseline Dataset. Cefas, UK. V1. https://doi.org/10.14466/CefasDataHub.34 |
| 41a50544-957c-4193-8bbf-25a25f749e85 | NA |
| 80479e14-2730-436d-acaa-b63bdc7dd06f | Baranova, O.K, T.D. O'Brien, T.P. Boyer and I.V. Smolyar (2009). Plankton data. Chapter 16 in Boyer, T. P., J. I. Antonov , O. K. Baranova, H. E. Garcia, D. R. Johnson, R. A. Locarnini, A. V. Mishonov, T. D. O'Brien, D. Seidov, I. V. Smolyar, M. M. Zweng, 2009. World Ocean Database 2009. S. Levitus, Ed., NOAA Atlas NESDIS 66, U.S. Gov. Printing Office, Wash., D.C., 216 pp., DVDs |
| 7d92856b-a7fe-4e99-89c9-cc1d56354134 | <p>  Mackie, A.S.Y., James, J.W.C., Rees, E.I.S., Darbyshire, T., Philpott, S.L., Mortimer, K., Jenkins, G.O. &amp; Morando, A., 2006. The Outer Bristol Channel Marine Habitat Study. - Studies in Marine Biodiversity and Systematics from the National Museum of Wales. BIOMÃ”R Reports 4: 249 pp. &amp; Appendix 228 pp.</p> |
| 28b3b579-c7d1-43ad-8cf6-3e5d6b3b2016 | Stockholm University, University of Gothenburg, Linnaeus University , Swedish Agency for Marine and Water Management, Swedish Environmental Protection Agency and Swedish Meteorological and Hydrological Institute (2017). SHARK - National Epibenthos monitoring in Sweden since 1992. |
| ce3c5c7d-daa0-42ed-9cdb-7100b1274b55 | Bernier D, Boulanger M, Bourdages H, NozÃ¨res C, Vanier C, Isabel L (2023). DFO Quebec Region Ecosystemic bottom trawl surveys 2004-2022. Version 3.6. Fisheries and Oceans Canada. Samplingevent dataset. http://iobis.org/mapper/?resource_id=2542 |
| df6977ea-a704-4c48-bd46-ebbbb95e0bbc | Natural Resources Wales (NRW) (2021): Marine Recorder Snapshot extract of surveys entered by NRW. v2.0. Marine Biological Association. Dataset/Samplingevent https://doi.org/10.17031/b3efts |
| ec9df3b9-3b2b-4d83-881b-27bcbcd57b95 | Josefson, A.; Rytter, D.; Department of Bioscience - AU, Denmark; (2015): Danish benthic marine monitoring data from ODAM. |
| acd6f1b1-2e9b-48a6-b022-cee41b28f3b1 | (2023): Port Phillip Bay Environmental Study Data 1992-1996 - benthic records. v1.3. CSIRO National Collections and Marine Infrastructure (NCMI) Information and Data Centre (IDC). Dataset/Samplingevent. https://www.marine.csiro.au/ipt/resource?r=ppbes_benthic&amp;v=1.3 |
| 89e23fc8-3f61-4480-9de3-358fe6eefe0b | National Museum of Natural History, Smithsonian Institution NMNH Invertebrate Zoology Collection Database. National Museum of Natural History, Smithsonian Institution, 10th and Constitution Ave. N.W., Washington, DC 20560-0193, 2001, Version 3.2.04 (0802221). |
| 9c8a2ac6-f394-4fa1-981e-227fa7b14675 | ICES Environmental Database (DOME), Phytobenthos community. Available online at http://dome.ices.dk. ICES, Copenhagen. Consulted on yyyy-mm-dd. |
| 386ac1f4-922d-4991-ada2-0ce50dfd3ba7 | Addinck, W. &amp; de Kluijver, M. (2003). North Sea observations of Crustacea, Polychaeta, Echinodermata, Mollusca and some other groups between 1986 and 2003. Expert Centre for Taxonomic Idenditification (ETI), the Netherlands. |
| 511a64f5-54c0-4ccf-8c80-b7adbd5adff5 | Rumohr, H. Historical quantitative benthos grab samples from the Southern Baltic Sea - Polish data. University Kiel, Christian-Albrechts-University Kiel; Leibniz Institute of Marine Sciences; Marine Ecology Division; Benthos Ecology Section. |
| cad31b12-0f59-4abf-9109-cb09ea40c297 | Rumohr, H. Historical quantitative benthos grab samples from the Southern Baltic Sea - German data. University Kiel, Christian-Albrechts-University Kiel; Leibniz Institute of Marine Sciences; Marine Ecology Division; Benthos Ecology Section. |
| 152259dc-9c20-4c1a-9644-8e4b509d4f73 | Buhl-Mortensen, L. (2014). MAREANO - Base-line mapping of hyperbenthic crustacea fauna obtained with RP-sledge. Institute of Marine Research, Norway https://doi.org/10.15468/gecvl4 |
| f7e3baa2-48c9-47d9-9219-0a457615d610 | DFO. (2016). Central and Arctic Multi-Species Stock Assessment Surveys   Version 6 In OBIS Canada Digital Collections. Bedford Institute of Oceanography, Dartmouth, NS, Canada. Published by OBIS, Digital http://www.iobis.org/. Accessed on â€“INSERT DATE |
| 43b58971-4fda-4210-a6ef-654fe0ce226d | NOAA (2018): National Benthic Inventory. v1. GBIF Secretariat. Dataset/Occurrence. https://nccos-coastalscience-products-web-ipt.azurewebsites.net/ |
| d28b4411-1710-4b08-9ec7-99b8c68ae4b2 | Swedish county administration boards, Swedish municipalities, Swedish coalitions of water conservation, Swedish companies and Swedish Meteorological and Hydrological Institute et.al.(2021). Regional monitoring, recipient control and monitoring projects of zoobenthos in Sweden since 1972. https://doi.org/10.15468/cesssx |
| e69be30f-84e8-4c21-a7ce-9fb4fb14ce6a | SeaSearch (2021): Marine Recorder Snapshot extract of surveys entered by SeaSearch. v2.0. Marine Biological Association. Dataset/Samplingevent https://doi.org/10.17031/yq0gbg |
| de2825af-7d83-4585-b6cb-118cff3774cd | Estonian Marine Institute, University of Tartu (2019): Benthic fauna in Estonian territorial waters 1993-2016. |
| 6c80820b-61d2-4c69-9f3b-9564c014e1f9 | Australian Antarctic Data Centre. RMT Trawl catch from the 1990/91 V6 AAMBER2 voyage. |
| 4948203e-5c4f-4454-9e49-074f408afb9b | Hellenic Centre For Marine Research, MedOBIS - Mediterranean Ocean Biodiversity Information System. Hellenic Centre for Marine Research; Institute of Marine Biology and Genetics; Biodiversity and Ecosystem Management Department, Heraklion, Greece. Http://www.medobis.org/ |
| e7c86904-aac7-4a17-a895-99a54c430d80 | Northeast Fisheries Science Center, National Marine Fisheries Service, NOAA, U.S. Department of Commerce. 2010. NEFSC Benthic Database. Northeast Fisheries Science Center, 166 Water Street, Woods Hole Laboratories, Woods Hole, MA 02543.Retrieved from http://www.usgs.gov/obis-usa/ |
| c87cb4d8-0757-442f-9166-fbc0489b9149 | Natural England (NE) (2021): Marine Recorder Snapshot extract of surveys entered by Natural England. v2.0. Marine Biological Association. Dataset/Samplingevent. https://doi.org/10.17031/thn0xd |
| e4234681-4822-4490-9113-bdac90911dca | Stockholm University, Gothenburg University, UmeÃ¥ University, Linnaeus University, Swedish Agency for Marine and Water Management, Swedish Environmental Protection Agency and Swedish Meterological and Hydrological Institute (2020). National zoobenthos monitoring in Sweden since 1971. https://doi.org/10.15468/fggzdr |
| efb1d61c-6de4-489a-bcb0-3dc17ddc2778 | Serejo, C.S.; Cardoso, I.A.; Moura, M.G.P.; Ferreira, B.C.A. (2014) ColeÃ§Ã£o de Crustacea do Museu Nacional da Universidade Federal do Rio de Janeiro, 8654 registros (atualizado em 29/05/2014) |
| 9ad0f8eb-b983-418d-b881-3b672d9e0005 | Melrose, C. 2010. Continuous Plankton Recorder Dataset (NOAA) - Zooplankton. National Oceanic and Atmospheric Administration (NOAA). |
| d69a1913-4536-4e23-ae6b-911c6359cc61 | See metadata record http://data.aad.gov.au/aadc/metadata/metadata_redirect.cfm?md=AMD/AU/AADC-00099 |
| 256fd492-824a-44a8-9998-4dc911d40680 | Lavaniegos Bertha E. (2020). Hyperiid amphipod abundances from the California Current (24-32Â°N). SEANOE. https://doi.org/10.17882/75270 |
| 2e9e9830-d7cf-43f3-ba38-12ac06a7c0ba | Pugh, P. Discovery Collections Midwater Database. National Oceanography Centre, Southampton SO14 3ZH, U.K, 2000. |
| d1001fdc-d4a2-4d01-bcd2-2e122f2ca537 | NA |
| e981eab6-f849-4891-8fac-495852829456 | Johns D, Broughton D (2019): The CPR Survey. v1.0. Marine Biological Association. Dataset/Samplingevent http://doi.dassh.ac.uk/data/1629 |
| 726e33e7-6415-45ae-9d20-7c9a60e01343 | Rumohr, H., Historical benthosdata from the North Sea and Baltic Sea from 1902-1912. Christian-Albrechts-University Kiel; Leibniz Institute of Marine Sciences; Marine Ecology Division; Benthos Ecology section, Kiel, Germany. |
| d2caf73d-7e52-4113-b2f6-b89b439a9a2f | Joint Nature Conservation Committee (JNCC) (2021): Marine Recorder Snapshot extract of surveys entered by JNCC. v2.0. Marine Biological Association. Dataset/Samplingevent https://doi.org/10.17031/mehqrq |
| ce1d93f3-8b0f-4ee7-9a4d-0393a6ec7fea | Van Guelpen, L., 2016. Atlantic Reference Centre Museum of Canadian Atlantic Organisms - Invertebrates and Fishes Data. Version 4 In OBIS Canada Digital Collections. Bedford Institute of Oceanography, Dartmouth, NS, Canada. Published by OBIS, Digital http://www.iobis.org/. Accessed on â€“INSERT DATE |
| c5ce6e76-4ad6-4fce-a683-f6bb01c70955 | Inomata K. Kawagucci S. Miwa T. and Yamamoto H (2023) Environmental baseline data in abyssal plain off Minami Torishima based on 16S rRNA gene, 18S rRNA gene, physicochemical parameters and meiofauna community, and megafauna occurrence. https://doi.org/10.48518/00017 |
| 2d2deee0-b592-4426-ab82-503b6bccc00d | Swedish county administration boards, Swedish municipalities, Swedish coalitions of water conservation, Swedish Meteorological and Hydrological Institute, et al (2017). SHARK - Regional monitoring and monitoring projects of Epibenthos in Sweden since 1994 |
| 0387adc4-c0b6-4072-b5f2-b819b4cfa30d | NatureScot (2021): Marine Recorder Snapshot extract of surveys entered by NatureScot. v2.0. Marine Biological Association. Dataset/Samplingevent. https://doi.org/10.17031/pqhlyg |
| 33452cc4-13c7-4355-894f-4a8833302841 | Hall-Spencer J.,Grall J., Gerovasileiou V.,Mavraki D.,Paranou P., Baily N.,Nikolopoulou S.,(2018): BIOMAERL.Maerl Biodiversity.Functional Structure And Antropogenic Impacts (1996-1998).Hellenic Center for Marine Research. |
| 0912e81c-c5ac-4a53-b719-a9ae6535f895 | Meurisse, L., Semal, P. (2020) Royal Belgian Institute of Natural Sciences Crustacea collection |
| 7e4228e5-a962-4b01-952f-7bf33e213a9c | Sameoto, D.D., Kennedy, M., Spry, J.S, Spry, J.M. (2013). Zooplankton datasets collected using the BIONESS sampler, ring nets and an Icelandic high speed sampler, 1967-2006. OBIS Canada Digital Collections. Published by OBIS http://www.iobis.org/. Accessed on â€“INSERT DATE |
| cfa6809d-50cf-4f29-99d1-9e9bbf39c59c | Marine Institute (2020), Irish Benthos monitoring as part of the Water framework directive since 2012 |
| 82c51e92-e66f-44ca-a02a-b6071c7e188f | Craeymeersh J., P. Kingston, E. Rachor, G. Duineveld, Carlo Heip, Edward Vanden Berghe, 1986: North Sea Benthos Survey. |
| 8b681c9d-f925-4a11-930d-d37c07b0560e | NA |
| 6f7372ff-0199-4e51-9728-69cb536aaa14 | Kennedy, M.K., St-Pierre., I. 2011. BioChem: Atlantic Zone Monitoring Program (AZMP) Quebec Region zooplankton collection. OBIS Canada Digital Collections. OBIS Canada, Bedford Institute of Oceanography, Dartmouth, Nova Scotia, Canada, Version 1, Digital, retrieved from http://iobis.org/. |
| acb3f792-0f33-42e1-a2a8-28eb681e8cdb | Citation_Information: Originator: G.E. Hopky, M.J. Lawrence and D.B. Chiperzak Publication_Date: 1994 Title: NOGAP B2, Data on the Meio- and Macrobenthos, and related bottom sediments from Tuktoyaktuk Harbour and Mason Bay, N.W.T., March, 1985 to 1988 Geospatial_Data_Presentation_Form: document Series_Information: Series_Name: Canadian Data Report of Fisheries and Ocean Science 939Issue_Identification: |
| 364e5a12-8028-4329-be50-fc548c2d6ce4 | Southwestern Pacific OBIS (2014). Biological observations from the Discovery Investigations 1925-1935. Southwestern Pacific OBIS, National Institute of Water and Atmospheric Research (NIWA), Wellington, New Zealand, 33337 records, Online http://nzobisipt.niwa.co.nz/resource.do?r=discovery_reports released on January 23, 2015. |
| 9b7acd33-4a40-4043-9383-e8c73b7ed7ba | Zoppi de Roa E, Palacio CÃ¡ceres M, Casanova E, Scott-FrÃ­as J (2022): Zooplankton of Morrocoy National Park 2000-2002. v1.9. Caribbean OBIS Node. Dataset/Samplingevent. https://ipt.iobis.org/caribbeanobis/resource?r=zoo-morrocoy-2000&amp;v=1.9 |
| 747eedb6-dca9-4c9a-849d-618bffeaa18f | Dounas C., 1988: Benthos Cretan Continental Shelf. Hellenic Centre for Marine Research, Greece |
| d556b9d4-7625-4aa2-894d-441eabae47f7 | Holte, B. (2014). MAREANO - Base-line mapping of fauna obtained with grab. Institute of Marine Research, Norway https://doi.org/10.15468/dlaxsw |
| 8dbd0669-92d1-41ef-a5db-527bc6aa1ec9 | Cusson M (2018): Biodiversity of benthic assemblages on the Arctic continental shelf: historical data from Canada (1955 to 1977). v1.4. Canadian node of the Ocean Biogeographic Information System (OBIS Canada). Dataset/Occurrence. http://ipt.iobis.org/obiscanada/resource?r=cusson_arcticbenthos&amp;v=1.4 |
| fbfec490-ee3a-404c-80b2-9432345bcb15 | NIOZ (2023) Benthos Westerschelde 1963-2012 |
| 5ad6c4ff-fb89-4edf-b7b0-212671fb27cf | Sumner, F. B., R. C. Osborn, L. J. Cole, and B. M. Davis. A biological survey of the waters of Woods Hole and vicinity. Bulletin of the U.S. Bureau of Fisheries. 1911. 31: 1-860 |
| 8debaccf-a981-4c77-8fad-ffb443dcf1f4 | Steffani, N; Pulfrich, A. (2013). De Beers Marine Benthic Monitoring Programme. Dataset published by AfrOBIS; consulted via iOBIS |
| 88bb5351-cbed-4448-abf8-fdeae8b1abaf | KÃ¼rzel, Kaiser, Brix, Colemann, Tandberg, Oldeland, LÃ¶rz (2022): Amphipod distribution data from the North Atlantic and Artic waters compiled from literature records published in 1931-2018. |
| a4695be4-9914-4cc2-ac24-201bd868db57 | Fonda-Umani, S. (2014). Macrobentos North Adriatic-ALPE ADRIA Project. University of Trieste; Marine Biology Laboratory https://doi.org/10.6092/046b3834-9c72-4e2d-920f-8db346c90407 |
| 9378019b-6657-4c21-81cd-1d2f19e30065 | dos Santos A, Niza H, Cunha M E, Quintela M D F (2022): Planktonic Production Cycles and their Relationship with Fishing Resources on the Portuguese Coast |
| d69036d6-00c8-445c-89aa-f9149cb1de31 | De Broyer C, Lowry J.K , Jazdzewski K. and Robert H. (2007). Catalogue of the Gammaridean and Corophiidean Amphipoda (Crustacea) of the Southern Ocean with distribution and ecological data. In: De Broyer C. (Ed.), Census of Antarctic Marine Life, Synopsis of the Amphipoda of the Southern Ocean. Volume 1. Bulletin de l'Institut Royal des Sciences Naturelles de Belgique , Biologie, 77 (suppl. 1), 1-325. |
| a2426321-7817-4086-ace6-f16ce16a3653 | LÃ©vesque D, Roux M, Robillard A (2023). DFO Quebec Region Invertebrate assemblages and submerged aquatic vegetation in coastal areas of the St. Lawrence Estuary and Gulf (north shore) using a drop photo camera system.. Version 1.9. Fisheries and Oceans Canada. Samplingevent dataset. https://ipt.iobis.org/obiscanada/resource?r=dl_cpd_ppo&amp;v=1.9 |
| 3232e8ce-2581-4bc3-8641-75d01311bcec | Sezgin, M. 2005. Crustacea data from 2004-2005. Sinop University Fisheries Faculty (SNU-FF). |
| 49d1b62c-124a-4854-b878-b83de130eb23 | Ocean Survey 20/20 (2013). International Polar Year and Census of Antarctic Marine Life Ross Sea voyage (TAN0802) biodiversity data. Southwestern Pacific OBIS, National Institute of Water and Atmospheric Research, Wellington, New Zealand, 8748 records, Online http://nzobisipt.elasticbeanstalk.com/resource.do?r=mbis_caml released on Dec 12, 2013. |
| 132ab7ea-1b4b-46ea-8f5f-6b8da98db2be | Marine Ecological Surveys Ltd. - UK (2009). Macrobenthos from the eastern English Channel in 1999 and 2001. |
| 837dff49-b281-4d12-a2b0-042095ff2945 | Iken K, Bluhm B (2022): US-CAN-Transboundary_BOEMproject_Beaufort Sea_Epibenthos_2012_2013_2014. v1.1. Deep-sea OBIS node. Dataset/Occurrence. https://ipt.iobis.org/obis-deepsea/resource?r=us-can-transboundary_boemproject&amp;v=1.1 |
| 43d19bf8-4d49-413b-bedb-4fa4547714fa | Finnish Environment Institute SYKE; (2018); Finnish Baltic Sea benthic monitoring, POHJE database |
| 3d08e18e-6146-4e27-ab0e-03504a65cbd4 | Houbin, C; Hoebeke, M (2019): Roscoff inventories: marine fauna and flora since 1800 https://doi.org/10.21411/qhtc-a855 |
| e13f0a51-f810-45d6-9503-64ecd7bedd70 | NIWA (2014). 2004 Ross Sea Biodiversity Survey (BIOROSS) data. Southwestern Pacific OBIS, National Institute of Water and Atmospheric Research, Wellington, New Zealand, 4093 records, Online http://nzobisipt.niwa.co.nz/resource.do?r=mbis_caml released on June 18, 2014. |
| 8acba7e7-2e50-4490-8328-b78a30472508 | ICES Environmental Database (DOME), Zoobenthos community. Available online at http://dome.ices.dk. ICES, Copenhagen. Consulted on yyyy-mm-dd. |
| fd1d4384-f317-47ee-aa8c-c20347bae849 | NA |
| 7fe97cd7-1d94-475a-bbec-144fbee1efd8 | Wolff G, Rowe G, Kennicutt M, Presley B, Bernhard J, Morse J, Montagna P, Nowlin W, Bryant W, Wade T (2023). The Deepwater Program: Northern Gulf of Mexico Continental Slope Habitat and Benthic Ecology - DgoMB: Macrofauna. Version 1.2. United States Geological Survey. Occurrence dataset. https://ipt-obis.gbif.us/resource?r=dgomb_macrofauna&amp;v=1.2 |
| 391a1608-87a7-4f70-950e-86643643f611 | Partridge V (2015): Acadia University: Invertebrate species distribution during winter conditions at the Windsor mudflat, an intertidal mudflat located in the upper Bay of Fundy, January - June, 1996. v1.2. Ocean Biogeographic Information System (OBIS Canada). Dataset/Occurrence. http://iobis.org/mapper/?resource_id=XXXX |
| ea1618ec-00de-41e8-a0f5-56b6f526780e | NIOZ (2023) Benthos Oosterschelde 1959-2015 |
| c7d062de-eeb2-42e8-843a-7086e0d14420 | University of Dundee (2018): NEODAAS- NERC Earth Observation Data Acquisition and Analysis Service. v2.0. Tropical and Subtropical Western South Atlantic OBIS. Dataset/Occurrence. http://doi.org/10.25607/cvamrq |
| e84e4864-bb77-486d-9940-d784014dd2fc | NA |
| 1ad423c7-dba3-426f-b814-f0808413a708 | Galbraith, Moira. (2013) DFO Pacific IOS zooplankton database - Line P. Version 1 In OBIS Canada Digital Collections. Bedford Institute of Oceanography, Dartmouth, NS, Canada. Published by OBIS, Digital http://www.iobis.org/. Accessed on â€“INSERT DATE |
| 53092237-536e-4ad2-bb28-00fc467dbd26 | Teaca, A.; Begun, T.; Muresan, M.; National Research and Development Institute for Marine Geology and Geoecology â€“ GeoEcoMar, Romania (2016): Historical benthos data from the Romanian Black Sea Coast between 1959 and 1961 |
| c4e7e0d6-aef2-480f-8157-134bd1c6b229 | Alfred Wegener Institut fÃ¼r Polar- und Meeresforschung; Senckenbergische Naturforschende Gesellschaft. - Germany (2015). Macrozoobenthos data from the southeastern North Sea in 2000. |
| 31931936-77cc-4562-a775-b1553fe7ffe8 | (2023): Port Phillip Bay Environmental Study Data 1992-1996 - infauna records. v1.4. CSIRO National Collections and Marine Infrastructure (NCMI) Information and Data Centre (IDC). Dataset/Occurrence. https://www.marine.csiro.au/ipt/resource?r=ppbes_infauna&amp;v=1.4 |
| 81903d9a-f9f2-4bee-ab8c-07609b308e6b | Kendrick G (2023): Invertebrates of the Recherche Archipelago , Western Australia (2002). v1.3. CSIRO National Collections and Marine Infrastructure (NCMI) Information and Data Centre (IDC). Dataset/Occurrence. https://www.marine.csiro.au/ipt/resource?r=frdc_recherche_invertebrates&amp;v=1.3 |
| ac30f88b-7d4d-4f3c-8204-ac362dc0feb5 | Sorbonne UniversitÃ©/CNRS - Institut de la Mer de Villefranche (IMEV), Sorbonne UniversitÃ©/CNRS - Laboratoire d'OcÃ©anographie de Villefranche (LOV); 2020; Plankton community in Bongo net, MOOSE-GE cruises, North-Western Mediterranean Sea. https://dx.doi.org/10.14284/480 |
| f6e5b0ee-d645-40fc-a433-17fd8965c941 | JODC (2013) JODC Dataset. (Available: http://www.godac.jamstec.go.jp/bismal/j/JODC_J-DOSS. Accessed: YYYY-MM-DD). |
| 6126d698-3e12-4f10-8b05-65122298d02e | Agri-Food and Biosciences Institute (AFBI) (2019): Benthic bycatch data from non-native Crepidula fornicata dredge surveys in Belfast Lough from 2013-2017. v3.3. Marine Biological Association. Dataset/Samplingevent https://doi.org/10.17031/bhwln1 |
| 3d8430ee-0281-4271-8449-67394407bc23 | Madin, L. and E. Horgan. Zooplankton Sampled with 10m2MOCNESS Net in Georges Bank 1995-1999. March 10, 2006. U.S. GLOBEC JGOFS/GLOBEC Data Server. Woods Hole Oceanographic Institution, USA: U.S. GLOBEC Data Management Office. Retrieved from http://www.usgs.gov/obis-usa/. |
| 7176d725-4046-4faa-8647-94ecf4dd8304 | Beyst, B.; Fockedey, N.; Hostens, K.; Mees, J.; Marine Biology Research Group - Ugent: Belgium; (2004): Ecological hyperbenthic data of the Scheldt estuary: historical data (1988-2001). Marine Data Archive. |
| d104db68-63cf-4f34-b92a-54bd55a07394 | Jo Taylor J (2023): Museums Victoria Marine Invertebrates Collection. v1.12. CSIRO National Collections and Marine Infrastructure (NCMI) Information and Data Centre (IDC). Dataset/Occurrence. https://www.marine.csiro.au/ipt/resource?r=nmv_marine_inverts&amp;v=1.12 |
| 92b694e1-1534-4d37-8e11-3c2c6d2d5edc | Bustamante R (2023): Southern Surveyor Voyage SS 03/2005 Box Core Infauna Samples, Gulf of Carpentaria, Australia (2005). v1.1. CSIRO National Collections and Marine Infrastructure (NCMI) Information and Data Centre (IDC). Dataset/Samplingevent. https://www.marine.csiro.au/ipt/resource?r=csiro_ss200503_infauna&amp;v=1.1 |
| 27530a2b-da53-4f3b-98b4-83d4b3e73082 | GrÃ©mare, A., J.M. Amouroux, , C. Labrune, 1998: Redit dataset. Observatoire OcÃ©anologique de Banyuls-Sur-Mer; Laboratoire d'OcÃ©anographie Biologique, France https://dx.doi.org/10.14284/184 |
| 46c52e34-73b7-45e6-a2ac-742e4c2058c5 | ICES Environmental Database (DOME), Phytoplankton community. Available online at http://dome.ices.dk. ICES, Copenhagen. Consulted on yyyy-mm-dd. |
| f3019ee2-a2f8-4a5c-acd9-1a546b363a17 | NA |
| 2a9b0eb5-8000-44e9-b985-be11c228e343 | Chardine, J. 2011. BioChem: Zooplankton collected from the Bay of Fundy, 2002-2007. OBIS Canada Digital Collections. OBIS Canada, Bedford Institute of Oceanography, Dartmouth, Nova Scotia, Canada, Version 1, Digital, retrieved from http://iobis.org/. |
| c49997fd-7f9d-404d-af03-96c36b8dea9a | Kotwicki L., 1996: Bay of Puck dataset. Polish Academy of Sciences, Institute of Oceanology, Department of Marine Ecology, Poland |
| 2c90a322-c002-44a3-a76e-e67d90e76a83 | Rumohr, H., D. Fleischer, 2004: N3 data of Kiel Bay. Leibniz Institute of Marine Sciences, Marine Ecology Division, Germany https://dx.doi.org/10.14284/182 |
| a28d3c22-183e-4e5e-a9ee-38240fcd4241 | Hopky, G.E. Lawrence, M.J. and Chiperzak, D.B.; Central and Arctic Region, Department of Fisheries and Oceans, Winnipeg, Manitoba R3T 2N6. (2004) NOGAP B2; Zooplankton Data from the Canadian Beaufort Sea Shelf, 1986. Canadian Data Report of Fisheries and Aquatic Sciences: 923 |
| b99832a1-320a-48b0-8a54-a01409a321af | Dahle S., R. Palerud, N. Anisimova, 1992: Benthic fauna around Franz Josef Land. Akvaplan-niva, Norway. |
| 1701dd73-9a96-48c8-9049-e5b1ca3c6993 | Williams A (2022): South East Fishery (SEF) Ecosystem Study 1993-1996: Fish Diet Data. Australia (1993-1997). v1.11. CSIRO National Collections and Marine Infrastructure (NCMI) Information and Data Centre (IDC). Dataset/Occurrence. https://www.marine.csiro.au/ipt/resource?r=csiro_sef_diet&amp;v=1.11 |
| a145f369-b272-46f7-86bc-08412b1ee927 | Sirenko B.I., ed. 2001. List of species of free-living invertebrates of Eurasian Arctic seas and adjacent deep waters. In: Explorations of the fauna of the seas. 51(59). St.Petersburg: 1-132. |
| ab25396e-888b-4073-af34-4868ac1db8da | Nelson, John. (2014). DFO Pacific IOS zooplankton database - Zooplankton samples collected during cruises to the Canadian Arctic, 2006-2009. Version 1 In OBIS Canada Digital Collections. Bedford Institute of Oceanography, Dartmouth, NS, Canada. Published by OBIS, Digital http://www.iobis.org/. Accessed on â€“INSERT DATE |
| 52ccf89d-12a5-47c3-8b36-e3e9e6212c5b | Carin J. Ashjian, Robert G. Campbell, Harold E. Welch, Mari Butler,Donna Van Keuren. (2003) Annual cycle in abundance, distribution, and size in relation to hydrography of important copepod species in the western Arctic Ocean (SHEBA). Deep Sea Research, 50: 1235-1261 |
| 2c6db58f-ae91-4a17-9f0f-7db28506b94f | NIWA (2018): NIWA Invertebrate Collection. v1.1. The National Institute of Water and Atmospheric Research (NIWA). Dataset/Occurrence. https://nzobisipt.niwa.co.nz/resource?r=obisspecify&amp;v=1.1 |
| cd42b44b-2560-450d-88e7-70b5778d0194 | Darnis, GÃ©rald, Barber, David G., and Fortier, Louis. 2015. CASES: The pre-winter assemblages of southeastern Beaufort Sea. Version 1 In ArcOD Digital Collections. Institute of Marine Science University of Alaska, Fairbanks, AK, USA. Published by OBIS, Digital http://www.iobis.org/. Accessed on â€“INSERT DATE |
| 2c30e8ee-3068-437c-bf94-499e06cdd4e4 | Steffani, N; Pulfrich, A. (2013). De Beers Marine Namibia Benthic Monitoring Programme. Dataset published by AfrOBIS; consulted via iOBIS |
| 4f46f9d8-dc63-4546-9dd5-075df3d25e4a | iziko South African Museum - Crustacean Collection |
| 387148c5-6ab1-477d-b5f5-e95a0ef01a41 | GrÃ©goire B (2022): Biodiversity of the Planning for Integrated Environmental Response Coastal Survey in the St. Lawrence Estuary and Gulf (2017-2021). v1. Fisheries and Oceans Canada. Dataset/Samplingevent. https://doi.org/10.26071/OGSL-90C40DBA |
| 231dc661-d8d9-4d49-9ff0-45bd21a08541 | NIWA (2014). New Zealand fish and squid distributions from research bottom trawls. Southwestern Pacific OBIS, National Institute of Water and Atmospheric Research (NIWA), Wellington, New Zealand, 486781 records, Online http://nzobisipt.niwa.co.nz/resource.do?r=obisprovider released on May 8, 2014. |
| 4aaa85a5-6cfb-45bf-9ca4-ca431db88849 | Harris R. &amp; Widdicombe C. (2007). L4 Plankton Monitoring Programme. Plymouth Marine Laboratory (PML), United Kingdom. Hdl.handle.net/10255/dryad.20312 |
| a419c8da-35ed-4b62-9709-39b56369c44e | Nancy Jacobsen Stout, Linda Kuhnz, Lonny Lundsten, Kyra Schlining, Susan von Thun, (2002) Video Annotation and Reference System (VARS) database, Year 2000, Monterey Bay Aquarium Research Institute, Moss Landing, California USA, Database, www.mbari.org/vars |
| 55fdf3b0-744a-4a29-a81e-7d9884be1a91 | Broms, C. (2014). IMR Zooplankton Norwegian Sea. Institute of Marine Research, Norway |
| f6352e4d-31b5-4baa-99e1-d39646043e88 | Koninklijk Nederlands Instituut voor Zeeonderzoek (Texel), National Institute for Coastal and Marine Management (Ministery of Transport and Public Works) - 2001. Macrobenthos in the Dutch Sector of the North Sea 2000-2001. |
| 44d02422-9ead-4392-8e0b-39aaefe2ce01 | Hopky, G.E. Lawrence, M.J. and Chiperzak, D.B.; Central and Arctic Region, Department of Fisheries and Oceans, Winnipeg, Manitoba R3T 2N6. (1994) NOGAP B2; Zooplankton Data from the Canadian Beaufort Sea Shelf, 1984 and 1985. Canadian Data Report of Fisheries and Aquatic Sciences: 922. |
| af201b38-f32e-4213-a901-fe16c5423a2a | The Norwegian Oil Industry Association, 2001: Offshore reference stations, North/Norwegian sea. The Norwegian Oil Industry Association (OLF), Akvaplan-niva and Det Norske Veritas, Norway |
| 18b9f6fd-0cca-4938-a6fa-d6b375dfa716 | Tuaty-Guerra, M.; GaudÃªncio, M.J. (2021). Benthic fauna of the Southwest Alentejo and Vicentine Coast Natural Park (SW Portugal) collected in August 2011 https://dx.doi.org/10.14284/464 |
| f0153b4d-22d6-4368-b89f-ea7c8aa56e67 | De Boyer C (2020): A collection of Antarctic Phipoda. v1.1. Antarctic Biodiversity Information Facility (ANTABIF). Dataset/Occurrence. https://ipt.biodiversity.aq/resource?r=2438_mista_ant_phipoda&amp;v=1.1 |
| 1d198847-58cf-4350-9d2c-8c8f611083c4 | NA |
| cf2113ef-d5f0-4586-9fc6-4b208e0bc245 | Olivier M., Lilley M., Lombard F., Stemmann L. (2015) Temporal evolution of zooplankton, surface observations, in the Northwestern Mediterranean Sea. |
| a6c5a265-a845-4f8c-8759-b47f1fdf8b26 | Dewicke, A; Marine Biology Section (MARBIOL) â€“ Ugent, Belgium; (2014): Hyperbenthic communities of the North Sea |
| 15f11e0d-44ec-4cb0-97cf-d2098b72e3a7 | Mackie, A.S.Y., P.G. Oliver, E.I.S. Rees, 1991: BiomÃ´r 1 dataset. Benthic data from the Southern Irish Sea from 1989-1991. National Museum and galleries of Wales, Cardiff, UK. |
| 78f97784-2904-4e60-8aed-defe1e6b4b24 | Historical dataset of marine biological records, Institute of Biology of the Southern Seas, NAS Ukraine |
| 26de959f-8916-43cf-9472-170c28358a8e | Bakkeplass, K. (2014). IMR Macroplankton surveys. Institute of Marine Research, Norway |
| 58be7fc9-bef4-42db-9d4f-07106a711468 | Teaca A.; Begun T.; Gomoiu M.-T.; Secrieru; National Research and Development Institute for Marine Geology and Geoecology â€“ GeoEcoMar, Romania (2016): Macrobenthos data from the Romanian part of the Black Sea between 2003 and 2011 |
| 506723d9-6a80-4d9c-b7fa-5d11ccefea95 | Havermans et al (2010) Molecular Phylogenetics and Evolution 55 (2010) 202â€“209 lysianassoids |
| c606c47a-3892-4645-9521-630c9085e59f | NA |
| 420d5051-7d48-4c63-94d8-2553b9aad236 | Leibniz-Institut fÃ¼r Meereswissenschaften an der UniversitÃ¤t Kiel - Germany (2015). Amrum Bank and inner German Bight Benthos |
| 14fa3c3e-259c-4af9-9314-eee1dc3a119b | Hassel, A. (2014). MAREANO - Base-line mapping of epifauna obtained with Beamtrawl. Institute of Marine Research, Norway https://doi.org/10.15468/iomgfj |
| cd902fc8-f52b-4bda-95b8-a6782c1d0953 | Zeidler W. &amp; De Broyer C., 2009. Catalogue of the Hyperiidean Amphipoda (Crustacea) of the Southern Ocean with distribution and ecological data. Bulletin de l'Institut Royal des Sciences Naturelles de Belgique 79 (Suppl. 1): 1-104. |
| dfdd80fe-1f64-4926-bc75-a639b6ec5925 | Sorbonne UniversitÃ©/CNRS - Institut de la Mer de Villefranche (IMEV), Sorbonne UniversitÃ©/CNRS - Laboratoire d'OcÃ©anographie de Villefranche (LOV); 2020; Plankton community in RÃ©gent (680Âµm) net, Point B, Villefranche-sur-Mer, France https://dx.doi.org/10.14284/477 |
| f5cca63c-2842-4146-b095-7d34e7aef236 | Cecchetto M, JaÅ¼dÅ¼ewska A M, Guzzi A, Grillo M, Noli N, Cometti V, Schiaparelli S (2023). Distribution records of Amphipoda based on the collection stored at the Italian National Antarctic Museum (MNA). Version 1.4. Italian National Antarctic Museum (MNA, Section of Genoa). Occurrence dataset. https://ipt.biodiversity.aq/resource?r=mna_amphipoda&amp;v=1.4 |
| 4c94cee2-f2f0-4a7b-825a-967bd991ca68 | Espinosa-Leal L (2021): AnfÃ­podos_cimar22. v1.1. Universidad de ConcepciÃ³n e Instituto Milenio de OceanografÃ­a (IMO). Dataset/Samplingevent. http://ipt.iobis.org/esp-obis/resource?r=anfipodos_cimar22&amp;v=1.1 |
| d94f3ea4-4519-4eb0-9d03-5cfc1caab13f | The Norwegian Oil Industry Association, 2002: Offshore reference stations, Norwegian/Barents Sea. The Norwegian Oil Industry Association (OLF), Akvaplan-niva and Det Norske Veritas, Norway |
| d2121da0-aa03-49bc-a9ea-f6c2c02c837a | Australian Antarctic Data Centre, Marine fauna survey of the Vestfold Hills and Rauer Island, 1981-82 |
| f070a9b6-b425-4dae-b7ca-d492b717dcc7 | Sloan, N.A., Bartier, P.M., Austin, W.C. 2004. Gwaii Haanas Invertebrates (Living marine legacy of Gwaii Haanas II: Marine invertebrate baseline to 2000). Parks Canada-Technical Reports in Ecosystem Science. OBIS Canada, Bedford Institute of Oceanography, Dartmouth, Nova Scotia, Canada, Version 1, Digital, retrieved from http://iobis.org/. |
| fb3fe774-a1e5-45cd-ab4c-398bf7932a7d | Inomata K. Kawagucci S. Miwa T. and Yamamoto H (2023) Environmental baseline data in abyssal plain off Minami Torishima based on 16S rRNA gene, 18S rRNA gene, physicochemical parameters and meiofauna community, and megafauna occurrence. https://doi.org/10.48518/00020 |
| 10b213e6-a9c4-459e-a40c-ef9edc461b97 | Pyle R (2016). Bernice P. Bishop Museum. Version 8.1. Bernice Pauahi Bishop Museum. Occurrence dataset https://doi.org/10.15468/s6ctus accessed via GBIF.org on 2018-11-16. |
| 6963aa86-a154-459c-aeb0-0496668512f6 | Japan Agency for Marine-Earth Science and Technology (2016 onwards). JAMSTEC Marine Biological Samples Database. https://doi.org/10.48518/00001. Accessed on yyyy-mm-dd. |
| b339f72f-eb69-40c5-b60c-1a37aaffdfb7 | SWPRON (2014). Marine biological observation data from coastal and offshore surveys around New Zealand. Southwestern Pacific OBIS, National Institute of Water and Atmospheric Research (NIWA), Wellington, New Zealand, Online http://nzobisipt.niwa.co.nz/resource.do?r=mbis_nz |
| aa16d305-d413-4c4a-90be-b1ec3298d58d | NA |
| 60cfb9fb-dbbf-4fcf-86a7-dda5343aadee | LeBrasseur, R.J., Doidge, D.A., 1966. Stomach contents of salmonids caught in the Northeastern Pacific Ocean - 1959 &amp; 1960. Circular, Statistical series, Fisheries Research Board of Canada, Biological Station, Nanaimo, Canada, 21, Vol. 3, 67 pp. |
| ff1eca2d-d949-42ce-9cb2-95c26b61d198 | Isla A, Aierbe E, Bode A, GonzÃ¡lez-QuirÃ³s R, Nogueira E, ValdÃ©s L; IEO-CSIC (2022) Zooplankton abundance and biomass from the RADIALES time series programme (N-NW Spain) |
| 6674b962-d4ba-4379-991a-6989a9983191 | Knutsen, T.; Dalpadado, P. (2014). IMR Zooplankton Barents Sea. Institute of Marine Research, Norway |
| 0806f576-7862-463c-b4ba-54a53341be45 | Ocean Mineral Singapore Pte. Ltd., (2017). OMSPMN12017 Scavengers Senckenberg. Available : DeepData, International Seabed Authority https://data.isa.org.jm/ Accessed: [YYYY-MM-DD]. |
| 17ce69d0-2504-4ae3-bc87-16b096863f51 | Markhaseva, E.L., Golikov, A.A., Agapova, T.A., Beig, A.A. 1985 Archives of the Arctic Seas Zooplankton 1 |
| a77de2cc-0e3f-485b-8c87-eb0545423957 | NA |
| 64f3e2d5-9e2c-4e18-ad50-3847d62152db | NA |
| e168c19e-d9e9-447c-b077-1faa487df4a6 | These data are from the Australian Continuous Plankton Recorder (AusCPR) survey part of the Integrated Marine Observing System (IMOS) - IMOS is a national collaborative research infrastructure, supported by the Australian Government. |
| c697826c-1da7-4916-a387-e35008a90415 | Cochrane, S. (2001). Macrobenthos from the Norwegian waters. Akvaplan-niva, Norway. |
| a4236fe5-21ab-4faa-9221-ed8c65f92c4d | The Norwegian Oil Industry Association, 2000: Offshore reference stations, Finnmark. The Norwegian Oil Industry Association (OLF), Akvaplan-niva and Det Norske Veritas, Norway |
| b2606b6f-f6d0-47ea-8a56-d086b20c60be | Bachelet, G., 2004: Benthos Gironde Estuary. UniversitÃ© Bordeaux 1, Station Marine d'Arcachon, Laboratoire d'OcÃ©anographie Biologique, France |
| e1ca35fd-bbc4-4da9-a27c-fea1502a4a30 | Gutt, Sirenko, Arntz, Smirnov &amp; De Broyer, 2000 |
| c2d77503-e959-48e6-b34d-097d0ff66d42 | Antoniadou C. (1998). Macro- and megafauna from the North Aegean Sea from 1997-1998. Aristotle University of Thessaloniki, Department of Biology, Laboratory of Zoology, Greece. |
| 1764b492-c904-456e-bcf6-bedffc86a10b | Zooplankton abundance, vertical and horizontal distribution (MOCNESS system), April-June 2001-02, continental margin west of Antarctic Peninsula, GLOBEC. |
| 20f08cfa-97d1-406b-8723-50075ee006c4 | Horton T (2019): Scavenging Amphipods, Porcupine Abyssal Plain Sustained Observatory, North Atlantic, 1985-2016. v1.2. The Discovery Collections. Dataset/Samplingevent. http://ipt.iobis.org/obis-deepsea/resource?r=pap_scavenging_amphipods&amp;v=1.2 |
| e8f6f42e-0e33-4382-9998-9a178eac3191 | Romagnan, J.B., Jalabert, L., Llopiz Monferrer, N., Merland, C., Trudnowska, E., Brandao, M., Elineau, A., Dimier, C., Picheral, M., Searson, S., Kandels-Lewis, S., Tara Oceans Consortium Coordinators, Karsenti, E., Pesant, S., Irisson, J.O., Lombard, F., Stemmann, L., Gorsky, G.; (2020) Occurrence of Eukaryotes in the size fraction &gt;300 micrometres, collected worldwide during the Tara Oceans expedition (2009-2013) using a Multinet and analysed on a ZooScan imaging platform https://dx.doi.org/10.14284/517 |
| 6e85c8a3-0513-47f3-a380-c67c9e976d84 | NA |
| fedb2f96-5db0-406c-8a31-4c98702ae57c | Kendall, M., S. Widdicombe, 1999: Plymouth sound dataset. Soft sediment macrobenthos from the Plymouth Sound from 1995. Plymouth Marine Laboratory, UK https://dx.doi.org/10.14284/297 |
| 9656db40-e4f9-4d8f-9023-38c7782efd9c | Cite data as:Rassweiler, A., Novak, M., Okamoto, D., Byrnes, J., Krumhansl, K. (2016). Global Kelp Timeseries from NCEAS/KEEN Working Group. Florida State University, Oregon State University, Simon Fraser University, University of Massachusetts Boston. Data accessed at http://catalogue-temperatereefbase.imas.utas.edu.au:/geonetwork/srv/en/metadata.show?uuid=ecbe5cc3-3fbf-4569-b5e8-07c2201fcb9c on [access date].    If you have kelp timeseries greater than 3 years in length, please consider contributing to the data set. Contact Jarrett Byrnes for details. |
| f9a6159e-d03b-45f2-a44a-eafb791e5987 | Kennedy, M.K., Spry, J.A. 2011. BioChem: Atlantic Zone Monitoring Program (AZMP) Maritimes Region zooplankton collection. OBIS Canada Digital Collections. OBIS Canada, Bedford Institute of Oceanography, Dartmouth, Nova Scotia, Canada, Version 1, Digital, retrieved from http://iobis.org/. |
| e7e9e58c-6c4c-4cc0-9df3-dd255fd303bd | dos Santos A, Niza H, Silva A, Almeida S (2023): Plankton Diversity Along the Portuguese Coast in the 1970s. v1.15. IPMA - Instituto PortuguÃªs do Mar e da Atmosfera. Dataset/Samplingevent. http://ipt.gbif.pt/ipt/resource?r=capec&amp;v=1.15 |
| e32ff326-333e-46c3-87c4-7641aad2fc9a | Chatzinikolaou E., Arvanitidis C. (2017). Benthic communities and environmental parameters in three Mediterranean ports (Sardinia, Crete, Tunisia). |
| 92af50ad-c9d7-4fe7-b012-eae0e77233a2 | NA |
| 80e7286d-6d8b-45ea-b279-eff2297752ae | Zooplankton from the bay of Biscay (1995-2004 MPDH surveys). Marine Research Unit, AZTI, Spain. https://doi.org/10.15468/1uh1hb |
| 3425145f-8016-403a-abca-9cb33a29108e | Saeedi H, Winterberg H, Alalykina IL, Bergmeier FS, Downey R, Golovan O, JaÅ¼dÅ¼ewska A, Kamenev GM, Maiorova A, Malyutina M, Minin K, Mordukhovich V, Petrunina A, Schwabe E, Brandt A (2018): NW Pacific deep-sea benthos distribution and abundance (Beneficial Project). v1.1. Deep-sea OBIS node. Dataset/Samplingevent. http://ipt.iobis.org/obis-deepsea/resource?r=beneficial_deepsea&amp;v=1.1 |
| 6e41986a-678c-498d-ac85-8513dfc952a9 | H.J. Hirche, K.N. Kosobokova, B. Gaye-Haake, I. Harms Originator: B. Meon and E.-M. NÃ¶thig (2006). Structure and function of contemporary food webs on Arctic shelves: A panarctic comparison. The pelagic system of the Kara Sea-communities and components of carbon flow |
| 3cd94a39-6a58-4f0e-9c74-e044e7a0e737 | Originator: Kosobokova K.N., Hirche H Publication_Date: 2009 Title: Zooplankton Eastern Arctic Ocean 1995, 1996, 1997, 1998 (Polarstern ARK-XI/1, ARK-XII, ARK-XIII, ARK XIV) Geospatial_Data_Presentation_Form: database Series_Information: Series_Name: Progress in Oceanography Issue_Identification: 82:265-280 |
| 210f1cc3-ac90-468e-9701-67cfae734ac6 | Puente A (2022): Littoral Monitoring Network of Cantabria (Invertebrates). |
| 798d34a8-1593-4543-886b-a72df94844b1 | Nehring, S., 1999. BfG-Monitoring in the German North Sea estuaries: Macrozoobenthos. Federal Institute of Hydrology. |
| 7166db26-05a1-4ca7-832b-19af8260f32e | Lakkis S., 2011. Le Zooplancton Marin du Liban (MÃ©diterranÃ©e orientale): Biologie, BiodiversitÃ©, BiogÃ©ographie. Publ.UniversitÃ© Libanaise, Beirut, Lebanon,No 23:566p. |
| f4db072d-b163-4e58-acf5-1d1a6b4ddbb2 | Tuaty-Guerra, M.; GaudÃªncio, M.J. (2021). Macrozoobenthos collected in marine waters off mainland Portugal in March and September 2010 https://dx.doi.org/10.14284/463 |
| 413657ae-72f4-4466-b9cb-f5e98230582a | Harris, L.R. (2013). The Les Harris Northwest Atlantic 0-100m zooplankton collection. Version 1 In OBIS Canada Digital Collections. Bedford Institute of Oceanography, Dartmouth, NS, Canada. Published by OBIS, Digital http://www.iobis.org/. Accessed on â€“INSERT DATE |
| b1ae4344-aa5a-49fb-80f6-e0e28e67833e | Kachalov, O.; Ukrainian Scientific Centre of Ecology of the Sea (UkrSCES); (2020): National Pilot Monitoring Studies Phyllophora July 2017, Improving Environmental Monitoring in the Black Sea - Phase II (EMBLAS-II), ENPI/2013/313-169, (Macrozoobenthos) |
| 00032856-12cd-46ff-b9d5-ddeaecae3c95 | Southwest Pacific OBIS (2013). Biological observation data from plankton surveys around New Zealand. Southwestern Pacific OBIS, National Institute of Water and Atmospheric Research (NIWA), Wellington, New Zealand, 36723 records, Online http://nzobisipt.niwa.co.nz/resource.do?r=mbis_plankton released on June 22, 2014. |
| 78acdd0c-0aa6-4dec-8b65-83794aa64476 | Tuaty-Guerra, M.; GaudÃªncio, M.J. (2021). Benthic macrofauna collected off Ericeira (central Portugal) in May 2001. https://dx.doi.org/10.14284/462 |
| 0c9db499-759b-46d8-8989-799f9ff9f235 | Blom W, Moriarty A (2018). Auckland Museum NZ Marine Collection. Version 1.11. Auckland War Memorial Museum. Occurrence Dataset https://doi.org/10.15468/plyefd accessed via GBIF.org on 2018-01-15. |
| a51cf5a2-1b8d-45ec-92ac-250836e80b63 | Benthos collected in the Azov Sea during several expeditions in 1934-1935. Institute of Biology of the Southern Seas of the National Academy of Sciences of Ukraine. |
| a914e242-0f35-4233-a9c5-429f7288631d | Federal Institute for Geosciences and Natural Resources of Germany, (2017). BGRPMN12017 Env Template BIONOD2012. Available : DeepData, International Seabed Authority https://data.isa.org.jm/ Accessed: [YYYY-MM-DD]. |
| c37c6228-542c-45a2-8bef-009ee03672a8 | NA |
| f70dc53f-b24c-4e6a-817f-7cd08bbe49ad | Przeslawski, R., Radke, L., and Hughes, M. 2009. Termporal and fine-scale  variation in the biogeochemistry of Jervis Bay. Geoscience Australia, Record 2009/12. Geoscience Australia, Canberra. 38pp. |
| b75c9982-d219-4156-8364-b9343f8b43db | YugNIRO-1 - Macrozoobenthos data and accompanied environmental data from the Black Sea Regions. |
| 67c16cec-808b-419d-9dd5-c1ca685af89c | Office FrancÌ§ais de la BiodiversiteÌ â€‹(OFB), Sorbonne UniversitÃ©/CNRS - Institut de la Mer de Villefranche (IMEV), Sorbonne UniversitÃ©/CNRS - Laboratoire d'OcÃ©anographie de Villefranche (LOV); 2020; Plankton community in WP2 net (200Âµm), PNMIR cruises, Parc Naturel Marin d'Iroise, France https://dx.doi.org/10.14284/481 |
| 549fa91f-0b54-4f42-afa5-ecb4597c33cc | Fonda-Umani, S.; Aleffi, F. (2014). Macrobentos North Adriatic-INTERREG-FVG Project. University of Trieste; Marine Biology Laboratory. https://doi.org/10.6092/0546f4f7-2de9-4996-bb83-8e5115e3e28d |
| ab06f0c6-3de3-447a-b339-a8517f2c95ed | NA |
| ca816ed5-be34-46d6-8e78-9bebcab8451a | Zooplankton abundance and population structures were assessed during April-September of 2001-2, Southern Ocean, GLOBEC |
| cbd631ea-c8ff-419f-8340-b29c7fdc416a | Romagnan, J.B., Jalabert, L., Olivier, M., Elineau, A., Brandao, M., Caray-Counil, L., Dimier, C., Picheral, M., Searson, S., Kandels-Lewis, S., Tara Oceans Consortium Coordinators, Karsenti, E., Pesant, S., Irisson, J.O., Lombard, F., Stemmann, L., Gorsky, G.; (2020) Occurrence of Eukaryotes in the size fraction &gt;200 micrometres, collected worldwide during the Tara Oceans expedition (2009-2013) using a WPII net and analysed on a ZooScan imaging platform. https://dx.doi.org/10.14284/521 |
| 34991b8f-e6a9-4870-812c-7ca24a729d13 | Janas U., 2002: Gulf of Gdansk dataset. University of Gdansk; Institute of Oceanography; Department of Marine Biology and Ecology, Poland https://dx.doi.org/10.14284/262 |
| b74b429a-4052-4f5b-bff3-fe0b5a2e8669 | WoRMS Editorial Board (2021). Type locality distributions from the World Register of Marine Species. Available from http://www.marinespecies.org at VLIZ. Accessed on YYYY-MM-DD. |
| dd1b9626-4dc8-47a1-b928-87e0cecea9f6 | China Ocean Mineral Resources Research and Development Association, (2015). COMRACRFC12015 Env Template Meiofaun. Available : DeepData, International Seabed Authority https://data.isa.org.jm/ Accessed: [YYYY-MM-DD]. |
| a8670051-b02c-4f6f-974d-caddc73b95ab | Barnard J L (2023). Marine Amphipoda of atolls in Micronesia, 1965. Version 1.0. Southwestern Pacific Ocean Biogeographic Information System (OBIS) Node. Occurrence dataset. https://nzobisipt.niwa.co.nz/resource?r=amphipoda_micronesia_1965&amp;v=1.0 |
| 4009980e-af6b-470a-b4be-16fb541b7c06 | Rees, H.L. et al. A comparison of benthic biodiversity in the North Sea, English Channel and Celtic Seas - Macroinfauna. Centre for Environment, Fisheries and Aquaculture Science; Burnham Laboratory, 12 Apr 2005, Essex, UK. |
| 04080bb2-fd9e-4b44-8cda-b6db6157ea9e | UK Seabed Resources Ltd. , (2016). UKSRLPMN12016 Senkenberg. Available : DeepData, International Seabed Authority https://data.isa.org.jm/ Accessed: [YYYY-MM-DD]. |
| d9118970-796c-485d-9d2c-b6853283907a | Fisheries Research Service, Marine Laboratory (2015). Macrobenthos samples collected in the Scottish waters in 2001. |
| 7dc633b1-f652-4cc4-8d89-5bfa7e208ed8 | Bossicart M.; Laboratorium voor Ekologie en Systematiek. VUB: Belgium; (2016): Zooplankton studies at a fixed station (West-Hinder) in the North Sea between 1977 and 1979. (http://www.vliz.be/en/imis?module=dataset&amp;dasid=4696) https://dx.doi.org/10.14284/266 |
| a2160400-5b14-45ce-b63a-b9b5d97e8de3 | Weslawski J.-M., E. Malec, R. JaskuÅ‚a, M. WÅ‚odarska-Kowalczuk, M. KÄ™dra, 2002: Polish Arctic Marine Programme. Macrobenthic data from Hornsund from 2002. Polish Academy of Sciences, Institute of Oceanology, Poland https://dx.doi.org/10.14284/183 |
| 1febfcdd-3e1b-46db-b73d-1bbe81ce22a6 | Miller R, NozÃ¨res C (2023). DFO Quebec Region MLI museum collection. Version 3.7. Fisheries and Oceans Canada. Occurrence dataset. http://iobis.org/mapper/?resource_id=2673 |
| b9efe6e8-dc63-47ef-a9c6-de82936746d1 | Klayn S, Karamfilov V (2021): Infauna from seagrass meadows in the coastal Bulgarian Black Sea (2013-2014). v1. Institute of Biodiversity and Ecosystem Research, Bulgarian Academy of Sciences (IBER-BAS). Dataset/Samplingevent. http://gp.sea.gov.ua:8082/ipt/resource?r=macrozoobenthos_seagrass_perseus_iber-bas&amp;v=1.0 |
| 547594d3-8bb3-46de-ba09-1e1f0ed6d629 | Espinosa-Leal L (2021): AnfÃ­podos_IMO. v1.1. Universidad de ConcepciÃ³n e Instituto Milenio de OceanografÃ­a (IMO). Dataset/Samplingevent. http://ipt.iobis.org/esp-obis/resource?r=anfipodos_cimar21&amp;v=1.1 |
| b42e9179-87a6-4686-a358-057d3e624c85 | UK Seabed Resources Ltd. , (2015). UKSRLPMN12015 Env Template Scavengers 032016. Available : DeepData, International Seabed Authority https://data.isa.org.jm/ Accessed: [YYYY-MM-DD]. |
| e971710e-054e-43f2-b23c-c149d6c76cb4 | Bertrand RICHER DE FORGES (IRD) &amp; Philippe BOUCHET (MNHN). 1998. Benthic species from the tropical Pacific. IRD-Noumea |
| 076221a7-0035-4f14-801f-22bb7d5b1be7 | OBIS Canada. 2011. FRB Eastern Arctic Investigations: The Calanus Series. OBIS Canada Digital Collections. OBIS Canada, Bedford Institute of Oceanography, Dartmouth, Nova Scotia, Canada, Version 2, Digital, retrieved from http://iobis.org/. |
| d684d4e8-5cc9-4669-a117-d8c7d5e1654a | Kevrekidis, T. &amp; Gouvis, N.; Bionomy of macrobenthic amphipods, molluscs and polychaetes in the Evros Delta, September 1981. Democritus University of Thrace; Department of Primary Education &amp; Aristotle University of Thessaloniki; Department of Biology |
| f3d7798e-7bf2-4b85-8ed4-18f2c1849d7d | Institut FranÃ§ais de Recherche pour l'Exploitation de la Mer â€“ IFREMER (2016): COMARGIS: Information System on Continental Margin Ecosystems https://doi.org/10.15468/0djslr |
| cbd9ed79-4fae-42ed-8ee3-00ca6b5028f2 | Australian Antarctic Data Centre (2017). RMT Trawl catch from the 1995/96 V4 BROKE voyage. |
| 843d3c1f-d82a-4ebd-b5f9-299706f04789 | Naumov, A. Benthos of the White Sea. A database. White Sea Biological Station, Zoological Institute RAS. |
| 0a39db7b-5797-4488-9970-5123ae7aad48 | Australian Antarctic Data Centre. Survey of benthic and other marine invertebrates of Prydz Bay, 1990/91 Voyage 6. |
| e53ff75c-cc73-4370-b074-36b34b8a7433 | Australian Antarctic Data Centre. RMT Trawl catch from the 1992/93 V6 KROCK voyage. |
| aec15b68-859f-41ed-aa27-11b4f7305a63 | UK Seabed Resources Ltd. , (2015). UKSRLPMN12015 Env Template Senckenberg 032016. Available : DeepData, International Seabed Authority https://data.isa.org.jm/ Accessed: [YYYY-MM-DD]. |
| e3009de8-2817-49b9-96fc-4e4cf2e76e24 | Antoniadou C. (1998). Macro- and megafauna from the North Aegean Sea from 1997-1998. Aristotle University of Thessaloniki, Department of Biology, Laboratory of Zoology, Greece. |
| 76f565d1-00e5-4093-a3e2-e0174091feaa | Yokohama Environmental Science Research Institute (2023) Marine benthic animal data along Yokohama City Coast. Available at https://doi.org/10.48518/00023. Accessed on YYYY-MM-DD. |
| 5adfc524-d5a8-4474-bc3a-bd97350fec3d | Hillewaert, H. (2000). Macrozoobenthos from the Belgian Continental Shelf, collected in 2000. Vlaamse Overheid; Beleidsdomein Landbouw en Visserij; Instituut voor Landbouw- en Visserijonderzoek; Kenniseenheid: Dier; Onderzoeksdomein Visserij, Belgium. |
| 159421b6-a764-4af1-9cc3-bc652dbeb12e | Southwestern Pacific OBIS (2014). British Antarctic (Terra Nova) Expedition, 1910-1913. Southwestern Pacific OBIS, National Institute of Water and Atmospheric Research (NIWA), Wellington, New Zealand, 1779 records, Online http://nzobisipt.niwa.co.nz/resource.do?r=terranova released on July 29, 2014 |
| 48f919e0-150b-4c2b-ae4a-745d39dc4f1d | See Metadata record for details http://data.aad.gov.au/aadc/metadata/metadata_redirect.cfm?md=AMD/AU/BROKE-West_RMT_Fish |
| a94aa77d-c064-48b6-9101-b2091f074c09 | Dos Santos, A.; Marraccini, A.; Afonso, C.; Correia, C.; Cunha, M. E.; Quintela, M. F. (2017). Mesopelagic Crustaceans of the North Western Portuguese Coast. https://dx.doi.org/10.14284/466 |
| af5cffee-635c-42b7-80df-9dbca0143e20 | Sorbonne UniversitÃ©/CNRS - Institut de la Mer de Villefranche (IMEV), Sorbonne UniversitÃ©/CNRS - Laboratoire d'OcÃ©anographie de Villefranche (LOV); 2020; Plankton community in WP2 net (200Âµm), Point B, Villefranche-sur-Mer, France https://dx.doi.org/10.14284/473 |
| 49b938b5-2d5a-43c0-92f9-26354f189f82 | OBIS (2018): UFPE Oceanography Zooplankton Research. v1. Tropical and Subtropical Western South Atlantic OBIS. Dataset/Occurrence. http://ipt.iobis.org/wsaobis/resource?r=ufpe_oceanography_zooplankton_research&amp;v=1.0 |
| aaa7c282-5909-4055-845c-026b0db8b86f | Matthias Steffens. (2006) Distribution and structure of macrobenthic fauna in the eastern Laptev Sea in relation to environmental factors. Polar Biology, 29(10):837-848 |
| f57b1db4-31be-4a1a-8828-9700664f7130 | Salabao L, Claes J, Gan Y, Van de Putte A, SchÃ¶n I (2022): Invertebrates from the ANTARXXVII Leg1 expedition to the Bransfield Strait, Antarctica - data. v1.6. SCAR - AntOBIS. Dataset/Occurrence. https://ipt.biodiversity.aq/resource?r=antarxxvii-leg1&amp;v=1.6 |
| 3bba3d8c-0f3a-4596-8b4c-245e4d9dc018 | Peer, D.L. (2016). FRB: Benthic communities in the Gulf of St. Lawrence as surveyed in February 1962. Version 1 In OBIS Canada Digital Collections. Bedford Institute of Oceanography, Dartmouth, NS, Canada. Published by OBIS, Digital http://www.iobis.org/. Accessed on â€“INSERT DATE |
| 5b9d8c82-3fdf-4aa3-8eaf-5399902a84ef | DFO. (2014). Atlantic Zone Monitoring Program (AZMP) DFO Quebec region phytoplankton. Version 1 In OBIS Canada Digital Collections. Bedford Institute of Oceanography, Dartmouth, NS, Canada. Published by OBIS. http://www.iobis.org/. (consulted on [date]) |
| bff60a89-23bb-4f31-9b40-7bbf2bb19b77 | This material is released under the Creative Commons Attribution 4.0 International licence. The citation in a list of references is: "IMOS [year-of-data-download], [Title], [data-access-URL], accessed [date-of-access]." Any users of IMOS data are required to clearly acknowledge the source of the material in the format described in the acknowledgments section. |
| 2dd370b3-69cb-47d5-9837-d3f84a65ea4a | Baumgartner, M.F. 2012. BioChem: Zooplankton collected from the Bay of Fundy and SW Scotian Shelf, 1999-2001. OBIS Canada Digital Collections. OBIS Canada, Bedford Institute of Oceanography, Dartmouth, Nova Scotia, Canada, Version 1, Digital, retrieved from http://iobis.org/. |
| 2da3c7ab-807e-4c57-a914-c196ed302036 | NA |
| fc18744b-4e55-4acc-8785-c29fb6692b34 | See Metadata record ttp://data.aad.gov.au/aadc/metadata/metadata_redirect.cfm?md=AMD/AU/ASAC_1189_benthic_database Contact Dave Watts for details on citation details. |
| bcd3a958-2fd8-4f8a-b5f7-88e690e36187 | Federal Institute for Geosciences and Natural Resources of Germany, (2015). BGRPMN12015 BIODIVERSITY. Available : DeepData, International Seabed Authority https://data.isa.org.jm/ Accessed: [YYYY-MM-DD]. |
| 4cf03bc4-0784-4990-bbc8-c25f9e214cb5 | Benthos collected in the Azov Sea in 1935 on board the R/V "N. Danilevskiy". Institute of Biology of the Southern Seas of the National Academy of Sciences of Ukraine. |
| 7666cde7-83ef-4161-b0f1-798770d33ca0 | Harding, Gareth C.H.(2016). Zooplankton distribution in the Arctic Ocean based on samples collected at Ice Island T-3 in 1964. Version 2 In OBIS Canada Digital Collections. Bedford Institute of Oceanography, Dartmouth, NS, Canada. Published by OBIS, Digital http://www.iobis.org/. Accessed on â€“INSERT DATE |
| 7a950cb1-88cc-4401-9368-1495e64586ff | Fabio Lang da Silveira and Rubens M. Lopes Rapid Marine Biodiversity Assessment of the Abrolhos Bank, Bahia, Brazil. WSAOBIS, SÃ£o Paulo, November 2008, Version 1, Publication. |
| 057859e2-3ff2-4f55-950f-62089486a7f7 | Van Dalfsen, J. (2000). Macrobenthos data from the Doggerbank - 2000. Institute for Marine Resources and Ecosystem Studies; Vestiging Den Helder, The Netherlands. |
| 270f3e70-ff9b-411d-b170-2bc914d83f26 | Olivas GonzÃ¡lez F J (2016): Biological Reference Collections ICM CSIC. Institute of Marine Sciences (ICM-CSIC). https://dx.doi.org/10.15470/qlqqdx |
| a449c6d1-7a79-4b84-a31d-028a3a70b0cb | LÃ¶rz, A.-N.; KÃ¼rzel, K.; Andres, H.G.; Tandberg, A.H.; Hughes, L.; Peart, W.; Krapp-Schickel, T.; Campean, A.J.; Stransky, B.; Guerra-GarcÃ­a, J.M.; Svavarsson, J.; Brix, S.; (2019); North Atlantic and Arctic Amphipoda sampled during IceAGE project. Marine Data Archive |
| 9651c30a-ea93-4e20-8cce-e9e951d9fe68 | National Museums Northern Ireland (NMNI) (2021): Marine Recorder Snapshot extract of surveys entered by National Museums Northern Ireland (NMNI). v2.0. Marine Biological Association. Dataset/Samplingevent. https://doi.org/10.17031/frdvov |
| 0ea4bbdc-050a-4329-a21a-002d45b91656 | Stelzer PS, Mazzuco ACA, Gomes LEO, Neto JM, Netto SA, Bernardino AF (2021). The Long-term Ecological Research Program Coastal Habitats of EspÃ­rito Santo - Macrofauna in Rhodolith Beds. v1.4. Tropical and Subtropical Western South Atlantic OBIS. Dataset/Samplingevent. http://ipt.iobis.org/wsaobis/resource?r=lter_hces_macrofauna_rhodolithbeds&amp;v=1.5 |
| 33402693-a77d-443e-87a7-f9597315f76c | NA |
| 3143fd54-9026-4d3a-a01b-ea22f91f6623 | Abarca-Avila M M, Palomo-Aguayo U, DÃ¡vila-JimÃ©nez Y, HernÃ¡ndez-AlcÃ¡ntara P, SuÃ¡rez-Mozo N Y, Rivas G, Soler-Membrives A, Silva-Morales I, HernÃ¡ndez-DÃ­az Y Q, SolÃ­s-MarÃ­n F A, Ugalde D, Morales-PatiÃ±o J R, Simoes N, Chiappa-Carrara X, Papiol Nieves V (2021): MACROBENTHOS COMPOSITION FROM THE RIO LAGARTOS HYPERHALINE COASTAL LAGOON SYSTEM, YUCATAN, MEXICO. v1.3. Caribbean OBIS Node. Dataset/Samplingevent. http://ipt.iobis.org/caribbeanobis/resource?r=macrobenthosriolagarto&amp;v=1.3 |
| f1a11336-64b8-46f4-ba10-5095d150039f | Brodie, Bill, Mowbray, Fran and Power, Debbie, 2013. DFO Newfoundland and Labrador Region Ecosystem Trawl Surveys. Version 1 In OBIS Canada Digital Collections. Bedford Institute of Oceanography, Dartmouth, NS, Canada. Published by OBIS, Digital http://www.iobis.org/. Accessed on â€“INSERT DATE |
| 9c5b3b91-d932-445f-899a-e1446846cdef | Indian Ocean Biodiversity Information System (IndOBIS)- Distribution records of marine organisms from the Indian Ocean. |
| 4fd95328-42c6-44cb-9347-64282f4946d5 | NORFANZ - biodiversity survey 2003, Norfolk Ridge and Lord Howe Rise |
| 30e505e2-27fc-434e-b8c1-19f8d3c02eb4 | China Ocean Mineral Resources Research and Development Association, (2018). COMRAPMN12018 Lander2017. Available : DeepData, International Seabed Authority https://data.isa.org.jm/ Accessed: [YYYY-MM-DD]. |
| 23c8118f-5343-4fca-a09a-de477348e0b2 | Michel, L.; Remy, F.; Mascart, T.; De Troch, M.; Lepoint, G.; (2021): Stable isotope ratios of C and N in benthic macrofauna from Mediterranean seagrass litter accumulations from Calvi Bay in 2011-2012. Marine Data Archive. https://dx.doi.org/10.14284/454 |
| 5803e1ae-b493-4174-83ed-260363d08047 | Sorbonne UniversitÃ©/CNRS - Institut de la Mer de Villefranche (IMEV), Sorbonne UniversitÃ©/CNRS - Laboratoire d'OcÃ©anographie de Villefranche (LOV); 2020; Plankton community in WP2 net (200Âµm), DYFAMED point, Ligurian Sea, France https://dx.doi.org/10.14284/479 |
| 163b3b00-c6bb-49dd-a990-c3c4f5b1bcc9 | Chatzigeorgiou G, Androulakis D, Skouradakis G, Rallis I, Gratsia E (2022): Polychaetes and Amphipodes from major ports in Greece in 2020-2021. v1.11. Hellenic Center for Marine Research. Dataset/Samplingevent. http://ipt.medobis.eu/resource?r=alienports_polychaetes&amp;v=1.11 |
| 97bdf279-6f78-4ed7-ad9d-27a5886f8ff2 | TÃ¼rkay, M. Senckenbergisches Sammlungsverwaltungssystem, SeSam. Senckenbergische Naturforschende Gesellschaft , Frankfurt, Germany. http://sesam.senckenberg.de/ |
| 6e18e05f-a198-49e2-a59d-edd721f5a677 | DÃ­az HernÃ¡ndez P, GutiÃ©rrez-Salcedo J M, Montoya-Cadavid E (2021): ColecciÃ³n de Plancton Mixto â€“ SecciÃ³n Zooplancton del Museo de Historia Natural Marina de Colombia â€“ Makuriwa (Invemar). v1.1. Instituto de Investigaciones Marinas y Costeras - Invemar. Dataset/Occurrence. https://doi.org/10.15472/arqxyv |
| c52d1311-dd2e-4761-a6f6-1ba9a28e0c66 | Kachalov, O., Tretiak, I., Ukrainian Scientific Centre of Ecology of the Sea (UkrSCES); (2020): National Pilot Monitoring Studies Phyllophora April 2017, Improving Environmental Monitoring in the Black Sea - Phase II (EMBLAS-II), ENPI/2013/313-169, (Macrozoobenthos) |
| 7b32d3ea-a8ad-455c-b1e8-b22a34ca17fd | NA |
| 9a2ca2e2-e8fb-4de6-9c7c-a532578494ca | CEFAS. - UK (2015). Macrobenthos from English waters between 2000-2002. |
| cdcddae2-a8e0-4c86-9e8d-f85134876937 | NA |
| cd691a3e-8f01-4166-8d2e-a084e22c9bec | DÃ¡vila Reyes D, GÃ³mez Lemus L A, MejÃ­a Torres L A, BolaÃ±o Lara M, Orjuela Rojas A, Ricardo R E, Caicedo Torrado I, Aguilar PÃ©rez M, Barrios VÃ¡squez E, Giraldo Escobar C, MÃ¡rquez Hoyos J, Rojas S, Vilarete E, Charrys J (2022): Monitoreos de los ecosistemas marinos de la bahÃ­a Portete, Puerto BolÃ­var, La Guajira. v1.1. Carbones del CerrejÃ³n Limited. Dataset/Samplingevent. https://doi.org/10.15472/jhiwsd |
| 2dc172af-a053-4e5a-9a38-b5425e0116c1 | Aleffi, I. F., 2004: LBMRev dataset. Marine Biology Laboratory, University of Trieste, Italy |
| 8629ec33-be4b-4384-933f-a511fbc29967 | Wenneck, T. de Lange, Falkenhaug, T. and O.A. Bergstad. 2008. Strategies, methods, and technologies adopted on the RV G.O. Sars MAR-ECO expedition to the mid-Atlantic Ridge in 2004. Deep-sea Research II. 55: 6-28. |
| 471a8de8-80f8-43f9-9443-88a45712feba | Ramirez-Llodra, E., Blanco, 2005. ChEssBase: an online information system on biodiversity and biogeography of deep-sea fauna from chemosynthetic ecosystems. Version 2. World Wide Web electronic publications, http://www.noc.soton.ac.uk/chess/database/db_home.php |
| 3dbbeff2-2801-411f-80e5-438222f1aaa4 | Originator: Howard M. Feder, Stephen C. Jewett, Arny L. Blanchard Publication_Date: 20060621Title: Southeastern Chukchi Sea (Alaska) macrobenthos Geospatial_Data_Presentation_Form: database Series_Information: Series_Name: Polar Biology Issue_Identification: 30:261-275 |
| ffda777b-ea11-4cdf-b0cf-7919a0a97670 | <p>  Robinson, K.A., Darbyshire, T., Van Landeghem, K., Lindenbaum, C., McBreen, F., Creaven, S., Ramsay, K., Mackie, A.S.Y., Mitchell, N.C., Wheeler, A., Wilson, J.G. &amp; O'Beirn, F. 2009. Habitat mapping for conservation and management of the southern Irish Sea (HABMAP). I: Seabed surveys. Studies in Marine Biodiversity and Systematics from the National Museum of Wales. BIÃ”MOR Reports 5(1): 234 pp.</p> |
| ac6789a8-aef9-4642-b67a-d4ffb25e8245 | NA |
| 2f257fe1-dc7d-45a6-8218-5995e1574b32 | Australian Antarctic Data Centre. RMT Trawl catch from the 1986/87 V7 AAMBER voyage. |
| fa8ee269-74a6-4e52-b576-fcf023e90f9e | Macrozooplankton concentrations estimated from MOCNESS tow samples, Continental Margin Western Antarctic Peninsula, GLOBEC |
| 62658cbe-5420-4c6f-91cd-0acb6d8bc03a | CSIRO - Southern Surveyor voyage SS 01/97, Tasmanian Seamounts study |
| ad96e7c9-c136-4fa6-ac1c-5c15b328c1b5 | J.W. Wacasey, E.G. Atkinson, L. Derick and I. Weinstein (1977). Zoobenthos data from the Southern Beaufort Sea, 1971-1975. Fisheries and Environment Canada. Fisheries and Marine Service Data report 41 |
| 140dcbab-d94c-48d5-94aa-221190cfbbb9 | Citation_Information: Originator: MANDATORY for FGDC: The originator of the data Publication_Date: Unknown Title: Barents Sea Zooplankton â€“ March and May 1998 and July 1999. Day/night stratified zooplankton sampling onboard the Norwegian R/V Jan Mayen. Program ALV. Online_Linkage: NA |
| d7976b9a-9066-4bd6-8b12-77ffae85d774 | Herrmann, M., J. Laudien, 2004: Soft bottom community structure and diversity in Arctic Kongsfjorden. Alfred-Wegener-Institute for Polar and Marine Research, Bremerhaven, Germany https://dx.doi.org/10.14284/263 |
| 849d1641-b8d5-4aeb-b534-94c2e1a844c6 | Sanvicente-AÃ±orve L, Hermoso-Salazar M, VelÃ¡zquez-RamÃ­rez B (2022): Pelagic amphipods (Crustacea: Amphipoda: Hyperiidea) from the southern Gulf of Mexico. v1.4. Caribbean OBIS Node. Dataset/Occurrence. https://ipt.iobis.org/caribbeanobis/resource?r=icml-lesp_pelagic_amphipods_s_gom&amp;v=1.4 |
| 52589ec3-90ff-45a4-b718-57739f03bf83 | Camelia Dumitrache, Adrian Filimon, Valeria Abaza - Zoobenthos data collected in the northern part of the Romanian littoral (Danube mouths) between 2011-2015. NIMRD - National Institute for Marine Research and Development " Grigore Antipa" |
| 55fabbc5-6449-4701-bb5d-4a37270cd3b8 | NA |
| e2f42da0-3dc1-49b0-8fd2-6bcc08d47925 | Dumitrache C.; Abaza V.; Filimon A. (2017). Macrozoobenthos data collected from the longitudinal profiles in the Romanian marine waters between 1986 and 1990. NIMRD-National Institute for Marine Research and Development "Grigore Antipa" |
| 3eb5bcd4-5258-48a4-93dd-ae14de6f7e56 | Zettler M. L., 2005: Macrozoobenthos baltic sea (1980-2005) as part of the IOW-Monitoring. Institut fÃ¼r Ostseeforschung WarnemÃ¼nde, Germany |
| 6a5bc28f-4dfe-4cbf-8a55-7e3a843997ab | Allain, V., Menkes, C., 2014. Nectalis 3 cruise, RV Alis. https://doi.org/10.17600/14004900 |
| fd7ade64-1931-4542-a92f-2b9d56ee295f | Federal Institute for Geosciences and Natural Resources of Germany, (2017). BGRPMN12017 Env Template MANGAN2013. Available : DeepData, International Seabed Authority https://data.isa.org.jm/ Accessed: [YYYY-MM-DD]. |
| 392bc81f-4a51-429e-93d2-be78799fdd82 | Feder HM, Jewett SC, Blanchard A. (2005) Southeastern Chukchi Sea (Alaska) epibenthos 1976. Polar Biology, 28: 402-421 |
| 704d7278-2647-436d-9bd0-8913a1d189cb | NA |
| b0a7add2-dd9e-4020-9ca4-5df048c8f6a2 | Fabri, M-C. et al., Ifremer BIOCEAN database (Deep Sea Benthic Fauna). Institut FranÃ§ais de Recherche pour l'Exploitation de la Mer, Ifremer, Issy-les-Moulineaux, France. World Wide Web electronic publication, http://www.ifremer.fr/isi/biocean |
| cf8dde43-858e-4e8a-927f-53c90ab5cd5e | Slabinsky, A., Somov, A., &amp; Pakhomov, E. (2022). Juday Net Zooplankton Data from the 2019 Gulf of Alaska International Year of the Salmon Expedition (Version 1.0) [Data set]. North Pacific Anadromous Fish Commission. https://doi.org/10.21966/ZW3V-MD11 |
| 307bff4d-7eb1-45cb-8573-542efd0ab6ba | Oleg Kachalov Ukrainian Scientific Centre of Ecology of the Sea (UkrSCES) |
| 6142952e-7bbc-434f-a055-6bf140e6463d | Montoya Cadavid E (2021): ColecciÃ³n de Artropodos del Museo de Historia Natural Marina de Colombia - Makuriwa. v2.2. Instituto de Investigaciones Marinas y Costeras - Invemar. Dataset/Occurrence. https://doi.org/10.15472/eateut |
| beab1dcb-8235-4072-85cd-d8c3373e4ae0 | NA |
| d53e35de-220e-4ce1-b670-41488d89a045 | Dewarumez, J.-M., Dauvin, J.-C. &amp; Desroy, N - 2000. Macrobenthos from Copale - Authie. Station Biologie de Wimereux, France. |
| 239b0a6b-0ac6-4ede-a8eb-89269c0f8655 | Dumitrache C.; Filimon A.; (2021). Macrozoobenthos data collected in the northern part of the Romanian littoral (Danube mouths) between 2000-2010. NIMRD - National Institute for Marine Research and Development " Grigore Antipa" |
| bc01451e-d990-4ad1-8315-e3fb6e9cf461 | NA |
| 3ac81162-1804-43c4-b766-aabe2abcc572 | The Danish Biodiversity Information Facility, Galathea II, Danish Deep Sea Expedition 1950-52 https://doi.org/10.15468/ouseij |
| 2b047106-47f5-45db-962f-f321924eb25b | Dumitrache C.; Abaza V.; Filimon A. (2017). Macrozoobenthos data collected in the Northern part of the Romanian littoral (Danube mouths) between 1977 and 1999. NIMRD-National Institute for Marine Research and Development "Grigore Antipa" |
| cc88dadf-56a4-4d09-9b02-77f967204701 | NA |
| 9625bb4a-bbbb-4cde-946f-e74f973fdba4 | Japanese Association for Marine Biology (2022) Regionally Integrated Marine Database (RINKAI), managed by JAMBIO, an association of Japanese university-based marine stations. Available at https://doi.org/10.48518/00010. Accessed on YYYY-MM-DD. |
| 48f832cb-d71f-4ba9-b711-3b8c6559f5db | Soetaert K. 1999: Size of Atlantic nematodes. Netherlands Institute of Ecology; Centre for Estuarine and Marine Ecology, Netherlands. Metadata available at http://mda.nioo.knaw.nl/imis.php?module=dataset&amp;dasid=668 |
| c8f9d44b-9c51-4a7b-b9b2-598393dc904c | Australian Antarctic Data Centre. RMT Trawl catch from the 1982/83 V2 ADBEX I voyage. |
| eddee438-1ec9-4e50-83ca-a7ee7bc324cc | Silveira FL, Lopes RM (2008): Marine Biodiversity in Ilha Grande Bay Rio de Janeiro State - Southwest Brazil. v1. Tropical and Subtropical Western South Atlantic OBIS. Dataset/Occurrence. http://ipt.iobis.org/wsaobis/resource?r=marine_biodiversity_in_ilha_grande_bay&amp;v=1.0 |
| 9db24ef5-0c19-4a5b-82a7-be3a31dec4b8 | The Plankton Society of Japan, The Japanese Association of Benthology (2018) Plankton&amp;BenthosResearch at http://www.godac.jamstec.go.jp/bismal/e/PlanktonBenthosResearch. Accessed on yyyy-mm-dd. |
| 13edfad1-54eb-486e-9711-f9e9eb01eb1a | Indian Ocean Biodiversity Information System (IndOBIS)- Distribution records of marine organisms from the Indian Ocean. |
| faa8ba52-0bdd-4fbf-ba2c-054aadaff072 | Laborda, A.J. (2007) CRAI-Experimental, Colecciones ZoolÃ³gicas ULE, ColecciÃ³n de MalacostrÃ¡ceos. https://doi.org/10.15468/yyaxl7 |
| 44fdd837-036d-4ea7-a70e-cd9bdc6133f6 | Whomersley, P., 2003: National Marine Monitoring Programme. Benthos data of the North Sea, Irish Sea, English Channel from 2002-2003. CEFAS, Burnham On Crouch, UK |
| 4312e526-3f49-4291-9ef6-91408686cb7a | Magee, S., Kenchington, E., Davis, D.S., Butler, M., (2016) Systematic list of the epibiotic taxa associated with scallop shells from the Digby area of the Bay of Fundy. Version 1 In OBIS Canada Digital Collections. Bedford Institute of Oceanography, Dartmouth, NS, Canada. Published by OBIS, Digital http://www.iobis.org/. Accessed on â€“INSERT DATE |
| f9979274-f5c3-4cee-928e-7e77b332546d | Hunt H (2019): UNBSJ: Long-term monitoring of benthic infaunal invertebrates at sites in Saint John Harbour, New Brunswick. v1.1. Canadian node of the Ocean Biogeographic Information System (OBIS Canada). Dataset/Occurrence. http://ipt.iobis.org/obiscanada/resource?r=unbsj_sjh_heatherhunt&amp;v=1.1 |
| 9d629feb-ba03-4a83-908c-794d94d30531 | Accession ID OGL-#####. The Ocean Genome Legacy Center. Northeastern University. Published on the web at ogl.northeastern.edu/catalog. |
| 98c3a0e6-ef76-4894-aabb-d7f01a0e7cea | Indian Ocean Biodiversity Information System (IndOBIS)- Distribution records of marine organisms from the Indian Ocean. |
| 80d96759-eeb8-4d68-9413-82425fa582d0 | Juillet C, LÃ©vesque I, NozÃ¨res C, Roy V (2022): DFO Quebec Region Biodiversity of the snow crab trawl survey in the St. Lawrence Estuary (2019). v1.2. Fisheries and Oceans Canada. Dataset/Occurrence. https://ipt.iobis.org/obiscanada/resource?r=dfo_que_est_co&amp;v=1.2 |
| afef5da2-614b-4208-aee6-c2413ed5ab76 | Muller-Karger F, Kelble C, Montes E, Djurhuus A, Rojas-Marquez J, Lopez-Figueroa N, Di Geronimo S, Rueda-Roa D, Murray T (2023). Time series of zooplankton abundance in the Florida Keys, collected by the South Florida Program (NOAA/AOML) and the Marine Biodiversity Observation Network (MBON). Version 1.8. United States Geological Survey. Samplingevent dataset. https://ipt-obis.gbif.us/resource?r=sfmbon_zooplankton&amp;v=1.8 |
| db9358a6-7487-4c74-8fd8-9d47c2a3a30e | Govaere J.; Laboratorium voor Morfologie en Systematiek der Dieren. RUG: Belgium; (2016): Analysis of the macrobenthic community near Nieuwpoort (1970-1971). (http://www.vliz.be/en/imis?module=dataset&amp;dasid=5432) https://dx.doi.org/10.14284/206 |
| 5b6251f6-a7a5-4dc9-994d-c9504b54776f | NA |
| 14d5f344-9e79-496f-b914-c98ea5b9617e | Stewart P L, Levy H A, Walker T R (2016): Benthic invertebrate surveys conducted between 2009-2011 as part of the Sydney Tar Ponds Cleanup and Coke Ovens Remediation Project.. v1.2. Canadian node of the Ocean Biogeographic Information System (OBIS Canada). Dataset/Samplingevent. http://ipt.iobis.org/obiscanada/resource?r=dillon_sydneytarponds_01&amp;v=1.2 |
| 443fde8d-61ca-47d6-8246-1c150d5e6166 | Klayn S, Karamfilov V (2021): Soft-bottom macrozoobenthos from the coastal Bulgarian Black Sea (2013-2014). v1. Institute of Biodiversity and Ecosystem Research, Bulgarian Academy of Sciences (IBER-BAS). Dataset/Samplingevent. http://gp.sea.gov.ua:8082/ipt/resource?r=macrozoobenthos_sand_perseus_iber-bas&amp;v=1.0 |
| 24b24b44-52ef-4f6a-b9a0-5fa20bfeff49 | Falkenhaug, T. (2014). IMR Zooplankton North Sea. Institute of Marine Research, Norway |
| 172bfa9a-ada7-48cf-aecd-8508c6128b7d | Breckenridge, J., Galbraith, M., King, J., Pinchuk, A., Stark, C., &amp; Pakhomov, E. (2023). Bongo Zooplankton Data from the R/V TINRO, NOAA Bell M. Shimada, F/V Northwest Explorer and R/V CCGS Sir John Franklin during the 2022 International Year of the Salmon Pan-Pacific Winter High Seas Expedition (v1.0). North Pacific Anadromous Fish Commission. https://doi.org/10.21966/ymv6-8024 |
| 5ea4f836-9e28-4542-ac4a-af10630fbb7a | Coral Reef Ecosystem Division (CRED), Pacific Island Fisheries Sciences Center, NOAA National Marine Fisheries Service. 2011. CRED REA Algal Quadrate Images in the Pacific Ocean 2002-2008. Coral Reef Ecosystem Division, Honolulu, HI.Retrieved from http://www.usgs.gov/obis-usa/. |
| 75e2a2b6-f58f-4c43-aefd-878cee7865b1 | Australian Antarctic Data Centre, RMT Trawl catch from 1985/86 V1 ADBEX III voyage (accessed through GBIF data portal) |
| 3e8a8d84-09c1-475f-a172-c4d69af5d2ca | Galbraith, M. (2013) DFO Pacific IOS zooplankton database - Zooplankton collection from Arctic cruises, 1987-2000. Version 1 In OBIS Canada Digital Collections. Bedford Institute of Oceanography, Dartmouth, NS, Canada. Published by OBIS, Digital http://www.iobis.org/. Accessed on â€“INSERT DATE |
| ba60e7b1-ca45-40e1-8de3-c9a70e25f8a3 | Dumitrache C.; Abaza V.; Filimon, A. (2017). Macrozoobenthos collected in the East Constanta sector of the Romanian marine waters between 1977 and 1999. NIMRD-National Institute for Marine Research and Development "Grigore Antipa" |
| 3cd86c95-eaff-4561-8498-eced07e2a4db | NA |
| 8f5213fe-787d-4ff9-b981-867c763c1dc8 | Tuaty-Guerra, M.; GaudÃªncio, M.J. (2021). Benthic fauna collected in the Prof. Luiz Saldanha Marine Park (ArrÃ¡bida, SW Portugal) between 2007 and 2009. https://dx.doi.org/10.14284/461 |
| e548f7dc-7f39-4597-8258-351bfe98aaf0 | Spry, J.M. 2012. BioChem: Zooplankton collected from Davis Strait, 1976-1977. OBIS Canada Digital Collections. OBIS Canada, Bedford Institute of Oceanography, Dartmouth, Nova Scotia, Canada, Version 1, Digital, retrieved from http://iobis.org/. |
| 27f7853d-48c4-4365-8b0b-158d8fffb7c7 | Althaus F (2023): Great Australian Bight Research Program, Australia (2015-2017). v1.11. CSIRO National Collections and Marine Infrastructure (NCMI) Information and Data Centre (IDC). Dataset/Samplingevent. https://www.marine.csiro.au/ipt/resource?r=csiro_gabrp&amp;v=1.11 |
| 6c32ba39-8852-496d-8520-b82384d3f931 | Reizopoulou S., Sigala K., Tsompanou M., Vasileiadou K., Pavloudi C., Arvanitidis C. (2016) Benthic communities in Amvrakikos Wetlands: Mazoma, Tsopeli,Tsoukalio, Rodia and Logarou lagoons (September 2010 â€“ July 2011. |
| d1c53013-998f-4ee1-8664-7b0d25a2fb8b | Nelson, W.A.; Neill, K.; Barr, N.; D'Archino, R.; Miller, S.; Stewart, R. (2012). Data from: Rhodolith Beds in Northern New Zealand: Characterisation of Associated Biodiversity. Southwestern Pacific OBIS, National Institute of Water and Atmospheric Research (NIWA), Wellington, New Zealand, 684 records, Online http://nzobisipt.niwa.co.nz/resource.do?r=rhodolithhabitats released on June 20, 2015. |
| 91fa6e0a-cfb0-43d9-8dcc-588ff31e3e4d | Teaca, A.; Begun, T.; Muresan, M.; National Research and Development Institute for Marine Geology and Geoecology â€“ GeoEcoMar, Romania (2016): Historical benthos data from the Pre-deltaic Romanian Black Sea Coast in 1960 |
| 1662ded3-666e-4e85-b4a7-fe7f103521bd | Klayn S, Karamfilov V (2021): Soft-bottom macrozoobenthos from the littoral zone of the Natura 2000 MPA Ropotamo, Southern Bulgarian Black Sea (2013). v1. Institute of Biodiversity and Ecosystem Research, Bulgarian Academy of Sciences (IBER-BAS). Dataset/Samplingevent. http://gp.sea.gov.ua:8082/ipt/resource?r=macrozoobenthos_sand_coconet_iber-bas&amp;v=1.0 |
| e09c824c-cbd8-4529-b382-5306b2b3a875 | Deudero, Vallespir, Obrador 2011. Atlas de Biodiversidad Marina del Mar Balear. http://www.ba.ieo.es |
| 554826ea-46d5-433d-b2bb-f4000b37123c | Cattrijsse, A.; Verween, A. ; Marine Biology Research Group - Ugent, Belgium (2004). Characterisation of the habitat of the Flemish banks, the maritime country banks and of the hinder banks on the basis of the hyper benthos communities. http://www.vliz.be/nl/imis?module=dataset&amp;dasid=153 |
| a85edab7-cd3f-43ae-b491-37ab2e7edf5a | Garside, Peter, Suthers, Iain, Biological, Earth &amp; Environmental Sciences, Faculty of Science, UNSW, and Mazumder, Debashish, Australian Nuclear Science Technology Organisation. The Size Based Ecosystems of Warm and Cold Core Eddies off Eastern Australia (2021). |
| 79511c09-9498-43ad-a7d5-a733b9da3be8 | RodrÃ­guez A, Berben A, Quiroga S (2023): ArtrÃ³podos no Insectos de las Colecciones BiolÃ³gicas de la Universidad del Magdalena. v1.0. Universidad del Magdalena. Dataset/Occurrence. https://doi.org/10.15472/ikcgta |
| bbf8d1a8-705e-43f7-bdcb-cced8bf4a40c | Ward, P., Tarling, G., Shreeve, R., &amp; ten Hoopen, P. (2020). Epipelagic mesozooplankton distribution and abundance in Southern Ocean Atlantic sector and the North Atlantic and Arctic 1996-2013 [Data set]. UK Polar Data Centre, Natural Environment Research Council, UK Research &amp; Innovation. https://doi.org/10.5285/5A711904-EF42-46A3-9F47-3F0D6B231F65 |
| 18d7384e-31ef-48a3-aa3a-bae1e32a38aa | Bermudez-Rivas C, Cuellar ChacÃ³n A, Herrera Carmona J C, Reyes J, Quintana H, Giraldo A, Moreno Y, CastrillÃ³n F, Gallego Zerrato J J, Ortiz Mieles L (2023): Diversidad, abundancia y distribuciÃ³n del ictioplancton y su relaciÃ³n con las condiciones oceanogrÃ¡ficas y los perÃ­odos mareales en la subregiÃ³n Sanquianga-Gorgona, frente al delta del rÃ­o PatÃ­a. v1.0. DirecciÃ³n General MarÃ­tima. Dataset/Samplingevent. https://doi.org/10.15472/xhfmaj |
| f7677584-eb01-44ca-96f9-a334381034fa | Alabama Museum of Natural History Invertebrate Zoology Collection https://arctos.database.museum/collection/ALMNH:Inv |
| 0724e96a-c4b4-4ab9-adde-ab6bf621b616 | NA |
| eb7eea58-e79f-4bbd-8296-c366270ac61f | Przeslawski, Rachel,McArthur, Matthew A.,Anderson, Tara J. (2013) Infaunal biodiversity patterns from Carnarvon Shelf (Ningaloo Reef), Western Australia. Marine and Freshwater Research 64 pp573- doi:10.1071/MF12240 |
| 6d44cfeb-de07-4efc-b71b-3d541cb4a413 | Zenetos A. &amp; C. Bogdanos, 1992: Kerkyra dataset. Hellenic Centre for Marine Research, Greece |
| 37406772-0bd9-4647-a699-cabc74a23741 | Goldsmit, Jesica. 2016. CAISN: Abundance and biomass of benthic invertebrates collected in four ports of the Canadian Arctic during summers of 2011 and 2012. Version 2 In OBIS Canada Digital Collections. Bedford Institute of Oceanography, Dartmouth, NS, Canada. Published by OBIS, Digital http://www.iobis.org/. Accessed on â€“INSERT DATE |
| 1a653b53-5fe5-4d67-b62c-a69294553304 | Romagnan, J.B., Jalabert, L., Brandao, M., Caray-Counil, L., Dimier, C., Picheral, M., Searson, S., Kandels-Lewis, S., Tara Oceans Consortium Coordinators, Karsenti, E., Pesant, s., Irisson, J.O., Lombard, F., Stemmann, L., Gorsky, G.; (2020) Occurrence of Eukaryotes in the size fraction &gt;680 micrometres, collected worldwide during the Tara Oceans expedition (2009-2013) using a RÃ©gent net and analysed on a ZooScan imaging platform. https://dx.doi.org/10.14284/522 |
| 9c1aeafd-0a62-4799-95f5-88068b718c36 | Hunt, B., Mahara, N., &amp; Pakhomov, E. (2021). Zooplankton Bongo Net Data from the 2019 and 2020 Gulf of Alaska International Year of the Salmon Expeditions [Data set]. https://doi.org/10.21966/7cmt-ca72 |
| bef066c4-4afc-4553-9e99-88285868aba4 | Rumohr, H. Holsatia-expedition 1887 - animals collected with a dredge during the expedition. Christian-Albrechts-University Kiel; Leibniz Institute of Marine Sciences; Marine Ecology Division; Benthos Ecology section, Kiel, Germany. |
| a003e72f-d8e5-4d94-9aed-4bc50862c4ad | NA |
| 5ded37eb-bf01-46ae-9470-d5e2863fd622 | Marine Institute (2003); Groundfish Survey Invertebrate Data, |
| 3d79aa7d-d918-4924-a8c4-bfcb799b63aa | NA |
| 5cfef543-d293-45b8-b656-c3628ff34b7c | NMFS-COPEPOD: the global plankton database. ONLINE. 2009. Available: http://www.st.nmfs.noaa.gov/plankton/index.html. |
| 531637ff-203d-447a-8595-519080144a1d | Finnish Environment Institute SYKE; (2018); Finnish Baltic Sea zooplankton monitoring |
| e0ce9f82-687e-4cbf-95d0-d27d1054bfe9 | Knauber H, Kohlenbach K, Yasuhara P, LÃ¼ter C, Werner R, Brandt A, Saeedi H (2023): Deep-sea benthic crustacean, polychaete, and sipunculid data from the Bering Sea. v1.0. Deep-sea OBIS node. Dataset/Samplingevent. https://ipt.iobis.org/obis-deepsea/resource?r=deep-sea_benthic_bering_sea_0&amp;v=1.0 |
| e54ba46d-583f-416a-ba4f-bb388b1de043 | Indian Ocean Biodiversity Information System (IndOBIS)- Distribution records of marine organisms from the Indian Ocean. |
| 62f819bf-7960-473d-9f95-ee9a5bc2e25f | Juillet C, LÃ©vesque I, NozÃ¨res C, Roy V (2022): DFO Quebec Region Biodiversity of the snow crab trawl survey in the St. Lawrence Estuary (2019). v1.2. Fisheries and Oceans Canada. Dataset/Occurrence. https://ipt.iobis.org/obiscanada/resource?r=dfo_que_bcn_co&amp;v=1.3 |
| 8d23af49-296a-4777-8e31-8b2c2dedad26 | Pulfrich, A. (2013). Namdeb Diamond Corporation Limited Marine Monitoring Programme: Offshore licences. Dataset published by AfrOBIS; consulted via iOBIS |
| a56cc5fc-9973-438d-a793-cdfd860bfef7 | Wacasey J (2013): DFO Zoobenthos data from upper Frobisher Bay, 1967 to 1973. v4.4. Fisheries and Oceans Canada. Dataset/Occurrence. http://iobis.org/mapper/?resource_id=2669 |
| c8eecb00-b65f-45c5-b04e-da9bd5dc18e0 | Fabio Lang da Silveira and Rubens M. Lopes Benthos of Paranagua Bay Region. Sao Paulo City, 24 December 2009, Version 1.0 |
| eae87607-a740-4f85-abf8-2af89764355b | Brulotte S, NozÃ¨res C (2023). DFO Quebec Region Biodiversity of the whelk (Buccinum) dredge survey in the St. Lawrence Estuary. Version 1.4. Fisheries and Oceans Canada. Samplingevent dataset. https://ipt.iobis.org/obiscanada/resource?r=dfo_que_est_bu&amp;v=1.4 |
| 5fbbc3e4-e951-4aa2-a16c-ac9258933d6c | Rumohr, H. Historical data on invertebrates from the Baltic Sea and Gdansk Bay. Christian-Albrechts-University Kiel; Leibniz Institute of Marine Sciences; Marine Ecology Division; Benthos Ecology section, Kiel, Germany. |
| 51078b37-d4a9-4a1d-9c94-92ee60299a3d | NA |
| f2648939-cd1e-4fe5-ac85-400ed753aa37 | (2018): Types_collection_Carcinolgy_MN_UFRJ. v2.0. Tropical and Subtropical Western South Atlantic OBIS. Dataset/Occurrence. http://doi.org/10.25607/b04qsc |
| 4f4d7534-c08d-427f-8fed-9e955c8242ce | B.P. Brooke, C.D. Woodroffe, M.Linklater, M.A.McArthur, S.L.Nichol, B.G.Jones, D.M.Kennedy, C.Buchanan, M.Spinoccia, R.Mleczko, A.Cortese, I.Atkinson and  M.Sexton, 2010. Geoscience Australia Record 2010/26. Geoscience Australia, Canberra. 125pp |
| 9d2f7b40-7028-432f-b022-52d6e2f6a47f | Cunha M E, Quintela F (2021): Assessment of zooplankton at the site of the sinking of the N.R.P. "S. Miguel". v1.4. IPMA - Instituto PortuguÃªs do Mar e da Atmosfera. Dataset/Samplingevent |
| 39b479a3-639e-48d2-9287-cc2580124e11 | Institut franÃ§ais de recherche pour l'exploitation de la mer, (2018). IFREMERPMN12018 SO239. Available : DeepData, International Seabed Authority https://data.isa.org.jm/ Accessed: [YYYY-MM-DD]. |
| eb5a052f-428e-4a6d-8058-3785c9356df7 | Citation_Information: Originator: K.N. Kosobokova Publication_Date: 1982 Title: Composition and distribution of the biomass of zooplankton in the central Arctic Basin 1975, 1976, 1977 Geospatial_Data_Presentation_Form: document |
| a82b670a-46b9-4f96-975e-4be51e0bb42d | Baird, H., Miller, K., Stark, J.S. (2012, updated 2017) Resilience of Antarctic marine benthic invertebrates and the ecological consequences of environmental change - Amphipod Data Australian Antarctic Data Centre - CAASM Metadata. |
| f180f1e9-817b-49f7-b5f4-04e1cc193f48 | Romagnan, J.B., Jalabert, L., Llopiz Monferrer, N., Elineau, A., Dimier, C., Picheral, M., Searson, S., Kandels-Lewis, S., Tara Oceans Consortium Coordinators, Karsenti, E., Pesant, S., Irisson, J.O., Lombard, F., Stemmann, L., Gorsky, G.; (2020) Occurrence of Eukaryotes in the size fraction &gt;300 micrometres, collected worldwide during the Tara Oceans expedition (2009-2013) using a Bongo net and analysed on a ZooScan imaging platform https://dx.doi.org/10.14284/518 |
| 739c0486-3adf-4e88-9f8d-f0b48ea9b5ad | NA |
| 04e3fd32-b08b-4806-a016-d2dff52ae55a | Jintsu-Uchifune, Y., Yamamoto, H. (2016) Marine organism occurrence data of the Asia-Pacific region extracted from literature. Available at http://www.godac.jamstec.go.jp/bismal/e/S9-5_Asia-Pacific. Accessed on yyyy-mm-dd. |
| 1b92d97a-3a1d-4bbf-9304-c56ba34ea041 | Fortier, L. Darnis, G. (2006) Vertical distribution of arctic mesozooplankton in Franklin Bay, southern Beaufort Sea, during winter 2003-04 |
| 5173de13-ca01-44e0-bb59-9b9dfda40266 | Massuti, Enric; GarcÃ­a, Cristina; Guijarro, Beatriz; Quetglas, Antoni; Gil de Sola, Luis; Instituto EspaÃ±ol de OceanografÃ­a (IEO); Spain; (2017): Demersal and mega-benthic species from the MEDITS (Mediterranean International Bottom Trawl Survey) program at the Spanish continental shelf and upper slope between 1994 and 2009. https://dx.doi.org/10.14284/420 |
| 7eb2ccad-2063-4ea8-b4e3-164dbc852fce | Danis, B., Christiansen, H., Guillaumot, C., Heindler, F., Houston, R., Jossart, Q., Lucas, K., Moreau, C., Pasotti, F., Robert. H., Wallis, B., SaucÃ¨de, T., [year accessed]. The Belgica 121 expedition to the Western Antarctic Peninsula: a high resolution biodiversity census. |
| 44513b57-0963-4668-a2ff-5f55c90fbc1b | NA |
| 7765772f-4817-4cb1-a87a-e5a6a885786a | Olivier M., CarrÃ© C., Buecher E., Braconnot J-C., MoitiÃ© M, Prejger F., Gasparini S., Stemmann L. (2015) Temporal evolution of zooplankton from 1959 to 2010 from Regent net in the Northwestern Mediterranean Sea |
| fec77f2d-d241-4b1d-8a93-f76b535e596b | Oug, E. &amp; B. Rygg (2000). Macrobenthos data from the Norwegian Skagerrak coast. Norwegian Institute for Water Research (NIVA) and Norwegian Pollution Control Authority. |
| b8439c16-2820-48e9-8ea0-7ccbd71b8805 | Davies C (2023): The Australian Zooplankton Database (1938 onwards). v1.8. CSIRO National Collections and Marine Infrastructure (NCMI) Information and Data Centre (IDC). Dataset/Occurrence. https://www.marine.csiro.au/ipt/resource?r=imos_azd_data&amp;v=1.8 |
| 857a8ddb-d144-416e-bc61-5f58280a0a01 | WÄ™slawski J. M. (1997). Svalbard tidal zone data. Polish Academy of Sciences; Institute of Oceanology (IOPAS), Poland. |
| bd967b0f-b665-4e07-9739-cae14726dc40 | Oleg Kachalov Ukrainian Scientific Centre of Ecology of the Sea (UkrSCES) |
| dd67ceb5-86b4-4841-a223-e668c1909dc6 | Australian Antarctic Data Centre. RMT Trawl catch from the 2005/06 V3 BROKE-West voyage - Zooplankton. |
| 856cfcd0-765d-4b5b-ba23-f371bd1da590 | Fonda-Umani, S. (2014). Meiobenthos North Adriatic-INTERREG-FVG-Projects. University of Trieste; Marine Biology Laboratory. https://doi.org/10.6092/f2baabe2-9891-4a1b-aeaf-de60fd91ee33 |
| 897b06c4-72f3-4e62-874c-71787b0a25f7 | Head, E.J.H. (2013). Ring net samples collected as part of a JGOFS cruise in the North Atlantic during the fall of 1992. Version 1 In OBIS Canada Digital Collections. Bedford Institute of Oceanography, Dartmouth, NS, Canada. Published by OBIS, Digital http://www.iobis.org/. Accessed on â€“INSERT DATE |
| d0dc72dc-0a2c-42f3-8ef8-e9c21e0d9b76 | National Institute of Aquatic Resources (Aqua) - DTU, Denmark; International Council for the Exploration of the Sea (ICES), Denmark (2016): Historic data (1908-1963) of benthic macrofauna from the Limfjord, Denmark |
| c07f3759-f350-4fed-856e-efda91fc4bfc | Van Avermaet P.; Coomans A.; Laboratoria voor Morfologie en Systematiek; Museum voor Dierkunde. RUG: Belgium; (2016): Epiphytic meiofauna and nematode community from seagrasses in Gazi and Nyali (Kenya) sampled in August 1989. (http://www.vliz.be/en/imis?module=dataset&amp;dasid=4426) https://dx.doi.org/10.14284/115 |
| 38e5dd79-5e2a-40aa-8638-7b72c8acbbae | Alexei Pinchuk (Unpublished) Arctic Ocean Chuckchi Sea August 1953-1954 Zooplankton vertical stratified collections on board of the Russian R/V Lomonosov, program ANII A-65 |
| 7967ece3-b59b-4dac-9615-b90cb65773bc | Teaca, A.; Begun, T.; Muresan, M.; National Research and Development Institute for Marine Geology and Geoecology â€“ GeoEcoMar, Romania (2016): Benthos data collected in the Black Sea during the Leg 2 EROS Cruise in 1997 |
| b41cd728-afdc-4b9c-ba42-43c265253ff5 | Zenetos A (2015): Kyklades-data of the Central Aegean Sea. |
| 36c1ed77-8e1b-4286-b1f0-cf8ddce930f4 | NA |
| c9ef68c5-b82b-4545-9d7c-aeaebcc5e99f | Mogias, A &amp; Kevrekidis, T; Spatial distribution of the macrobenthic fauna in Laki Lagoon (Evros Delta, N. Aegean Sea); Democritus University of Thrace; Department of Primary Education. Http://www.vliz.be/imis/imis.php?module=dataset&amp;dasid=2972 |
| d1b61ade-eb7f-437d-ada1-1cf2298ef4e9 | Althaus F (2023): CSIRO, Cruise SS200507, Benthic Biodiversity, Western Australia, 2005. v1.9. CSIRO National Collections and Marine Infrastructure (NCMI) Information and Data Centre (IDC). Dataset/Occurrence. https://www.marine.csiro.au/ipt/resource?r=csiro_ss200507&amp;v=1.9 |
| 17cb6ba1-1b99-4715-9d8f-1a435537928a | Cattrijsse, A. ; Marine Biology Research Group - Ugent, Belgium (2004). Spatial and temporal epibenthos and hyperbenthos variations at the Belgian Continental Shelf monitoring stations. Http://www.vliz.be/nl/imis?module=dataset&amp;dasid=156 |
| 2f9a8b1c-1e84-4943-a0b7-d43dd258b18f | Oleg Kachalov Ukrainian Scientific Centre of Ecology of the Sea (UkrSCES) |
| c393d973-4791-45b8-bb2b-a97879a6b9cb | Mogias, A &amp; Kevrekidis, T; Monthly variation in the macrozoobenthic community structure in Laki Lagoon (Evros Delta, N. Aegean Sea); Democritus University of Thrace; Department of Primary Education. Http://www.vliz.be/imis/imis.php?module=dataset&amp;dasid=2974 |
| 9106e863-8a29-4bd2-8983-fe7a071760cb | Bossicart M.; Daro M.H.; Mommaerts J.P.; Polk P; Laboratorium voor Ekologie en Systematiek. VUB: Belgium; (2015): Zooplankton studies in the Southern Bight of the North Sea between 1971 and 1974. https://dx.doi.org/10.14284/5 |
| 40d53f34-ba5c-477a-a9f7-6b221b17c6ff | Ocean Mineral Singapore Pte. Ltd., (2018). OMSPMN12018 NUS Data. Available : DeepData, International Seabed Authority https://data.isa.org.jm/ Accessed: [YYYY-MM-DD]. |
| bb8a2596-a3e6-4d2d-99cc-2680a480d4d3 | Espinosa-Leal L (2021): anfipodos_mopex. v1.0. Universidad de ConcepciÃ³n e Instituto Milenio de OceanografÃ­a (IMO). Dataset/Samplingevent. http://ipt.iobis.org/esp-obis/resource?r=anfipodos_mopex&amp;v=1.0 |
| 7cacae49-e435-449e-82ae-9a45f48821ca | Citation_Information:Originator: Douglas R. Redburn Publication_Date: 19740500 Title:The Ecology of the Inshore Marine Zooplankton of the Chukchi Sea near Point Barrow, Alaska Geospatial_Data_Presentation_Form: document |
| afa5b0e8-826d-4433-b698-beb176ef7880 | Flanders Marine Institute (VLIZ), Belgium (2022): LifeWatch observatory data: zooplankton observations in the Belgian Part of the North Sea https://dx.doi.org/10.14284/584 |
| 0e40e8a9-3210-462a-9215-4aff0ee607f2 | Australian Antarctic Data Centre. RMT Trawl catch from the 2005/06 V3 BROKE-West voyage - Fish. |
| 7084556b-d220-4c73-a258-525525bdd6cd | Pavloudi C., Christodoulou M., Mavidis M. (2016). Comparative study of the organismic assemblages associated with the demosponge Sarcotragus foetidus Schmidt, 1862 in the coasts of Cyprus and Greece. |
| b56e4c0d-7688-4b09-9a1e-34a9e6430d51 | Roy V (2021): DFO Quebec Region Coastal biodiversity of the benthic epifauna of the St. Lawrence Estuary (2018-2019). v1.2. Fisheries and Oceans Canada. Dataset/Samplingevent. https://doi.org/10.26071/ogsl-c2a02113-e69c |
| 955179d9-77a5-4bdf-a796-1a3d1ee4b2b8 | Belley R, Boulanger, M-P, NozÃ¨res C, Roy V, Sean A-S (2023). DFO Quebec Region Mingan Archipelago Icelandic Scallop survey. Version 1.0. Fisheries and Oceans Canada. Occurrence dataset. https://ipt.iobis.org/obiscanada/resource?r=dfo_que_min_ci&amp;v=1.0 |
| 22fcfe7e-1923-4928-9a06-2aa2326227e8 | NA |
| 34370444-238d-4908-9ffb-73d98e2057b0 | Originator: Mikael K Sejr Publication_Date: 2009 Title: Macrobenthic species composition and diversity in the Godthaabsfjord system, SW Greenland Geospatial_Data_Presentation_Form: document Series_Information: Series_Name: Polar Biology Issue_Identification: 33;4 421-431 |
| 4c130a18-8fa7-4cae-b710-00da365d7f34 | Caroll A (2023): Ecology / Infaunal morphospecies identifications from the Vlaming Sub-basin (GA0334), Australia (2012-2013). v1.10. CSIRO National Collections and Marine Infrastructure (NCMI) Information and Data Centre (IDC). Dataset/Samplingevent. https://www.marine.csiro.au/ipt/resource?r=ga_vlaming_infauna&amp;v=1.10 |
| 930000f4-81ef-421b-8884-042abdf8fbc8 | CSIRO - Southern Surveyor voyage SS 10/2005, benthic biodiversity of the deep continental shelf and slope in Australia's SW region |
| 9df9e620-d008-4cd2-82d6-aecc6af23644 | Nasi F., Auriemma R., &amp; Cibic T. (2018): Macrobenthos collected in the Po River Delta - North Adriatic Sea (RITMARE Project) in December 2014. OGS (Istituto Nazionale di Oceanografia e di Geofisica Sperimentale), Division of Oceanography. Italy. https://doi.org/10.6092/gtc1-bp60 |
| 33b5f094-38b0-4ad2-82d8-1e57f9bc291c | NIWA (2015). Antarctic Biodiversity Studies 2006 - Ross Sea, Scott Island, and Balleny Islands (TAN0602). Southwestern Pacific OBIS, National Institute of Water and Atmospheric Research, Wellington, New Zealand, 1061 records, Online http://nzobisipt.niwa.co.nz/resource.do?r=tan0602 released on April 17, 2015. |
| 2a93fe93-2fdc-41b3-b36a-5deb261c02db | Swadling, K.M., Eriksen, R.S., Beard, J.M. and Crawford, C.M. (2018) Zooplankton sampling in the coastal waters of south eastern Tasmania. Institute for Marine and Antarctic Studies, University of Tasmania. Data accessed at http://metadata.imas.utas.edu.au/geonetwork/srv/en/metadata.show?uuid=2f950283-8f4b-41b7-a379-a3b3af0c48e0 on (access date). |
| 069b16f7-3305-4ef5-9cd1-cefceae25e92 | Nikolopoulou Stamatina, Dimitriou D. Panagiotis and Tsikopoulou Irini (2016). Digitation of The fishery grounds near Alexandria. X Amphipoda benthonica. Notes and Memoirs No 11. Hydrobiology and Fisheries Directorate, 1936, Egypt |
| 36144583-0ec3-43af-8f73-0b2f9407bd65 | Sorbonne UniversitÃ©/CNRS - Institut de la Mer de Villefranche (IMEV), Sorbonne UniversitÃ©/CNRS - Laboratoire d'OcÃ©anographie de Villefranche (LOV); 2020; Plankton community in Juday-Bogorov (330Âµm) net, Point B, Villefranche-sur-Mer, France https://dx.doi.org/10.14284/478 |
| c7796179-94ff-4620-98a5-ce066e83f7ef | KÄ™dra M. (2006). Kongsfjorden monitoring data â€“ grid â€“ 2006. Polish Academy of Sciences, Institute of Oceanology (IOPAS), Poland. |
| d0d3ccce-e385-4e37-912a-a12133c7b9af | Petrov A., Povchun A.S., Zolotrev P.N. Initial data set (1980-1989) on abundance and biomass of soft-bottom macrozoobenthos of Karkinitsky gulf, Western Crimea, Ukraine. Institute of Biology of Southern Seas, Ukraine. |
| e60b0b08-b1f6-4472-bc01-ef6b8c760863 | Maclellan, D.C. and J.B. Sprague. 2016. FRB: Bottom fauna of Saint John Harbour and estuary as surveyed in 1959 and 1961. Version 1 In OBIS Canada Digital Collections. Bedford Institute of Oceanography, Dartmouth, NS, Canada. Published by OBIS, Digital http://www.iobis.org/. Accessed on â€“INSERT DATE |
| 379f8b86-e4a1-4b50-90e3-9b0ff4450671 | Australian Antarctic Data Centre. RMT Trawl catch from the 1984/85 V5 SIBEX2 voyage. |
| bc496dae-96c7-45db-a82b-27d51d1258ce | Ecopetrol S.A. , AquabiÃ³sfera S.A.S. (2023): Biodiversidad asociada al Estudio de Impacto Ambiental para el Ã¡rea de perforaciÃ³n exploratorioa Marina - APEM RubÃ­. v1.0. Ecopetrol S.A.. Dataset/Samplingevent. https://doi.org/10.15472/qn3c7g |
| fb706b08-4826-485e-abfa-cd96905d8e3e | Wijsman, J.; Koninklijk Nederlands Instituut voor Onderzoek der Zee - NIOZ Yerseke: Netherlands; (1995): Spatial distribution in sediment characteristics and benthic activity on the northwestern Black Sea shelf: macrobenthos. Marine Data Archive https://dx.doi.org/10.14284/525 |
| 35425466-4e93-48b8-9e91-b8a76c026ee1 | Dewarumez J.-M. &amp; Desroy N. - 2000. Eastern Channel dataset. Station Marine de Wimereux, France. |
| accd827e-e428-4262-b289-be8064e0434d | MartÃ­nez Arbizu, P. &amp; Veit-KÃ¶hler, G. (2002). ANDEEP-1: Antarctic deep-sea meiofauna. Forschungsinstitut Senckenberg; Deutsches Zentrum fur Marine BiodiversitÃ¤tsforschung (DZMB), Germany. |
| ee924936-1a03-400d-a21d-16a2a4587bda | R. Auriemma, F. Nasi, &amp; Del Negro P. (2018): Macrobenthos monitoring in the Trieste harbour, North Adriatic Sea (Port Authority) in June 2013 and March 2015. OGS (Istituto Nazionale di Oceanografia e di Geofisica Sperimentale), Division of Oceanography. Italy. https://doi.org/10.6092/04wy-4b44 |
| ba135f30-d828-4ed4-a6d2-84101293c49a | NA |
| 6f017f69-5d59-4253-b73d-d9338cc51646 | Federal Institute for Geosciences and Natural Resources of Germany, (2017). BGRPMN12017 Env Template MANGAN2010. Available : DeepData, International Seabed Authority https://data.isa.org.jm/ Accessed: [YYYY-MM-DD]. |
| a11a7cff-6f7e-4d34-8d39-fc86cb8783db | Rumohr, H. The Baltic Expedition 1901 of the German sea fisheries association. Christian-Albrechts-University Kiel; Leibniz Institute of Marine Sciences; Marine Ecology Division; Benthos Ecology section, Kiel, Germany. |
| f780666c-3aaf-45a1-a3f5-5fa6ae279f02 | Australian Antarctic Data Centre. RMT Trawl catch from the 1983/84 V5 ADBEX II voyage. |
| 45e7c641-c317-4cae-8b01-0b00cae1029e | Oleg Kachalov Ukrainian Scientific Centre of Ecology of the Sea (UkrSCES) |
| b364da26-0d6a-4ad7-a3bf-c88abc5c1958 | Oleg Kachalov Ukrainian Scientific Centre of Ecology of the Sea (UkrSCES) |
| 96f4a5d3-3e3a-4ccb-8343-b1db99074b1f | Michel L (2019): Staying cool also has consequences: increased sea ice cover disrupts food web structure in East Antarctica-Data. v1.1. SCAR - AntOBIS. Dataset/Occurrence. https://ipt.biodiversity.aq/resource?r=ddu_isotopes_verso_2013_2015&amp;v=1.1 |
| 162aa046-c8c0-46b8-8600-23fd93a0acc0 | Kennedy, M.K., Spry, J.A. 2011. BioChem: Zooplankton collected from the Gully, summer 2006 and 2007. OBIS Canada Digital Collections. OBIS Canada, Bedford Institute of Oceanography, Dartmouth, Nova Scotia, Canada, Version 1, Digital, retrieved from http://iobis.org/. |
| 4b667f4a-2c2c-401e-8fbb-9be0bdf41421 | Meyer H K (2022): Morphotaxa Occurrence Data on Schulz Bank 2017-2018, Arctic Mid-Ocean Ridge. v1.2. Deep-sea OBIS node. Dataset/Samplingevent. https://ipt.iobis.org/obis-deepsea/resource?r=morphotaxa_occurrence_schulz_arctic&amp;v=1.2 |
| 31d93350-097e-456c-8e12-8af658c1107b | Indian Ocean Marine Fauna Voucher Specimens Collections (CMLRE), Kochi, India. Version 1.0. Centre for Marine Living Resources &amp; Ecology. Occurrence dataset (IndOBIS). |
| 1ed6515b-6587-448b-8987-1646acbb4641 | Zooplankton collected in the Mediterranean Sea in 1959 on board the R/V Akademik S. Vavilov. Institute of Biology of the Southern Seas of the National Academy of Sciences of Ukraine. |
| 7e5277ae-fca3-455c-b2aa-4a0c4f4a7a2e | Indian Ocean Biodiversity Information System (IndOBIS)- Distribution records of marine organisms from the Indian Ocean. |
| 32d3fa59-a675-4044-aafe-f360d2a52f8a | Occhipinti-Ambrogi A., 2002: Cesenatico dataset. Department of Ecologia del Territorio , University of Pavia, Italy |
| 7fa785bd-da15-43f3-8b74-2ccc76396381 | Jim Hamilton. (2014). BioChem: Canadian Arctic ThruFlow Study plankton collection. Version 1 In OBIS Canada Digital Collections. Bedford Institute of Oceanography, Dartmouth, NS, Canada. Published by OBIS, Digital http://www.iobis.org/. Accessed on â€“INSERT DATE |
| c71febef-1155-47c8-a9d1-bc46f911d2e9 | Jian L.; Heip C.; Fundamental and Applied Marine Ecology Post Graduate Program (FAME). VUB: Belgium; Delta Institute for Hydrobiological Research: The Netherlands; (2017): Spatial distribution of meiobenthos in the Voordelta (October 1988). (http://www.vliz.be/en/imis?module=dataset&amp;dasid=4233) https://dx.doi.org/10.14284/275 |
| 1cdb4401-902f-4f3d-aaee-50ab0258aa27 | UK Seabed Resources Ltd. , (2015). UKSRLPMN12015 Env Templaye Megafauna 032016. Available : DeepData, International Seabed Authority https://data.isa.org.jm/ Accessed: [YYYY-MM-DD]. |
| 69be003f-bb8e-4bdd-ac63-6d2dcc119222 | Kendall M., 1996: Arctic soft-sediment macrobenthos. Plymouth Marine Laboratory, UK |
| 94ce69e6-6cc8-4f51-a804-8b9268238cdf | NA |
| 1a4daaac-e207-4648-804e-aaf5efa7a0a5 | The Danish Biodiversity Information Facility, Marine Benthic Fauna List, Island of LÃ¦sÃ¸, Denmark. https://doi.org/10.15468/ty0smg |
| b4853d39-b86a-43b3-9ad4-b8e84d95fd6a | Federal Institute for Geosciences and Natural Resources of Germany, (2017). BGRPMN12017 Biodiveristy. Available : DeepData, International Seabed Authority https://data.isa.org.jm/ Accessed: [YYYY-MM-DD]. |
| f8792cd3-f0b5-42e9-94b8-d42879206527 | Oleg Kachalov Ukrainian Scientific Centre of Ecology of the Sea (UkrSCES) |
| a72786ef-6af6-451c-b339-d34d13e8c978 | Sezgin, M. 1999. Macrobenthos data from 1999. Sinop University Fisheries Faculty (SNU-FF). |
| 4d2a31a3-bb76-4ffe-a2f9-817e975f6ba9 | Diveboard - Scuba diving citizen science observations. Online at http://www.diveboard.com and http://ipt.diveboard.com/resource.do?r=diveboard-occurrences. https://dx.doi.org/10.15468/tnjrgy |
| bc699d5e-059e-4a45-bc0c-18a0faaa295a | Wade, E.J. 2011. DFO Gulf Region Snow Crab Research Trawl Surveys. Version 1. In OBIS Canada Digital Collections. Bedford Institute of Oceanography, Dartmouth, NS, Canada. Published by OBIS, Digital http://www.iobis.org/. Accessed on â€“INSERT DATE |
| 0c6317fc-ea7c-420f-ab09-80928d564650 | Teaca, A.; Begun, T.; Muresan, M.; National Research and Development Institute for Marine Geology and Geoecology â€“ GeoEcoMar, Romania (2016): Historical benthos data from the Romanian Black Sea Coast between 1954 and 1968 |
| 9cdcfc4a-791f-45b4-8923-ed2011602e6c | Jerez-Guerrero M, Criales-HernÃ¡ndez M I (2021): Zooplancton del estrecho de Bransfield y alrededores de Isla Elefante, verano austral 2019-2020, durante la VI ExpediciÃ³n CientÃ­fica de Colombia en la AntÃ¡rtica. v2.3. Universidad Industrial de Santander. Dataset/Samplingevent. https://doi.org/10.15472/c3ggag |
| 41dd1b13-c30d-44ce-b4ad-ba0e648b4351 | Yuzhmorgeologiya, (2015). YUZHPMN12015 Biodata B6. Available : DeepData, International Seabed Authority https://data.isa.org.jm/ Accessed: [YYYY-MM-DD]. |
| 90faff6c-f5fe-4997-bd48-5b4651f5a11f | CSIRO - Prawn bycatch survey, Torres Strait, Gulf of Carpentaria and Arafura Sea, 1997-1998    CSIRO Division of Marine Research (1997). FRV Southern Surveyor. Cruise Report SS 08/97. Miscellaneous Publication. CSIRO Division of Marine Research, 21 pp. |
| 926c3278-ca1a-495b-80d0-fbf494f54699 | NA |
| bf57b87d-08f0-4a70-a541-bb328fe762d3 | NA |
| aefdfcac-74b8-42ac-88c6-ae67133ee4f3 | NA |
| 8c8af6f1-8ba3-4e2e-ae01-8436259fcea9 | Marquez B, Troccoli L, MarÃ­n B, DÃ­az-Ramos J R (2022): IOV-UDO-ZOOPLANCTON-PLAYA COLORADA. v1.6. Caribbean OBIS Node. Dataset/Samplingevent. https://ipt.iobis.org/caribbeanobis/resource?r=iov_udo_zooplancton_pc&amp;v=1.6 |
| 779199d7-d691-41e5-aa38-4acd4e8098f6 | UK Seabed Resources Ltd. , (2015). UKSRLPMN12015 Env Template Macrofauna 032016. Available : DeepData, International Seabed Authority https://data.isa.org.jm/ Accessed: [YYYY-MM-DD]. |
| 1fe0bc91-a326-4fc6-a7c3-4b0cdd2fd9b2 | NA |
| 1d7b68ff-72ec-4a3a-b8e5-0e369c9696a3 | NA |
| 51e93be3-9850-4fbb-9a07-88813527e942 | NA |
| 7a571da5-07d4-493f-a695-b9d0291dba1a | Vatova A., 1936: Fauna Bentonica dataset; Instituto di Biologia Marine per lâ€™Adriatico, Venezia, Italy |
| 0ef4bb91-c27c-4e90-b679-c170252dddb6 | 45: Marine Biological Association of the United KingdomÂ (MBA); Continuous Plankton Recorder Survey (CPR) (2023): 2021 Natural Resources Wales (NRW) Skomer zooplankton survey. v1.1. Marine Biological Association. Dataset/Samplingevent. 10.17031/64b9039539533 |
| 2dffebfc-8132-445e-a127-3a360208a7b3 | Bello S, NuÃ±ez S, SanjuÃ¡n MuÃ±oz A, Linero Rosado C, Paramo J, Ardila N, Franco A, Polo C (2021): EvaluaciÃ³n del macrobentos de la plataforma continental del Caribe colombiano. v1.0. Universidad del Magdalena. Dataset/Occurrence. https://doi.org/10.15472/yvwdf6 |
| aa60750c-4921-43e1-8146-7b16aad675ac | Coral Reef Ecosystem Division (CRED), Pacific Island Fisheries Sciences Center, NOAA National Marine Fisheries Service. 2011. CRED Rapid Ecological Assessment of Benthic Habitat Cover in the Pacific Ocean 2005-2010. Coral Reef Ecosystem Division, Honolulu, HI.Retrieved from http://www.usgs.gov/obis-usa/. |
| 796780d8-2753-48eb-8dc8-02f16a023e59 | Kuznetsova, N., Somov, A., &amp; Pakhomov, E. (2022). Juday Net Zooplankton Data from the 2020 Gulf of Alaska International Year of the Salmon Expedition (Version 1.0) [Data set]. North Pacific Anadromous Fish Commission. https://doi.org/10.21966/G9WR-VS33 |
| 4eaf3ab8-463a-4c8d-838a-9b9c6ca4bfd3 | NA |
| 1518e369-0e28-4734-b85e-bc1c1ed4f00d | Ishida S (2016). Marine Invertebrata specimen database of Osaka Museum of Natutal History. National Institute of Genetics, ROIS. Occurrence dataset https://doi.org/10.15468/zhubgk accessed via GBIF.org on yyyy-mm-dd. |
| 6ca7564e-82dd-42ec-b75a-a4164c3dda29 | Kachalov, O.; Ukrainian Scientific Centre of Ecology of the Sea (UkrSCES); (2020): National Pilot Monitoring Studies 2017, Improving Environmental Monitoring in the Black Sea - Phase II (EMBLAS-II), ENPI/2013/313-169, (Macrozoobenthos) |
| 4945cc97-c830-42bd-aec0-036f74937b93 | NA |
| 2f6e3dbd-64b0-4371-9a67-6e98305a32e0 | Originator: Mikael K Sejr Publication_Date: 2000 Title:Macrozoobenthic community structure in a high-arctic east Greenland Geospatial_Data_Presentation_Form: document Series_Information: Series_Name: Polar BiologyIssue_Identification: 23:792-801 |
| 0fcb583e-e7b2-4bed-864c-f73d2d770439 | S. Kobyliansky, A. Orlov, and N. Gordeeva, "Composition of deepsea pelagic ichthyocenes of the Southern Atlantic, from waters of the range of the Mid-Atlantic and Walvis Ridges," Journal of Ichthyology 50 No.10, 932-949 (2010) |
| 376b6d39-629d-4df6-95a7-6765f72d8369 | AntoliÄ‡ B, Å½uljeviÄ‡ A, DespalatoviÄ‡ M, CvitkoviÄ‡ I (2021): Environmental impact assessments in the eastern part of Adriatic sea - species list of benthic invertebrates and phytobenthos (2000-2010) |
| e5ba539d-923f-42f6-9daf-2d36a7dfe13e | Australian Antarctic Data Centre (2018). RMT Trawl catch from 1985/86 V1 ADBEX III voyage. |
| a7a946da-3764-43c4-a84a-ffe8df855b37 | De Olazabal A., &amp; Civitarese G (2018): Mesozooplankton in the South Adriatic Sea (MEDGES Experiment) in March 2013. OGS (Istituto Nazionale di Oceanografia e di Geofisica Sperimentale), Division of Oceanography. Italy. https://doi.org/10.6092/001y-7x62 |
| ea742725-fd62-4520-8497-b5571d55efe6 | Rumohr, H., 1995: Kiel Bay intercalibration data set. Leibniz Institute of Marine Sciences, Marine Ecology Division, Germany |
| f0bbfd00-2631-4006-b6bc-5c88f166852c | NA |
| 190f3394-a704-413b-a430-26b91a555dcb | DFO. (2014). Entomostraca collected in and around the Strait of Belle isle during expeditions in 1923. Version 1 In OBIS Canada Digital Collections. Bedford Institute of Oceanography, Dartmouth, NS, Canada. Published by OBIS. http://www.iobis.org/. (consulted on [date]) |
| 5a6bb73e-8780-4859-ac05-84eb3875b930 | Kachalov, O.; Ukrainian Scientific Centre of Ecology of the Sea (UkrSCES); (2020): National Pilot Monitoring Studies Phyllophora August 2017, Improving Environmental Monitoring in the Black Sea - Phase II (EMBLAS-II), ENPI/2013/313-169, (Macrozoobenthos) |
| 16f543b9-df51-4164-970e-aafb8878fbce | NA |
| 748486a6-fef0-4eee-bdf3-40bba9e621ae | Tamara Cibic, Rocco Auriemma &amp; Federica Nasi (2018): Macrobenthos monitoring in the Mar Piccolo of Taranto in June 2013 and April 2014. OGS (Istituto Nazionale di Oceanografia e di Geofisica Sperimentale), Division of Oceanography. Italy. https://doi.org/10.6092/2bqm-v560 |
| f1ad2d12-8f7e-4b56-848c-ffa9c6103eb1 | Stockholm University, UmeÃ¥ University, Swedish Meteorological and Hydrological Institute, Gothenburg University, Swedish Agency for Marine and Water Management and Swedish Environmental Protection Agency (2022). SHARK - National marine environmental monitoring of zooplankton in Sweden since 1979 https://doi.org/10.15468/edkb5r |
| e8f8c72a-5a67-472a-b5ef-033e98369629 | De Pauw C.; De Coninck L.A.P.; Faculteit Wetenschappen. RUG: Belgium; (2016): Ecological study of the plankton in the port of Ostend in 1965. (http://www.vliz.be/en/imis?module=dataset&amp;dasid=4531) https://dx.doi.org/10.14284/194 |
| 7faf5339-04a6-4c1d-9770-62c42f8d2934 | Olivier M., Prejger F., Gasparini S., Stemmann L. (2015) Temporal evolution of zooplankton from 2004 to 2010 from WP2 net in the Northwestern Mediterranean Sea. |
| 286fc9ac-2812-49a0-9f76-f1e59d9777b2 | Kachalov, O., Ukrainian Scientific Centre of Ecology of the Sea (UkrSCES); (2020): National Pilot Monitoring Studies 2016, Improving Environmental Monitoring in the Black Sea - Phase II (EMBLAS-II), (Macrozoobenthos) |
| 723bda06-7421-4669-b25c-3f4f4b9bec78 | CSIRO - Southern Surveyor Voyage SS 02/2007, biodiversity survey for SE Marine Protected Areas including the Tasmanian Sea Mounts Marine Reserve |
| a9ac85d8-67a1-49bf-8ee8-815536022ce6 | Global Sea Mineral Resources NV, (2016). GSRPMN12016 MarBiol UGent. Available : DeepData, International Seabed Authority https://data.isa.org.jm/ Accessed: [YYYY-MM-DD]. |
| 7de27276-f9f7-40a2-bbd3-18512b50fe28 | Dos Santos, A.; Marraccini, A.; Stehle, M.J.; LourenÃ§o, P.; Pires, R.; Queiroga, H. (2017). Zooplankton and Ichthyoplankton of the North Western Portuguese Coast in May 2002 https://dx.doi.org/10.14284/465 |
| a2446547-4c94-4df3-a6dc-ccfcdcbb4596 | Universidad Antioquia &amp; Universidad CES (2017). Macroalgas, equinodermos y crustÃ¡ceos de la Isla Cayo Serranilla durante la ExpediciÃ³n Seaflower 2017 - Proyecto Colombia BIO. VersiÃ³n 1.0. 197 registros, aportados por: Quiceno P, Quang L, LondoÃ±o MH, Zapata JL. Conjunto de datos/Registros biolÃ³gicos. |
| 2b43868c-0ddb-4932-b936-53c21fad5abd | (2018): Extreme_South_BR. v2.0. Tropical and Subtropical Western South Atlantic OBIS. Dataset/Occurrence. http://doi.org/10.25607/7u22zf |
| 57f47b43-dcf8-4c23-a56b-719cec84f8f3 | Bernier D, Bourdages H, NozÃ¨res C, Vanier C, Boulanger M, Isabel L (2023). DFO Quebec Region Ecosystemic bottom trawl survey 1990-2005. Version 1.1. Fisheries and Oceans Canada. Samplingevent dataset. https://ipt.iobis.org/obiscanada/resource?r=quebec_occurence&amp;v=1.1 |
| 5d6813fd-a0e2-4d9f-93c0-2bb399fbad84 | Oleg Kachalov Ukrainian Scientific Centre of Ecology of the Sea (UkrSCES) |
| 2d1fac84-2333-4021-ad88-f2af59683dcb | Kevrekidis, T; Monthly variation in the macrozoobenthic community structure in Monolimni Lagoon (Evros Delta, N. Aegean Sea); Democritus University of Thrace; Department of Primary Education. http://www.vliz.be/imis/imis.php?module=dataset&amp;dasid=2975 |
| 512f12ff-d7f9-49d0-b9e3-b9ceb68d837a | De Pauw N.; Rijksuniversitair Centrum Antwerpen. RUCA: Belgium; (2015): Study on plankton at the port of Ostend in 1965. (http://www.vliz.be/en/imis?module=dataset&amp;dasid=5154) https://dx.doi.org/10.14284/72 |
| e830dc46-156e-4646-ba4c-6d305580f167 | NA |
| 26fb9cdc-1f1a-453a-b074-ad7f395d1122 | Marine species from published papers of the Kenyan EEZ in the period 1975-2014, collected by Kenya marine and Fisheries Research Insitute |
| bb0b9375-c875-4cb6-8889-6f783e1015be | Nauru Ocean Resources Inc., (2022). NORIPMN12022 Env Template BIO. Available : DeepData, International Seabed Authority https://data.isa.org.jm/ Accessed: [YYYY-MM-DD]. |
| 61a1cc7e-9fa7-4006-b79e-e04f0c80fb08 | NA |
| 2020529f-3220-4f4f-9e0f-bd4edc3df85b | China Ocean Mineral Resources Research and Development Association, (2018). COMRAPMS12018 zooplankton. Available : DeepData, International Seabed Authority https://data.isa.org.jm/ Accessed: [YYYY-MM-DD]. |
| 7b3bf64f-e4c1-48e3-b136-70177ee07fa8 | Ahrens M, Osorio Cardoso J S, Bernal de la Torre L A, Prado Valencia K, Molina F, Lopez Sanchez M, Andramunio Acero C P, Dorado Roncancio J (2021): Levantamientos portuarios de fitoplancton, zooplancton, perifiton y macrozoobentos en el PacÃ­fico colombiano. v1.0. FundaciÃ³n Universidad de BogotÃ¡ Jorge Tadeo Lozano. Dataset/Samplingevent. https://doi.org/10.15472/fxemvo |
| 2741dff2-017c-4d2d-8e3a-b04fc06619b9 | Bax N (2023): Habitat and fisheries production in the south east fishery ecosystem - Zooplankton records from three Southern Surveyor voyages, shelf waters, South East Australia (1994-1996). v1.11. CSIRO National Collections and Marine Infrastructure (NCMI) Information and Data Centre (IDC). Dataset/Samplingevent. https://www.marine.csiro.au/ipt/resource?r=csiro_se_aus_zooplankton&amp;v=1.11 |
| 061760d0-53ed-4e5a-8e94-59100015b28d | Encana Corporation 2015. Encana: Deepwater benthic taxa collected as part of a survey of Stonehouse during August-September 2002. Version 1 In OBIS Canada Collections. Bedford Institute of Oceanography, Dartmouth, NS, Canada. Published by OBIS. http://www.iobis.org/. (consulted on [date]) |
| 4732665a-947e-426c-b58c-25a10d98d35d | dos Santos A, Marraccini A, Niza H (2022): Zooplankton of the South and South-Western Portuguese Coast in Winter 2006-07. v1.9. IPMA - Instituto PortuguÃªs do Mar e da Atmosfera. Dataset/Samplingevent |
| 06aaf685-c9db-4ff0-b45f-bf7b78df1c2f | Willems K.A.; Vanosmael C.; Claeys D.; Vincx M.; Heip C.H.R.; Marine Biology Section, Zoology Institute. RUG: Belgium; (2016): Macro-and meiobenthos of a sublittoral sandbank in the Southern Bight of the North Sea (5 September 1978). (http://www.vliz.be/en/imis?module=dataset&amp;dasid=4229) https://dx.doi.org/10.14284/132 |
| dadd8252-0f72-417a-be62-9bde09e93c61 | Fabio Lang da Silveira and Rubens M. Lopes Marine Biota Along the West Coast of Ceara State, Northeast Brazil. WSAOBIS, SÃ£o Paulo, June 2008, Version 1, Publication. |
| 276b09b1-0e57-4128-a8b1-c15998da3081 | Tecchio, S.; Ramirez-Llodra, E. (2018): Megafaunal data from the 2009 BIOFUN trans-Mediterranean deep-sea cruise https://dx.doi.org/10.14284/311 |
| d3f51225-e412-4752-beb9-6ab415bf0c81 | Davies, Claire H.,Beckley, Lynnath E.,Richardson, Anthony J. (2022) Copepods and mixotrophic Rhizaria dominate abundances in the oligotrophic Indian Ocean. Deep Sea Research Part II: Topical Studies in Oceanography pp105136-. |
| 969cc685-b8dd-40ac-9419-7ec65a50820c | Sainte-Marie B, Roy V, NozÃ¨res C, Boulanger M-P (2023): DFO Quebec Region Baie Sainte-Marguerite Snow Crab Trawl Survey 2006-2009. v1.1. Fisheries and Oceans Canada. Dataset/Samplingevent. https://ipt.iobis.org/obiscanada/resource?r=dfo_que_bsm_co&amp;v=1.1 |
| 62b1e8fb-fb6d-461a-870c-a6445815112d | MarBEF (2006) MarBEF Publication Series data. Available online on EurOBIS. Consulted on dd-mm-yyyy. |
| 8c536d29-506c-49e3-8332-28818f175c79 | Institut franÃ§ais de recherche pour l'exploitation de la mer, (2015). COMRAPMS12015 ENV. Available : DeepData, International Seabed Authority https://data.isa.org.jm/ Accessed: [YYYY-MM-DD]. |
| 6951ef75-9c6d-4a89-8c3e-59d73d2c0bf2 | NA |
| 363961a9-9f45-46df-980b-0b076e66a296 | ICES Environmental Database (DOME), Zooplankton community. Available online at http://eggsandlarvae.ices.dk. ICES, Copenhagen. Consulted on yyyy-mm-dd. |
| 5d206e57-370c-453f-a882-b54d517294e7 | van der Reis, A. L., Beckley, L. E., Olivar, M. P., &amp; Jeffs, A. G. (2022). Nanopore short-read sequencing: A quick, cost-effective and accurate method for DNA metabarcoding. Environmental DNA, 00, 1â€“ 15. https://doi.org/10.1002/edn3.374 |
| 601b9550-48f5-403f-903a-e22712a90d7e | Deep Ocean Resources Development Co. Ltd., (2018). DORDPMN12018 Env Mn Bio. Available : DeepData, International Seabed Authority https://data.isa.org.jm/ Accessed: [YYYY-MM-DD]. |
| 928ef84a-129a-4e57-a484-f13bc9c94989 | (2022): Barcoding Dutch Harbor and St Paul's Marine Invertebrates. v1.2. United States Geological Survey. Dataset/Occurrence. https://www1.usgs.gov/obis-usa/ipt/resource?r=serc_invasions_pribilofs&amp;v=1.2 |
| 5ecfbf93-d5f7-4b0f-a766-efd2481a7457 | Gambi, C. &amp; Danovaro, R. (1997). Meiofauna of the North Adriatic Sea. Polytechnic University of Marche, Faculty of Sciences, Department of Marine Sciences, Italy. |
| bdbfda38-c0fd-483c-85eb-4202baff1e52 | Neville, C., Somov, A., Esenkulova, S., &amp; LaForge, R. (2022). Trawl Catch and Species Abundance data from the 2020 Gulf of Alaska International Year of the Salmon Expedition [Data set]. North Pacific Anadromous Fish Commission. https://doi.org/10.21966/4J6T-GB64 |
| cc62b9e8-dd4a-4638-8061-b72721568e33 | Le Bourg B, SaucÃ¨de T, Charpentier A, Lepoint G, Gan Y, Michel L N (2022): Stable isotope ratios and elemental contents of C, N and S in benthic organisms sampled during the PROTEKER 5 summer campaign in Kerguelen Islands (2016) - data. v1.4. SCAR - AntOBIS. Dataset/Samplingevent. https://ipt.biodiversity.aq/resource?r=proteker2016-event&amp;v=1.4 |
| f9ad999f-6460-404f-a594-189bafe3b52d | NA |
| 2b5f9b56-27bf-4000-8f95-3e6e703c6a85 | Gowlett-Holmes K (2023): CSIRO Marine Invertebrates Image Collection (MIIC). v1.31. CSIRO National Collections and Marine Infrastructure (NCMI) Information and Data Centre (IDC). Dataset/Occurrence. https://www.marine.csiro.au/ipt/resource?r=csiro_miic&amp;v=1.31 |
| dde3f81b-dc2f-4fb0-92de-a56fcdf6c63d | Danovaro, R. (1997). Meiofauna and nematodes from the Atacama slope and trench. Polytechnic University of Marche; Faculty of Sciences; Department of Marine Sciences, Italy. |
| 6862ae8c-db0d-44ef-9375-c50e50c2b74e | Hernandez N, Guerrero-Rios R (2022): Macrobentos de cuatro playas de alta energÃ­a ubicadas en la PenÃ­nsula de La Guajira, noroeste del Golfo de Venezuela. v1.7. Caribbean OBIS Node. Dataset/Samplingevent. https://ipt.iobis.org/caribbeanobis/resource?r=macrobentoslaguajira&amp;v=1.7 |
| 7afba27f-8547-4899-829e-b4bd25765322 | Friedlander A M, Goodell W, Salinas-de-LeÃ³n P, Ballesteros E, Berkenpas E, Capurro A P, CÃ¡rdenas C A, HÃ¼ne M, Lagger C, Landaeta M F, MuÃ±oz A, Santos M, Turchik A, Werner R, Sala E (2020): Argentinaâ€“Chile National Geographic Pristine Seas Expedition To The Antarctic Peninsula - Deep Sea Cam Data. v1.2. SCAR - AntOBIS. Dataset/Occurrence. https://ipt.biodiversity.aq/resource?r=natgeo_prist0cean_wap_deepseacam_2020&amp;v=1.2 |
| dd7b09c3-137c-443d-a145-2401f400c441 | Boles W (2022): Australian Museum Ornithology Collection - marine records. v1.12. CSIRO National Collections and Marine Infrastructure (NCMI) Information and Data Centre (IDC). Dataset/Occurrence. https://www.marine.csiro.au/ipt/resource?r=am_ornithology&amp;v=1.12 |
| 5ae7ac9f-0347-49d1-b022-7739dda78ccf | SWPRON, 2018. Data from: Scientific results of the New Zealand Government Trawling Expedition, 1907. Waite, E.R. (ed.). Records of the Canterbury Museum, Vol. 2, No. 2. |
| 544ffde0-fa23-4185-8a05-eac1d48edbe2 | NA |
| 14a2ca00-2e7c-4481-9817-2e84ae52ac2e | Mueller H (2022): Records of coral reef inhabiting Caprellidae from Bora Bora and Moorea, Society Islands (Crustacea: Amphipoda). v1.0. Southwestern Pacific Ocean Biogeographic Information System (OBIS) Node. Dataset/Occurrence. https://nzobisipt.niwa.co.nz/resource?r=caprellidae_borabora&amp;v=1.0 |
| b922024c-ae33-45dd-88fc-70c82d65b365 | NA |
| 5ed811db-baa6-4b24-9109-c48c718681a5 | Wildlife Trusts (2021): Marine Recorder Snapshot extract of surveys entered by Wildlife Trusts. v2.1. Marine Biological Association. Dataset/Samplingevent https://doi.org/10.17031/zwrsjz |
| 81330b73-f70d-4a58-b54f-b9e1f62355d3 | The Danish Biodiversity Information Facility, NivÃ¥ Bay species list, Zealand, Denmark https://doi.org/10.15468/97nj93 |
| 7f29807d-c940-4136-9ccd-3baa1e7e9bab | Finnish Environment Institute SYKE; (2018); Finnish Baltic Sea zooplankton monitoring |
| f32f0bc2-c74e-46af-94b9-815e9dd70c5f | NA |
| 6af80242-edfd-4dd2-b957-8e715789e479 | Lanna, Campos, Bassoi. 2008. South American Antarctic MarineBiodiversity Literature. |
| a48f40d5-b098-4c5a-b001-2f58fe15bb69 | Lopez, A. et. al. (2022): Megabenthos Golfo de Venezuela. v1.6. Caribbean OBIS Node. Dataset/Samplingevent. https://ipt.iobis.org/caribbeanobis/resource?r=megabenthos_golfovzla&amp;v=1.6 |
| 4bbfb75e-7b41-4f8f-b475-051d8c21860a | GarzÃ³n-Ferreira J, Gil D L, RodrÃ­guez A, MejÃ­a N, Rojas J, Navas Camacho R, Bejarano S, Duque G, LÃ³pez M, Herrera O, Posada S, Zapata F, Aguirre A, Bernal C, Manrique N, MuÃ±oz C, Ortiz M, RodrÃ­guez M, Orozco C, Zea S, Olaya J, Vega J, Santodomingo N, GÃ³mez K, BolaÃ±os N, Abril A, GutiÃ©rrez C, GÃ³mez LÃ³pez D I, Torres J, Prato J, Zamora-MartÃ­nez D L (2021): Sistema de Monitoreo de Arrecifes Coralinos - SIMAC: Estructura Coralina. v1.2. Instituto de Investigaciones Marinas y Costeras - Invemar. Dataset/Samplingevent. https://doi.org/10.15472/na9fdj |
| 49f74e10-b23b-4aca-a0f2-62b4f1109dd3 | Species assemblages, biomass and regional habitat characterisation across the offshore Kimberley region. John Keesing, Roland Pitcher, Joanna Strzelecki. WAMSI Kimberley Marine Research Program Final Report Subproject 1.1.1.5 September 2018 |
| 05bde978-ec4c-419b-92cf-94e267e750ad | Elena Arashkevich and Anna Pasternak. Arctic Ocean: Amundsen and Nansen basins July-August 2001. Vertical and stratified zooplankton sampling onboard the Swedish icebreaker Oden, program FAMIZ |
| 2c001849-8854-43dd-889f-7130f9335dce | CSIRO - Southern Surveyor Voyage SS 05/2007, benthic biodiversity of the deep continental shelf and slope in Australia's NW region |
| 7d68eb81-2606-4019-b549-008d633de2b1 | Kachalov, O., Guchmanidze, A.; Ukrainian Scientific Centre of Ecology of the Sea (UkrSCES), National Environmental Agency of the Ministry of Environment Protection and Natural Resources (GE-NEA); (2020): Joint Open Sea Surveys August 2017, Improving Environmental Monitoring in the Black Sea - Phase II (EMBLAS-II), ENPI/2013/313-169, (Macrozoobenthos) |
| 210efc7c-4762-47ee-b4b5-22a0f436ef44 | Silliman K, Anderson S, Thompson L (2023). eDNA from Gulf of Mexico Ecosystems and Carbon Cruise 2021 (GOMECC-4). Version 1.6. United States Geological Survey. Occurrence dataset. https://ipt-obis.gbif.us/resource?r=noaa-aoml-gomecc4&amp;v=1.6 |
| 500c9a4a-30c7-4b85-82f8-ce89b8c27512 | Petrov, A; Milovidova, N. , Alyomov S., Shadrina L. Initial data set (1982-1992) on abundance and biomass of soft-bottom macrozoobenthos , key abiotic variables in near-bottom layers of Sevastopol bay, SW Crimea, Ukraine. Institute of Biology of Southern Seas, Ukraine. |
| 7f5f426e-80bc-4f3b-8d1c-697b086287e8 | See Metadata record for details http://data.aad.gov.au/aadc/metadata/metadata_redirect.cfm?md=AMD/AU/ASAC_2070 |
| 03ff8525-b899-4fd4-8eb4-676308b3aa21 | Amon D (2017): ABYSSLINE UKSRL Contract Area CCZ Megafauna 2013-2015: Arthropoda. v1.2. Deep-sea OBIS node. Dataset/Occurrence. http://ipt.iobis.org/obis-deepsea/resource?r=abyssline_uksrl_contract_area_ccz_megafauna_2013-2015_arthropoda&amp;v=1.2 |
| 2276166e-f74f-48ee-9907-2405d727fbd4 | Campos N H, Lozano Mendoza N A, Correal-CastaÃ±eda M F, GÃ³mez Cubillos C (2023): CaracterizaciÃ³n de los grupos macrofaunales dominantes (crustÃ¡ceos, anÃ©lidos y moluscos), asociada a las comunidades bentÃ³nicas entre los 10 y los 100 m de profundidad en la Isla Cayos de Bajo Nuevo y Ã¡reas aledaÃ±as. v1.1. Universidad Nacional de Colombia. Dataset/Samplingevent. https://doi.org/10.15472/3l1k81 |
| 6c444d85-e355-46a9-939f-6b2dc6ac9def | Interoceanmetal Joint Organization, (2016). IOMPMN12015 Env Template annex 11. Available : DeepData, International Seabed Authority https://data.isa.org.jm/ Accessed: [YYYY-MM-DD]. |
| fcd4f2bf-2352-44e2-8552-2c04848ff30b | Kovalishina, S., Kulakova. I.; Ukrainian Scientific Centre of Ecology of the Sea (UkrSCES), Institute of marine biology, National Academy of Sciences of Ukraine; (2020): National Pilot Monitoring Studies 2016, Improving Environmental Monitoring in the Black Sea - Phase II (EMBLAS-II), ENPI/2013/313-169, (Meiobenthos) |
| 9e3dea3d-6b47-4564-8616-14d54ffc153f | Dorado Roncancio E F (2017): ComposiciÃ³n y abundancia de la comunidad zooplanctÃ³nica presente en la Isla Cayos de Serrana.. v2.1. Instituto de Investigaciones Marinas y Costeras â€“ INVEMAR. Dataset/Samplingevent. http://doi.org/10.15472/ndt8or |
| ec28374c-a11d-4bb9-89a1-7189127f4fd8 | Originator: Rita Horner and David Murphy Publication_Date: 1985 Title:Species Composition and Abundance of Zooplankton in the Nearshore Beaufort Sea in Winter-Spring 1978-1980 Series_Information: Series_Name: Arctic Issue_Identification: 38: 201-209 Other_Citation_Details: NA Online_Linkage: Geospatial_Data_Presentation_Form: document |
| f378dbb6-0513-4a1b-81fb-075a985fef97 | Australian Institute of Marine Science (AIMS). 2009, CReefs Australia - Ningaloo, Western Australia, https://apps.aims.gov.au/metadata/view/7f82b6ac-427c-4b9b-a36c-12799cab6ae6, accessed 16-Jul-2018 |
| 07590136-0a80-4c78-b6b5-ad107fb56d03 | O'Hara T (2023): Sampling the abyss: latitudinal biodiversity patterns along the base of Australia's eastern continental margin - RV Investigator voyage IN2017_V03, Australia (2017). v1.8. CSIRO National Collections and Marine Infrastructure (NCMI) Information and Data Centre (IDC). Dataset/Occurrence. https://www.marine.csiro.au/ipt/resource?r=in2017_v03_abyss&amp;v=1.8 |
| f88d9180-1f1e-414c-b212-5e37f39af8b5 | Albano M J, Rumbold C, Chiesa I L, Vazquez G, Spivak E D, Obenat S M (2021): Occurrence records of the invasive amphipod Monocorophium acherusicum, Mar del Plata, Argentina. v1.0. Southwestern Pacific Ocean Biogeographic Information System (OBIS) Node. Dataset/Occurrence. https://nzobisipt.niwa.co.nz/resource?r=m_acherusicum_argentina&amp;v=1.0 |
| 26866583-f350-4d53-8fc0-c3eabb886aa5 | Finenko, G.; Abolmasova, G.; Datsyk, N.; Anninsky, B.; Romanova, Z. (2012). Mesozooplankton abundance and biomass in Sevastopol Bay and inshore waters off the Crimean Coasts of the Black Sea. Institute of Biology of the Southern Seas NASU. |
| 48beeea5-60d5-4e11-ad3c-754a4a814da9 | Japan Oil, Gas and Metals National Corporation, (2016). JOGMECCRFC12016 Env Template 2016 Edokko data. Available : DeepData, International Seabed Authority https://data.isa.org.jm/ Accessed: [YYYY-MM-DD]. |
| a4c50c8c-e853-45c5-b5b5-30907ec9a8bb | Wolff G, Rowe G, Kennicutt M, Presley B, Bernhard J, Morse J, Montagna P, Nowlin W, Bryant W, Wade T (2023). The Deepwater Program: Northern Gulf of Mexico Continental Slope Habitat and Benthic Ecology - DgoMB: Meios. Version 1.3. United States Geological Survey. Samplingevent dataset. https://ipt-obis.gbif.us/resource?r=dgomb_meio&amp;v=1.3 |
| 56e55f76-6dda-403d-805b-f8922fa1766d | NA |
| e3a1422d-c3f3-41bc-8ff9-fd293d4fbbaa | GarcÃ­a-UrueÃ±a R, Coneo-GÃ³mez S (2021): Fauna crÃ­ptica del Bajo de las Ãnimas, Caribe colombiano. v1.0. Universidad del Magdalena. Dataset/Occurrence. https://doi.org/10.15472/awgfxy |
| f8ef97b4-23f4-4ec7-8158-87179fc8a517 | Steyaert, M. (1994). Meiobenthos at the stations 115, 702, 790 on the Belgian Continental Shelf. Ghent University, Department of Biology, Marine Biology Section (MARBIOL), Belgium |
| 13977fac-0505-4aad-af35-7d1884933d9d | Bessey, Cindy, Jarman, Simon N., Stat, Michael, Rohner, Christopher A., Bunce, Michael, Koziol, Adam, Power, Matthew, Rambahiniarison, Joshua M., Ponzo, Alessandro, Richardson, Anthony J. and Berry, Oliver (2019). DNA metabarcoding assays reveal a diverse prey assemblage for Mobula rays in the Bohol Sea, Philippines. Ecology and Evolution 9 (5) 2459-2474. |
| 380fe998-3ac8-46b1-add1-ec518e043d63 | Kaiser, Stefanie; Brix, Saskia; Brenke, Nils; Brandt, Angelika; Martinez Arbizu, Pedro; MÃ¼hlenhardt-Siegel, Ute; Saaedi, Hanieh; KÃ¼rzel, Karlotta; WÃ¤gele, Johann-Wolfgang (2022) Benthic macrofauna from the abyssal equatorial and South Atlantic collected during the DIVA 1-3 expeditions (2000, 2005, 2009) using an epibenthic sledge. |
| b028c32d-98bc-49dd-b2ae-038d9bff101f | Veit-KÃ¶hler, G. &amp; Laudien, J. (2005). Arctic meiofauna succession. Forschungsinstitut Senckenberg; Deutsches Zentrum fur Marine BiodiversitÃ¤tsforschung (DZMB), Germany. |
| fec85a62-4784-41e3-804f-8f41bd1140b5 | NA |
| 03579385-106b-4df9-9b61-393735989db2 | Friedlander AM, Caselle JE, Ballesteros E, Brown EK, Turchik A, Sala E (2017): The Real Bounty: Marine Biodiversity in the Pitcairn Islands. v1.2. Southwestern Pacific Ocean Biogeographic Information System (OBIS) Node. Dataset/Occurrence. https://nzobisipt.niwa.co.nz/resource?r=pitcairn&amp;v=1.3 |
| e79bb430-0145-4b13-bcf9-89024cfe64ee | Bruneau B, Paille N, NozÃ¨res C, Roy V (2023): DFO Quebec Region Magdalen Islands Lobster Survey. v1.3. Fisheries and Oceans Canada. Dataset/Occurrence. https://ipt.iobis.org/obiscanada/resource?r=dfo_que_idm_ha&amp;v=1.3 |
| 82ffb702-cecf-41dc-9314-649c08051ec1 | Paranjape, Madhu A. and Robert J. Conover. 2013. BioChem: Zooplankton of St. Margarets Bay 1968 to 1971. Version 2 In OBIS Canada Digital Collections. Bedford Institute of Oceanography, Dartmouth, NS, Canada. Published by OBIS, Digital http://www.iobis.org/. Accessed on â€“INSERT DATE |
| cdfb6d8d-14d1-4232-aace-35ba00ff9e12 | CSIRO - Southern Surveyor voyage SS 01/2000, SE large marine domain and the Great Australian Bight |
| e8c2a9de-09d9-4301-a5f6-9af5eaa1ed12 | Polish Monitoring Programme - Monitoring of the Baltic Sea, Poland; (2014), PMS, IMWM Database: zooplankton |
| 50903a57-ee9f-4367-b2cd-0b36dcf4a6ad | SWPRON (2017). Catch data from New Zealand research trawls. Southwestern Pacific OBIS, National Institute of Water and Atmospheric Research (NIWA), Wellington, New Zealand, 15157 records, Online http://nzobisipt.niwa.co.nz/resource.do?r=trawl released on April 19, 2017. |
| 4462e452-1184-44eb-982c-ca2551931ee8 | Fonda-Umani, S.; De Olazabal, A. (2017).Mesozooplankton South Adriatic-PRISMA1-Flussi Project. University of Trieste; Marine Biology Laboratory https://doi.org/10.6092/f8dde52c-cb1f-4e27-908f-c04d43c4e859 |
| f65c4c82-9725-478c-bd46-3d90e64454ef | Mykhailo Nabokin Ukrainian Scientific Centre of Ecology of the Sea (UkrSCES) |
| c9bf012b-0e7b-44fc-b2af-d036da49489f | de Olazabal, A.; Fonda-Umani, S. (2014). Mesozooplankton North Adriatic-Gulf of Trieste LTER time-series. National Institute of Oceanography and Experimental Geophysics (OGS) https://doi.org/10.6092/759d611d-3767-4f5e-8808-553fc54b8931 |
| 0aa9762f-8b38-4515-bd49-f287a4feffd5 | Federal Institute for Geosciences and Natural Resources of Germany, (2017). BGRPMN12017 Env Template MANGAN2016. Available : DeepData, International Seabed Authority https://data.isa.org.jm/ Accessed: [YYYY-MM-DD]. |
| 14242931-d643-4f29-a505-4d8eab59751b | Bluhm, B.A., MacDonald IR, Debenham C, Iken K. (2004) Macro and Megabenthic Communities in the High Arctic Canada Basin: Initial Findings. Polar Biology, 28: 218-231 |
| 5344851d-7639-4949-8d5b-6a6be2035c14 | Li, B., Dickie, P. 2012. BioChem: Bedford Basin Monitoring Program zooplankton collection (1991+). OBIS Canada Digital Collections. OBIS Canada, Bedford Institute of Oceanography, Dartmouth, Nova Scotia, Canada, Version 1, Digital, retrieved from http://iobis.org/. |
| 48b53def-46f8-4e6f-9fae-fb4d90a24fd1 | Federal Institute for Geosciences and Natural Resources of Germany, (2016). BGRPMN12016 Biodiversity Envdata. Available : DeepData, International Seabed Authority https://data.isa.org.jm/ Accessed: [YYYY-MM-DD]. |
| 3f980ed3-99d1-45b8-a8b8-923c8ac8bf82 | Nelson, W.; Cummings, V.; Dâ€™Archino, R.; Halliday, J.; Marriott, P.; Neill, K. (2017): Macroalgae of the Balleny Islands and western Ross Sea, Southern Ocean. v1.1. The National Institute of Water and Atmospheric Research (NIWA). Dataset/Samplingevent. https://nzobisipt.niwa.co.nz/resource?r=balleny_marcoalgae&amp;v=1.1 |
| 059f5fef-917c-450a-9a3f-ed93eddc853f | Franzo A. and Del Negro P. (2020): Meiofauna monitoring in the Trieste harbour, northern Adriatic Sea (Port Authority), 2013 and 2015. OGS (Istituto Nazionale di Oceanografia e di Geofisica Sperimentale), Division of Oceanography. Italy. Dataset/Occurrence. https://doi.org/10.6092/b3rs-4076 |
| 55cdfd04-c597-43c1-9420-c1c2601071a4 | Weitkamp, L., Farley, E., Howard, K., Garcia, S., Murphy, J., &amp; Moss, J. (2022). Trawl Data from the R/V Bell M. Shimada during the 2022 International Year of the Salmon Pan-Pacific Winter High Seas Expedition (Version 1.0) [Data set]. North Pacific Anadromous Fish Commission. https://doi.org/10.21966/NT8W-JE90 |
| 8e1a7146-8eba-4c00-8243-88747d75296c | de Olazabal, A.; Fonda-Umani, S. (2014). Netplankton North Adriatic-ALPE ADRIA Project. University of Trieste; Marine Biology Laboratory https://doi.org/10.6092/57f2fc2f-0315-4399-8510-4d42cd9119e1 |
| 55e369ca-ff48-4024-909f-7a3ce2d74dcc | Ingels J, Brooke S (2022): Meiofauna and Nematode abundance from the West Florida Escarpment, NOAA Hydrosmac project. v1.0. United States Geological Survey. Dataset/Samplingevent. https://www1.usgs.gov/obis-usa/ipt/resource?r=noaa_hydrosmac_fl_meiofauna&amp;v=1.0 |
| 61887f35-b176-466c-87c2-8f8a52bc224a | Roux M, LÃ©vesque D, Scallon-Chouinard P (2023). DFO Quebec Region Fish and invertebrate assemblages in coastal areas of the St. Lawrence Estuary (north shore) sampled with a beam trawl. Version 1.1. Fisheries and Oceans Canada. Samplingevent dataset. https://ipt.iobis.org/obiscanada/resource?r=dl_cha_ppo&amp;v=1.1 |
| efe02bea-8a90-48f2-bb6b-d65141295d1d | Citation_Information:Originator: MANDATORY for FGDC: The originator of the data Publication_Date: Unknown Title: Barents Sea Zooplankton â€“ March and May 1998 and July 1999. Zooplankton sampling on board the Norwegian R/V Jan Mayen, program ALV Online_Linkage: NA |
| 1834801f-a3df-4551-a956-c3ea671e62a6 | Agri-Food and Biosciences Institute (AFBI) (2019): Benthic bycatch from dredge surveys for seed mussel (Mytilus edilus) stock assessment, Outer Ards, Northern Ireland. v3.6. Marine Biological Association. Dataset/Samplingevent https://doi.org/10.17031/2z09sx |
| 35c130fd-b7a6-473d-a6eb-26618c27824b | Van Gaever, S. (2000). Meiobenthos of the Darwin mounds (North-East Atlantic). Ghent University, Department of Biology, Marine Biology Section (MARBIOL), Belgium. |
| 03e33888-23b1-42f2-a0d3-caf759fa858c | Cornthwaite M (2023). DFO Pacific Groundfish Synoptic Trawl Surveys - Queen Charlotte Sound. Version 2.0. Fisheries and Oceans Canada. Samplingevent dataset. https://doi.org/10.25607/uflf4c |
| 043e4751-b140-45e0-af27-2170b708a6bc | NA |
| 2d332d86-1801-4651-8643-706a15f2d5da | NA |
| 574a6f59-d0d6-432d-bb04-bf52c14e3ccd | Criales HernÃ¡ndez M I, Benavides Serrato M, Jerez Guerrero M (2021): Estudio de la comunidad zooplanctÃ³nica y fitoplanctÃ³nica en los ecosistemas de arrecifes coralinos mesofÃ³ticos del Parque Nacional Corales de Profundidad. v1.1. Universidad Industrial de Santander. Dataset/Samplingevent. https://doi.org/10.15472/p70rj0 |
| 65ea0c83-0824-48d4-99ac-6b9f755b11e0 | Currie, D; Sorokin, Shirley J (2011): A preliminary assessment of the deepwater benthic communities of the Great Australian Bight Marine Park. Report to the South Australian Department of Environment and Natural Resources and the Commonwealth Department of Sustainability, Environment, Water, Populations and Communities, SARDI Aquatic Sciences Publication, Adelaide, F2011/000526-1 Access at https://pir.sa.gov.au/research/publications/research_reports/research_reports_2011 |
| dfb911f2-138a-42b4-95d0-37ed041b9490 | NA |
| a4257cff-3c2c-4a60-ab29-91f65545bb08 | Indian Ocean Biodiversity Information System (IndOBIS)- Distribution records of marine organisms from the Indian Ocean. |
| b99fe048-7da1-4558-a118-c98c213f4fde | Franzo A. and Del Negro P. (2020): Meiofauna of the Mar Piccolo of Taranto (Ionian Sea), 2013-2014. OGS (Istituto Nazionale di Oceanografia e di Geofisica Sperimentale), Division of Oceanography. Italy. Dataset/Occurrence. https://nodc.inogs.it/metadata/doidetails?doi=10.6092/cbcda0f7-4599-4fce-ba2c-c337879443ee |
| 2f458cd8-c969-4a36-8f18-b970f80ee3f4 | Belley, R, Boulanger M-P, Roy V, Sean A-S, NozÃ¨res C (2023). DFO Quebec Region Magdalen Islands Sea Scallop Survey. Version 1.0. Fisheries and Oceans Canada. Occurrence dataset. https://ipt.iobis.org/obiscanada/resource?r=dfo_que_idm_pm&amp;v=1.0 |
| c1092532-8c5b-488f-baec-4532c0f25e46 | Hildebrand, L.P. (2016) Distribution, composition and abundance of zooplankton in Chignecto Bay during 1978. Version 1 In OBIS Canada Digital Collections. Bedford Institute of Oceanography, Dartmouth, NS, Canada. Published by OBIS, Digital http://www.iobis.org/. Accessed on â€“INSERT DATE |
| d6d09a68-4349-49d7-9991-474fb75578e0 | Cornthwaite M (2023). DFO Pacific Groundfish Synoptic Trawl Surveys - Hecate Strait. Version 2.0. Fisheries and Oceans Canada. Samplingevent dataset. https://doi.org/10.25607/mzbjdr |
| d19d6c41-ab39-467e-871c-472ae67bbd57 | Estonian Marine Institute, University of Tartu (2019): Zooplankton biomass and abundance in Estonian territorial waters 1994-2016 |
| 0ed9d24d-4826-4b07-b345-7c3e1bea582a | Shiganova, T. Zooplankton from the north-eastern Black Sea for 1994-2005. P.P. Shirshov Institute of Oceanology, Russian Academy of Sciences, Russia. |
| 5ed4177f-5460-45c0-83cf-a98b1c5a8410 | China Ocean Mineral Resources Research and Development Association, (2015). COMRACFRC120015 Env Template 2015 demersal scavenger. Available : DeepData, International Seabed Authority https://data.isa.org.jm/ Accessed: [YYYY-MM-DD]. |
| 5c0e6a1c-adce-40ca-88c5-f1dfb1e650f9 | Interoceanmetal Joint Organization, (2014). IOMPMN12014 Env Bio. Available : DeepData, International Seabed Authority https://data.isa.org.jm/ Accessed: [YYYY-MM-DD]. |
| 99e432a3-9be6-4374-b6d2-91e29d6a5987 | RodrÃ­guez Toscano A J, CedeÃ±o-Posso C M, Montoya-Cadavid E, Borrero Perez G H, Lizarazo RodrÃ­guez N P, Osorno Arango A M, Martinez Campos B, Bustos OrdoÃ±ez H F, Acero Pizarro A, Campos Campos N H, Cantera-Kintz J R, DueÃ±as L F, Polanco FernÃ¡ndez A, Zea Sjorbeg S (2023): CaracterizaciÃ³n de la fauna epibentÃ³nica y demersal de la Reserva Natural Cordillera Beata. v2.3. Instituto de Investigaciones Marinas y Costeras - Invemar. Dataset/Samplingevent. https://doi.org/10.15472/vl4qz2 |
| 2888968e-dc59-4ae4-873b-236b0c7a3e5a | SWPRON (2019): Biological records from the U.S Antarctic Service Expedition, 1939-41. v1.1. Southwestern Pacific Ocean Biogeographic Information System (OBIS) Node. Dataset/Occurrence. https://nzobisipt.niwa.co.nz/resource?r=usase&amp;v=1.1 |
| afd29291-68e3-4c18-8428-958ef71f87ec | Cornthwaite M (2023). DFO Pacific Groundfish Synoptic Trawl Surveys - West Coast Haida Gwaii. Version 2.0. Fisheries and Oceans Canada. Samplingevent dataset. https://doi.org/10.25607/iwvftg |
| 81ace119-09fd-467e-8f6e-74ca29b01634 | King, J. R., Tabata, A., Freshwater, C., &amp; Zubkowski, T. (2022). Trawl Data from the CCGS Sir John Franklin during the 2022 International Year of the Salmon Pan-Pacific Winter High Seas Expedition [Data set]. North Pacific Anadromous Fish Commission. https://doi.org/10.21966/gmrz-ad56 |
| 7ed58608-0db6-4a9b-a8f1-4527d2002214 | China Ocean Mineral Resources Research and Development Association, (2017). COMRACRFC12017 Env Template DY29 zooplankton. Available : DeepData, International Seabed Authority https://data.isa.org.jm/ Accessed: [YYYY-MM-DD]. |
| cef48fb7-f74c-45a6-9871-7c5f346b7729 | Quiroga S, Lagos A M, Leon M V, Colorado A, Giraldo D (2020): CaracterizaciÃ³n de organismos asociados a microplÃ¡sticos en las playas de Santa Marta. v1.1. Universidad del Magdalena. Dataset/Occurrence. https://doi.org/10.15472/gdddsu |
| 1a5aca77-611e-4496-9702-0a938aa27d9c | Danish Centre for Environment and Energy (2019). Zooplankton data for Danish marine monitoring (ODAM) from 1991 - 2011 |
| 287f44a3-d5b7-4efb-8708-809d1a5323ae | Saeedi H &amp; Brandt A (2020): NW Pacific Deep-sea Benthos Biodiversity (Beneficial Project). v1.2. Deep-sea OBIS node. Dataset/Samplingevent. http://ipt.iobis.org/obis-deepsea/resource?r=deep-sea_biodiversity_benthos_beneficial&amp;v=1.2 |
| 2c9890c1-45f9-4ac7-a61a-3ec987659045 | China Ocean Mineral Resources Research and Development Association, (2015). COMRACFRC120015 Env Template 2015 macrofauna. Available : DeepData, International Seabed Authority https://data.isa.org.jm/ Accessed: [YYYY-MM-DD]. |
| 7406cdba-3aa9-4563-a003-64caeec04058 | Svetlana Kovalishina, Ukrainian Scientific Centre of Ecology of the Sea (UkrSCES), Irina Kulakova Institute of marine biology, NAS of Ukraine |
| 556cc02f-da65-4233-85b0-cebc2c3aadfc | Soetaert K.; Heip C.H.R.; Vincx M.; Sektie Mariene Biologie, Laboratorium voor Morfologie en Systematiek der Dieren. RUG: Belgium; (2017): Deep-sea meiobenthos at Calvi (Corsica) sampled in September 1982. (http://www.vliz.be/en/imis?module=dataset&amp;dasid=5483) https://dx.doi.org/10.14284/279 |
| b7735837-9add-4b42-be20-f4d4a2e5bf13 | Persoone G.; Laboratorium voor Oekologie. RUG: Belgium; (2016): The importance of fouling in the port of Ostend in 1964. (http://www.vliz.be/en/imis?module=dataset&amp;dasid=4584) https://dx.doi.org/10.14284/123 |

**Table S2.** Datasets used in this study were extracted from GBIF.

| **dataset_key** | **dataset_title** | **N of records** |
| --- | --- | --- |
| 8821e36f-2d25-44c8-a95e-fb978f38ecde | SER Site-based Surveys | 28 |
| bbee118c-a50e-4f7f-bca1-d5bfefc5360b | SER Species-based Surveys | 86 |
| 48b67072-8da1-4ad1-b187-5eb62acc91fe | Macroinvertebrados asociados a los rios Napi y San Francisco, departamento del Cauca, 2018 | 3 |
| 88967ea9-19ea-4f4d-b137-879ee00ebda8 | CardObs : Observations naturalistes issues de l'outil de saisie et gestion CardObs mis en place par le Service du Patrimoine Naturel (MNHN) / UMS PatriNat (OFB - CNRS - MNHN) - Données naturalistes de CERCOPE (Jean-Louis PRATZ) | 186 |
| e1beb83c-9b83-4d17-ac5e-d24e09507ec5 | BoBO - Botanic Garden and Botanical Museum Berlin Observations | 1 |
| 61b2264c-f762-11e1-a439-00145eb45e9a | Schussenaue bei Berg | 2 |
| 2444d607-5bbc-4b7a-a804-fd9fbaebd6c6 | Efectos del Cambio Ambiental en las comunidades de organismos de los ríos mediterráneos | 126 |
| 427a6290-0c65-11dd-84d2-b8a03c50a862 | Lund Museum of Zoology (MZLU) | 5702 |
| 2ca312ee-40cf-49e0-b864-31531b7eba81 | AM: Freshwater Invertebrates (1900-2005) | 161 |
| 965169e4-f762-11e1-a439-00145eb45e9a | BIOLOGICAL SCIENCES DATA | 1110 |
| 619b7e4c-f762-11e1-a439-00145eb45e9a | Lillachtal mit Kalktuffquelle bei Weißenohe | 1 |
| f2fefe8b-58fa-4b2e-9ddd-756664b5ddd3 | Zoobenthos data from the Southern Beaufort Sea, 1971-1975 | 304 |
| 61939e52-f762-11e1-a439-00145eb45e9a | Triebesbach (Zeulenroda-Triebes) | 1 |
| 7a96ecf6-f762-11e1-a439-00145eb45e9a | Grünes Germersheim | 1 |
| d015c8d2-b847-4007-a401-7c4685d53218 | Explore Your Shore | 15 |
| a4263910-5764-40f4-b375-9f43f4cb757c | Manipulative field experiment to study the predation by epibenthos on infaunal macrobenthos in a Ceriops tagal mangrove at Gazi Bay in 1992 | 39 |
| 6171366e-f762-11e1-a439-00145eb45e9a | Artenvielfalt Kreis Gießen | 1 |
| 637bc9ce-f762-11e1-a439-00145eb45e9a | Natur-Erlebnisgebiet der Naturschutz-Akademie Hessen und Umgebung | 1 |
| f61e69d1-e79f-4ccb-bd92-56a7cefcf1e4 | Deer Creek Riparian Restoration Ecological Monitoring | 77 |
| da209453-8d41-4a29-b759-1f5e1a2b6980 | The Deepwater Program: Northern Gulf of Mexico Continental Slope Habitat and Benthic Ecology - DgoMB: Macros | 272 |
| 0628cc70-37e0-4353-949d-e3990bc2a341 | Benthic amphipods from Alacranes Reef, Campeche Bank, Mexico | 588 |
| 7557f69d-22f8-4cd8-9168-16d60f7170fd | Standing water cartography, Recorder-Lux database | 1 |
| 7a71dbbe-f762-11e1-a439-00145eb45e9a | Bayerische Donau - Riedlingen | 1 |
| 7a75bd56-f762-11e1-a439-00145eb45e9a | Artenvielfalt rund um die Dalbek-Schule | 1 |
| 02f57aa9-12ae-4c8c-9d9f-f8b3245cf31a | Crustacea collection (TSZCr) The Arctic University Museum of Norway | 8289 |
| c48b060b-5473-480a-9790-3cc0e0c10f55 | Biology and Ecology of Cryopelagic Amphipods from Arctic Sea Ice Collected near Franz Josef Land in the summer of 1994 | 11 |
| ab1280cb-11f0-495d-9dd6-f121598eebe2 | Siphonoecetini Just, 1983 (Crustacea, Amphipoda, Ischyroceridae) 11: Cephaloecetes schioettei sp. nov. from The Philippines | 11 |
| 719dc980-f762-11e1-a439-00145eb45e9a | Einen Tag lang Forscher sein - Die 5c der Erich-Kästner-Schule erforscht das Bachemer Wiesental | 1 |
| 0c839b4b-6db2-4d4b-9478-3b213b2e3d4e | COLECCIÓN ECOLÓGICA DEL IBN (CONICET-UNT) PROYECTO PUNA | 192 |
| 62a4f78c-f762-11e1-a439-00145eb45e9a | Weide an der Mosselde / Dortmund-Kirchlinde/Westerfilde | 1 |
| 7910cc7c-8483-4551-83b1-a2cd2709a317 | Megabenthos biodiversity of the northwest coast of Paraguana Peninsula (Venezuela) | 5 |
| 627bd474-f762-11e1-a439-00145eb45e9a | Selz (Ingelheim am Rhein) | 1 |
| 31c8db7d-098d-4339-a77b-901865139f1c | DFO Zoobenthos data from upper Frobisher Bay, 1967 to 1973 | 398 |
| 7066ae08-1550-4071-8058-168456c427f6 | Maritimes 4VSW Research Vessel Surveys | 11 |
| 89e8b7d4-f762-11e1-a439-00145eb45e9a | Naturschutzgebiet Heiliger Hain (Wahrenholz) | 2 |
| 625c474e-f762-11e1-a439-00145eb45e9a | Bäche, Quellen und Teiche im FFH-Gebiet Mühlhauser Halde | 1 |
| 9649d058-f762-11e1-a439-00145eb45e9a | Tag der Artenvielfalt im Taubental | 1 |
| 8928e328-f762-11e1-a439-00145eb45e9a | Macrozooplankton concentrations estimated from MOCNESS tow samples, Continental Margin Western Antarctic Peninsula, GLOBEC | 57 |
| 89c8f44e-f762-11e1-a439-00145eb45e9a | Artenvielfalt der Nordsee - Helgoland | 9 |
| 89bba0c8-f762-11e1-a439-00145eb45e9a | NSG Karwendel | 1 |
| 3027f82b-ca59-4d44-a11d-51bde63fb8db | Diversity of meiobenthos in tropical seagrass beds of Gazi Bay (Kenya) sampled from 16 to 19 July 1996 | 14 |
| 95991682-f762-11e1-a439-00145eb45e9a | NSG Dellwiger Wald, Dortmund | 1 |
| 9543fe6c-5a30-454e-bc1d-0ee9c186fd79 | Study of epifauna and meiobenthos by means of cage experiments in a Ceriops tagal and Avicennia marina mangrove at Gazi Bay (Kenya) in August and September 1992 | 12 |
| 9589146c-f762-11e1-a439-00145eb45e9a | Perchtoldsdorfer Heide | 2 |
| 08afaadf-34ba-4aeb-be23-027da1e38676 | Records of protected animal, plant and fungi species in Ukraine | 6 |
| b2257a13-0e1f-4899-98ea-aacb812509aa | Mesozooplankton in the South Adriatic Sea (MEDGES Experiment) in March 2013 | 2 |
| 7a945eaa-f762-11e1-a439-00145eb45e9a | Grundschule Sandheide | 1 |
| 30471991-67e4-495a-bd59-a7c936bc2e66 | The family Izinkalidae fam. nov. (Crustacea: Amphipoda: Lysianassoidea) in Australian waters | 2 |
| edeee57f-9e48-4202-b387-9cb5224e7b1b | JODC Dataset | 1463 |
| c6ad8415-263a-4674-81fc-f590bcaaaea0 | FBIP:Invertebrates community dynamics in temporary wetland ecosystems | 3 |
| 0cfae73c-cef7-41e7-9e41-b41cb29be7c2 | Argentina–Chile National Geographic Pristine Seas Expedition To The Antarctic Peninsula - Deep Sea Cam Data | 16 |
| f0c3a31b-df3b-4670-ac1d-0c58d13e1420 | Marine Invertebrate from Argentina, Uruguay and Chile | 104 |
| 87f42a91-8dfd-44f1-aabb-8b6ef14aba82 | Canadian Museum of Nature Crustacea Collection | 15478 |
| 3c428404-893c-44da-bb4a-6c19d8fb676a | Alien macroinvertebrates in Flanders, Belgium | 1214 |
| e2cde7e3-0b90-40ea-93ad-de06e6ed3a3c | Benthos Chirnov Basin 1986 and 2002 | 671 |
| fd61e581-4db6-4fa8-8c27-79e118459f42 | Structure and function of contemporary food webs on Arctic shelves: A panarctic comparison. The pelagic system of the Kara Sea- communities and components of carbon flow | 88 |
| 5aa8c4ed-3df2-4179-8e6a-16222f2dbe1c | Levantamientos portuarios de fitoplancton, zooplancton, perifiton y macrozoobentos en el Pacífico colombiano | 27 |
| 61c88f72-f762-11e1-a439-00145eb45e9a | Erft in Selikum (Neuss) | 1 |
| 968dbe0f-a8a0-498f-816a-cb5b4b5c041b | Species Composition and Abundance of Zooplankton in the Nearshore Beaufort Sea in Winter-Spring 1978-1980 | 15 |
| 4aeef7f2-a35b-405e-bcc6-7024a251ddab | Structures and Nutrition Requirements of Macrozoobenthic Communities in the area of the Lomonossov Ridge, 1995-1998 | 85 |
| 97171b6d-e543-45f5-bc87-41e5090d3a69 | Registro de macrofauna bentónicas submareales de fondos blandos, variables físicas y químicos en la zona sur de Chile - Registro 2 | 19 |
| c988d44d-e992-4653-be46-124cefa0d314 | Zooplankton Abundancies White Sea, 1972 | 17 |
| 89a92395-88a2-4904-962f-20184a06b383 | Registro de macrofauna bentónicas submareales de fondos blandos, variables físicas y químicos en la zona sur – Registro 3 | 21 |
| 1d1f4137-7653-4eac-ab67-7487dae51739 | ZooplanktonBeaufortSeaNOGAP2 | 808 |
| 8927ad78-f762-11e1-a439-00145eb45e9a | Zooplankton abundance and population structures assessed during April-September of 2001-2, Southern Ocean, GLOBEC | 143 |
| 40ee219d-ab5f-4917-9c7a-6137dba77b05 | BenthosChukchiFN762_1976_Falk5 | 29 |
| 9cdfd6cb-b1b0-4534-bdb0-a50a0e68076a | Under-ice Amphipods in the Greenland Sea and Fram Strait (Arctic): Environmental Controls and Seasonal Patterns Below the Pack Ice | 66 |
| 8ec254cc-7da8-4055-b3f0-ded781a43522 | Chucki_Seaooplankton1953/4 | 12 |
| e87e14f1-76e9-48a1-8378-98cc909eb301 | WTSWW Data: All Taxa (West Wales) | 10 |
| 2e844554-d9bf-4540-8c6d-2999ad5ced21 | IMOS National Reference Station (NRS) - Zooplankton Abundance | 86 |
| 52855660-ce87-4312-9538-fefde37134b6 | ZooplanktonBeaufortSeaNOGAP1 | 866 |
| d888b684-3c55-44cd-b7d1-253d8cad1ca7 | Zooplankton Bering Strait Tiglax 1991 | 39 |
| 9bfd4499-050e-41b7-93a8-72d9e2490e21 | Macrozoobenthos composition, abundance and biomass in the Arctic Ocean along a transect between Svalbard and the Makarov Basin 1991 | 71 |
| 177d1017-a295-481e-9b02-27391288c5e0 | Seasonal dynamics of sub-ice fauna below pack ice in the Arctic (Fram Strait) | 39 |
| 11e43d34-3a92-4ed0-b735-6cb56a3169ca | Laptev Sea and Nansen Basin Zooplankton, 1993 | 120 |
| 7b579758-f762-11e1-a439-00145eb45e9a | Antarctic Isopods | 45 |
| 7b67c51a-f762-11e1-a439-00145eb45e9a | South American Antarctic Marine Biodiversity Literature | 27 |
| 4b07e08f-3be0-4a5c-ae7d-e450e6a7a1fb | Barents Sea Zooplankton – March and May 1998 and July 1999. Zooplankton sampling on board the Norwegian R/V Jan Mayen, program ALV | 8 |
| 0a1b70f1-ea1e-405d-aa4e-4142888bd8d3 | Barents Sea Zooplankton – March and May 1998 and July 1999. Day/night stratified zooplankton sampling onboard the Norwegian R/V Jan Mayen. Program ALV | 27 |
| 6a31497b-234c-4d24-a3aa-e56d8655f57b | CASES2003_2004 | 32 |
| b394cf35-d6d1-4d9d-9d5b-693229207b02 | Records of macrozoobenthos organisms in several water bodies of Ukraine during 2007-2021 | 50 |
| 78fb84f9-b5e1-4067-a1b1-730aca8c1bdc | Greenland Macrobenthos Young Sound, 1996 | 10 |
| ddf006f2-5c76-47d5-9180-4a52d8d5d4e0 | Greenland macrobenthos 2006 | 48 |
| 240f38f1-ada4-4eac-a9b5-28dd8308d01d | Maritimes Spring Research Vessel Surveys | 7 |
| 310dbb60-e369-4ec2-a6d8-8e4de843b208 | Pacific Multispecies Small Mesh Bottom Trawl Survey | 29 |
| 78c508c2-f762-11e1-a439-00145eb45e9a | Matter enrichment of the macrofauna community of MAC station 13200-005 | 16 |
| 3c3020b4-6113-43db-b702-94b76aa5ed71 | NW pacific deep-sea benthos distribution and abundance (Beneficial Project) | 469 |
| 8359171b-9432-421e-88fc-56a55f1c098c | Invertebrate Site Register - England (1738-2005) | 178 |
| 73393ec0-632e-4878-8077-76426fbeb9f1 | Gloucestershire Historic Wildlife Sightings prior to 1st Jan 2000 | 42 |
| 2a54253f-401a-4c0c-8abf-bd9b5cdc8011 | Invasive Non-Native Species | 250 |
| 2370f519-6f22-4b1c-a780-1c90a1c7250b | To the origin of Lake Baikal endemic gammarid radiations, with description of two new Eulimnogammarus spp. | 2 |
| 0284b82d-cdd1-4d67-83bd-c8e42e988adb | Merseyside BioBank (unverified) | 303 |
| 449ae494-44ef-4dc4-b0c8-c1d25ab13c92 | Inventaires de la faune exotique envahissante - Sciences participatives sur les espèces animales introduites (EEE-FIF) | 1 |
| e36c72ed-3b15-49f8-97fc-faaa467eb59e | NRW Regional Data: South East Wales Non-sensitive species | 356 |
| 7b9545bc-f762-11e1-a439-00145eb45e9a | Antarctic Amphipoda | 143 |
| 7004324e-f762-11e1-a439-00145eb45e9a | Meiofauna abundances at the Arctic Håkon Mosby Mud Volcano (HMMV) of sediment core PS64/357-1 | 5 |
| 6fa9bfa8-f762-11e1-a439-00145eb45e9a | Distribution of meiobenthos in sediment core M19_229-2 of the northeastern Atlantic (Table 1) | 4 |
| a74db578-ac84-4907-ab8f-8de6eaa7df56 | Marine metagenomes Metagenome | 1 |
| 50361be0-d9cd-11de-b793-b8a03c50a862 | University of Amsterdam (NL) – Benthos monitoring of the North Sea research database | 4168 |
| 3c1a56f8-b63a-492f-bb77-babb497204fb | Marine Biological Sample Database, JAMSTEC | 1236 |
| 7ec20c70-32e9-4548-86f8-3c915bffefdf | Plankton&BenthosResearch | 86 |
| 25f99a40-2327-4c57-9631-1a0825d834fa | Asia-Pacific Dataset | 327 |
| 049fe945-6ef0-445a-bd88-f7337af1bb90 | Universidad de San Carlos de Guatemala - Colección de Crustáceos | 14 |
| 2e4cc37b-302e-4f1b-bbbb-1f674ff90e14 | Biofokus | 262 |
| 5458db14-d1c8-45ac-9bf3-8cc027df3c94 | Colección de Plancton Mixto – Sección Zooplancton del Museo de Historia Natural Marina de Colombia – Makuriwa (Invemar) | 274 |
| f06ed729-104f-4e51-9db3-dfd9b228a1be | Vascular plant field notes, NTNU University Museum | 1 |
| 988007b1-1179-42e0-863c-bb9f1bdfa6f7 | Pisces (Luomus) | 1 |
| 6c7d1ea7-6586-46f9-a3b4-714f249cd838 | New and little known species of Lepechinella (Crustacea, Amphipoda, Lepechinellidae) and an allied new genus Lepesubchela from the North Atlantic | 4 |
| c8ba2098-74e5-4379-82dc-e83804b59e3f | Alabama Museum of Natural History Paleontology specimens (Arctos) | 13 |
| ef6e0754-8bf2-4854-9aa3-3a2a1a90810b | NIEK_NES | 793 |
| 929eb10b-732f-4247-b899-4e3e5681cb4b | The Macrobenthos of Penobscot Bay, Maine | 85 |
| 0001480b-76ca-4f30-86bc-f4292481554b | Bibliographie de la faune, la flore et la fonge de France métropolitaine et outre-mer - Bulletin mensuel de la société linnéenne de Lyon [1932- ] | 5 |
| aab0cf80-0c64-11dd-84d1-b8a03c50a862 | Lund Botanical Museum (LD) | 1 |
| 636d3274-f762-11e1-a439-00145eb45e9a | Tag der Artenvielfalt am Bruckenwasen | 1 |
| 1cfd5ba9-621f-4abd-97bc-20beb462e2fd | New genera, species and records of Maeridae from Australian Waters: Austromaera, Ceradocus, Glossomaera, Hamimaera, Huonella gen. nov., Linguimaera and Maeraceterus gen. nov. (Crustacea: Amphipoda) | 181 |
| 60c45f15-135d-42fb-8b8e-dd11c6375ad8 | Censo de biodiversidad marina Edo. Miranda | 1 |
| 6ac3f774-d9fb-4796-b3e9-92bf6c81c084 | naturgucker | 518 |
| a6d84a4e-d2c1-4cf1-924f-58d67f592a32 | Survey data of tidal flats on the Monitoring sites 1000 project, BDCJ | 156 |
| 38b4c89f-584c-41bb-bd8f-cd1def33e92f | Artportalen (Swedish Species Observation System) | 4219 |
| 84a649ce-ff81-420d-9c41-aa1de59e3766 | ALA species sightings and OzAtlas | 6 |
| 6201977c-8904-4e14-842f-734f8fc4a137 | Etat initial de la rivière Saucats avant travaux et aménagements pour la restauration de la continuité écologique 2018 - Inventaire macroinvertébrés | 31 |
| b61823f1-58a8-4e7b-a3e8-0c37de8923e7 | A new species of the Hyalella ‘ azteca’ complex (Crustacea: Amphipoda: Hyalellidae) from Florida | 3 |
| 99006141-bf02-4821-8e6e-5818a1cbf70a | imr_mareano_beamtrawl | 2084 |
| e7f1c38e-2a85-4c6e-80aa-d052766a916f | imr_mareano_rpsledge | 9147 |
| d30a617e-cac3-470c-a070-601376f45d6b | imr_mareano_grab | 4276 |
| 7a52c058-f762-11e1-a439-00145eb45e9a | Crustacea Collection of Natural History Museum and Institute, Chiba | 4 |
| 081facf0-b768-41ad-b692-3b30a1bcbb1b | International Polar Year and Census of Antarctic Marine Life Ross Sea voyage (TAN0802) biodiversity data | 123 |
| 2f5fa57d-1fae-444c-9669-258b7fb918b4 | Carnarvon Basin Wetland Invertebrate Survey | 18 |
| 32ba1b9a-b06d-417e-98ea-eb0cfb67466a | Benthic Macroinvertebrate Diversity in the middle Doce river basin, Brazil | 18 |
| 821cc27a-e3bb-4bc5-ac34-89ada245069d | NMNH Extant Specimen Records (USNM, US) | 37932 |
| bbb81807-fc4c-4e4e-bc1c-2caee84a4a06 | Savu Sea-Indonesia eDNA Dataset | 1 |
| 13b1bb11-25d4-4a7d-8e42-7b7c761e2c21 | Juday Net Zooplankton Data from the 2020 Gulf of Alaska International Year of the Salmon Expedition | 43 |
| 2429f28c-dc43-40e7-92f6-49206e0729fb | FBIP:IZIKO-UCT:Historical Survey (1930-1980) | 75 |
| 926b493e-d5c4-4dd8-a2c3-9853ea92aad8 | A new genus and species of Ingolfiellidae (Crustacea, Ingolfiellida) from the hyporheic zone in the Sierra de la Ventana, and its biogeographic relevance | 1 |
| 36ae4600-17b1-437c-bdc5-7a3c98b1bff4 | Rhodolith Beds in Northern New Zealand: Characterisation of Associated Biodiversity | 74 |
| bd87a8e3-8408-4df2-a7a7-f5f405620ab8 | Ohio Wesleyan University Invertebrate Specimens (Arctos) | 2 |
| 3fab912e-e927-4f1c-a97c-eb446cd609e0 | ClimateWatch | 18 |
| 50804764-9c66-4448-8537-6767167e83c5 | NRW Regional Data: all taxa (excluding sensitive species), West Wales | 15 |
| d415c253-4d61-4459-9d25-4015b9084fb0 | The New York Botanical Garden Herbarium (NY) | 1 |
| f0e953e6-155c-4999-b814-7193939340eb | A new species of genus Parhyale Stebbing, 1897 (Crustacea, Amphipoda, Hyalidae) from Gujarat State, India | 4 |
| 3236cdc4-2f4d-4bd6-b53f-54fd8d8a0aa8 | Midden-Limburg Fish Ponds Survey | 10 |
| e0aff3c8-3e32-412b-888c-fde75c2e8750 | BioFresh Pond Data | 50 |
| 2dd78289-9a8e-4f20-8c87-1e572a2bf051 | Additional records of Elasmopus vachoni Mateus & Mateus, 1966 (Crustacea: Amphipoda: Maeridae) from European waters (Tarifa, southern Spain) | 1 |
| dcc54c42-6834-4a88-94b0-597505d28cee | Two new species of amphipods (Crustacea: Amphipoda: Photidae) from Korean waters with a redescription of Gammaropsis longipropodi | 3 |
| 84b72ee4-f762-11e1-a439-00145eb45e9a | Arctic Ocean Diversity | 11924 |
| 7ebef267-9d72-4c21-a276-cc84281a8590 | NatureMapr | 1 |
| 5a474c62-9f7c-465c-8df1-3dddda11bb1f | Counts of hyperbenthos of Gazi Bay (Kenya) sampled in October 1994 | 24 |
| ae3b4cbe-473f-4f90-be9e-c2d330a9843a | Macrobenthos monitoring in the Trieste harbour, North Adriatic Sea (Port Authority) in June 2013 and March 2015 | 36 |
| 2cda8cb6-fde6-4cb1-8301-1c91bb3c2889 | A new species of Victoriopisa bantenensis (Crustacea: Amphipoda: Eriopisidae) from West Java, Indonesia | 1 |
| 67c54f85-7910-4cbf-8de4-6f0b136a0e34 | Continuous Plankton Recorder Dataset (SAHFOS) | 1046 |
| b124e1e0-4755-430f-9eab-894f25a9b59c | Norwegian Species Observation Service | 539 |
| df12ca07-f133-4550-ab3b-fde13f0e76ba | Lajitietokeskus/FinBIF - Notebook, general observations | 35 |
| b2f2cfc7-bd72-403b-9a58-8d0bcdc6e1d7 | Coleção de Crustacea do Museu Nacional (MNRJ - CARCINO) | 3018 |
| 223f6e89-c7b5-4e8c-99a2-da2752f16435 | North Marine Region demersal fish and invertebrate collection records (DEW collation), Australia (1980 - 1997) | 2 |
| f2ab6184-eff7-4069-a9bb-e4f366aeefff | British Antarctic (Terra Nova) Expedition, 1910-1913 | 473 |
| 6f6b1102-5baa-4e24-86be-073a90b14db0 | 1915-2016 Department for Environment Food & Rural Affairs (Defra), Marine Strategy Framework Directive (MSFD) Collation of invasive non-indigenous species | 720 |
| 1c736f5f-18d4-40d3-b25a-ec9d3c3500c7 | Argyll Biological Records Dataset | 28 |
| da23c0cd-312a-46c2-9da6-c29f21b4bf79 | Marine Data from The Wildlife Trusts (TWT) Dive Team; 2014-2018 | 5 |
| dfddad59-5bc5-4e35-8b35-334eed43bba9 | Freshwater samples in MZNA-INV-FRW: Macroinvertebrate samples from the water quality monitoring network along the Ebro Basin | 5328 |
| 1758af10-9070-4772-a2b5-40ca85612db3 | Ensigeropus, a new genus of the family Platyischnopidae (Amphipoda: Amphilochidea) from Northern Chile | 8 |
| 7ec77cf4-cc93-4bdc-b7d7-f3a50a4c84ee | Inventaire de la Réserve naturelle de l'Etang noir - Données de présence récoltées | 3 |
| e672066e-0d00-41ec-bd55-5375ae88acf4 | Monitoreo complementarios de algas, invertebrados y peces en el Parque Nacional Arrecifes de Cozumel | 9 |
| 7b702b2e-f762-11e1-a439-00145eb45e9a | RMT Trawl catch from the 1983/84 V5 ADBEX II voyage | 17 |
| 3186cd3f-a7d0-4e64-bb60-630e20a47fa6 | Monitoring data from the federal state Bavaria (Germany) | 990 |
| e12cbcf7-b081-434f-9532-d93ef76cdb44 | A national macroinvertebrate dataset collected for the biomonitoring of Ireland’s river network, 2007–2018 (EPA) | 9620 |
| 90d11e31-c18a-49b2-bf29-cb86c48b90d3 | AMI-KMNP dataset: records of aquatic macroinvertebrates from SE Hungary | 629 |
| 2a636fd6-dc73-49c0-9e5b-26bd05532313 | New Zealand fish and squid distributions from research bottom trawls 1964-2008 | 9 |
| 02d1e772-54ee-4767-b4b8-c35f0c7270ba | NBIS Records to December 2016 | 1287 |
| 24baf0da-60a9-4a13-99d7-a8c860c3cb30 | Azorean Biodiversity Portal | 1341 |
| 7ecd1a7d-e63c-4307-86dd-ec41b575589f | Réalisation du dossier d’avant-projet pour l’extension de la Réserve Naturelle Nationale des Marais d’Yves (17) - Inventaires endofaune benthique | 5 |
| 6bae1725-5a4d-4e69-8ac2-968a7f091124 | Marine invertebrates registered on the Nansen Legacy JC3 Winter Gaps cruise 19th February - 11th March 2022 | 4 |
| 5e94bf18-506b-4484-bbf4-59c29bcba6c7 | DFO Quebec Region Biodiversity of the snow crab trawl survey in the Lower North Shore (2018) | 31 |
| be8bd010-baa3-4d7a-89a2-537c38aa3c85 | DFO Quebec Region Biodiversity of the snow crab trawl survey in the St. Lawrence Estuary (2019) | 85 |
| bed78790-cbec-44af-82af-fe78e9692287 | Environmental Monitoring database (MOD) DNV | 183993 |
| 6ca76c0f-be62-4db2-9bdf-1e13ffd6b0d2 | DFO Quebec Region Magdalen Islands Lobster Survey | 6 |
| 85b0b9ce-1fac-46a6-ac25-c4ea3645b7af | artsprosjektet_46-15_noramph | 561 |
| 4e8df027-a8e6-4d7a-b576-0c5a83b66403 | DFO Quebec Region Baie Sainte-Marguerite Snow Crab Trawl Survey 2006-2009 | 20 |
| 056ddd7e-bec5-448d-8463-94dc359b81f4 | DFO Quebec Region Biodiversity of the whelk (Buccinum) dredge survey in the St. Lawrence Estuary | 119 |
| f134aac2-9737-46da-a6d7-d4f106881a61 | An experimental approach to study epi-and infauna of mangrove forests in Gazi Bay (Kenya) between August 1992 and July 1993 | 94 |
| a4016504-4522-4bd3-85db-89df1ebce440 | New discoveries for the subfamily Phtisicinae Vassilenko, 1968 (Crustacea: Senticaudata) from the Brazilian coast | 9 |
| eaa86a97-f30f-4240-979c-7160052e9327 | Monitoreos de los ecosistemas marinos de la bahía Portete, Puerto Bolívar, La Guajira | 371 |
| c4db5fd0-e2b6-4737-995e-d895bc596d86 | Morphotaxa Occurrence Data on Schulz Bank 2017-2018, Arctic Mid-Ocean Ridge | 50 |
| 505cabc3-7550-4fd1-96dc-6d77d5ffc6de | Monitoring data from the federal state Hesse (Germany) | 3010 |
| 9575ab07-e816-4cd6-aa99-483343a825e6 | bioman_belgium | 12 |
| ca8c4575-a763-4271-9376-c962981d402a | CardObs : Observations naturalistes issues de l'outil de saisie et gestion CardObs mis en place par le Service du Patrimoine Naturel (MNHN) / UMS PatriNat (OFB - CNRS - MNHN) - Données naturalistes de Jean-Michel LEMAIRE | 1 |
| 4a10326b-603b-429a-8b6a-81e4897b0e33 | A new species of the genus Liljeborgia Spence Bate, 1862 (Crustacea: Amphipoda: Liljeborgiidae) associated with the burrows of the spoon worm Urechis unicinctus in the Sea of Japan | 6 |
| 3924362e-fa83-4a62-a5e2-6f764c872555 | Zooplankton Abundance_Umbrella net_sea ice zone 2007/12 | 2 |
| f74dfed8-d09b-4b18-8443-c20754410e16 | Zooplankton abundance in the Kerguelen Axis region 2016 | 81 |
| 791165a0-f762-11e1-a439-00145eb45e9a | Benthic macrofauna abundance of sediment core 13201-002 | 10 |
| 7912ea9c-f762-11e1-a439-00145eb45e9a | Benthic macrofauna abundance of subcores of sediment core 12930-065 | 52 |
| 79099c3a-f762-11e1-a439-00145eb45e9a | Benthic macrofauna abundance of sediment core 13200-073 | 10 |
| 79027cfc-f762-11e1-a439-00145eb45e9a | Benthic macrofauna abundance of sediment core 13200-025 | 10 |
| 97cf5eac-f762-11e1-a439-00145eb45e9a | Early succession in benthic hard bottom communities in Kongsfjorden, Svalbard - abundance | 308 |
| eacb9186-a68f-4f0a-8c32-94e6eb35e194 | Programme Ecoscope: données d'observations des écosystèmes marins exploités | 30 |
| dc780d9c-9d52-43e0-af26-98ab6a1c3eb3 | Species lists for benthic communities of Norwegian fjords from environmental surveys (data used in Sen et al. Estuarine, Coastal and Shelf Science 2022) | 207 |
| fd7d3a3d-7f94-495f-9df6-842d12e2e4b7 | KiEco Freshwater Ecology: River Macroinvertebrates | 16820 |
| 5d2f6092-6dcd-496b-abba-6d9e95ccc801 | Monitoring data from the federal state Rhineland-Palatinate (Germany) | 787 |
| 75a0a75e-94c4-4c0f-a60d-fb424a9080d5 | Mesopelagic Community off the North Western Portuguese Coast between 1998 and 2000 | 41 |
| 4bdc7171-c006-41b8-b3bf-e6f5227206b3 | MACROBENTHOS COMPOSITION FROM THE RIO LAGARTOS HYPERHALINE COASTAL LAGOON SYSTEM, YUCATAN, MEXICO | 131 |
| e697326b-48d3-44ee-8b8d-0c9ee26c16d7 | Meroplankton in Isfjorden 2016 to 2017 | 31 |
| ec9650f2-c3b7-4a0d-ae1a-24c3433a2539 | Two new species of Hyalella (Crustacea, Amphipoda, Hyalellidae) from state of Rio Grande do Sul, Southern Brazil | 2 |
| 5bf10f28-f0b1-40de-9df6-ac9d8ba22480 | Megafauna of the UKSRL exploration contract area and eastern Clarion-Clipperton Zone in the Pacific Ocean: Annelida, Arthropoda, Bryozoa, Chordata, Ctenophora, Mollusca | 3 |
| 5e8e721a-f830-47a5-ac25-5371f50a7e48 | Suivi et inventaire de la réserve naturelle de Saucats - La Brède - Inventaire et suivi Entomologiques de 2012-2013 | 19 |
| 97914720-f762-11e1-a439-00145eb45e9a | Benthic meiofauna abundance in surface sediment of core PLG93_BCORE3 | 7 |
| 30ae587e-eb14-4bd4-868d-61fb59885b12 | Spatial distribution in sediment characteristics and benthic activity on the northwestern Black Sea shelf: macrobenthos | 33 |
| 71b44837-09a8-481b-a5d8-a4ff5ab966ac | DONNÉES NATURALISTES ATBI MERCANTOUR HORS PÉRIMÈTRE PARC | 7 |
| 66f6192f-6cc0-45fd-a2d1-e76f5ae3eab2 | Diveboard - Scuba diving citizen science observations | 12 |
| 035941f6-46af-457b-9c23-bfb21ffd0904 | Inventaire des invertébrés marins benthiques et des algues de la Guadeloupe : expédition Karubenthos 2012 - Expédition Karubenthos 2012 : inventaire des Mollusques et des Crustacés de la Guadeloupe | 114 |
| fbaa63e3-945b-43d9-920b-812215a5bb1f | Southern Maine Community College Gulf of Maine Invertebrate Data | 48 |
| 6225b5f8-f762-11e1-a439-00145eb45e9a | Binsenwiesen | 1 |
| 6686c610-9d99-459e-b229-fdf3ac35a197 | Crustaceos del Museo de Ciencias naturales de la Universidad Simón Bolívar | 7359 |
| 51c51934-43f1-467c-b1b5-2e1c48fdd51b | CSIRO, Cruise SS200510, Benthic Biodiversity, Western Australia, 2005 | 27 |
| 245e011d-184e-4220-9a47-aafbba8c81f5 | Macrobenthos North Adriatic-ALPE ADRIA Project | 152 |
| eccf3cd7-d6cf-4a4e-a7ee-11fb4e9c1106 | Macroinvertebrados acuáticos asociados a proyectos de Ganadería Sostenible en Colombia (GCS) | 30 |
| 2887d5c4-23c3-4849-8a47-8f52291f30cd | Macroinvertebrados acuáticos presente en vertientes del Río Cali | 2 |
| 1bec5de3-758c-4ed2-ab13-1597601ad07a | Terrestrial and limnic invertebrates systematic collection, NTNU University Museum | 253 |
| 054cfdf9-ccff-4eb3-a815-ee934cf11e04 | Cryptobiont assemblages's dataset of coral reefs using ARMS in B10 Reef and Mahahual Reef of the Great Caribbean | 147 |
| d1009903-09f3-477a-ab2a-a102a658e390 | Meiobenthos North Adriatic-INTERREG-FVG-Projects | 49 |
| d3aa0bbe-b7f7-47fc-bd82-952c7cb24355 | University of Alberta Freshwater Invertebrate Collection (UAFIC) | 86 |
| b5192815-5e90-45b4-a17d-62856a3ead51 | Monitoring data from the federal state North Rhine-Westphalia (Germany) | 4826 |
| 628bb48f-a435-466b-b6c5-e328a3909cab | Lepidepecreoides stoddartae sp. nov. from the Falkland Islands (Amphipoda: Tryphosidae) | 8 |
| 62438092-f762-11e1-a439-00145eb45e9a | Biosphäre Bliesgau | 1 |
| 0d862dd2-12af-4445-92c9-d81d76598b19 | Macroalgae of the Balleny Islands and western Ross Sea, Southern Ocean | 6 |
| e20d61a9-4ba1-41d6-9256-722e8c57cb34 | Benthic invertebrate surveys conducted between 2009-2011 as part of the Sydney Tar Ponds Cleanup and Coke Ovens Remediation Project. | 90 |
| 83b1eeda-f762-11e1-a439-00145eb45e9a | BioChem: Davis Strait and Baffin Bay Zooplankton | 787 |
| 5e34ffdf-2648-4ecf-ab53-555e3d3d8d2b | Monitoring data from the federal state Mecklenburg-Western Pomerania (Germany) | 1020 |
| 9a73f108-5ee8-4e9b-a1f7-8f2c5f0dc0d6 | Monitoring data from the federal state Berlin (Germany) | 113 |
| 625d777c-f762-11e1-a439-00145eb45e9a | Landschaftspark St.Leonhard-Deisendorf | 1 |
| e35506ee-7213-4909-9062-3c4e0f5851cf | Anfípodos_cimar22 | 1082 |
| 28120c52-2b34-4db4-b348-812ee1eaf958 | Stable isotope ratios and elemental contents of C, N and S in benthic organisms sampled during the PROTEKER 5 summer campaign in Kerguelen Islands (2016) - data | 10 |
| cf8ba399-e93a-455e-9ed4-6fbb849e549d | Macroinvertebrados asociados a la ciénega Caño Negro, Medio Baudó, Chocó. 2021 | 4 |
| 997fd207-009e-4387-a4ed-967a01637a72 | Kenai National Wildlife Refuge, Alaska (KNWR) Invertebrate specimens (Arctos) | 1 |
| fc8ca55e-75cf-4c1e-be46-66a4d7119b1b | New records of Nuuanuidae (Crustacea: Amphipoda: Senticaudata) from Korean waters, with descriptions of one new genus and two new species | 2 |
| 29ce88ff-5cf1-47fd-af44-8477c52c910c | Isle of Man historical wildlife records 1990 to 1994 | 14 |
| 92c9ae64-2267-4747-98d4-48dc7ae459be | Juday Net Zooplankton Data from the 2019 Gulf of Alaska International Year of the Salmon Expedition | 60 |
| 312bb844-4980-4b1a-a64b-e79f9ac083a4 | Manscape | 19 |
| 42221846-d81b-4602-8895-869d5593783a | Trawl Data from the R/V Bell M. Shimada during the 2022 International Year of the Salmon Pan-Pacific Winter High Seas Expedition | 1 |
| 76df66d1-8755-4381-b02d-865748aa2561 | Caracterización de organismos asociados a microplásticos en las playas de Santa Marta | 1 |
| f9d0e547-ce5a-4814-be05-19540309c2df | Minute but constant morphological differences within members of Stenothoidae: the Stenothoe gallensis group with four new members, keys to Stenothoe worldwide, a new species of Parametopa and Sudanea n. gen. (Crustacea: Amphipoda) | 5 |
| 1280795f-eb48-4582-a3c8-8aa2544b3f35 | Macrobenthos monitoring at long-term monitoring stations in the Belgian part of the North Sea from 2001 on | 2559 |
| d56e1889-ab4e-4637-8446-558579902075 | Nahant Collection | 13 |
| 7e8ffb71-b4ab-40af-9ff1-514920b5ccd5 | Numérisation des données faune contenues dans les Bulletins de la Société Linnéenne de Bordeaux - Bulletin de la Société Linnéenne de Bordeaux, Tome 148 (N.S) n° 41 (3), 2013 - Données faune | 24 |
| deae7cbd-0c20-422f-a7f9-4e8acfb57c4a | Monitoring data from the federal state Saxony-Anhalt (Germany) | 625 |
| 04280626-04d1-4127-939d-931ceed3a9c0 | (Tab. 9.C) Presence and abundance of benthic fauna at station FINO 1 in 2005-2007 | 33 |
| 6d28a527-bf84-4e58-b133-0e60987b5e7c | NaGISA Project | 1227 |
| 4be707fb-c868-4548-9cb7-5354d8cdccd9 | Inventaire de la Réserve Naturelle de l'étang de Cousseau - Inventaire Entomologiques | 2 |
| 36becc50-3386-4454-88ef-8d72f7f48585 | Zooplancton del estrecho de Bransfield y alrededores de Isla Elefante, verano austral 2019-2020, durante la VI Expedición Científica de Colombia en la Antártica | 57 |
| 78ff8409-15b9-456f-9793-291b030190a7 | MSB Parasite Collection (Arctos) | 2 |
| 5fe48e2c-a83d-43b7-952a-e1e62c49ad23 | Monitoring data from the federal state Saxony (Germany) | 308 |
| 2931765e-0682-4a38-85eb-032964cc3792 | SeamountsOnline | 54 |
| 62c2f728-f762-11e1-a439-00145eb45e9a | Bachabschnitt der Nette (Osnabrück) | 2 |
| d6daa6b6-fe00-4a7c-a6f6-d957a7f45698 | Réseau de suivi des macro-invertébrés aquatiques - Inventaire macroinvertébrés | 432 |
| 976e77c2-f762-11e1-a439-00145eb45e9a | Abundance of benthos infauna at station GIK23038-1 | 1 |
| bf75c1e6-54d7-4ff6-8f18-c6c578a4ce05 | (Table 4) Abundance of under-ice amphipods on flat ice and ice ridges sampled during the 2005 Hidden Ocean expedition | 108 |
| 976d1f76-f762-11e1-a439-00145eb45e9a | Abundance of benthos infauna at station GIK23037-3 | 1 |
| 976fdd74-f762-11e1-a439-00145eb45e9a | Abundance of benthos infauna at station GIK23040-1 | 6 |
| 976297ae-f762-11e1-a439-00145eb45e9a | Abundance of benthos infauna at station GIK23002-1 | 3 |
| 97693442-f762-11e1-a439-00145eb45e9a | Abundance of benthos infauna at station GIK23017-1 | 1 |
| 6a8e7e44-4ee1-4431-a0d9-8c55f7d40b83 | Macrobenthos monitoring in the Mar Piccolo of Taranto in June 2013 and April 2014 | 29 |
| 62f99788-f762-11e1-a439-00145eb45e9a | Kinderwald Hannover | 1 |
| 6fa5b732-f762-11e1-a439-00145eb45e9a | Distribution of meiobenthos in sediment core M19_216-2 of the northeastern Atlantic (Table 1) | 3 |
| 82e0273d-a8f2-4c68-aa48-b572a449bff2 | Zooplankton community in Gazi Creek sampled monthly between 1990 and 1991 | 34 |
| 8c67835d-a31b-467c-9a0d-d61913844518 | Macroinvertebrados asociados a fuentes hídricas del cerro Alto Utría, municipio de Bahía Solano, Chocó. 2021 | 8 |
| a8dbc2f4-188c-4d49-b5bf-add7e31f62c2 | Benthic macrofauna from the abyssal equatorial and South Atlantic collected during the DIVA 1&3 expeditions (2000, 2009) using an epibenthic sledge | 18 |
| e45afc03-4897-4bdf-ade1-bc07198674e7 | Bongo Zooplankton Data from the R/V TINRO, NOAA Bell M. Shimada and F/V Northwest Explorer during the 2022 International Year of the Salmon Pan-Pacific Winter High Seas Expedition | 328 |
| d6751eb6-844b-4b9d-93d5-a842a2f98412 | Benthic macrofauna from the abyssal equatorial and South Atlantic collected during the DIVA 2 expedition (2005) using an epibenthic sledge | 7 |
| 5199d77b-fc69-4c85-94f2-4d3e63e777ff | National Benthic Inventory | 20319 |
| 62ef6a6a-f762-11e1-a439-00145eb45e9a | Weser und Weseraue bei Höxter, Godelheim und Boffzen | 3 |
| aaf01eba-1bbd-43bb-b0b9-a1f6641051e2 | (Table B) Fauna associated with Bathymodiolus azoricus mussel beds within the Lucky Strike hydrothermal field, Mid-Atlantic Ridge | 24 |
| 08015d18-e850-4d59-bb8f-36e92dad757c | (Table C) Fauna associated with Bathymodiolus azoricus mussel beds within the Rainbow hydrothermal field, Mid-Atlantic Ridge | 20 |
| 633058b8-f762-11e1-a439-00145eb45e9a | Bachpatenschaft Beeke | 1 |
| da27a3b0-5b28-4e4a-9742-dfc77a1e539f | Crustacea specimens of Ryukyu University Museum (Fujukan) | 66 |
| 7fcf1da7-2c7a-4e69-a60d-efa5f0b4ec77 | HBIs and sterols in zooplankton Nansen Legacy Q3 | 8 |
| 81ffa2d0-f762-11e1-a439-00145eb45e9a | Nekton and plankton counts from RMT (320 µm) hauls of cruise GAE 1975/76 | 256 |
| 8205fbda-f762-11e1-a439-00145eb45e9a | Nekton and plankton counts from RMT (4500 µm) hauls of cruise GAE 1975/76 | 272 |
| 176bec90-cdc1-497b-b38d-60f24038e6c5 | Colección de Macroinvertebrados Acuáticos de la Amazonia Colombiana | 6 |
| 60e410ed-897a-475c-b772-6abaf229ed9e | Evaluación del macrobentos de la plataforma continental del Caribe colombiano | 24 |
| 84c333ce-f762-11e1-a439-00145eb45e9a | Galathea II, Danish Deep Sea Expedition 1950-52 | 135 |
| 81d07b0e-f762-11e1-a439-00145eb45e9a | Abundance of benthic infauna in surface sediments from the North Sea sampled during LITTORINA cruise LI1986/1 | 814 |
| 8e9974a4-12e2-475f-9aec-5abdf24a1f50 | Programme CROMIS: carnet de plongée en ligne de la FFESSM - Observations d'espèces subaquatiques collectées par les utilisateurs de CROMIS | 41 |
| 04daaaf2-0d57-414d-8656-28c0137b638a | New Mexico Museum of Natural History and Science (NMMNHS) Insect specimens (Arctos) | 1 |
| b929f23d-290f-4e85-8f17-764c55b3b284 | Bernice P. Bishop Museum | 1172 |
| 81965da2-f762-11e1-a439-00145eb45e9a | Nekton and plankton counts from Wechselnetz-Hai (300 µm) hauls of cruise GAE 1975/76 | 46 |
| de8fec01-4cf7-4062-a6e8-ec489184b992 | Colección de Referencia de Macroinvertebrados Bentónicos del Museo de Ciencias de la Universidad El Bosque | 6 |
| 350f00a7-db1f-4133-bc07-71de716339da | Rockfish Recruitment and Ecosystem Assessment Survey, Catch Data | 205 |
| 25bf34e6-48ef-41aa-9b62-876ca0c66a2a | Invertebrates from the ANTARXXVII Leg1 expedition to the Bransfield Strait, Antarctica - data | 160 |
| 7e380070-f762-11e1-a439-00145eb45e9a | Natural History Museum (London) Collection Specimens | 1133 |
| 2b87bd63-7431-4ba1-a728-4821d20e282e | BOEM Beaufort Sea, Alaska, Fish and Invertebrate Haul and Catch, with Oceanography, 2008 | 11 |
| 44e8b95a-1850-4507-8fa0-a3a07f5dd936 | Crustacean collection of the National Museum of Nature and Science | 101 |
| 379d343c-81e6-4487-91b0-bf8ce92028da | Macrobenthos of the mangroves of Gazi Bay (Kenya) sampled in September 1990 | 10 |
| c136b812-46b5-4225-ae28-3466f5bdcd3f | Biodiversity of Zmiinyi Island | 2 |
| e707e6da-e143-445d-b41d-529c4a777e8b | Suffolk Biodiversity Information Service (SBIS) Dataset | 1360 |
| 5e2c9b3a-9d7c-4871-af8c-144e4f40e9d2 | Marine biological observation data from coastal and offshore surveys around New Zealand | 344 |
| 2bf7b2c0-34dc-4458-8ee4-4f99fac03b33 | Biodiversity4all Research-Grade Observations | 4 |
| 6ec0db76-23c5-44b5-80f8-ad7e2aa17773 | RHS monitoring of native and naturalised plants and animals at its gardens and surrounding areas | 12 |
| 37a1816f-d4fd-4d30-bf74-72ca5bf0eb90 | Marine Species Records from Skomer Marine Conservation Zone (MCZ) Marine Monitoring Programme | 1609 |
| 25d4f82b-b2bc-4197-bc11-27c8e9a25439 | Shropshire Ecological Data Network database | 35 |
| 66f39e15-e74d-491c-b5d2-397cd6bad884 | DASSH Data Archive Centre expert sightings records | 2 |
| 83c964d4-f762-11e1-a439-00145eb45e9a | Gwaii Haanas Invertebrates (OBIS Canada) | 1254 |
| 8598edb6-f762-11e1-a439-00145eb45e9a | IndOBIS, Indian Ocean Node of OBIS | 180 |
| 7d472812-f762-11e1-a439-00145eb45e9a | Crustacea in surface sediments off Sylt collected during HEINCKE cruise HE262 | 14256 |
| 4797da32-5693-4281-a8f1-bf99bae66ddb | NSW Waterwatch | 242 |
| 486ba5e5-4e22-47b5-82f4-9d7c16879276 | Manx Wildlife Trust - Records | 3 |
| 2f46fe6f-b9fd-4dba-8ca2-2cb1368ceed8 | UAM Fish Collection (Arctos) | 1 |
| 11fcc792-8225-48e1-9eb5-9a4e617e55ab | Melitidae, the Eriopisella group * | 2 |
| af18f3f8-f899-4c97-af47-8a110f856f92 | Pelagic amphipods (Crustacea: Amphipoda: Hyperiidea) from the southern Gulf of Mexico | 485 |
| 2c12acd9-1600-487d-a809-2c2743381112 | Ontario BioBlitz Species Records | 5 |
| f3102dce-38a7-4dc5-bd54-3b3e3e76cb1f | Hyperbenthos community in the salt marsh of Saeftinghe in 1990 and 1991 | 76 |
| e351951e-32da-46d6-b61c-e9816d39e0d4 | North Merseyside Local Species Data (DEFRA Grant 23169) | 5 |
| 7b6f0500-f762-11e1-a439-00145eb45e9a | RMT Trawl catch from the 1982/83 V2 ADBEX I voyage | 205 |
| 040c5662-da76-4782-a48e-cdea1892d14c | International Barcode of Life project (iBOL) | 20783 |
| 90599043-45e0-46ed-b521-04140fbdd830 | Microbial diversity and skin mucosal microbiota in farmed Atlantic salmon with ulcerative disorders, raw sequence reads | 1 |
| 7e20d620-f762-11e1-a439-00145eb45e9a | Crustacea in surface sediments off Sylt collected during HEINCKE cruise HE272 | 14256 |
| 84ca3ea8-f762-11e1-a439-00145eb45e9a | Nivå Bay species list, Zealand, Denmark | 9 |
| 95e635d4-f762-11e1-a439-00145eb45e9a | Banco de Datos de la Biodiversidad de la Comunitat Valenciana | 337 |
| 71a0da97-201f-4fee-982a-d138e21a0ecb | Estructura Ecológica de la Subregión Norte de Caldas, Contrato para actividades científicas y tecnológicas No. 222-2018 entre Corpocaldas y WCS | 4 |
| 0e3d6f05-a287-4ffd-852d-4e17db22d810 | ecoscope_observation_database | 314 |
| efde0b80-02d6-4e50-9e6a-4b4329bfeec6 | Records of Invasive Non Native Species held on the Cofnod database | 14 |
| a443552e-b521-4d0e-875f-0b96fc860cc7 | LifeWatch observatory data: zooplankton observations by imaging (ZooScan) in the Belgian Part of the North Sea | 181 |
| 7bf9486e-f762-11e1-a439-00145eb45e9a | Crustacea in surface sediments off Sylt collected during HEINCKE cruise HE278 | 14256 |
| 7b7269b6-f762-11e1-a439-00145eb45e9a | RMT Trawl catch from the 1986/87 V7 AAMBER voyage | 76 |
| 1e09d985-8f58-4a34-ab86-17e55f203c8a | niek_2022 | 81 |
| f1966ff2-964a-4ca8-8be3-85b818a49899 | Benthic macrofauna from experimental wood and rock substrates deployed for 10 months (2020-2021) in the Southern California Borderland. | 24 |
| ba0b34a0-c7b8-11de-b279-f12cadb93607 | National Invasive Species Database | 427 |
| c1c5ef4b-8309-4f04-9247-3f7644260c85 | Occurrences in SinBiota | 626 |
| 7124b64e-f762-11e1-a439-00145eb45e9a | Abundance of macrozoobenthos in surface sediments sampled during POLARSTERN cruise ANT-V/1 | 27 |
| 712244c2-f762-11e1-a439-00145eb45e9a | Benthos counted on dredged samples during Valdivia cruise VA53 | 113 |
| 700bb528-f762-11e1-a439-00145eb45e9a | Meiofauna abundances at the Arctic Håkon Mosby Mud Volcano (HMMV) of sediment core PS64/390-1c | 5 |
| fb98e53c-add6-4870-b745-db11cbea753d | LERN Invasive Non-native Species Records | 141 |
| 700934ec-f762-11e1-a439-00145eb45e9a | Meiofauna abundances at the Arctic Håkon Mosby Mud Volcano (HMMV) of sediment core PS64/367-1 | 5 |
| b7cd5ad0-f65a-4de1-a1bb-318cf5ae12f8 | LERN Records | 211 |
| 6fba3bd0-f762-11e1-a439-00145eb45e9a | Distribution of meiobenthos in sediment core M23_199 of the northeastern Atlantic (Table 1) | 4 |
| 82b2e911-9636-47e1-ba1b-e8ab3fb93f9e | RMT Trawl catch from the 1984/85 V5 SIBEX2 voyage | 178 |
| 409db43e-79bc-4497-b100-ef54766b3651 | RMT Trawl catch from the 2003/04 V3 BROKE-West voyage | 75 |
| f1a528c7-0efd-48d5-abde-15199aa465ec | A new crab-associated amphipod of the genus Isaeopsis Barnard, 1916 (Amphipoda Senticaudata: Ischyroceridae) from southern Chile | 2 |
| 1ff05317-283d-474a-b72b-078b87c5ed55 | aen-jc2-1-mesozooplankton-metadata | 40 |
| 0ce9fc33-d200-43da-8340-697b09610f74 | aen-q2-mesozooplankton-biodiversity | 32 |
| 82074562-f762-11e1-a439-00145eb45e9a | Nekton and plankton counts from neuston net (300 µm) hauls of cruise GAE 1975/76 | 399 |
| 82087c52-f762-11e1-a439-00145eb45e9a | Nekton and plankton counts from bongo net (300, 500 µm) hauls of cruise GAE 1975/76 | 19 |
| 26826f72-6fed-4b8f-94d5-6201f2dfab5b | (Figure 2) Vertical density profiles of meiofauna taxa in surface sediments of Potter Cove, King George Island, Antarctic Peninsula | 15 |
| 93ebfc8f-c27a-4451-9ec2-f73af2dcc18e | (Table III) Abundance of crustacea orders during RSS James Clark Ross cruise JR144 | 15 |
| c2e10b6c-52b2-45d7-819a-66383fb6dfd6 | Nansen Legacy Seasonal Study Q1 2nd March - 25th March 2021 Mesozooplankton Biodiversity | 51 |
| 7d17a2b2-8751-41b5-ac9b-48effc1ea236 | BRERC species records from all years at full resolution excluding Notable Species within the last 10 years | 541 |
| 43524467-3aab-4074-8b2d-5c600f79c85e | Kyklades-data of the Central Aegean Sea | 61 |
| 5d5fe7b9-62b6-4781-91a2-64b0c8a733e6 | Tab. 5: Artenzusammensetzung, mittlere Individuendichte, Diversität und Eveness der benthischen Gemeinschaft | 10 |
| c1492854-5d15-40e9-9ee7-24a18ac05112 | Biodiversitätsdatenbank Salzburg | 3 |
| 7e1f7de8-f762-11e1-a439-00145eb45e9a | Crustacea in surface sediments off Sylt collected during HEINCKE cruise HE258 | 14256 |
| baa7d7c9-8b9b-4378-91fb-b82522dfbf32 | Fatty acids in zooplankton Nansen Legacy Q4 | 19 |
| 95e464b4-e286-414b-9c59-6fdc9a4afc14 | Lipid classes in zooplankton Nansen Legacy Q4 | 6 |
| 021366ac-62e5-4acc-a439-4c37b3288d87 | HBIs and sterols in zooplankton Nansen Legacy Q4 | 14 |
| 34ed3819-a996-47ad-bc36-ee0a8471a57d | Fatty acids in zooplankton Nansen Legacy Q3 | 10 |
| e00f784b-e406-4cb0-8117-559b924ab3f4 | PELD-ELPA Continuous monitoring of the micro and mesozooplankton of the Patos Lagoon estuary and adjacent coastal area | 66 |
| 48125541-00d5-498e-af76-9472e51c459c | aen-q3-mesozooplankton biodiversity | 68 |
| cd6fe3a0-eee9-4066-8420-e36313ed833d | FBIP:IZIKO-UCT:Historical Invertebrates (1930-1980) | 759 |
| 43f81826-e665-4112-925e-1426b7043f43 | Création d'une usine de traitement innovant des co-produits de volailles - Inventaire faune - flore | 1 |
| 7917fde8-f762-11e1-a439-00145eb45e9a | Benthic macrofauna abundance of subcores of sediment core 13200-045 | 52 |
| 791488d4-f762-11e1-a439-00145eb45e9a | Benthic macrofauna abundance of subcores of sediment core 12930-084 | 52 |
| 78fe475e-f762-11e1-a439-00145eb45e9a | Benthic macrofauna abundance of sediment core 13200-008 | 10 |
| 78f94178-f762-11e1-a439-00145eb45e9a | Benthic macrofauna abundance of sediment core 13078-015 | 14 |
| 7905489c-f762-11e1-a439-00145eb45e9a | Benthic macrofauna abundance of sediment core 13200-047 | 10 |
| 86442d3e-f762-11e1-a439-00145eb45e9a | RMT Trawl catch from the 1980/81 V5 FIBEX voyage | 406 |
| 89e1d32e-f762-11e1-a439-00145eb45e9a | Wanderweg am Windebyer Noor (bei Eckernförde) | 1 |
| 79101128-f762-11e1-a439-00145eb45e9a | Benthic macrofauna abundance of sediment core 13200-081 | 10 |
| 78f7eda0-f762-11e1-a439-00145eb45e9a | Benthic macrofauna abundance of sediment core 13078-013 | 14 |
| 78e59a2e-f762-11e1-a439-00145eb45e9a | Benthic macrofauna abundance of sediment core 12930-084 | 14 |
| 89e33052-f762-11e1-a439-00145eb45e9a | BUND - Dassower See (Lübeck/Dassow) | 3 |
| 8647dc9a-f762-11e1-a439-00145eb45e9a | Marine fauna survey of the Vestfold Hills and Rauer Island, 1981-82 | 66 |
| 89268632-f762-11e1-a439-00145eb45e9a | Zooplankton Abundance Based on Taxa and Life Stages or Size | 250 |
| 12d4db8d-5529-43da-b790-2796cfd5c09b | Fish and zooplankton from RMT-8 net hauls on the BROKE voyage | 1062 |
| 5583a79b-9487-4d1a-b405-448e951ac6f4 | aen-q4-mesozooplankton biodiversity | 75 |
| ba20df08-d873-4738-931d-a595126eb822 | (Table 2) Megafauna density 2002, 2004 and 2007 in the deep-sea observatory AWI-HAUSGARTEN, Fram Strait | 3 |
| 8a6232d7-f93e-45a3-8b16-556aa732cd39 | Colorado Plateau Museum of Arthropod Biodiversity | 4 |
| 54dd1589-f730-4c64-a891-06f2f25f71b1 | Colección Carcinológica de Yucatán | 275 |
| 6fa33412-f762-11e1-a439-00145eb45e9a | Distribution of meiobenthos in sediment core M19_201-2 of the northeastern Atlantic (Table 1) | 4 |
| 6fa8643c-f762-11e1-a439-00145eb45e9a | Distribution of meiobenthos in sediment core M19_223-2 of the northeastern Atlantic (Table 1) | 4 |
| 2b00ca94-c839-4a28-8c58-1412523958f1 | Records of protected animals species in Ukraine | 6 |
| f6861927-f5cb-4d82-8645-580b1d2cb4d3 | Notes on biodiversity of Ukraine, Slovak Republic, Uzbekistan and Turkiye | 1 |
| 9793e4bc-f762-11e1-a439-00145eb45e9a | Benthic meiofauna abundance in surface sediment of core PLG93_BCORE5 | 8 |
| 8c3e7db0-5c1a-49df-af56-9cb84d8511ff | Patrick Roper’s Notebooks | 5 |
| 7b474f9c-f762-11e1-a439-00145eb45e9a | Amphipoda Hyperiidea of the Southern Ocean: catalogue and occurrences | 992 |
| a99ca3c9-7448-4bf6-aba5-0ef61b01232c | Rotherham Biological Records Centre - Non-sensitive Records from all taxonomic groups | 181 |
| 7b92bd24-f762-11e1-a439-00145eb45e9a | Ant'Phipoda Database (New records) | 1747 |
| 94c74198-9eaf-4b43-a349-0b566dcd648a | Invertebrate records from sites that are mainly across Scotland | 45 |
| 2e6a7bc5-1cb4-47e3-9628-fe1f1ac0c548 | Epimeria cleo sp. nov., a new crested amphipod from the Ross Sea, Antarctica, with notes on its phylogenetic affinities (Crustacea, Amphipoda, Eusiroidea, Epimeriidae) | 8 |
| 7a184e2e-1307-4473-afeb-6355a01b89d6 | Trawl catch data from Nansen Legacy Cruise - Arctic Basin Joint Cruise 2-2 | 34 |
| 7b52e794-f762-11e1-a439-00145eb45e9a | Weddell Sea macrozoobenthos EASIZ I | 339 |
| 6328b2a2-f762-11e1-a439-00145eb45e9a | Bäche im Mooswald (Vörstetten) | 1 |
| 8044ee43-c5fe-44da-ad96-cc1a9cfb91ba | UAM Insect Collection (Arctos) | 311 |
| 0ed0a394-5276-433e-bf8d-09ceaaee45e9 | Biota occurrence data from plankton surveys around New Zealand | 432 |
| 631aacbf-2f3d-4247-87d2-2fba6d0e293a | Meiofaunal abundance in sediments obtained during Belgica cruise BG06/13, Gollum Channels and Whittard Canyon | 85 |
| ece4ea69-9ec5-4773-a5dc-7a99b245e415 | Macroinvertebrate abundance in river biotopes across England and Wales | 1024 |
| cb0eb5bf-4759-4aa1-92b5-4143b0a64e15 | Kenai National Wildlife Refuge, Alaska (KNWR) Insect specimens (Arctos) | 12 |
| 4975ef02-e7cf-47b1-b659-8eec5e79ac30 | Meiofaunal abundance in sediments obtained during RRS Charles Darwin cruise CD179 | 60 |
| 631fb1ca-f762-11e1-a439-00145eb45e9a | Altendorfer Dorfbachhöhle / Sächsische Schweiz | 1 |
| 8c779294-1cc4-4257-95db-8f2a4e7e0f8e | (Table A) Abundance of species detected in mussel beds within hydrothermal fields on the Mid-Atlantic Ridge | 16 |
| 00539f2f-63f5-4f87-8df7-75b62293cb1d | Saltern of Margherita di Savoia Metagenome | 1 |
| 68efff74-9529-4d39-87cf-7d8a227498e4 | (Table B) Abundance of benthic fauna associated with shrimp swarms at hydrothermal fields on the Mid-Atlantic Ridge | 13 |
| 8a0313ea-f762-11e1-a439-00145eb45e9a | Grundwasserlebensraum im Englischen Garten (München) | 1 |
| 74557d01-7001-4862-860c-bd188f20ab15 | Meiofauna abundances in sediments from the Håkon Mosby Mud Volcano (HMMV) obtained during expeditions with Polarstern and Pourquoi Pas? | 14 |
| cc2031d1-395d-4052-8ce1-796a08c3dbf2 | DASSH Data Archive Centre - Statutory Surveys | 37590 |
| dc9b4f81-f75e-44e8-bb19-845e665ccb81 | The Ecology and Biogeography of Heard Island Marine Benthos 1987/88 | 22 |
| 96385e98-93b5-4e4a-8081-6e30b738816a | Biological observations from the Discovery Investigations 1925-1952 | 1830 |
| ead6339f-39f8-46be-b059-d1c48d88ab29 | Marine invertebrate collection NTNU University Museum | 3866 |
| ce8530a2-cd4b-4e51-9927-76bf49b5a9a6 | Welsh Invertebrate Database (WID) | 533 |
| 111105f7-661d-416e-bb9d-9e291d5fdb1f | EPA - National Lakes Assessment - Zooplankton - 2007 | 1 |
| 81cdf992-f762-11e1-a439-00145eb45e9a | Abundance of benthic infauna in surface sediments from the North Sea sampled during cruise DeHolland1986 | 2208 |
| 81cf34a6-f762-11e1-a439-00145eb45e9a | Abundance of benthic infauna in surface sediments from the North Sea sampled during cruise Senckenberg_04_1986 | 377 |
| 89dd9c32-f762-11e1-a439-00145eb45e9a | Biologische Station im Kreis Wesel | 1 |
| 81c024c0-f762-11e1-a439-00145eb45e9a | Abundance of the benthic infauna of the North Sea sampled during Victor Hensen cruise VH1486 | 2592 |
| 81b65972-f762-11e1-a439-00145eb45e9a | Abundance of benthic infauna in surface sediments from the North Sea sampled during Walther Herwig cruise WH220 | 84 |
| 817d4fc4-f762-11e1-a439-00145eb45e9a | (Table 4) Fauna composition of the community of Nephtys ferruginea - Amphiura acrystata in bottom sediments from the shelf edge in the California upwelling zone | 2 |
| cef450df-7155-4c9d-9eb3-7b9b4d868bfa | Sheffield and Rotherham Wildlife Trust - Records | 2 |
| 7f2209d6-f762-11e1-a439-00145eb45e9a | Abundance of macrobenthos organisms in the northern Wadden Sea in 2010 | 36 |
| 492d63a8-4978-4bc7-acd8-7d0e3ac0e744 | Norwegian Biodiversity Information Centre - Other datasets | 10821 |
| 97eccb39-8a7b-4711-82ef-5f61afc1cf0c | Dr Mary Gillham Archive Project | 91 |
| 7f20ae7e-f762-11e1-a439-00145eb45e9a | Abundance of macrobenthos organisms in the northern Wadden Sea in 2009 | 36 |
| b25ad09a-0626-4316-842e-d7bd76959ef5 | Análisis taxonómicos de macrofauna bentónica de Navantia Dársena de Cartagena y Navantia Ría de Ferrol (2021) | 8 |
| 95e8b28c-f762-11e1-a439-00145eb45e9a | Colecciones Zoológicas de la Universidad de León, Colección de Malacostráceos | 63 |
| 248934a5-3a64-4374-a0ba-2065ef420504 | Macrofauna collected on colonization surfaces at the East Pacific Rise 9 50 N hydrothermal vent field in 1998-2017 | 41 |
| 430fea04-64f9-4959-bdc2-f5f0835eecb3 | Invertebrate surveys of various ponds in Scotland between 2010 and 2015 | 50 |
| 7e9eeb78-f762-11e1-a439-00145eb45e9a | Species occurring in the mytilid and alvinellid communities within the 9°N hydrothermal field at the East Pacific Rise | 96 |
| 6647ca7e-cf35-4eb1-8b0c-b8756a1ba6bd | Marine Intertidal Phase 1 Species Dataset from the Countryside Council for Wales 1996-2005 | 986 |
| 928304f1-d313-47cd-b17b-0232bc27b727 | Seasearch Marine Surveys in Ireland | 85 |
| 44ded7da-4330-4741-bc1f-ba8d21e3e016 | Barcoding Dutch Harbor and St Paul's Marine Invertebrates | 2 |
| c45c8530-fd7d-4f30-a9e4-c6d243a4b404 | Seasearch Marine Surveys in the Isle of Man | 46 |
| 990e845f-836b-4467-bc5f-46c8495259ec | Seasearch Marine Surveys in Scotland | 718 |
| a899709f-9a73-4cac-84d8-a7dcc7b70280 | Seasearch Marine Surveys in the Channel Islands | 18 |
| 5ca32e22-1f1b-4478-ba7f-1916c4e88d67 | Inland water macroinvertebrate occurrences in Flanders, Belgium | 9552 |
| da6a07ed-9eee-460d-9448-910f542c1a7b | The crustaceans collection (IU) of the Muséum national d'Histoire naturelle (MNHN - Paris) | 5603 |
| 82d23348-0fee-4e2d-9152-48136ef54b2d | Seasearch Marine Surveys in Wales | 341 |
| eaf9401e-a5e3-4a27-89ad-2d6ac6559167 | Natural history museum data on Canadian Arctic marine benthos | 463 |
| 4caaf0c8-bd51-4d80-86ba-5544792d65ce | The structure of the cataract causing P 23 T mutant of human gamma-D crystallin | 4 |
| 04167aa4-4148-4691-a264-031922765fc1 | Effect of short-term meteorological disturbance on submergem aquatic vegetation and associated fauna | 18 |
| 7d4dcbe0-f762-11e1-a439-00145eb45e9a | Crustacea in surface sediments off Sylt collected during HEINCKE cruise HE206 | 14832 |
| 7d45e4a2-f762-11e1-a439-00145eb45e9a | Crustacea in surface sediments off Sylt collected during HEINCKE cruise HE218 | 6912 |
| 7d489aa8-f762-11e1-a439-00145eb45e9a | Crustacea in surface sediments off Sylt collected during HEINCKE cruise HE241 | 14256 |
| aba69a99-f9c9-45c5-b82e-385a59c0a8ce | Meiofauna and Nematode abundance from the West Florida Escarpment, NOAA Hydrosmac project | 2 |
| 108c14f7-d055-46d8-9306-c30b81984d30 | Macroinvertebrados bentónicos encontrados en ríos y quebradas del área de influencia del Distrito Regional de Manejo Integrado Cristales, Castillejo o Guacheneque (Boyacá) | 10 |
| 78e2e4a0-f762-11e1-a439-00145eb45e9a | Benthic macrofauna abundance of sediment core 12930-073 | 14 |
| c1df184e-1340-4ab1-a045-b6e4752f7fa2 | Data from Defra Family Organisations supplied to Staffordshire Ecological Record | 161 |
| 790b4a8a-f762-11e1-a439-00145eb45e9a | Benthic macrofauna abundance of sediment core 13200-075 | 10 |
| 2a101f26-35e1-4197-94ec-bd25033292a7 | Staffordshire Wildlife Trust Nature Reserves Inventory | 5 |
| 667e0044-3ff1-4365-8ecf-e637c7b31fa4 | A collection of Antarctic Phipoda | 1764 |
| 53bfee71-d855-4bbf-a017-06db1780ddfa | Zooplankton Species Biomass and Abundance Data, Arctic Marine Biodiversity Observing Network (AMBON) Chukchi Sea research cruise on the Norseman II, 11 August - 3 September 2015 | 83 |
| 6fb8f8e2-f762-11e1-a439-00145eb45e9a | Distribution of meiobenthos in sediment core M23_149 of the northeastern Atlantic (Table 1) | 3 |
| 614c6f96-f762-11e1-a439-00145eb45e9a | Feriendorf Ober-Seemen | 1 |
| 6fb2c918-f762-11e1-a439-00145eb45e9a | Distribution of meiobenthos in sediment core M23_108 of the northeastern Atlantic (Table 1) | 5 |
| 5df3c9be-d9a1-4c36-a5bc-bdf88b78dbe3 | Marine sites, habitats and species data collected during the BioMar survey of Ireland. | 642 |
| 96c10340-e43d-4542-8d34-7dd0c4b3b010 | Fauna y flora del municipio de Paipa, Boyacá | 3 |
| 6fc31b4c-f762-11e1-a439-00145eb45e9a | Distribution of meiobenthos in sediment core M23_199 (2) of the northeastern Atlantic (Table 1) | 4 |
| 619e1184-f762-11e1-a439-00145eb45e9a | Schulgelände Paul-Gerhardt-Schule-Dassel | 2 |
| 6fc1d8d6-f762-11e1-a439-00145eb45e9a | Distribution of meiobenthos in sediment core M23_149 (2) of the northeastern Atlantic (Table 1) | 3 |
| 6fbf4fbc-f762-11e1-a439-00145eb45e9a | Distribution of meiobenthos in sediment core M23_122 (2) of the northeastern Atlantic (Table 1) | 7 |
| 6fb7b95a-f762-11e1-a439-00145eb45e9a | Distribution of meiobenthos in sediment core M23_134 of the northeastern Atlantic (Table 1) | 4 |
| 61c198ac-f762-11e1-a439-00145eb45e9a | Kindergarten | 1 |
| 6fb67e0a-f762-11e1-a439-00145eb45e9a | Distribution of meiobenthos in sediment core M23_133 of the northeastern Atlantic (Table 1) | 2 |
| 61ded8a4-f762-11e1-a439-00145eb45e9a | Herrensee-Gebiet (Fischbachtal im Odenwald) | 1 |
| 61e42bd8-f762-11e1-a439-00145eb45e9a | Quellgebiet Flossach - Klassen 4 a und 4 b VS Tussenhausen | 1 |
| a9b4672d-3772-4428-a923-42e0b6e04a45 | Macrobenthos and Meiobenthos Tuktoyaktuk Harbor and Mason Bay 1985-1988 NOGAP | 200 |
| 6207feb4-f762-11e1-a439-00145eb45e9a | Naturschutzgebiet Lippeaue (Marl) - Pfadis in Sickingmühle | 1 |
| ef5c63cc-ed75-47b2-a6fd-60969f4fad07 | Kiel Bay intercalibration data set | 9 |
| 62168560-f762-11e1-a439-00145eb45e9a | Gieselbachtal Fulda-Harmerz | 1 |
| 6219dd46-f762-11e1-a439-00145eb45e9a | Rückkehr der Biber in Rheinland-Pfalz - Biber in der Primmerbach | 1 |
| a6f9f411-ab90-4d94-82de-f5edf8c3fa2a | Las colecciones del Museu Valencià d'Història Natural | 1 |
| 622aa1f8-f762-11e1-a439-00145eb45e9a | Isartal Dingolfing | 1 |
| 867c8490-f762-11e1-a439-00145eb45e9a | Zooplankton in the bay of Biscay (1995-2004 Yearly spring DEPM surveys) | 512 |
| 623ecebc-f762-11e1-a439-00145eb45e9a | Gewässer des Wartbergparks Stuttgart (beim Naturlabor der Umweltakademie Baden-Württemberg) | 2 |
| 9c064156-0252-4441-9021-63b4107ff9b7 | DFO Quebec Region Coastal biodiversity of the benthic epifauna of the St. Lawrence Estuary (2018-2019) | 39 |
| 629870fc-f762-11e1-a439-00145eb45e9a | Schulhof A.-Lindgren-Schule (Elmshorn) | 1 |
| 62a7764c-f762-11e1-a439-00145eb45e9a | Mittelriede Höhe Gliesmarode-Braunschweig | 1 |
| 2fa0cdfb-11c5-443e-b836-b4ea8191cfda | Macrobentos de cuatro playas de alta energía ubicadas en la Península de La Guajira, noroeste del Golfo de Venezuela | 2 |
| 03e5566b-7802-45cc-a1be-bac840e2a133 | Ross Sea Biodiversity Survey 2004 (BioRoss) | 667 |
| 62c57912-f762-11e1-a439-00145eb45e9a | Draubiotop Lavamünd | 2 |
| 62ce8b92-f762-11e1-a439-00145eb45e9a | Issumer Fleuth | 1 |
| 62db1876-f762-11e1-a439-00145eb45e9a | AKG-Gelände in Bensheim | 8 |
| 62dd9ace-f762-11e1-a439-00145eb45e9a | Brenz (Heidenheim) | 1 |
| 62ea62d6-f762-11e1-a439-00145eb45e9a | Heider Bergsee (Brühl) | 2 |
| d3ed14a0-da64-4179-9643-4927deeb5126 | Stoke-on-Trent Environmental Survey results (1982-1984) | 58 |
| 62ee2b50-f762-11e1-a439-00145eb45e9a | Baumbach Arnsberg, Klasse 12, Fachbereich Biologie | 1 |
| 2ae1fe04-6f12-4766-bdee-50e5b2dc1148 | Species within North Ayrshire from 1984 - Present | 1 |
| 6316812c-f762-11e1-a439-00145eb45e9a | LBV-Kindergruppen Markt Tussenhausen | 1 |
| 6329f84c-f762-11e1-a439-00145eb45e9a | Hache im Ellernbruch (Sudweyhe / Weyhe) | 1 |
| 636e91aa-f762-11e1-a439-00145eb45e9a | GEO-Hauptveranstaltung im Nationalpark Bayerischer Wald | 2 |
| 6373dcf0-f762-11e1-a439-00145eb45e9a | Rohrmeistereiplateau und angrenzendes Gebiet | 1 |
| a71b06c8-c62d-46ed-8daa-f10b0aeca0da | Marine Offshore Seabed Survey data held by JNCC | 4825 |
| d80c46be-b600-44d5-9758-ef5159d40002 | Zooplankton Bongo Net Data from the 2019 and 2020 Gulf of Alaska International Year of the Salmon Expeditions | 159 |
| f8c6823b-c854-48b1-a449-5cc1704bc7a3 | Hypogean macro-Crustacea records | 1661 |
| 6403d346-f762-11e1-a439-00145eb45e9a | Gronau - auf der Suche nach dem Neunauge | 2 |
| 4d0b1b2a-3209-49ce-bb93-7503e4868019 | NIWA Invertebrate Collection | 5586 |
| 642f5c46-f762-11e1-a439-00145eb45e9a | "Ahrschleife bei Altenahr" | 1 |
| 90f2713a-79ac-4d96-9a66-889a5fb9abb1 | Staying cool also has consequences: increased sea ice cover disrupts food web structure in East Antarctica-Data | 27 |
| 647d2728-f762-11e1-a439-00145eb45e9a | BUND Naturschutzzentrum St. Julian | 1 |
| 64885832-f762-11e1-a439-00145eb45e9a | Art´n Vielfalt im Kinderwald | 1 |
| 29cd134d-99ac-4393-85ac-c03c1aa36452 | Caracterización bionómica de zonas de dragado y de afección de obras en la Ría de Ferrol | 51 |
| 6490a924-f762-11e1-a439-00145eb45e9a | Die Bachspezies | 1 |
| 9e31304a-0379-4b8e-8fb2-e0850abb0f0b | Análisis taxonómicos de macrofauna bentónica para el Plan de Vigilancia Ambiental de Navantia Cartagena | 15 |
| 7149e310-f762-11e1-a439-00145eb45e9a | Mangfalltal | 1 |
| 714f5cd2-f762-11e1-a439-00145eb45e9a | LBV 100 - Artenvielfalt am Rothsee | 1 |
| 4f8355f0-07b5-4b0b-a08c-34b719fc3ad5 | Informe "Control de Organismos" en la Ría de Ferrol 2019 | 2 |
| 715e8a40-f762-11e1-a439-00145eb45e9a | Schatzinsel Norderney | 5 |
| 71613c86-f762-11e1-a439-00145eb45e9a | Biotope in Rheine - Aktion 350 | 1 |
| 716fe722-f762-11e1-a439-00145eb45e9a | Nationalpark Jasmund | 1 |
| 71712cae-f762-11e1-a439-00145eb45e9a | Bulau | 2 |
| 551108a8-3391-42b1-b4ec-88253d5d01fb | Complementary Benthic Biodiversity Baseline Survey, Block 58, Suriname - 2021 - TotalEnergies E&P Suriname B.V. | 3 |
| 4bc14221-60af-43e5-8e92-0c2b262b9945 | Bringing Reedbeds to Life Invertebrate Survey of three key reedbed sites in England in 2009, 2010 | 54 |
| 717b3a8c-f762-11e1-a439-00145eb45e9a | Rur | 1 |
| eb97c199-0555-4da2-94e4-e9d6182d3944 | Ecological study of the fish fauna near the mouth of the Zwin in 1985 and 1986 | 11 |
| 4e1e783d-421c-404e-aa95-803235c8fe16 | Geotargeting spatial and temporal data of Italian freshwater high-altitude macroinvertebrates | 5 |
| 71ac5248-f762-11e1-a439-00145eb45e9a | Biotope entdecken im Kockmecker Siepen (Sauerland) | 1 |
| 71ad9112-f762-11e1-a439-00145eb45e9a | Listhof und Umgebung | 1 |
| 8f925919-18a5-4bfb-af5b-db45e18e3e14 | Macrobenthos of the Western Scheldt estuary in September 1978 | 3 |
| 7a709d94-f762-11e1-a439-00145eb45e9a | Bremerhaven | 1 |
| 7a73205a-f762-11e1-a439-00145eb45e9a | Bayerische Donau - Tapfheim | 1 |
| 7a7d7c80-f762-11e1-a439-00145eb45e9a | "Schule am Inselsee" Güstrow | 1 |
| 7903e39e-f762-11e1-a439-00145eb45e9a | Benthic macrofauna abundance of sediment core 13200-045 | 10 |
| 7a8e1086-f762-11e1-a439-00145eb45e9a | Fluss - Vielfalt | 1 |
| 7a91d4d2-f762-11e1-a439-00145eb45e9a | Gemeinschaftsaktion Koordinatoren für nachhaltige Bildung/Wittstocker Grundschulen | 1 |
| 89754f1a-f762-11e1-a439-00145eb45e9a | BÜG | 1 |
| 89795042-f762-11e1-a439-00145eb45e9a | Klutensee | 1 |
| 8984fb2c-f762-11e1-a439-00145eb45e9a | FFH-Gebiet Ahrbachtal | 1 |
| 898909e2-f762-11e1-a439-00145eb45e9a | GEO-Hauptveranstaltung (NLP Harz / Hochharz) | 2 |
| 898bb3f4-f762-11e1-a439-00145eb45e9a | Artenvielfalt auf den Elbwiesen (Dessau) | 3 |
| 899341c8-f762-11e1-a439-00145eb45e9a | Schlichemquelle (Tieringen/Meßstetten) | 1 |
| 4d456575-b001-4c60-b583-3dbf2520562a | Peces y macroinvertebrados del río Saza-Gámeza, cuenca alta del río Chicamocha, macrocuenca del Magdalena-Cauca, Boyacá, Colombia | 3 |
| 89a04454-f762-11e1-a439-00145eb45e9a | Langenberger Forst am Ochsenweg/ Niebüll-Leck | 1 |
| 89acc3b4-f762-11e1-a439-00145eb45e9a | Königstetten | 2 |
| 89b0c176-f762-11e1-a439-00145eb45e9a | Erzental (Oberotterbach) | 1 |
| 89b797e4-f762-11e1-a439-00145eb45e9a | Erlengraben/Lipp-Tal (Östringen) | 2 |
| 3eddefab-b0f5-4d68-83a6-ccc0d42c38ca | Caracterización biótica realizada para el estudio de impacto ambiental interacción de operaciones títulos mineros 4079, 1910T, 1913T y 02-004-98 | 2 |
| 89d55c66-f762-11e1-a439-00145eb45e9a | Artenvielfalt auf der Weide - GEO-Hauptveranstaltung in Crawinkel | 1 |
| 89e0691c-f762-11e1-a439-00145eb45e9a | GEO-Hauptveranstaltung (Duisburg) | 4 |
| d3bb4a63-7277-482c-b1e3-413301720130 | Scientific Results of the New Zealand Government Trawling Expedition, 1907 | 3 |
| 89ee5cde-f762-11e1-a439-00145eb45e9a | Artenvielfalt der Nordsee - Bremerhaven (Dorum-Neufeld) | 8 |
| 89f52a6e-f762-11e1-a439-00145eb45e9a | Pilstingermoos | 1 |
| 8a00478c-f762-11e1-a439-00145eb45e9a | 20 Jahre Naturschutzgebiet Dreienberg | 4 |
| 8a0df3e6-f762-11e1-a439-00145eb45e9a | GEO-Hauptveranstaltung Bodden (Vilm) | 8 |
| f885d3e2-0310-43f5-a902-50dda62e6a68 | Diet results from Adelie penguins at Bechervaise Island and Whitney Point, 2012/13 | 243 |
| 8a2380d0-f762-11e1-a439-00145eb45e9a | Langes Tannen in Uetersen | 1 |
| 7e48f393-1f30-4ca7-98f9-3106358f2a1e | Ampelisca Krøyer, 1842 (Amphipoda: Ampeliscidae) in the Persian Gulf and the Gulf of Oman | 16 |
| ee069354-4c2f-4ace-ae26-86243fc0592a | Antarctic Biodiversity Studies 2006 (Ross Sea, Scott Island, and Balleny Islands) (TAN0602) | 97 |
| 78e431f2-f762-11e1-a439-00145eb45e9a | Benthic macrofauna abundance of sediment core 12930-081 | 14 |
| 78c6453e-f762-11e1-a439-00145eb45e9a | Matter enrichment of the macrofauna community of MAC station 13368-008 | 16 |
| 8a538438-f762-11e1-a439-00145eb45e9a | Schulprojekt (Bremen) | 1 |
| 62eceb00-f762-11e1-a439-00145eb45e9a | Beisetal bei Niederbeisheim | 2 |
| 8a5fc6f8-f762-11e1-a439-00145eb45e9a | Lüner Holz (Lüneburg) | 1 |
| 8a669410-f762-11e1-a439-00145eb45e9a | Frohlinder Mühlenbach (Dortmund-Kirchlinde) | 1 |
| c700f790-3418-4f26-bccc-cf001dd38057 | A taxonomic study on the Phtisicidae (Crustacea: Amphipoda) of New South Wales, Australia | 23 |
| 62e025aa-f762-11e1-a439-00145eb45e9a | Glemstal (Leonberg) | 2 |
| 8a850076-f762-11e1-a439-00145eb45e9a | 3. Tag der Artenvielfalt Hockenheim | 2 |
| a0a4d131-f53f-43b2-a1ba-254473b8a006 | Marine Invertebrate voucher specimens at the Florida Biodiversity Collection, Florida Fish and Wildlife Conservation Commission | 4233 |
| 8a8bde64-f762-11e1-a439-00145eb45e9a | Hintere Halde | 1 |
| 570ceb60-ac58-4b98-9723-54000ee7b391 | FBIP:SAEON: Historical Research Survey Database (1897-1949) | 15 |
| 8aa2023e-f762-11e1-a439-00145eb45e9a | Freigelände Naturschutzscheune Reinheimer Teich (Kreis Darmstadt-Dieburg) | 1 |
| 959d80f0-f762-11e1-a439-00145eb45e9a | Leben in und an der Ruwer sowie ihren Nebenbächen | 1 |
| 95a7b110-f762-11e1-a439-00145eb45e9a | Klosterwald Itzehoe | 1 |
| 95ac1e4e-f762-11e1-a439-00145eb45e9a | Kaulsdorf | 1 |
| 95c27b3a-f762-11e1-a439-00145eb45e9a | Kohlstattbrunnental | 2 |
| 34fbcf59-d9bb-47e7-9672-99e13ea8c736 | Royal BC Museum - Invertebrates Collection | 1168 |
| 62c6c79a-f762-11e1-a439-00145eb45e9a | Spreewaldfließe und Feuchtwiese bei Lübbenau | 1 |
| 648f44da-f762-11e1-a439-00145eb45e9a | Vergleich der Fauna eines naturbelassenen mit einem wasserwirtschaftlich veränderten Gewässer | 1 |
| 8a628bd6-f762-11e1-a439-00145eb45e9a | Stadtpark Sulzbach-Rosenberg | 1 |
| 959654c4-f762-11e1-a439-00145eb45e9a | Tag der Artenvielfalt mit SchülerInnen des Europa-Gymnasiums in Wörth am Rhein | 1 |
| 8aa9a002-f762-11e1-a439-00145eb45e9a | Walldorf-Wiesloch: "Natur über den Gleisen" | 3 |
| 61a22378-f762-11e1-a439-00145eb45e9a | Wangerooge | 1 |
| 8a80de10-f762-11e1-a439-00145eb45e9a | Weidenhüttendorf an der Würm (München) | 2 |
| fea77396-7b26-44cb-8580-aefadc8db276 | The cryptogamy collection (PC) at the Herbarium of the Muséum national d'Histoire Naturelle (MNHN - Paris) | 8 |
| 631a64ea-f762-11e1-a439-00145eb45e9a | Wattenmeer-Safari (Wurster Watt) | 1 |
| 818504f8-f762-11e1-a439-00145eb45e9a | Abundance of benthic infauna in surface sediments from the North Sea sampled during Belgica cruise BG86/1 | 494 |
| 7c0cd863-8b81-4937-84f9-2f596fd3fa79 | SPC NECTALIS Zooplankton/Micronekton specimens, New Caledonia 2014 | 450 |
| 62566d9c-f762-11e1-a439-00145eb45e9a | Wulfsmuehle/Pinnau | 1 |
| 89b4d964-f762-11e1-a439-00145eb45e9a | Stausee (Oberdigisheim/Meßstetten) | 2 |
| 6b974e5b-562e-4631-9ad7-660354e07d9d | Fauna of the splash zone of Lake Baikal | 17 |
| 7acc5972-f762-11e1-a439-00145eb45e9a | Wasser-Lernort Nettemündung | 1 |
| 61be0dc2-f762-11e1-a439-00145eb45e9a | Stever | 1 |
| 36094b2b-bd50-4c6d-af52-264a155fc76f | MBON POLE TO POLE: SANDY BEACH BIODIVERSITY OF YUCATAN COAST | 26 |
| 62439f70-31d3-47c8-bda8-8cdf9e1f3710 | (Table 4) Biomass of (benthic) amphipods in the Bering and Chukchi seas | 47 |
| 8995784e-f762-11e1-a439-00145eb45e9a | Mooswald (Freiburg) | 1 |
| 50f48e48-1c94-4a50-889b-860b27ca4471 | Vinogradovopleustes punctatum, new genus, new species, a pleustid amphipod from the Okhotsk Sea (Crustacea: Amphipoda: Gammaridea: Pleustidae: Pleusymtinae) | 6 |
| 891e34fa-f762-11e1-a439-00145eb45e9a | Antarctic Amphipod Crustaceans: Ant'Phipoda Database (BIANZO) | 6702 |
| 3a1e6d69-9ec6-4dc9-bdaa-c2d77bc7e174 | TWIC General Records (2015 - present) | 2 |
| 898a53c4-f762-11e1-a439-00145eb45e9a | Hamberger Brücke / Würmtal (Pforzheim) | 2 |
| b577bbff-2ed4-40ff-9690-ff3da147775b | Freshwater crustaceans in Antarctic and sub-Antarctic lakes | 8 |
| 13a0db74-c9d4-41b2-9100-3bb4621dbd79 | (Tab. 9.B) Presence and abundance of benthic fauna at station FINO 1 in 2005-2007 | 9 |
| 977a916a-f762-11e1-a439-00145eb45e9a | Abundance of benthos infauna at station GIK23071-1 | 1 |
| 62b6831c-f762-11e1-a439-00145eb45e9a | Naturschutzgebiet Kochertgraben | 3 |
| 6fc0903e-f762-11e1-a439-00145eb45e9a | Distribution of meiobenthos in sediment core M23_123 (2) of the northeastern Atlantic (Table 1) | 2 |
| 16c2d561-696a-4704-bfab-0a064e6fbccc | (Tab. 9.D) Presence and abundance of benthic fauna at station FINO 1 in 2005 | 40 |
| 37f48e00-1fe8-11dc-b461-b8a03c50a862 | Dutch Foundation for Applied Water Research (STOWA) - Limnodata Neerlandica | 50521 |
| b019c833-0b1a-4658-a27e-80b46bf4aa06 | Macrofauna, entomofauna y vegetación vascular de la Isla Cayo Serranilla durante la Expedición Seaflower 2017 - Proyecto Colombia BIO | 3 |
| 8a916226-f762-11e1-a439-00145eb45e9a | Wupperaue bei Kemna (Wuppertal) | 2 |
| 7006b5b4-f762-11e1-a439-00145eb45e9a | Meiofauna abundances at the Arctic Håkon Mosby Mud Volcano (HMMV) of sediment core PS64/363-1a | 5 |
| 700cf14a-f762-11e1-a439-00145eb45e9a | Meiofauna abundances at the Arctic Håkon Mosby Mud Volcano (HMMV) of sediment core PS64/395-1 | 5 |
| 7007f3ca-f762-11e1-a439-00145eb45e9a | Meiofauna abundances at the Arctic Håkon Mosby Mud Volcano (HMMV) of sediment core PS64/363-1b | 5 |
| 7001b262-f762-11e1-a439-00145eb45e9a | Meiofauna abundances at the Arctic Håkon Mosby Mud Volcano (HMMV) of sediment core PS64/324-1 | 5 |
| 6ffcb2a8-f762-11e1-a439-00145eb45e9a | Meiofauna abundances at the Arctic Håkon Mosby Mud Volcano (HMMV) of sediment core PS64/313-1 | 5 |
| 6fbb78f6-f762-11e1-a439-00145eb45e9a | Distribution of meiobenthos in sediment core M23_203 of the northeastern Atlantic (Table 1) | 5 |
| 6fa1f8e0-f762-11e1-a439-00145eb45e9a | Distribution of meiobenthos in sediment core M19_197-2 of the northeastern Atlantic (Table 1) | 5 |
| 0c4dc4f2-c11e-4e8b-8169-cabac6384735 | n_niek_2021 | 153 |
| 46c9d9a2-f8f8-48fd-b90c-412fc635dd61 | Meiofaunal abundance in sediments obtained during RRS Discovery cruise D297 | 79 |
| 6fb02e74-f762-11e1-a439-00145eb45e9a | Distribution of meiobenthos in sediment core M19_246-2 of the northeastern Atlantic (Table 1) | 5 |
| f1d8b81b-4c3c-467d-87ea-bad6fe626441 | Tab. 6: Relative Abundanz einzelner Arten der Endofauna | 14 |
| 6faee618-f762-11e1-a439-00145eb45e9a | Distribution of meiobenthos in sediment core M19_239-2 of the northeastern Atlantic (Table 1) | 4 |
| 6fab09e4-f762-11e1-a439-00145eb45e9a | Distribution of meiobenthos in sediment core M19_232-2 of the northeastern Atlantic (Table 1) | 5 |
| 6f9f78e0-f762-11e1-a439-00145eb45e9a | Distribution of meiobenthos in surface sediments of the northeastern Atlantic (Table 1) | 9 |
| 6f82b994-f762-11e1-a439-00145eb45e9a | Meiobenthic assemblages colonizing artificial soft sediments at 20 m depth in Arctic glacial Kongsfjorden, Spitsbergen, Svalbard - individual number | 16 |
| be9bf8ca-92f0-4b72-8504-da2cc753a955 | (Table 2b) Biomass of macrobenthos species in samples from Cruise AMK54 stations in the Novaya Zemlya Trough | 11 |
| 976a7514-f762-11e1-a439-00145eb45e9a | Abundance of benthos infauna at station GIK23024-1 | 3 |
| ce416850-8934-11dc-9962-b8a03c50a862 | Ghent University - Zoology Museum - Insect collection | 3 |
| 97953538-f762-11e1-a439-00145eb45e9a | Benthic meiofauna abundance in surface sediment of core PLG93_BCORE6 | 8 |
| 9796833e-f762-11e1-a439-00145eb45e9a | Benthic meiofauna abundance in surface sediment of core PLG93_BCORE7 | 8 |
| bed01630-5697-4965-94b1-e1d1ebdcbec9 | Australian Institute of Marine Science (AIMS) - CReefs Ningaloo Reef Biodiversity Expedition, Australia (2008 - 2010) | 9 |
| 89a9fe7c-f762-11e1-a439-00145eb45e9a | Zukünftiges NSG Höftland/Bockholmwik | 2 |
| c4a764e4-8455-425b-b553-253f9f9dfbac | Yorkshire Naturalists Union Marine and Coastal Section Records | 36 |
| 976696b0-f762-11e1-a439-00145eb45e9a | Abundance of benthos infauna at station GIK23011-2 | 7 |
| 89a5e170-f762-11e1-a439-00145eb45e9a | GEO Hauptveranstaltung Tirol (Innsbruck) | 1 |
| 9763ea6e-f762-11e1-a439-00145eb45e9a | Abundance of benthos infauna at station GIK23004-3 | 1 |
| 81b2a96c-f762-11e1-a439-00145eb45e9a | Abundance of benthic infauna in surface sediments from the North Sea sampled during HEINCKE cruise HE133 | 7920 |
| 81cb7816-f762-11e1-a439-00145eb45e9a | Abundance of benthic infauna in surface sediments from the North Sea sampled during Friedrich Heincke cruise FHE238 | 465 |
| 89a1c2c0-f762-11e1-a439-00145eb45e9a | Altholzparzelle Eilenriede Hannover | 1 |
| 6b3877d5-4869-43aa-af12-92c703fd5b6d | Marine data from Natural Resources Wales (NRW) Technical Support (Research & Monitoring) Contracts, Wales | 6111 |
| 815fd610-f762-11e1-a439-00145eb45e9a | Abundance of benthic infauna in surface sediments from the North Sea sampled with TYRO in 1986 | 3975 |
| 6d4427fc-932b-4814-b323-ee7980788f3b | Australian River Assessment System | 1115 |
| 879dbd41-ba54-4544-8804-578562e2bda5 | Community structure and seasonal fluctuation of macrobenthos in a Ceriops tagal mangrove sediment at Gazi Bay in 1992 and 1993 | 69 |
| ce1faba4-d8d9-46ac-ba26-cffe03eadc99 | Upper Murrumbidgee Waterwatch | 32 |
| 917f5583-f6ce-4f66-bdfd-e5150dbc5200 | Colección Entomológica del Programa de Biología - Universidad de Caldas (CEBUC) | 151 |
| 9b8b786c-9d18-4a6d-bada-fd4d6eec05f5 | Macroinvertebrados acuáticos presentes en la ciénaga de montaño, Carmen del Darién, 2020 | 16 |
| 94e0c0ec-dd80-4e66-919c-a0d3bd9bfa0f | Zooplankton studies at a fixed station (West-Hinder) in the North Sea between 1977 and 1979 | 27 |
| 7afb6dbd-6485-42c2-aee7-c730bd81d2cd | Zooplankton Species Distribution and Abundance Data, Arctic Marine Biodiversity Observing Network (AMBON) Chukchi Sea research cruise, August 2017 | 237 |
| 3dc26c61-3adf-45d7-876c-6358a37ff3f9 | Macrobenthos North Adriatic-INTERREG-FVG Project | 91 |
| 4ebfcad1-f099-4468-ba23-83c7ad4247e0 | Netplankton North Adriatic-ALPE ADRIA Project | 60 |
| 8a6ac2b0-f762-11e1-a439-00145eb45e9a | Tiergarten Straubing | 1 |
| 09e90dfb-5b1b-4dd9-a796-e2fba53d26f0 | Inventory and BioBlitz Records from rare Charitable Research Reserve | 16 |
| deb97a37-48cc-4b5d-83fe-4f7384769c5e | Biota presente en el humedal laguna La Linda, departamento del Tolima | 10 |
| 789d286b-73b7-4d8c-a8c6-7a2cfe84a576 | Rådgivende Biologer | 94 |
| 7eae038e-7a8e-492e-a4dc-43238cce7c52 | Auburn University Museum of Natural History Mollusks | 10 |
| 62b54d08-f762-11e1-a439-00145eb45e9a | Tauchen und Meer 02 | 1 |
| 89c0ef1a-f762-11e1-a439-00145eb45e9a | Langes Tannen | 1 |
| 89ca51ae-f762-11e1-a439-00145eb45e9a | Gesamtartenliste Bremerhaven, Helgoland und Sylt | 18 |
| d18935d7-260c-4633-af39-7eb7ee6f1e14 | Bibliographie de la faune, la flore et la fonge de France métropolitaine et outre-mer - Bibliographique de la faune, la flore et la fonge de France métropolitaine et outre-mer | 32 |
| 75db885c-65a7-499e-a28a-4206723007d0 | A detailed benthic faunal and introduced marine species survey of Port Davey, Bathurst Channel and Bathurst Harbour in SW Tasmania, Australia (2007) | 152 |
| 4655f1f3-60a5-4346-a343-84f5836a4585 | Auburn University Museum of Natural History Invertebrates | 170 |
| 6197c830-d9c7-11de-b793-b8a03c50a862 | Museo Argentino de Ciencias Naturales "Bernardino Rivadavia" (MACN). Invertebrates National Collection (MACNIn) | 766 |
| 2ce1d8a6-8219-4653-870a-4ea181d4989f | Données Faune Base SIRFF - FNE Centre-Val de Loire - FNE Centre-Val de Loire - Données SIRFF 2018-2020 | 5 |
| 313fd6b1-2c8b-4419-a5dd-dc3176d62abc | Taxonomy and vertical export rates of protist cells, planktonic protist carbon (PPC) and zooplankton abundance and biomass from long-term sediment traps in the shelves north and east of Svalbard, as part of the Nansen Legacy project and the Arctic PRIZE project | 36 |
| 54facf62-bf0c-43e7-9972-4fdc407fa6c5 | Déterminations et observations du Forum "Le Monde des Insectes" (LMDI) - Données photographiques validées de la galerie du forum "Le Monde des Insectes" | 38 |
| 7a877d20-f762-11e1-a439-00145eb45e9a | 2. Aspacher GEO-Tag der Artenvielfalt | 1 |
| 0eb4ad83-f80d-4d86-83d8-f3a55ddd216f | CardObs : Observations naturalistes issues de l'outil de saisie et gestion CardObs mis en place par le Service du Patrimoine Naturel (MNHN) / UMS PatriNat (OFB - CNRS - MNHN) - Données naturalistes de Xavier JAPIOT | 23 |
| 762304f7-def1-4031-af54-547862e1e43d | Elasmopus sivaprakasami sp. nov., a new species of amphipod (Senticaudata, Maeridae) from Gujarat State, India | 1 |
| 25e3e8f1-86ee-440c-adfc-8e63759a6505 | Programme d’acquisition et de valorisation de données naturalistes BioObs - Observations naturalistes des Amis de BioObs. | 198 |
| fdaf857b-4a9d-4721-b60b-9e6d038e81f1 | Mesozooplankton South Adriatic-PRISMA1-Flussi Project | 3 |
| 7a7ebc8a-f762-11e1-a439-00145eb45e9a | Exkursion in der Ehrbachklamm/ an den Erbach | 1 |
| ebedfe50-dd03-4028-9506-7d43f83878cd | Epibenthos and demersal fish monitoring data in function of wind energy development in the Belgian part of the North Sea | 1 |
| 19e7f71d-ad1e-4a03-9a24-983e74fe3809 | Active meso- and meiofauna in the nature reserve "Het Zwin" (Knokke) in 1969 | 5 |
| 790ebca6-f762-11e1-a439-00145eb45e9a | Benthic macrofauna abundance of sediment core 13200-078 | 10 |
| 7b7393c2-f762-11e1-a439-00145eb45e9a | RMT Trawl catch from the 1990/91 V6 AAMBER2 voyage | 136 |
| 88de7dce-f762-11e1-a439-00145eb45e9a | RMT Trawl catch from the 2005/06 V3 BROKE-West voyage - Fish | 74 |
| 5d78d011-4390-4b5f-9aae-958b337a3031 | Macrobenthos of the Western Scheldt (Ossenisse, Valkenisse, Terneuzen and Vlissingen) on 27 and 28 September 1978 | 112 |
| 70006b78-f762-11e1-a439-00145eb45e9a | Meiofauna abundances at the Arctic Håkon Mosby Mud Volcano (HMMV) of sediment core PS64/323-1 | 5 |
| d8f8115d-9d8a-473a-be44-ae7d34f034d8 | The importance of fouling in the port of Ostend in 1964 | 1 |
| 791fbace-f762-11e1-a439-00145eb45e9a | Macrofauna abundance in surface sediment sampled during cruise DI231 | 5 |
| 6d437ff3-5140-4de3-ad97-80016181760a | N3 data of Kiel bay | 201 |
| 97814082-f762-11e1-a439-00145eb45e9a | Abundance of benthos infauna at station POS128/2_281 | 1 |
| 70825b58-e23f-41ce-9de1-1f472278a2fd | Zooplankton monitoring in the Belgian Part of the North Sea between 2009 and 2010 | 165 |
| 97852364-f762-11e1-a439-00145eb45e9a | Abundance of benthos infauna at station PS1240-1 | 4 |
| 6ffb63c6-f762-11e1-a439-00145eb45e9a | Meiofauna abundances at the Arctic Håkon Mosby Mud Volcano (HMMV) of sediment core PS64/312-1 | 5 |
| f67d4560-51fa-4443-9ce3-acced5515eec | Further investigations of the effects of the Nella Dan oil spill 1988/94 | 84 |
| 6fb40972-f762-11e1-a439-00145eb45e9a | Distribution of meiobenthos in sediment core M23_122 of the northeastern Atlantic (Table 1) | 5 |
| 520237b3-b0b3-47b5-90e1-af12098d6128 | Marine Records from Pembrokeshire Marine Species Atlas | 2226 |
| 9b5fd002-17a9-4061-ac4c-39b516e26c9d | Epifauna community at Waarde and Saeftinghe (Westerschelde) in 1991 | 30 |
| 88261147-1a23-4a4d-be95-4eb5173c9b20 | Evaluation of the effect of disposal of dredging material on macrobenthos communities in the Maas plain (1988) | 19 |
| a79c2b50-6c8a-11de-8226-b8a03c50a862 | Queensland Museum provider for OZCAM | 1414 |
| e1e5921c-5d04-4015-9943-137790fc3bc0 | Spatial distribution of the macrozoobenthos on the 'Slikken van Vianen' (Oosterschelde) in 1979 | 57 |
| 4a1fb401-3186-4e60-bbd4-f61de71a0c76 | Zooplankton sampling in the coastal waters of south eastern Tasmania, Australia (2009-2015) | 13 |
| 217a1e61-33a1-471e-bb05-76cd2e22cb06 | Données LPO Réserves Naturelles Nationales - Données des réserves naturelles nationales co-gérées par la Ligue pour la protection des oiseaux (LPO) et l'Office français de la biodiversité (OFB) | 593 |
| a03680a2-dc7d-4fc1-9f43-0535b0d8fcd6 | Zooplankton counts measured at the West-Hinder in 1902 | 14 |
| 36e71fad-810e-4eb2-8f4f-fa7eda4ed5c6 | REDIT 1 | 798 |
| 63fce98c-f762-11e1-a439-00145eb45e9a | Naturschutzgebiet Börstig bei Hallstadt | 1 |
| 53334055-d64e-48cd-8c83-2295d3891782 | Resilience of Antarctic marine benthic invertebrates and the ecological consequences of environmental change - Amphipod Data 2002/10 | 123 |
| 1033d90e-c10c-4ce8-87d0-0f8227300765 | Deep-sea meiobenthos at Calvi (Corsica) sampled in September 1982 | 1 |
| 8983b7c6-f762-11e1-a439-00145eb45e9a | AKG-Gelände (Bensheim) | 1 |
| 5f9dc2e9-3952-4341-b46d-cf4e4d002f3a | Seasonal variation of the zooplankton community at Gazi, Lamu and Malindi (Kenya) sampled between 1990 and 1992 | 1 |
| f7924a95-abc6-4b20-b365-1a9f8a4bbf82 | New species of Victoriopisa Karaman & Barnard, 1979 (Crustacea: Amphipoda: Eriopisidae) from Vembanad backwaters, Southwest coast of India | 2 |
| 4f546e51-1449-4f5a-a927-3c6fdc505b9b | Meiofauna abundances in sediments from the Storegga Slide (Norwegian Sea) obtained during the VICKING expedition in 2006 | 4 |
| b5e4bc1b-ec14-4ff1-8eef-9c15ab6a7b75 | Polish Arctic Marine Programme | 75 |
| 69b14a72-b13a-472f-81d4-fb1b184d9462 | Recolonization of a disturbed sandsediment by macrobenthos in the Sluice dock (Oostende) in 1980 | 7 |
| 851602bd-a9eb-48c0-9f3b-1fa13babbd85 | Meiofauna abundances in sediments from the Nyegga pockmark area (Norwegian Sea) obtained during the VICKING expedition in 2006 | 6 |
| c063bd29-d63b-4ff5-bb79-adb1a4533b1a | COLETA - IMAR/DOP-Uac reference collection from 1977 to 2012 | 29 |
| 7128853a-f762-11e1-a439-00145eb45e9a | Macro benthos in surface sediments sampled during POLARSTERN cruise ANT-XXIII/8 | 319 |
| 9a546219-a51e-446d-8ed9-b356df938fed | Data collected during the expeditions of the e-learning projects Expedition Zeeleeuw and Planet Ocean | 7 |
| b12ed28a-f3db-4cbb-a975-1619272a74f9 | (Table 2a) Abundance of macrobenthos species in samples from Cruise AMK54 stations in the Novaya Zemlya Trough | 24 |
| 9758adf3-77f1-464b-b6ab-79e6d605e297 | Analysis of the macrobenthic community near Nieuwpoort (1970-1971) | 27 |
| 7d95137d-4dbe-41ff-9569-a890c84abd6b | Saisie naturaliste opportuniste dans SICEN Occitanie - Données opportunistes du CEN Languedoc-Roussillon | 2 |
| 78c3c4ee-f762-11e1-a439-00145eb45e9a | Matter enrichment of the macrofauna community of MAC station 13077-001 | 16 |
| e59a285e-4afc-4b62-894f-fb40d9e63b07 | Occurrence of zoobenthos organisms in the sampled Central-Yakutian Lakes, Appendix 2-4 | 8 |
| 6c827cf4-5d22-454e-98b1-4acbf0cb5aca | niek_nes_22 | 164 |
| 0222cdf0-aec1-4e02-921f-383841cca268 | Biota presente en el humedal Turbera de Alfombrales, departamento del Tolima | 4 |
| 68513375-3aa5-4f6f-9975-d97d56c21d61 | Illinois Natural History Survey Insect Collection | 27 |
| cee6464f-cae2-4aab-aa8b-0429a2cb3af0 | Programme Ecoscope: données d'observations des écosystèmes marins exploités (Réunion) | 1 |
| 7b4322d2-4415-4273-86ef-1b8317db089b | Biota presente en el humedal laguna El Meridiano, departamento del Tolima | 1 |
| 415860c4-137b-4617-9d10-a51885e35491 | Rifle sediment bacterial community Targeted Locus (Loci) | 1 |
| 921ae6e9-d890-4826-b6ff-aef43cdcb409 | uncultured prokaryote Targeted loci environmental | 1 |
| 59bd5714-f805-495e-9378-20cd488f6086 | Antarctic and New Zealand Byblisoides (Amphipoda: Ampeliscidae) with a key to the world species | 2 |
| c5ee2810-2f42-44ce-8b75-bfe6944074a0 | Alien aquatic invertebrates of Republic of Karelia: Baikalian Amphipoda (Gmelinoides fasciata (Stebbing, 1899) | 26 |
| 037e41e2-2ccf-4655-96d6-1b1949c4f1e6 | Description of Orthoprotella bicornis, new species, and Paraprotella teluksuang, new species (Crustacea: Amphipoda) from Johor, Malaysia with special reference to unusual sexual bias towards females in Paraprotella | 5 |
| 22bc7ea9-a5a5-4faa-936f-d1d6215b1bb5 | Meiobenthos of target sites of Crimea in 2009/2010 | 22 |
| 9631e56a-f762-11e1-a439-00145eb45e9a | COMARGIS: Information System on Continental Margin Ecosystems | 676 |
| 96309e26-f762-11e1-a439-00145eb45e9a | Ifremer BIOCEAN database (Deep Sea Benthic Fauna) | 381 |
| 81b8c87e-f762-11e1-a439-00145eb45e9a | Abundance of benthic infauna in surface sediments from the North Sea sampled during two Michael Sars cruises in 2000 | 73 |
| 81b3deb8-f762-11e1-a439-00145eb45e9a | Abundance of benthic infauna in surface sediments from the North Sea sampled during cruise Dana00/5 | 57 |
| 78ffcf34-f762-11e1-a439-00145eb45e9a | Benthic macrofauna abundance of sediment core 13200-017 | 10 |
| 78ded5cc-f762-11e1-a439-00145eb45e9a | Benthic macrofauna abundance of sediment core 12930-063 | 14 |
| 78d9e724-f762-11e1-a439-00145eb45e9a | Benthic macrofauna abundance of sediment core 12930-048 | 14 |
| 812edb01-f542-49d3-93fc-641b08125df5 | Mesozooplankton North Adriatic-gulf of Trieste C1-LTER time-series | 5 |
| 78e181be-f762-11e1-a439-00145eb45e9a | Benthic macrofauna abundance of sediment core 12930-068 | 14 |
| 13edff6d-715f-48b3-8c10-9f3547dcf061 | Two new species of eyeless amphipods from a coastal area in Japan (Crustacea: Amphipoda: Hadziidae, Melitidae), with reinstatement of the genus Paraniphargus Tattersall, 1925 | 6 |
| 1f624c93-2d62-44b0-8867-ae3d15ee5239 | Ampithoidae (Crustacea: Amphipoda) from the Persian Gulf and the Gulf of Oman | 8 |
| 4dcfa4f3-7ebe-47d6-bd93-a85315c9f180 | Sbp-BioBlitz2017_60years-BiodiversityData | 2 |
| 34877684-bbd1-45f2-a205-8f1cc9ad58d7 | Soft sediment assemblages at Casey Station: Brown Bay Grid - samples taken along a pollution gradient. | 62 |
| 42f293d6-1b3e-4ca0-9d70-a7d1dcc9a209 | Macro-invertebrados de la cuenca alta y media del río Bita, Vichada (Colombia) | 1 |
| ab0a9802-739c-40b2-b5af-5d41f42f5619 | Red Sea Leucothoidae (Crustacea: Amphipoda) including new and re-described species | 1 |
| 0a07a36b-37fb-4f0c-883e-924aa79acc6d | A new genus and species, Panamapisa guaymii gen. nov. sp. nov., the first record of the family Eriopisidae Lowry & Myers, 2013 from Central America | 5 |
| 38660b46-e5cf-4386-8553-96f8d60bce25 | Two species of Talitridae (Crustacea, Amphipoda) from Kenya | 4 |
| a050bfe3-8e9a-4d0e-ac7b-27be216aeb66 | LIGNE NOUVELLE MONTPELLIER PERPIGNAN DUP PHASE 1 - Crustacé aquatique d'eau douce - Inventaire non protocolé | 9 |
| 9a23e0ce-ffdb-4ec1-9796-403ffb9ada90 | A new deepwater species of Calliopiidae, Halirages helgae (Crustacea, Amphipoda), with a synoptic table to Halirages species from the northeast Atlantic | 1 |
| 71bc8e1a-0dbd-4a5f-8b3c-ef278252f6bb | Porcupine Marine Natural History Society Records | 15 |
| 9d7baaac-57db-4852-9993-7f0e7f15635b | Metabarcoding Data from an Inventory of Freshwater Invertebrates from the Miller Creek Watershed, Kenai Peninsula, Alaska, USA | 32 |
| 8442816f-cf5f-4c21-9d3d-5c368b4006d8 | Waterbug Census | 65 |
| f9a70dab-004b-45ad-90cb-24d8ff645b44 | Centre for Biodiversity Genomics - Canadian Specimens | 1688 |
| c83a2438-0703-47fa-8b95-17d84e8c762f | CSIRO, Cruise SS200001, Marine Biodiversity, South and Southeast Australia, 2001 | 1 |
| 872065ec-6593-4eb8-8adb-fb3b73fdece3 | RACCORDEMENT ELECTRIQUE DE LA FERME EOLIENNE FLOTTANTE DE GROIX ET BELLE-ILE - Création de la liaison sous-marine et souterraine à 63 000 volts - Campagne benthos Rocheux2015-Raccordement du parc éolien Groix/Belle-île | 21 |
| 295eaad6-eae5-4b90-8d41-278a3a9e6645 | Biota presente en el humedal Lagunas Las Mellizas, departamento del Tolima | 3 |
| 6d1be689-76c7-4124-9f5e-e57d3f93613e | Biological records from the U.S Antarctic Service Expedition, 1939-41 | 11 |
| 8e2961c9-5996-474b-9bfc-90370e04448b | Ecological surveys from Bardon Hill undertaken in the period 2000 to 2007 | 4 |
| 8a718492-f762-11e1-a439-00145eb45e9a | Werremündung im Schwarzatal | 1 |
| 2a2bf4d1-5747-4c03-ae7b-328a7768345b | Stable isotope data from marine sediment and invertebrates from Davis Station 2009/10 | 16 |
| 8a943492-f762-11e1-a439-00145eb45e9a | An der Ohm, (Wettsaasen) | 1 |
| ee8e66ff-2f2c-47e2-978b-be52996d5b0f | Jassa (Crustacea: Amphipoda): a new morphological and molecular assessment of the genus | 7 |
| 56a946bf-bd42-4a54-8543-4380d92f9762 | INNS Data: All Taxa (West Wales) | 38 |
| bcfc6dcd-587a-4f56-9ffd-7c846804c627 | Redescription of Sternomoera moneronensis (Crustacea: Amphipoda: Gammaridea) from Moneron Island, the Russian Far East | 8 |
| d34a52f7-b652-4321-98ce-822545a9f24e | Four Species of Dulichiidae (Crustacea: Amphipoda) from Japan, with the Description of a New Genus and Two New Species | 18 |
| 8645653c-f762-11e1-a439-00145eb45e9a | RMT Trawl catch from 1985/86 V1 ADBEX III voyage | 60 |
| 8a985fea-f762-11e1-a439-00145eb45e9a | Artenvielfalt der Nordsee - Sylt | 2 |
| 58b7e7e3-48e0-40c3-9b7c-7f48fbee23c0 | Benthos of Iranian parts of the Caspian Sea | 127 |
| 2ce65268-cf51-4ecb-bb36-ce615564c255 | PondNet data 2012-2014 | 9 |
| c6951371-fbed-41b6-a1dc-dbbd1d2723d9 | 2004-2012 Bishop et al. Occurrence of non-native sessile invertebrates on the English coast | 23 |
| cc58a5e4-a757-41f7-b1cc-5ce591119965 | The first Arctic conspicuously coloured Pleusymtes (Crustacea: Amphipoda: Pleustidae) associated with sea anemones in the Barents Sea | 4 |
| 9797cd98-f762-11e1-a439-00145eb45e9a | Benthic meiofauna abundance in surface sediment during cruise CD86 | 1 |
| fae37d1f-5b7a-4fe2-8cfa-8948aefbcac2 | Natural England Marine Monitoring surveys | 13052 |
| 793c3890-6c8a-11de-8226-b8a03c50a862 | Northern Territory Museum and Art Gallery provider for OZCAM | 1167 |
| 96a2b704-f762-11e1-a439-00145eb45e9a | Fish catch from 1996/97 Voyage 1 WASTE (WOCE Antarctic Southern Transect Expedition) | 3 |
| 93efc16a-7eb1-4792-bdec-de1ea69b8c65 | DFO Quebec Region MLI museum collection | 1298 |
| 8ab49566-f762-11e1-a439-00145eb45e9a | VSN-Wiese | 1 |
| 57b44b1c-d9c9-40fb-89db-04f30f091f88 | Stable Isotope Compositions of tubeworms, mussels, and their associated fauna in Gulf of Mexico hydrocarbon seeps | 15 |
| 6affc398-dfcb-4ac1-86a1-d8b9566e2dac | CSIRO, Cruise SS200702, Marine Biodiversity Survey, Southeast Australia, 2007 | 21 |
| 8ab8d9c8-f762-11e1-a439-00145eb45e9a | Tag der Artenvielfalt | 3 |
| 7d08feb6-f762-11e1-a439-00145eb45e9a | Survey of benthic and other marine invertebrates of Prydz Bay, 1990/91 Voyage 6 | 113 |
| 6da969ce-0833-4bb6-a4e6-5013a50eefef | Nonindigenous Aquatic Species (NAS) Database Non-freshwater Specimens | 955 |
| c1fc2df7-223b-4472-8998-70afb3b749ab | European Molecular Biology Laboratory Australian Mirror | 49 |
| ecc8be51-7a2b-4723-9fd8-62fd542fd01a | Groundwater amphipods of the genus Niphargus Schiødte, 1834 in Boyer-Ahmad region (Iran) with description of two new species | 4 |
| 91aa3d5b-6f77-4135-a823-cef438a60dfa | Référentiel taxonomique national (TAXREF) - Inventaire des Collectivités d'outre-mer et de la métropole (ICOM_15) issu des données biogéographiques de TAXREF v15 | 233 |
| 592d585d-6186-4093-8375-77ad80da7c9a | Carnarvon Basin (Aquatic Projects) - Invertebrates | 18 |
| 322ec841-f604-4024-ab49-897dc47f74c3 | Port Phillip Bay Environmental Study Data 1992-1996 - benthic records | 1711 |
| ea9f08a6-9c19-4101-99c6-1e6428c78277 | RNF \| Ichtyofaune Prés salés, socle commun (Espaces protégés de la LPO France et de l'OFB) - RNF Prés salés - Biométrie de l'ichtyofaune relâchée \| RNN Moëze-Oléron | 4 |
| 81b78f9a-f762-11e1-a439-00145eb45e9a | Abundance of benthic infauna in surface sediments from the North Sea sampled during cruise Tridens00/5 | 960 |
| 81b52746-f762-11e1-a439-00145eb45e9a | Abundance of benthic infauna in surface sediments from the North Sea sampled during cruise Cirolana00/5 | 276 |
| a983a1df-ed19-4fe8-9d1e-47fa9c29dd56 | Benthic Epifauna Biomass and Abundance Data, Arctic Marine Biodiversity Observing Network (AMBON) research cruise on the Norseman II, 9 August 2015 - 3 September 2015 | 548 |
| 9f9cf852-babb-4c52-b17f-f81dbe96c527 | Benthic Epifauna Biomass and Abundance Data, Arctic Marine Biodiversity Observing Network (AMBON) research cruise, August 2017 | 209 |
| 07a203b7-21a7-4c01-b8a4-cf906f4bc1c0 | 1778-1998 Ivor Rees North Wales Marine Fauna Ad-hoc sightings shore and ship-based surveys | 402 |
| a59435cf-8cb7-4c09-917d-937c156f054e | Invertebrates of the Recherche Archipelago , Western Australia (2002) | 39 |
| a307e4d7-1de2-4adc-95d5-a0a8d5f57236 | Natural History Museum Rotterdam - Specimens | 30 |
| 775ec344-b8d4-41ed-a087-ee2d0637faba | IOV-UDO-ZOOPLANCTON-PLAYA COLORADA | 5 |
| c2d59cb8-6001-48c9-b41d-96af31028066 | Pelagic amphipods (Crustacea: Amphipoda: Hyperiidea) in western Mexico. 2 Family Eupronoidae | 17 |
| d75a7bfa-4559-4567-b205-dd08e7dce524 | Floresorchestia kongsemae sp. n. a new species (Crustacea: Amphipoda: Talitridae) from Kasetsart University, Bangkok, Thailand | 3 |
| 92580ab9-28a5-490f-9a6a-a5fa45449332 | The Australian Zooplankton Database (1938 onwards) | 8 |
| 978fee52-f762-11e1-a439-00145eb45e9a | Benthic meiofauna abundance in surface sediment of core PLG93_BCORE2 | 9 |
| 40c0f670-ee87-4576-be9d-2725e0b47035 | SA Fauna (BDBSA) | 652 |
| 16b0aa56-90aa-436c-bfe8-b5af83f84575 | Biodiversity Research and Teaching Collections - TCWC Marine Invertebrates | 315 |
| a1e3d691-c4d9-4fdd-a52c-d6f377f57769 | Classic localities of aquatic Arthropoda from the Western Balkans | 22 |
| f48a5a24-f17c-4a56-a86c-48618fc6b631 | Zooplankton NOGAP32b 1986 | 1106 |
| fa375330-6c8a-11de-8226-b8a03c50a862 | South Australian Museum Adelaide provider for OZCAM | 1857 |
| b54efda7-4ae3-4c67-9b89-c7b699dffb19 | Onisimus turgidus (Sars, 1879) (Amphipoda, Uristidae), an overlooked amphipod from sea anemones in Northern Norway | 3 |
| 97929cf6-f762-11e1-a439-00145eb45e9a | Benthic meiofauna abundance in surface sediment of core PLG93_BCORE4 | 8 |
| b6dc7422-a09d-4986-b5ac-e8f6f50a9732 | Biodiversity weekend observations, Recorder-Lux database | 3 |
| 27637276-6ce8-4b6c-8688-36d04bb47616 | A new species of Ledoyerella, L. kunensis sp. nov. (Amphipoda, Senticaudata, Kamakidae) from South West Africa | 3 |
| 25a1d6a3-0aac-46f0-b73c-c69451b5f906 | The Amphipoda (Crustacea) of New Caledonia: Aoridae | 3 |
| 3272d7a6-47c3-416c-9a02-5b18c553f132 | New lysianassoid genera and species from south-eastern Australia (Crustacea: Amphipoda) | 9 |
| 86b50d88-f762-11e1-a439-00145eb45e9a | MAL | 2 |
| 6205a718-f762-11e1-a439-00145eb45e9a | Umgebung des Spalatin Gymnasium Altenburg | 1 |
| 7f1f6064-f762-11e1-a439-00145eb45e9a | Abundance of macrobenthos organisms in the northern Wadden Sea in 2008 | 36 |
| 7d4b3c40-f762-11e1-a439-00145eb45e9a | Crustacea in surface sediments off Sylt collected during HEINCKE cruise HE255 | 14256 |
| 09035b62-5cf1-4ac9-8632-2eb9c46695a5 | RNF \| Ichtyofaune Prés salés, socle commun (Espaces protégés de la LPO France et de l'OFB) - RNF Prés salés - Biométrie de l'ichtyofaune relâchée \| RNN Baie de l'Aiguillon | 1 |
| be212283-072e-4e50-b938-689e3c82f5c6 | Yorkshire Wildlife Trust Shoresearch | 14 |
| 7f1e1e7a-f762-11e1-a439-00145eb45e9a | Abundance of macrobenthos organisms in the northern Wadden Sea in 2007 | 36 |
| 61727cb8-f762-11e1-a439-00145eb45e9a | Lebensraum Fluß/Zwickauer Mulde in Wolkenburg | 1 |
| c9759371-0592-4121-8649-fabded2229f5 | Caracterización de la Artropofauna terrestre presente en islotes de Acandí y Nuquí, Chocó. 2021 | 2 |
| ee5f5e73-28e3-4018-abc9-12dbf3a07553 | SEWBReC Myriapods, Isopods, and allied species (South East Wales) | 691 |
| edd017b6-bf00-4b00-93ad-9789c42a36b1 | Species data for Scottish waters held and managed by Scottish Natural Heritage, derived from benthic surveys 1993 to 2018 | 7199 |
| e2413993-3e6c-4519-abe5-02b75cb645c6 | Invertebrate Common Standards Monitoring and ISIS Test Data | 84 |
| efb3f1a0-43d5-4ae6-a58f-4fd61043756a | National Trust for Scotland Species Records | 24 |
| 956bc674-6022-4c52-833e-c5fb39dc837a | Löydös Open Finnish Observation Database | 3 |
| d7d23b73-9119-494d-b3ca-40da6f7bd3ed | Fauna presente en el humedal Albania, departamento del Tolima | 1 |
| 6a3aa184-0a82-41f1-bdef-dfb9eea5310e | Fauna presente en el humedal La Huaca, departamento del Tolima | 3 |
| 9773cd8a-f762-11e1-a439-00145eb45e9a | Abundance of benthos infauna at station GIK23046-1 | 2 |
| d7585361-2860-4a6a-aa05-f3abb4f7dde9 | Numérisation des données faune contenues dans les Bulletins de la Société Linnéenne de Bordeaux - Bulletin de la Société Linnéenne de Bordeaux, Tome 136 (N.S) n° 29 (1), 2001 - Données faune | 2 |
| 3c02253e-da74-49dd-b059-c693727406fd | Wetland Invertebrate and Environmental Data 2010 Sampling Events | 243 |
| 9c240935-a231-4067-a7ff-e171761c90dd | Macroinvertebrate abundance data from the littoral zone of a number of large New Zealand lakes | 4 |
| 472206fd-12be-4beb-84fe-0b9b971627db | Central West - NSW Waterwatch | 2 |
| 52422715-c1db-40a0-ba16-b84a295d248a | Seasearch Marine Surveys in England | 1557 |
| d38d6841-de2a-4a16-b39e-495402d8592b | Observations faune et flore du Parc national des Écrins | 3 |
| a07c2783-b8c4-41bf-805c-225e9ee610c6 | RNF \| Habitats benthiques intertidaux, volet Taxons, des espaces protégés de la LPO France et de l'OFB - RNF Benthos - Relevés taxonomiques \| RNN Lilleau-des-Niges | 345 |
| fd9b91c7-0788-4c8d-b43d-84953f510663 | RNF \| Habitats benthiques intertidaux, volet Taxons, des espaces protégés de la LPO France et de l'OFB - RNF Benthos - Relevés taxonomiques \| RNN Moëze-Oléron | 186 |
| e5d462f6-054a-4c6d-a9c2-568e275a5f05 | Zooplankton of Morrocoy National Park 2000-2002 | 27 |
| 36449c1f-679d-4235-b34e-1c275ebcd968 | Benthic Biodiversity Baseline Survey, Block 58, Suriname - 2021 - TotalEnergies E&P Suriname B.V. | 1527 |
| 78faa982-f762-11e1-a439-00145eb45e9a | Benthic macrofauna abundance of sediment core 13078-017 | 14 |
| 7919b8e0-f762-11e1-a439-00145eb45e9a | Benthic macrofauna abundance of subcores of sediment core 13200-073 | 52 |
| 791cd2dc-f762-11e1-a439-00145eb45e9a | Macrofauna abundance in surface sediment sampled during cruise DI226 | 5 |
| 78d40e58-f762-11e1-a439-00145eb45e9a | Benthic macrofauna abundance of sediment core 12930-022 | 14 |
| 78d83d70-f762-11e1-a439-00145eb45e9a | Benthic macrofauna abundance of sediment core 12930-044 | 14 |
| 78d595b6-f762-11e1-a439-00145eb45e9a | Benthic macrofauna abundance of sediment core 12930-028 | 14 |
| 17cc3e70-8dcf-4c8b-8059-a5df17610afb | First record of the suborder Colomastigidea (Amphipoda) from Brazilian waters, with description of a new species of Colomastix Grube, 1861 | 6 |
| 67fabcac-a638-40a6-9bea-aeca8aced9f1 | The Danish Environmental Portal, species and habitats-database "Danmarks Miljøportals Naturdatabase" | 580 |
| 18d0270e-899b-4a16-82f2-aa87d93bb1df | Cawthron Institute freshwater invertebrate data | 1080 |
| 8c535464-4c81-4417-a032-1d817fb352ae | National indicator data for river condition in New Zealand | 4007 |
| 7ba8437e-f762-11e1-a439-00145eb45e9a | Landwehrbach | 1 |
| 590e6ac1-bd84-4bac-9d7b-6478be745a4b | Diversity and distribution of North Atlantic Lepechinellidae (Amphipoda: Crustacea) | 16 |
| 7b84c0a2-f762-11e1-a439-00145eb45e9a | Collection Crustacea - ZMB | 2394 |
| 8295f33e-f762-11e1-a439-00145eb45e9a | Collection Crustacea - SNSD | 38 |
| 6197ea48-f762-11e1-a439-00145eb45e9a | Dellwiger Bach (Dortmund) | 1 |
| 5a6977c1-ece6-44b8-b71d-43cb1d4e0919 | DMNS Marine Invertebrate Collection (Arctos) | 1 |
| eb36edcc-0fad-478f-bceb-d2c99526f827 | Programmes de connaissance sur les zones humides d'Occitanie - Données zones humides CEN M-P | 2 |
| 816fbbb6-f762-11e1-a439-00145eb45e9a | (Table 1) Fauna composition of the community of Amphiodia urtica - Nephtys ferruginea in bottom sediments from the central part of the shelf in the California upwelling zone | 5 |
| 96866224-1d21-4007-98a5-d36259e80e90 | mabik_al | 1 |
| 979a5dce-f762-11e1-a439-00145eb45e9a | Benthic macrofauna abundance and biomass in surface sediment during cruise PLG93 | 6 |
| 70790a0d-6cb3-4127-9bf9-8e8c55a1e1b9 | Macroinvertebrados y perifiton pertenecientes a cuatro regiones de Colombia | 7 |
| e3e48696-f083-4c3a-8c5b-efb1589c54db | Atlas of Life in the Coastal Wilderness | 7 |
| 8956ea59-442e-43b9-aeee-7aaef84a87f4 | Mesopelagic Crustaceans of the North Western Portuguese Coast between 1998 and 2000 | 41 |
| 64612a0a-f762-11e1-a439-00145eb45e9a | FFH-Gebiet Paartal | 1 |
| 645fdc04-f762-11e1-a439-00145eb45e9a | Unter hellen Zinnen und finsteren Grotten | 1 |
| cc380a38-100a-408f-84c3-864de932d52c | Bob Merritt dataset of Nottinghamshire invertebrates | 53 |
| 7a2530fc-f762-11e1-a439-00145eb45e9a | RMT Trawl catch from the 2005/06 V3 BROKE-West voyage - Zooplankton | 13 |
| ee0321a6-9e6d-4501-94aa-f12cef9c5680 | David Dodds Associates Ltd - Species Records | 2 |
| 8dcd9007-4e5b-4319-928e-8fb72d628361 | Riverina - NSW Waterwatch | 3 |
| 1af83152-24f7-4df7-afbc-b213b62175bb | Estonian University of Life Sciences Institute of Agricultural and Environmental Sciences Entomological Collection | 5 |
| 61e67a8c-f762-11e1-a439-00145eb45e9a | Wirbach-Taubental (Bad Blankenburg) | 1 |
| 7723cb57-f3e8-4ee2-b188-d95b8d34983e | Two new species of the genus Microlysias (Crustacea, Amphipoda, Tryphosidae) from Korean Waters | 5 |
| e142db13-5b0e-4f15-b652-6e72537b4337 | Marine Nature Conservation Review (MNCR) and associated benthic marine data held and managed by English Nature | 1365 |
| 594e1181-c38a-4f44-b78c-ceab50351cca | National Trust Species Records | 367 |
| 6b924aa4-a926-4ea6-93d5-cec6de5223c8 | RNF \| Habitats benthiques intertidaux, volet Taxons, des espaces protégés de la LPO France et de l'OFB - RNF Benthos - Relevés taxonomiques \| RNN Baie de l'Aiguillon | 4 |
| b56e76a0-2adb-4168-8d46-156a4fe7f10e | Environment Agency England and Wales (2012) Non-native Species records v1 | 25733 |
| 7a498826-f762-11e1-a439-00145eb45e9a | Museu de Ciències Naturals de Barcelona: MCNB-Art | 5 |
| 1a0de42c-8a6c-4ed4-b96a-6527ff14cdfa | Temporal evolution of zooplankton, surface observations, in the Northwestern Mediterranean Sea, Villefranche-sur-mer 1898-1917 | 107 |
| 2401cd5e-26a8-4a0c-9007-e5e6137a1364 | Peracarida of Bernardo O’Higgins National Park (S Chile) | 115 |
| 4b0a57d0-1d9a-462e-aaf5-2a727081a207 | Port Phillip Bay Environmental Study Data 1992-1996 - infauna records | 1597 |
| c149ff8f-ef93-45ce-961c-ea50795fafb3 | Temporal evolution of zooplankton by WP2 net in the Northwestern Mediterranean Sea, Villefranche-sur-mer 2004-2010. | 17 |
| bfb9c2d2-4f05-43f3-b7fd-16b497467178 | FBIP: Offshore Benthic Macrofauna Data | 321 |
| a0a72065-2cd9-4542-9fdf-58533734d5ed | Temporal evolution of zooplankton by Regent net in the Northwestern Mediterranean Sea,Villefranche-sur-mer, 1959-2010. | 53 |
| 6fbcbf68-f762-11e1-a439-00145eb45e9a | Distribution of meiobenthos in sediment core M19_216-2 (2) of the northeastern Atlantic (Table 1) | 4 |
| d091aba1-29e0-4a70-aab9-4ed0523f3a1f | Registro de macrofauna bentónicas submareales de fondos blandos, variables físico-químicos en la zona sur – Registro 5 | 869 |
| 6fa6f71e-f762-11e1-a439-00145eb45e9a | Distribution of meiobenthos in sediment core M19_217-2 of the northeastern Atlantic (Table 1) | 3 |
| 6fac4e30-f762-11e1-a439-00145eb45e9a | Distribution of meiobenthos in sediment core M19_234-2 of the northeastern Atlantic (Table 1) | 2 |
| 16c3a9d0-89ca-48dc-9a43-cdd80a3c355a | Estudio Diagnóstico Levantamiento Biodiversidad Región Arica y Parinacota 2013 | 1 |
| e4096595-7ff5-4b9a-8953-56c2e0fa3076 | Registro de macrofauna bentónicas submareales de fondos blandos variables físicas y químicos en la zona sur – Registro 4 | 120 |
| 6ffdee0c-f762-11e1-a439-00145eb45e9a | Meiofauna abundances at the Arctic Håkon Mosby Mud Volcano (HMMV) of sediment core PS64/314-1 | 5 |
| 700a6f06-f762-11e1-a439-00145eb45e9a | Meiofauna abundances at the Arctic Håkon Mosby Mud Volcano (HMMV) of sediment core PS64/390-1a | 5 |
| f9e48836-88b1-47d3-bb82-7a62ea22dd7d | HBRG Highland Seashore Project Dataset | 2 |
| be6d107e-7f86-45b2-bb3d-cd2a94452e7b | Registro de macrofauna bentónicas submareales de fondos blandos, índices biológicos (AMBI), variables físicas y químicos en la zona sur – Registro 6 | 204 |
| c7799856-9d08-4c02-a2ac-8ac97242f752 | IBSA Surveys from Bennelongia Environmental Consultants | 162 |
| 7fd1bebe-942f-4cb3-ae10-8a29648e47cd | Central Tablelands - NSW Waterwatch | 4 |
| c2d8bc68-8f4d-465e-80b2-734a4bb9d806 | Base de datos de la Sala de Colecciones Biológicas de la Universidad Católica del Norte (SCBUCN) Chile | 54 |
| eac12db6-6a11-4e28-ad4b-b6d752c5a58c | Registro de macrofauna bentónicas submareales de fondos blandos, variables físicas y químicos en la zona sur – Registro 1 | 15 |
| 9c42048d-db20-4a13-9748-71f103227c7d | Manning & Great Lakes - NSW Waterwatch | 9 |
| 628e7ff2-f762-11e1-a439-00145eb45e9a | Riedkanal Bötzingen | 2 |
| 00c913d4-7148-46b3-9aa6-b2a158fa721b | Invertebrate data from Selected Grazing Marshes | 19 |
| 6388f283-7569-40c8-8e94-7e449fe0e966 | Irish Lagoon Surveys 2016 - 2017 | 141 |
| 339d7931-baca-4660-b650-93836399567f | SNH Invertebrate Site Condition Monitoring of Invertebrate Assemblages at ten SSSIs, 2011-12 | 3 |
| 80e91c3c-f762-11e1-a439-00145eb45e9a | Crustacea specimens of Kuroshio Biological Research Foundation | 23 |
| 62897048-f762-11e1-a439-00145eb45e9a | Bach | 1 |
| 628c0696-f762-11e1-a439-00145eb45e9a | Römertal (Steinpleis) | 1 |
| 48c9e50e-5fe5-443f-995f-a8f3bcc24fa6 | Suivi de travaux : Vannes de l’Estrabeau (Engranne) Moulin des 4 ponts (Gestas) - Inventaire macroinvertébrés | 19 |
| 8bb31819-cdb1-44b1-b3e8-6315e011c3ce | Macro-invertebrates of the Desna river basin | 18 |
| 632b49ae-f762-11e1-a439-00145eb45e9a | Kohlbach (Sulzfeld) | 1 |
| b95c5c6e-f65f-49f8-944d-de4fcef25577 | Macroinvertebrados presentes en la Bahía de Tumaco iiap 2018 | 4 |
| 1f4cc518-5213-43d8-ba98-ee53e5271d9b | Three new species of Hyalella (Crustacea: Amphipoda: Hyalellidae) from the Southern Brazilian Coastal Plain | 4 |
| 638110a0-f762-11e1-a439-00145eb45e9a | Biosphärenpark Wienerwald - Wiener Steinhofgründe | 1 |
| eb509ab6-3bd8-479b-a255-46ec514fe6af | Museu Paraense Emílio Goeldi - Carcinológica Collection | 4 |
| 8226ec75-9bba-4351-a101-79306a7dcd55 | Avon Baselining - Invertebrates | 9 |
| 32c50ac2-bfd6-462c-a8ca-4806b75907d0 | Pelagic amphipods (Crustacea: Amphipoda: Hyperiidea) in western Mexico. 3 Family Lestrigonidae | 112 |
| ba9eadc3-7247-4841-8272-e7af87754011 | Review of amphipods of the family Pleustidae Buchholz, 1874 (Crustacea Amphipoda) from the coastal waters of Sakhalin Island (Far East of Russia). I. Subfamily Neopleustinae Bousfield & Hendrycks, 1994 | 14 |
| 7201065d-95ac-4706-bb2b-9fd04209fc8d | Marine Invertebrata specimen database of Osaka Museum of Natutal History | 97 |
| 59df094c-e61d-4cc4-a779-970fab936dba | CSIRO, Cruise SS199701, Marine Biological Survey, South Tasmania, Southeast Australia, 1997 | 45 |
| 2985efd1-45b1-46de-b6db-0465d2834a5a | Tasmanian Natural Values Atlas | 1369 |
| 713c2900-f762-11e1-a439-00145eb45e9a | GEO-Hauptveranstaltung in "Wildtierland" | 2 |
| 82cf41bc-5aa5-4c9c-b86f-845bf591d002 | The pre-winter 2007 vertical distribution of zooplankton in the Cape Bathurst and North Water polynyas, and Lancaster Sound, Canadian Arctic | 29 |
| 713d9d94-f762-11e1-a439-00145eb45e9a | Sahrbachtal Kreis Ahrweiler | 1 |
| 7ade6912-d3c9-4d41-8292-ef315e673c9d | New gammaroid family, genera and species from subterranean waters of Japan, and their phylogenetic relationships (Crustacea: Amphipoda) | 4 |
| 5ff379c6-80b3-4c10-a154-b0e7fbf35a2f | Canada_BasinMegabenthos2002 | 4 |
| 8e6636ad-0b6a-426a-9515-985288f558ba | On the composition of the benthic fauna of the western Fram Strait | 35 |
| dad9eb2e-7749-4c99-b6cd-2b161d6419b0 | Composition and distribution of the biomass of zooplankton in the central Arctic Basin 1975, 1976, 1977 | 57 |
| 7137f448-f762-11e1-a439-00145eb45e9a | Biosphärenpark Wienerwald - Pfaffstätten | 2 |
| ed69f2e5-90d8-41ae-a653-b2cfb215f04d | données sur les ENS - données opportunistes sur les ENS 17 par l'association Environat | 1 |
| 59b549c0-7da9-4095-98c1-56da90837723 | DEMNA-DNE : Occurrences of benthic macroinvertebrates in running waters of Wallonia, Belgium | 6991 |
| bafa5083-8081-42f8-9201-04c511f45098 | Collections of Bioclass, school #179, Moscow | 84 |
| a414b648-a007-4ade-b993-b575013c5c70 | Macroinvertebrados asociados a diferentes sustratos presentes en el Golfo de Urabá 2018 | 33 |
| 6fddae5d-1ecf-4818-8a80-c56cf2494b4f | ZINRAS_Arctic_Benthos | 4 |
| 45af7bce-7df9-446c-8ccc-0ae1c750a335 | The Sea Ice Fauna of Frobisher Bay, Arctic Canada 1981 and 1982 | 8 |
| 0491f582-2bb3-4787-9324-05f1a77f20b7 | Abundance and diversity of the Amphipoda (Crustacea) from the Greenlandic shelf | 4452 |
| 451eb991-c1f4-479f-b1f8-7c1b4e8f9114 | Archives of the Arctic Seas Zooplankton | 681 |
| 4e78f4b4-4844-4e7c-b010-8dfa81d8563c | Ice Amphipods Canada Basin | 3 |
| a6ed9633-4efd-45c6-8bc7-41b16cf0b6ea | MBON POLE TO POLE: SANDY BEACH BIODIVERSITY OF BARRA DEL CHUY, URUGUAY | 15 |
| 53d3d9b9-8486-4cc6-8d97-dca8976fcf6e | Redescription of the rare, deep-sea hyperiidean amphipod Megalanceoloides remipes (K. H. Barnard, 1932) (Crustacea: Amphipoda: Hyperiidea), including the first description of males | 4 |
| 80576d53-992a-41f3-a3e3-dfd0fa0242ee | Ireland's BioBlitz | 57 |
| d48e028f-1b68-41ca-b206-57a747b8a894 | New genera and species of Urothoidae (Amphipoda) from the Brazilian deep sea, with the re-assignment of Pseudurothoe and Urothopsis to Phoxocephalopsidae | 11 |
| bc778fb5-3634-4da8-9297-0d6c2cdde0eb | The Ericthonius group, a new perpective on an old problem (Crustacea: Amphipoda: Corophioidea) | 7 |
| 6ba8e2df-519e-4fb2-84c5-d964a746f7e1 | ZUEC-CRU - Coleção de Crustacea do Museu de Zoologia da UNICAMP | 814 |
| bffa8458-36d3-4580-b446-7ac281a4785f | Macrobenthos Chukchi Sea, 1986 | 304 |
| 3b63eb62-1080-49f1-bf60-8beb6fa16994 | National River Water Quality Network Database (Macro-invertebrates) 1990-2008 | 248 |
| d7fd91b2-25fe-4541-8b66-b37fe38941b2 | ATBI Parc national du Mercantour / Parco naturale Alpi Marittime - EXPLOR'NATURE 2018, inventaire biologique de la commune de Sospel | 2 |
| fcf464de-3ef3-477d-90a2-6002f3c78143 | National River Water Quality Network Database (Macro-invertebrates) 2009-2018 | 144 |
| 25e8acc1-f6e9-4737-a2e0-996da57fc73d | Review of amphipods of the family Pleustidae Buchholz, 1874 (Amphipoda) from the coastal waters of Sakhalin Island (Far East of Russia). II. Subfamily Eosymtinae Bousfield & Hendrycks, 1994 | 3 |
| 28b321a4-8ca4-4535-97e3-3f96562af979 | SIO_FAMIZ | 7 |
| 0e1d85a0-b480-40c6-a4a1-65193806b424 | WhiteSeaPlankton | 475 |
| 9cc67633-537a-4acc-8d37-92feeaddb2cd | Monitoreo Ambiental del Proyecto La Colosa | 799 |
| 40a08be7-0642-4129-b12a-51d547dc2a74 | CASES: The pre-winter assemblages of southeastern Beaufort Sea | 50 |
| 2a096847-3148-4a09-a47c-ee42f573ae75 | IPOE_Benthos_Steffens | 86 |
| 868b5ca1-bb27-4d6f-b243-37ad77486a68 | The Ecology of the Inshore Marine Zooplankton of the Chukchi Sea near Point Barrow, Alaska | 36 |
| db758f3c-d74c-4e42-b66a-3f66107e1dc0 | RNF \| Habitats benthiques intertidaux, volet Taxons, des espaces protégés de la LPO France et de l'OFB - RNF Benthos - Relevés taxonomiques \| RNN Belle-Henriette | 90 |
| 95be1cf2-f762-11e1-a439-00145eb45e9a | Lustadter Wald . | 1 |
| 2ecc7f2a-f797-4131-84e5-0420fe9858de | Ferme pilote d'éoliennes flottantes de Groix & Belle-Ile et son raccordement au Réseau Public de Transport d'Electricité - Campagne d’investigations benthos – Ferme pilote de Groix & Belle-Ile | 45 |
| 350e81e8-ff86-41bb-8c71-5a6875fe9144 | Schellenberg A. 1936. The fishery grounds near Alexandria. Amphipoda Benthonica. Notes and memoirs No 18. | 133 |
| 456619c2-c56e-4d4b-86df-ddc80750bda8 | IMOS - Zooplankton Abundance and Biomass Index (CPR) | 72 |
| 04209a70-813b-421a-a250-e893b8836cdc | Data on biodiversity of a boreal mire and its hydrographic network (Shichengskoe mire, North-Western Russia) | 1 |
| a267b6a7-91f9-457c-889a-481e7aa920b6 | SIO Benthic Invertebrate Collection | 279 |
| 6230a436-f762-11e1-a439-00145eb45e9a | Dalbekschlucht | 2 |
| 4e3e00d6-6a60-401d-af5a-1a4816755f72 | SILENE-FAUNE-PACA - Parc_National_du_Mercantour_2017_12_18 | 1 |
| 6fa47516-f762-11e1-a439-00145eb45e9a | Distribution of meiobenthos in sediment core M19_202-2 of the northeastern Atlantic (Table 1) | 4 |
| 1d743188-1e65-4d99-a814-fa3fd51f1490 | Biological Reference Collections ICM-CSIC | 230 |
| 190ebf60-604c-48c3-a905-c26956572429 | Literature review of the Scheldt estuary with emphasis on the biotic components | 3 |
| 421890f7-e909-40ed-a573-b1c9529a55fa | Catch data from New Zealand research trawls since 2008 | 5 |
| 24c56165-469b-426a-98bb-2cbdef596680 | NW Pacific Deep-sea Benthos Biodiversity (Beneficial Project) | 1 |
| 1a6d5bcd-cb62-468f-967f-fd25e18c3b8c | River Biologists' Database (EPA) | 285 |
| 78dd6a48-f762-11e1-a439-00145eb45e9a | Benthic macrofauna abundance of sediment core 12930-059 | 14 |
| 78d6f5b4-f762-11e1-a439-00145eb45e9a | Benthic macrofauna abundance of sediment core 12930-039 | 14 |
| 78e76c46-f762-11e1-a439-00145eb45e9a | Benthic macrofauna abundance of sediment core 12930-093 | 14 |
| f6b72f89-09c9-4805-be09-96198ab19050 | Réserves Naturelles de France (RNF) - RNF - Données de l'association Vivarmor Nature | 462 |
| 69217c7b-5773-4015-a802-6af216b24c97 | DFO Quebec Region Biodiversity of the Planning for Integrated Environmental Response Coastal Survey in the St. Lawrence Estuary and Gulf (2017-2021) | 424 |
| bc2c3c27-97b6-473e-abe0-6f8478b3e2f8 | Colección de Invertebrados del Instituto de Investigación de Recursos Biológicos Alexander von Humboldt (IAvH-I) | 7 |
| daae5a66-7bb4-4431-ac3c-a1bf70ca3407 | Stackpole National Nature Reserve Species Inventory and Ad-hoc Sightings from Across Pembrokeshire | 17 |
| 05b22baa-5b85-4212-a93a-2fe81f5254dd | Base BOMBINA du Parc Naturel régional Lorraine - Modernisation des ZNIEFF du PnrL | 17 |
| 98af357b-541e-4f16-b35c-8ca68767be99 | River macroinvertebrate data for 2005 and 2006 | 271 |
| 2fd18368-47e7-49e8-9912-014093c433b3 | Coleção Zoológica da Universidade Federal de Mato Grosso do Sul - Crustacea (ZUFMS-CRU) | 1 |
| 9e2f8841-097e-4eba-b015-dddaf1708c7e | CardObs : Observations naturalistes issues de l'outil de saisie et gestion CardObs mis en place par le Service du Patrimoine Naturel (MNHN) / UMS PatriNat (OFB - CNRS - MNHN) - Données naturalistes de Pierre NOEL (M2MNHN) | 2 |
| d0e133c0-6c8a-11de-8226-b8a03c50a862 | Queen Victoria Museum Art Gallery provider for OZCAM | 603 |
| dce8feb0-6c89-11de-8225-b8a03c50a862 | Australian Museum provider for OZCAM | 31010 |
| 0102d2af-7f21-4238-b1fa-cbbeeee60423 | Naturalis Biodiversity Center (NL) - Crustacea | 20 |
| 4f28afd9-f50e-42aa-b49f-60d9f637ab2c | Colección Limnológica Universidad de Antioquia | 8 |
| 0b02615b-9e28-4425-b29f-1209f9a0a896 | Biological survey of the intertidal chalk reefs between Folkestone Warren and Kingsdown, Kent 2009-2011 | 38 |
| 8507dbf0-f762-11e1-a439-00145eb45e9a | CNCR/Colección Nacional de Crustaceos | 968 |
| f946666e-67dc-4848-9fa8-2162f3559e33 | Données d'occurrences Espèces issues de l'inventaire des ZNIEFF | 678 |
| f7fd7255-5be6-48cf-b76a-821cb84aa93f | BRERC Notable Species records within the last 10 years | 145 |
| 72d25812-af76-4ef0-9206-0f3c25d1aada | CardObs : Observations naturalistes issues de l'outil de saisie et gestion CardObs mis en place par le Service du Patrimoine Naturel (MNHN) / UMS PatriNat (OFB - CNRS - MNHN) - Données naturalistes de Julien BIRARD [Inventaire Eclair 18 juin] | 2 |
| db1abc39-7b60-4103-815d-820fa37cb62b | Protected and Invasive Species Records Collected Through Environment Agency Survey 1995 - 2021 | 22368 |
| 6bf0f3b3-053d-435d-ac96-1074625b937c | The First Comprehensive Description of the Biodiversity and Biogeography of Antarctic and Sub-Antarctic Intertidal Communities | 258 |
| 4fd22274-dd46-49bd-b592-47f2a867e8b1 | A revision of the bathyal and abyssal necrophage genus Cyclocaris Stebbing, 1888 (Crustacea: Amphipoda: Cyclocaridae) with the addition of two new species from the Atlantic Ocean | 8 |
| 6bcfbc19-4a2a-496f-ae62-5dcb36e335fb | CardObs : Observations naturalistes issues de l'outil de saisie et gestion CardObs mis en place par le Service du Patrimoine Naturel (MNHN) / UMS PatriNat (OFB - CNRS - MNHN) - Données naturalistes de Pierre NOEL (pnoel) | 2 |
| a3ed9fb7-68a3-4a4e-9d3b-8375bedd234f | New and additional records of hyperiidean amphipods of the infraorder Physosomata (Crustacea: Amphipoda: Hyperiidea) from the Antarctic Zone of the Southern Ocean | 11 |
| cefd283f-acf1-4da1-a46a-b5079925a6e8 | Zooplancton en Bahia Magdalena, Baja California Sur, Mexico | 25 |
| e3ce628e-9683-4af7-b7a9-47eef785d3bb | Earth Guardians Weekly Feed | 52 |
| 7ad079e7-f905-444f-a228-01746e3b7c6b | Visiolittoral : Base de données de la communauté de travail du Conservatoire du littoral - Visiolittoral : surveillance naturaliste des sites du Conservatoire du littoral | 18 |
| 7c93d290-6c8b-11de-8226-b8a03c50a862 | Western Australian Museum provider for OZCAM | 2467 |
| d3c28e6c-1192-4ef4-a38a-df790df16dc3 | Estonian Museum of Natural History Department of Zoology | 10 |
| e5bb465f-e713-45c1-8e0f-2c6125494b44 | A generic review of the lysianassoid family Uristidae and descriptions of new taxa from Australian waters (Crustacea, Amphipoda, Uristidae) | 229 |
| a32c9375-e432-467d-83fe-7be2408b6a48 | Benthic biota of CIMAR-Fiordos and Southern Ice Field Cruises | 162 |
| 33591b80-0e31-480c-82ce-2f57211b10e6 | Freshwater benthic invertebrates ecological collection NTNU University Museum | 1292 |
| 8f6c0996-30aa-42d3-9331-d2e319e01554 | Inventaires naturalistes du Service du Patrimoine naturel / UMS PatriNat - Inventaire de l'îlot du Lédénez Vraz | 4 |
| a031de99-c6cc-4826-b8aa-18ce01116184 | Base BOMBINA du Parc Naturel régional Lorraine - Inventaires "Mares" Parc naturel régional de Lorraine | 2 |
| ef1132ad-daaf-4dea-97eb-d5a6707208b8 | A review of the hyperiidean amphipod superfamily Vibilioidea Bowman and Gruner, 1973 (Crustacea: Amphipoda: Hyperiidea) | 12 |
| 116fd540-36a8-4c36-8274-67d90815fd5f | Comparative study of the organismic assemblages associated with the demosponge Sarcotragus foetidus Schmidt, 1862 in the coasts of Cyprus and Greece | 35 |
| 62e99340-528c-4baf-bbd7-bf0df2f186b9 | data.mnhn.lu observation data | 1 |
| 83ea4307-fd04-467c-af87-271a963841fc | Benthic communities and environmental parameters in Amvrakikos Wetlands: Mazoma, Tsopeli,Tsoukalio, Rodia and Logarou lagoons (September 2010 – July 2011) | 285 |
| fe4baa57-8f75-472b-982a-7711e3739873 | Réserves Naturelles de France (RNF) - RNF - Données de la Fédération des Réserves Catalanes | 5 |
| df662766-0716-4bea-bad0-158e985071c6 | Gulf of Gdansk | 28 |
| f7d6a733-fa5e-43ba-b06e-e70778953ead | Tasmanian Museum and Art Gallery provider for OZCAM-Arthropoda | 443 |
| bd1c0da1-f98e-43d7-b183-b8fa6e43a528 | Summary on benthic studies of the Southern Bight of the North Sea and its adjacent continental estuaries (Western Scheldt and Eems-Dollard) between 1976 and 1978 | 10 |
| 7ba26ff7-d8cb-4d95-912c-38cd8223c983 | BIOMAERL.Maerl Biodiversity.Functional Structure And Antropogenic Impacts (1996-1998). | 434 |
| 696df93e-6421-47e3-9fcc-c2a63fbae36f | Floresorchestia xueli, a new terrestrial crustacean (Amphipoda, Talitridae) from Yunnan, China | 3 |
| 3256a52e-1892-4425-b4dc-5ebb474692e8 | Macro- and megafauna from the North Aegean Sea from 1997-1998 | 517 |
| edfa3828-8b3b-45ff-836e-ff7a28442967 | Macro-and meiobenthos of an Avicennia marina mangrove in Gazi Bay in 1992 | 1 |
| 39905320-6c8a-11de-8226-b8a03c50a862 | Museums Victoria provider for OZCAM | 19946 |
| 9b11f305-fb7a-4a65-826e-7fb97af06e5f | Benthic communities and environmental parameters in three Mediterranean ports (Sardinia, Crete, Tunisia) | 278 |
| b7ca8e9d-5609-4356-b5c3-b2ca7071cf58 | Inventaire des Invertébrés marins de la Réserve Naturelle Nationale de Saint-Martin | 49 |
| 7ce13b6a-f762-11e1-a439-00145eb45e9a | (Table 2) Median abundances of macrobenthos in surface sediments | 180 |
| 7c425360-f762-11e1-a439-00145eb45e9a | (Table A-2.1 to A-2.7) Abundance of macrobenthos in surface sediments in the Arctic Ocean | 1422 |
| 00255299-4034-4f8d-86ed-805dacc0ec5d | Marine sites, habitats and species data collected during the BioMar survey of Ireland. | 642 |
| 790d1504-f762-11e1-a439-00145eb45e9a | Benthic macrofauna abundance of sediment core 13200-077 | 10 |
| b146a93c-657b-4768-aa51-9cabe3dac808 | AxIOM: Amphipod crustaceans from insular Posidonia oceanica seagrass meadows | 1775 |
| 2397e566-062d-4dd9-96cb-f65d9d5ad757 | Abundance of megabenthic species in trawl catches per station in addition to table 2 during POLARSTERN cruise ARK-VIII/2 (EPOS) | 576 |
| 7907f7d6-f762-11e1-a439-00145eb45e9a | Benthic macrofauna abundance of sediment core 13200-052 | 10 |
| 791b77a2-f762-11e1-a439-00145eb45e9a | Macrofauna abundance in surface sediment sampled during cruise DI222/2 | 5 |
| 791e2b78-f762-11e1-a439-00145eb45e9a | Macrofauna abundance in surface sediment sampled during cruise DI229 | 5 |
| 78fc80f4-f762-11e1-a439-00145eb45e9a | Benthic macrofauna abundance of sediment core 13200-004 | 10 |
| 79216572-f762-11e1-a439-00145eb45e9a | Macrofauna abundance in surface sediment sampled during cruise DI237 | 3 |
| 7916423c-f762-11e1-a439-00145eb45e9a | Benthic macrofauna abundance of subcores of sediment core 13078-015 | 51 |
| 79069c60-f762-11e1-a439-00145eb45e9a | Benthic macrofauna abundance of sediment core 13200-051 | 10 |
| 78f69752-f762-11e1-a439-00145eb45e9a | Benthic macrofauna abundance of sediment core 13077-098 | 14 |
| 79012be0-f762-11e1-a439-00145eb45e9a | Benthic macrofauna abundance of sediment core 13200-020 | 10 |
| 78ec0954-f762-11e1-a439-00145eb45e9a | Benthic macrofauna abundance of sediment core 13077-023 | 14 |
| 78f54442-f762-11e1-a439-00145eb45e9a | Benthic macrofauna abundance of sediment core 13077-093 | 13 |
| 78e92fcc-f762-11e1-a439-00145eb45e9a | Benthic macrofauna abundance of sediment core 12930-095 | 14 |
| 78eaa8f2-f762-11e1-a439-00145eb45e9a | Benthic macrofauna abundance of sediment core 13077-019 | 14 |
| 478e52db-0450-47f0-a763-ad3bcdaba6d9 | Non Fish Species Occurrence in ASEAN Region | 2 |
| a6086844-4a71-4915-aa58-790a12949e6d | Componentes biológicos estudiados en el área de influencia del tramo D, poliducto SUFAZ. La primera fase del Plan de Monitoreo y Seguimiento en el marco del proyecto SUFAZ | 2 |
| 84301d1c-15da-46ae-b8dd-6aeb47fa98e8 | Amaryllididae * | 1 |
| 8e434587-b145-47ef-9b35-fa3a130798a3 | Amphilochidae * | 4 |
| e6c97f6e-e952-11e2-961f-00145eb45e9a | Anymals+plants - Citizen Science Data | 4 |
| c2500323-6124-46e0-a008-06aedef14ad1 | Cyproideidae * | 6 |
| 25ab46cc-e4e0-4b43-8835-4078aefa83aa | Unciolidae * | 1 |
| dd03244d-a241-4d58-9ade-094fafb78310 | Liljeborgiidae * | 3 |
| 3bb32f83-5659-4a46-a4ef-5ead6bf9e689 | Megaluropidae * | 2 |
| a898db92-1916-47b9-9bee-5b7a1a0682a4 | Melphidippidae * | 1 |
| d15e654a-5d49-4939-9306-152f48e910a3 | Phliantidae * | 1 |
| aa4fb5eb-d529-46e5-a8ee-233d2c9ec349 | Synopiidae * | 4 |
| 3d55327c-e5d5-402b-aa7d-c537505e2876 | Urohaustoriidae * | 4 |
| a1574d82-a9e0-4b63-b026-b2475613765f | Wandinidae * | 1 |
| 977520a4-f762-11e1-a439-00145eb45e9a | Abundance of benthos infauna at station GIK23055-1 | 1 |
| 1af1afa5-bb7b-467c-8f5c-6fac7e4537d8 | Macrozoobenthos abundance of the intertidal zone of a sandy beach at the Island Algodoal-Maiandeua, Pará, Brazil | 72 |
| 14fefec5-7b5c-471e-bdee-71015622b05e | Hurleyella, a new genus of Nearctic Dolichopodidae (Diptera) | 3 |
| a786ae17-dcd8-4302-abb3-72d1a528b413 | (Table 2) Species density and composition of an inshore and offshore station in Kongsfjord, Svalbard | 18 |
| 895ee173-1561-4d52-9418-d721748a0942 | (Table 2c) Percentages of energy flow of macrobenthos species in samples from Cruise AMK54 stations in the Novaya Zemlya Trough | 24 |
| 327b65d7-c3d6-45f9-8bc9-5ed6e6a2717a | (Table A) Fauna associated with Bathymodiolus azoricus mussel beds within the Menez Gwen hydrothermal field, Mid-Atlantic Ridge | 16 |
| 60eb6b5a-dfc5-4edc-8750-a534e8809d45 | Macroinvertebrados bentónicos del muro de San Carlos- Zulia- Venezuela | 2 |
| c106367a-759e-40bc-9d23-c522c59dbdbe | Macrofauna abundances from mesocosm experiments at eastern and western branches of the Whittard canyon | 34 |
| 78ed6b3c-f762-11e1-a439-00145eb45e9a | Benthic macrofauna abundance of sediment core 13077-059 | 14 |
| 78c24b28-f762-11e1-a439-00145eb45e9a | Matter enrichment of the macrofauna community of MAC station 12930-005 | 16 |
| 78eed760-f762-11e1-a439-00145eb45e9a | Benthic macrofauna abundance of sediment core 13077-063 | 14 |
| 66551429-5e35-4848-94f5-b13bcad669a5 | SILENE-FAUNE-PACA - CEN_PACA_2017_12_18 | 3 |
| 70057258-f762-11e1-a439-00145eb45e9a | Meiofauna abundances at the Arctic Håkon Mosby Mud Volcano (HMMV) of sediment core PS64/362-1 | 5 |
| 7002f028-f762-11e1-a439-00145eb45e9a | Meiofauna abundances at the Arctic Håkon Mosby Mud Volcano (HMMV) of sediment core PS64/356-1 | 5 |
| 7120fc8e-f762-11e1-a439-00145eb45e9a | Benthos counted on dredged samples during Valdivia cruise VA44 | 92 |
| 632c969c-f762-11e1-a439-00145eb45e9a | Tauchen und Meer | 1 |
| f2ec825d-145a-42fd-9bfe-a411a557bc47 | Invertebrates (Type Specimens) of the Swedish Museum of Natural History | 209 |
| c47c303d-9ef5-4b02-ba57-cea6e20c580f | Study on plankton at the port of Ostend in 1965 | 9 |
| 84d4a910-f762-11e1-a439-00145eb45e9a | Priest Pot species list, Cumbria, Britain | 2 |
| 86b7940e-f762-11e1-a439-00145eb45e9a | AAD Benthic Sampling Database | 377 |
| 14f3151a-e95d-493c-a40d-d9938ef62954 | CAS Entomology (ENT) | 3 |
| c9d089f6-7cf5-4204-9952-c8ac79da38de | Water Framework Directive AGE, Recorder-Lux database | 672 |
| 3ca514bf-717b-40cb-a576-7af2635f7fe8 | Seasonal fluctuation of the meiobenthos community in Avicennia marina mangrove sediments at Gazi Bay (Kenya) between 1992 and 1993 | 13 |
| 0645ccdb-e001-4ab0-9729-51f1755e007e | NSW BioNet Atlas | 14 |
| 09c38deb-8674-446e-8be8-3347f6c094ef | Jordal | 1 |
| 68ce269d-4a2d-4a49-9555-36f01aa66bf9 | Macrobenthos monitoring in function of the Water Framework Directive in the period 2007-2009 | 391 |
| 06a94895-8b0e-4021-9cdf-3e9476b2bf2f | Système d'Information sur la Nature et les Paysages d'Ile de France - Données de la structure ONEMA/AFB provenant de la base de donnée du SINP Île-de-France CETTIA | 1 |
| 5a7eac3e-a0be-42bb-81eb-adcd2eb31806 | DRAGAGE DE LA BAIE DE TXINGUDI - Inventaire macrofaune benthique | 38 |
| 0e988b71-2cc1-4cd3-a026-ef14c056b05c | Coastal and Marine Species Database | 8 |
| 322f41ce-1e12-494a-98ea-af94241b09ed | Kongsfjorden/Spitsbergen - soft bottom fauna | 13 |
| c6bb9397-f67f-4ecd-a85a-a27debe6ce3c | Macrobenthos monitoring in function of aggregate extraction activities in the Belgian part of the North Sea | 2994 |
| aeab6ee6-c27f-4f1c-8c20-418decd54684 | REBENT - Réseau national de surveillance des biocénoses benthiques côtières | 20321 |
| 979bd8fc-f762-11e1-a439-00145eb45e9a | Benthic macrofauna abundance and biomass in surface sediment during cruise PLG95A | 14 |
| 978e88c8-f762-11e1-a439-00145eb45e9a | Benthic meiofauna abundance in surface sediment of core PLG93_BCORE1 | 8 |
| 32de56e2-db99-4f9a-bc23-54d629013809 | Ogasawara DNA barcode database | 8 |
| 50c9509d-22c7-4a22-a47d-8c48425ef4a7 | iNaturalist Research-grade Observations | 2449 |
| 547f2b71-ba25-4254-aaf1-4823600e7784 | Merseyside BioBank Active Naturalists (unverified) | 31 |
| 9799170c-f762-11e1-a439-00145eb45e9a | Benthic macrofauna abundance and biomass in surface sediment during cruise CD86 | 25 |
| 3151231f-0ad5-480d-8529-456dd4a74e55 | Manomet Intertidal Green Crab Project - Powered by Anecdata.org | 1 |
| 70f49e37-aea1-4ede-a21a-03057bc0a984 | Données Faune Base SIRFF - FNE Centre-Val de Loire - Système d’Information Régional sur la Faune et la Flore - FNE Centre-Val de Loire - Données 2016 | 3228 |
| d423429a-696f-4bce-b65e-7cbbdf9e87ab | The Caucasian relicts: a new species of the genus Niphargus (Crustacea: Amphipoda: Niphargidae) from the Gelendzhik-Tuapse area of the Russian southwestern Caucasus | 8 |
| 4e61cc4a-6bfd-4816-b2b8-b066e21b6235 | Ohio Wesleyan University Paleontology Specimens (Arctos) | 1 |
| 4f5c4cb2-271c-47b4-8d95-7cc9d5bee766 | Analysis of the genus Sunamphitoe Spence Bate, 1857 (Amphipoda: Ampithoidae) with descriptions of eight new species | 24 |
| 784367b4-ff7a-49bf-af68-041daaa19ba2 | Nansen Legacy JC1 mesozooplankton biodiversity | 68 |
| e2b9a599-b5b4-4196-8c2a-d57f678208be | Données naturalistes CEN Centre-Val de Loire - Conservatoire d'espaces naturels Centre-Val de Loire - Données Faune au 1/11/2020 | 31 |
| 85b1cfb6-f762-11e1-a439-00145eb45e9a | UF Invertebrate Zoology | 2827 |
| 1aed0317-9b87-477a-9b2f-6db0381ce85c | A new species of the genus Rhinoecetes Just, 1983 (Crustacea: Amphipoda: Ischyroceridae) from Japan | 1 |
| 6d56415d-b007-4273-9c74-bcd6b2467434 | The CPR Survey | 124 |
| fc4e2dc0-3d87-4881-b59f-192c0afde08f | Macrobenthos: temporal patterns for stations 115b and 330 in the Belgian Part of the North Sea | 10 |
| d45b8e8a-1f32-40b0-a132-bd690bd90b30 | ZFMK Hymenoptera collection | 2 |
| 713e3cc9-4ae8-452f-91b2-081d9a0bd630 | Study of epibenthos and demersal fish in and around the dredging areas of the Belgian Continental Shelf (1977-1981) | 1 |
| f6c4b312-6a86-4fb1-a210-b861f8d1c899 | Colección Zoológica de la Universidad del Tolima (CZUT) - Macroinvertebrados | 56 |
| d185ab72-3518-4cb8-b9ad-79010d6eef70 | Invertebrata varia (Luomus) | 2266 |
| 48060741-47d3-4093-86e6-2ec484323cc5 | DFO Pacific Groundfish Synoptic Trawl Surveys - Hecate Strait | 1 |
| 3d4cac0a-7441-4099-8eed-8a270d522f8f | DFO Pacific Groundfish Synoptic Trawl Surveys - Queen Charlotte Sound | 5 |
| c6ca96bc-7cf5-4fe7-ac44-d01e06394ef3 | Lizard Island Research Station | 16 |
| 3b91f3e4-17da-4965-a8d5-4e8c9296477b | SeaWatch-B: citizens monitoring the Belgian North Sea from the beach (2014-2018) | 2 |
| e3004062-f320-4d91-8192-3ec889e468dc | Kent Wildlife Trust Shoresearch Intertidal Survey 2004 onwards | 182 |
| 84a47405-034b-4ab3-bb49-b2ab36a21a88 | ForestCheck Invertebrates | 134 |
| b6a37bbd-95d2-4c5e-8b9e-3fbefd8bd8cd | Woodmeadow Invertebrate Survey 2016 | 1 |
| bb281016-1373-441e-bc6d-db8fcb6bc890 | Amphipoda (Crustacea) from Palau, Micronesia: Families Maeridae and Melitidae | 3 |
| c44ce8a6-ecd3-4516-b741-0268a35fd117 | SHARK - National marine environmental monitoring of Zoobenthos in Sweden since 1971 | 14662 |
| 3b88fd26-936c-4fe7-bbb7-684ba6264cf1 | PIKE – Distribution, extinctions and introductions of freshwater fish in Sweden | 2 |
| a46ef430-15e2-11dd-b4e8-b8a03c50a862 | Limnodata | 1132 |
| 9fcd8de5-a2b6-4c72-b863-debe63ee3a72 | DFO Pacific Groundfish Synoptic Trawl Surveys - West Coast Haida Gwaii | 20 |
| eca0f10b-998a-4450-85c0-d2fae635000a | SHARK - National marine environmental monitoring of zooplankton in Sweden since 1979 | 34 |
| 813d800b-7d9f-4550-9e04-1e13bb114e7e | The Rock Pool Project database - intertidal species records from rocky shore habitats - from February 2019 | 149 |
| 8646a0c8-f762-11e1-a439-00145eb45e9a | RMT Trawl catch from the 1995/96 V4 BROKE voyage | 788 |
| 02a4348e-69b2-4b8c-a474-79361ef37e3c | SHARK - Regional marine environmental monitoring, recipient control and monitoring projects of Zoobenthos in Sweden since 1972 | 26427 |
| 233ba471-91a2-47a4-9946-b7e844f2124c | Montgomeryshire Wildlife Trust records held by BIS | 2 |
| 7118a4ac-2cb2-4c10-b283-c8d2b7c7d5d5 | Riverfly Census - Aquatic invertebrate species occurrence, for the calculation of pressure biometric scores in English and Welsh rivers covering the period 2015 - 2018 | 586 |
| 2c7c72b2-ac29-4be3-a95f-6b09e8f4b932 | Isle of Man historical wildlife records 1995 to 1999 | 17 |
| 56aa0680-0c60-11dd-84cd-b8a03c50a862 | Invertebrates Collection of the Swedish Museum of Natural History | 716 |
| f3823f80-cd2d-4bbb-b9de-b27ac275ea7e | Macrobenthos and Phytoplankton monitoring in the Belgian coastal zone in the context of the EU Water Framework Directive (WFD) | 510 |
| b6528a71-4b81-4919-81d0-6f290763bcdd | Description of a new species of Liropus (Crustacea, Amphipoda) from Puerto Vallarta, Pacific coast of Mexico | 2 |
| 8a863029-f435-446a-821e-275f4f641165 | Observation.org, Nature data from around the World | 1401 |
| 63651725-99d5-4e9f-b78d-cb9dc13df22e | Zooplankton and phytoplankton densities in the oyster farms of Gazi Creek (Kenya) sampled in October 1992 | 6 |
| 2c28a663-db16-48d3-9cd0-3c7ec8d8d873 | NINA Vanndata øvrige arter | 1498 |
| c74e5e22-fe3b-40ca-bb70-558f5fb0b0f0 | Gestion de sites - Données invertébrés sites CEN-LR | 1 |
| 2150bbfa-51d0-4557-ab15-fb9459881eb7 | INNS Data: All Taxa (South East Wales) | 3 |
| aba10cb3-e79b-45f8-91a7-ef79d2133af9 | City of Edinburgh Natural Heritage Service - Ranger Ad-hoc records and sightings | 2 |
| 83e62a56-2140-439c-88fc-5573f28a0519 | Description of a widely distributed but overlooked amphipod species in the European Alps | 1 |
| 8ab25605-ec09-4d57-9dbd-7da2b032e5e8 | Tirons of the world: a review of ‘ tironid’ amphipods, description of new genera and species, and establishment of a new subfamily Tironinae Stebbing, 1906 stat. nov. (Crustacea, Synopiidae) | 19 |
| 0d9accc4-309d-44ad-901e-f221e1d0ec80 | Isle of Man wildlife records from 01/01/2000 to 13/02/2017 | 79 |
| 653ed711-e58d-465f-95f7-3d9f60442297 | Marine benthic dataset (version 1) commissioned by UKOOA | 15419 |
| d03f444a-7192-4fa0-bf7a-63714585c611 | England Non Native Species records 1965 to 2017 | 32601 |
| 139a966c-22d5-486b-bff2-cfcbccd6fdfc | Marine Nature Conservation Review (MNCR) and associated benthic marine data held and managed by JNCC | 20109 |
| 67891a7d-e2d4-4a82-969b-42116915d167 | Subtidal macrobenthos monitoring in function of a foreshore suppletion at the Belgian coast, period 2013-2016 | 295 |
| bf6ec1a0-c7dd-456c-bbd9-465e626a3267 | Macrobenthos monitoring at long-term monitoring stations in the Belgian part of the North Sea between 1979 and 1999 | 479 |
| c3c56ab1-706c-4e7f-85f8-f6f860e203f2 | Merseyside BioBank (verified) | 7 |
| 83a517aa-f762-11e1-a439-00145eb45e9a | Atlantic Reference Centre Museum of Canadian Atlantic Organisms - Invertebrates and Fishes Data | 3773 |
| f88d3365-634e-411b-9901-e5ca069de5ac | Feeding rhythms of the common goby Pomatoschistus microps at the brackish tidal marsh 'Het verdronken land van Saeftinge' in 1994 | 35 |
| be19d04a-044f-4def-a298-186a3616be8f | Macrobenthos of the Voordelta (Grevelingen, Oosterschelde) in 1988 and 1989 | 47 |
| 27448b7d-f4c5-4ccf-9ffc-31c91bf42cec | Scavenging Amphipods, Porcupine Abyssal Plain Sustained Observatory, North Atlantic, 1985-2016 | 167 |
| 158cea08-e128-447b-8154-30d3f0bfe9ce | Evaluación de la efectividad de las métricas derivadas de los invertebrados acuáticos para establecer la integridad ecológica de ríos de páramo: caso de estudio del Parque Chingaza | 5 |
| 83b0cc08-f762-11e1-a439-00145eb45e9a | Grand Manan Basin Benthos | 21 |
| 37beb6ea-e591-41e3-b781-e384250dc42c | Victorian Biodiversity Atlas | 1784 |
| f1c4df18-12d6-40cb-ab51-5bb0d7f08d6e | Estonian Naturalists’ Society | 13520 |
| 4ffe66a0-6c8b-11de-8226-b8a03c50a862 | Tasmanian Museum and Art Gallery provider for OZCAM | 599 |
| f38cc07c-49a8-4ca4-8882-5cdde1733c12 | Sustainable Rivers Audit | 1411 |
| 0e5c0373-f957-4ce3-b8d9-a9054df51fc5 | MDFRC macroinvertebrate survey | 697 |
| 2368ec8a-ba39-4a8f-bc34-69b5857ba965 | Eurythenes atacamensis sp. nov. (Crustacea: Amphipoda) exhibits ontogenetic vertical stratification across abyssal and hadal depths in the Atacama Trench, eastern South Pacific Ocean | 1 |
| 4bce5a53-c1d9-4e06-a773-cfd18ccd198f | BioChem: Zooplankton of St. Margarets Bay 1968 to 1971. | 10 |
| 8a173e64-7f56-4310-9046-e48062533e20 | A new species and new record of the genus Sinocorophium (Crustacea, Amphipoda, Corophiidae) from Korean Waters | 2 |
| 82d2673d-b093-4f6c-8e0d-75be79174bf0 | Projet de confortement de l'ouvrage de protection face à l'érosion et rechargements en sable associé - Macrofaune benthique | 2 |
| 83ac301c-f762-11e1-a439-00145eb45e9a | Bay of Fundy Species List | 147 |
| 477b3b5b-a24f-4fee-8c8d-0fe2d2ef1791 | Zooplankton community and pelagic copepods in Tudor creek (Mombasa, Kenya) between 1984 and 1987 | 10 |
| c7919f38-10fd-4806-81e2-4e81135b6af0 | HBRG Other Invertebrates Dataset | 25 |
| 82544e93-afc6-4126-a36f-9abd5bf7e162 | Abundance of mesozooplankton in the Mediterranean Sea during PELMED 1995 and PELMED 1996 | 49 |
| af0e9077-cfce-4559-9fca-70ad730eb57f | Abundance of mesozooplankton from nine cruises in Northern Adriatic Sea during NA64 | 18 |
| 0e0efc07-474c-402e-92af-feaf3445e4f7 | An inquiline deep-water bryozoan / amphipod association from New Zealand, including the description of a new genus and species of Chevaliidae | 1 |
| 33b837c9-dd05-48e3-b4ad-3273eb292125 | Ecological study of the plankton in the port of Ostend in 1965 | 3 |
| 8b8657af-5ba4-49bf-b665-e58aa5158409 | Two new species of the genus Victoriopisa Karaman & Barnard, 1979 (Crustacea: Amphipoda: Eriopisidae) from mangrove communities of Vietnam with a review of previous records | 2 |
| 202af80e-3bd1-4856-9f67-833588872230 | Local BioBlitz Challenge 2013 | 2 |
| d48850ad-3f90-434f-b105-09584cd147db | Macrozoobenthos, Joint Open Sea Surveys August 2017, EMBLAS-II | 3 |
| d9f7b2f6-03b3-4cd3-817b-c61949d79b1d | Système d’évaluation de l’état des Eaux (SEEE) - Données hydrobiologiques sur l'état des eaux de surface - Système d’évaluation de l’état des Eaux (SEEE) - Invertébrés | 24965 |
| 951a8449-9d4e-44f6-8e29-cc9ecbabf0ed | Amphipoda of Desna river basin | 50 |
| 6758c39f-119f-401b-bf6e-c458ea4f4dca | Revision of Leucothoe (Amphipoda, Crustacea) from the Southern Ocean: a cosmopolitanism concept is vanishing | 6 |
| 6f19bb5e-ef43-492e-af23-608f2d84e064 | DNA metabarcoding assays reveal a diverse prey assemblage for Mobula rays in the Bohol Sea, Philippines | 1 |
| 3b2b90d2-fd5a-493b-9221-412a591cb12a | CardObs : Observations naturalistes issues de l'outil de saisie et gestion CardObs mis en place par le Service du Patrimoine Naturel (MNHN) / UMS PatriNat (OFB - CNRS - MNHN) - Données naturalistes de CHAUVIN Jacques | 2 |
| b9f09593-6aa2-4481-8e98-51b5626af5ac | Hermesorchestia alastairi gen. et sp. nov. from Australia (Talitridae: Senticaudata: Amphipoda: Crustacea) | 16 |
| c19a2731-65c5-4f62-87d0-afbf49d1c7ce | Tidal and diurnal rhythms of the hyperbenthos at the 'Vlakte van de Raan' on 14 October 1996 | 243 |
| d3290fe3-c924-4708-87d5-1955db1f9077 | Spatial distribution of meiobenthos in the Voordelta (October 1988) | 13 |
| 5f04cc93-43ce-4ee2-8fe7-93a0bf5b8702 | kerkyra | 101 |
| 51de9c85-0337-4c68-9783-f13bc0aac15f | Commissioned surveys and staff surveys and reports for Scottish Wildlife Trust reserves - Unassessed data | 22 |
| 821765c9-59dc-4310-9970-d82c2eb9eff7 | Survey and monitoring records for Scottish Wildlife Trust reserves from reserve convenors and Trust volunteers - Unassessed data | 11 |
| 1bfb9389-ed6e-4415-81fe-c235e56fc70d | Study of epifauna and meiobenthos using field exclusion experiments in a Ceriops tagal and Avicennia marina mangrove at Gazi Bay (Kenya) in August and September 1992 | 18 |
| cabdbbb1-1226-4c2e-b90b-fae0742b5091 | Platorchestiinae subfam. nov. (Amphipoda, Senticaudata, Talitridae) with the description of three new genera and four new species | 7 |
| 4b84979a-e3de-4956-b9c7-91e178dd46b1 | The talitrid amphipod genus Talorchestia from the South China Sea to the Indonesian Archipelago (Crustacea, Senticaudata) | 22 |
| e87404ca-8d9b-4e13-9cf6-8fb0408bb3cf | CardObs : Observations naturalistes issues de l'outil de saisie et gestion CardObs mis en place par le Service du Patrimoine Naturel (MNHN) / UMS PatriNat (OFB - CNRS - MNHN) - Données naturalistes de BLOND Cyrille | 2 |
| 5ecc61f9-5ea0-4200-9ca7-377a452a0323 | Análisis taxonómicos de macrofauna bentónica para el Plan de Vigilancia Ambiental de las instalaciones de prueba de la sección 3 de Navantia Cartagena | 6 |
| f900725d-db2f-4771-9330-7960ce68d4b5 | Invertebrates compiled by W.Block | 6 |
| bfd91dba-b0f0-4724-9c55-8b5ecd35ee67 | Four new species of Cymadusa Savigny, 1816 (Amphipoda: Ampithoidae) and new records of C. filosa Savigny, 1816 from Brazilian coast | 7 |
| 353cfdc2-dda2-46fc-b613-18d519536ec0 | A new Hyalella species (Crustacea: Amphipoda: Hyalellidae) from South American Highlands (Argentina) with comments on its cuticular ultrastructure | 3 |
| d9ad8d5b-dd70-4efe-8a01-76ea5babc6b8 | Pelagic amphipods (Crustacea: Amphipoda: Hyperiidea) in western Mexico. 7 Superfamily Platysceloidea. Family Oxycephalidae | 54 |
| e9fb53be-c235-4070-986a-33faea0919e5 | A new species of Microdeutopus, M. periergos sp. nov. (Crustacea, Amphipoda, Senticaudata, Aoridae) from Cyprus (East Mediterranean Sea) | 5 |
| 9668b676-f762-11e1-a439-00145eb45e9a | Collection Crustacea SMF | 990 |
| 7b7bb606-f762-11e1-a439-00145eb45e9a | Collection Crustacea - ZIM Hamburg | 248 |
| b8b1c620-3356-480c-80e9-7fb113ea80d6 | Type material housed in the Carcinological Collection of the Museo de La Plata, Argentina | 2 |
| e10e2266-a58f-4679-98b8-2152dbcaaa21 | First island species of Hyalella (Amphipoda, Hyalellidae) from Florianópolis, state of Santa Catarina, Southern Brazil | 1 |
| 46723a60-13b9-4495-8977-5fc1cf1bf31e | Macro-and meiobenthos of a sublittoral sandbank in the Southern Bight of the North Sea (5 September 1978) | 23 |
| 7247dc2e-b862-4555-b533-7701d183271b | Programme d'observation naturaliste participative avec l'application mobile INPN Espèces - Application INPN Espèces: Observations naturalistes, participatives et opportunistes, fondées sur des photographies | 27 |
| 26098c25-8f7f-4c71-97ac-1d3db181c65e | NMNH Material Samples (USNM) | 2342 |
| df73d0c5-ddbd-4e36-8dd0-b49a0b6e4ab3 | A revision of the genus Pontocrates Boeck, 1871 (Amphipoda, Oedicerotidae) with the description of P. moorei sp. nov. and the re-establishment of P. norvegicus (Boeck, 1860) | 41 |
| 7b61ebd6-f762-11e1-a439-00145eb45e9a | Antarctic Marine Species Sequence Data | 19 |
| 4900eaac-756a-4e05-85ac-4f568030d62f | Taxonomic study of the genus Gammarus (Amphipoda, Gammaridae) from Xinjiang, China, with description of a new species | 1 |
| 962f59bc-f762-11e1-a439-00145eb45e9a | Collections and observation data National Museum of Natural History Luxembourg | 90 |
| c0e410ad-0d01-49f3-a837-71982c0e0c32 | TWIC Biodiversity Field Trip Data (1995-present) | 9 |
| ec186b76-898c-435b-9aea-ebbd5785a25f | Maritimes Summer Research Vessel Surveys | 101 |
| 5a0e9dc9-d2b0-489e-91b6-c1e2aca0d62c | Arctic benthic invertebrate collection of the Zoological Institute of the Russian Academy of Science | 6005 |
| 30b16b55-3967-4ff1-a6db-2b828be1f75a | Macroinvertebrados bentónicos de playas de arena (Monte Hermoso-Pehuen Có, Buenos Aires, Argentina) | 5 |
| 6ee7cc49-13fc-4cc3-b6d1-ab3e50903ec4 | Zooplankton Eastern Arctic Ocean Polarstern, 1995-1998 | 158 |
| 5e1d5399-d797-47cc-9c19-a401b9e1c1c6 | SACOI3 - inventaire faune flore et habitats | 16 |
| 804741e6-f762-11e1-a439-00145eb45e9a | Diversidad bentónica del ambiente intermareal e infralitoral somero de Progreso, Yucatán | 7897 |
| 2206386c-93c8-49e0-a9b2-5efff0c20536 | Anfípodos_IMO | 496 |
| 79e21918-887b-4b40-9305-abc5f57494dd | Biodiversity of Luiz Saldanha Marine Park | 635 |
| acf2b7a6-7f3c-443b-a3fe-307fda431e51 | Siphonoecetinae (Crustacea: Amphipoda: Ischyroceridae) 7: Australian Concholestids, Ambicholestes n. gen., with a Description of Six New Species, and a New, Restricted Diagnosis for Caribboecetes Just, 1983 | 32 |
| 00ed18ea-4993-4809-a656-62363baa4808 | Canadian Museum of Nature Parasite Collection | 12 |
| 64a446eb-b7c8-4685-a80b-70830e4419a4 | Cold-water corals off Angola as refuge for a new Aeginella species (Crustacea: Amphipoda: Caprellidae) | 2 |
| 23bab45d-e5c8-43fd-8fc5-6a8668becd16 | Données issues des cartes validées dans le cadre de l'Atlas de la Biodiversité Départementale et des Secteurs Marins | 118 |
| afaa80a2-6167-47e1-baae-5450206d07dd | The Arthropoda Varia Collection at the Zoologische Staatssammlung München | 1 |
| 402c81b6-8d69-4650-8826-92f91d2728a7 | Epipelagic mesozooplankton distribution and abundance in Southern Ocean Atlantic sector and the North Atlantic and Arctic 1996-2013 | 205 |
| c665b271-454b-4844-a039-426dac634908 | Leicestershire and Rutland Environmental Records Centre records 2015-2019 | 9 |
| c13b687b-5750-456a-856f-4b64303f713f | Leicestershire and Rutland Environmental Records Centre records pre 2000 | 1246 |
| fd7cda6d-0bdb-44ed-9319-010c286d4633 | Leicestershire and Rutland Environmental Records Centre records 2010-2014 | 119 |
| 3965d122-ad7d-463e-90be-7eaa006bea18 | Leicestershire and Rutland Environmental Records Centre records 2000-2009 | 240 |
| 1948a896-92c8-42de-a01a-38176a407e5a | 1848-1982 Lundy Field Society The Marine Fauna of Lundy | 458 |
| 219285f8-b3c9-4c34-963e-bdd3c14692fd | Isle of Man Historical Wildlife Records pre-1990 | 8 |
| e64fcb2f-1600-4092-b432-68d351d465a8 | Stable isotope ratios of C and N in benthic macrofauna from Mediterranean seagrass litter accumulations from Calvi Bay in 2011-2012 | 261 |
| e6cfb0ad-4507-4aa9-8f8d-58b3b6e6cd4f | Zooplankton studies in the Southern Bight of the North Sea between 1971 and 1974 | 24 |
| fc5d68a6-b511-4ccf-bdeb-84c209a4c824 | Macroalgas, equinodermos y crustáceos de la Isla Cayo Serranilla durante la Expedición Seaflower 2017 - Proyecto Colombia BIO | 2 |
| 3e0e4ec9-1905-4cae-9691-c0fa79361ac3 | A dataset of marine macroinvertebrate diversity from Mozambique and São Tomé and Príncipe | 69 |
| 4b301041-dbb5-4ac8-87e0-6ad96526a364 | Oison : outil de saisie des observations naturalistes réalisées par les agents de l'Office français de la biodiversité (OFB) - Données d'observations "espèce" réalisées par des agents de l'OFB jusqu'au 31 décembre 2018. | 4 |
| 829598f1-ef80-4731-85b2-006b10bc0208 | Zooplankton biomass and species composition and abundance in the southeastern Caribbean Sea (Cariaco Basin, October 2001 – January 2017) collected by the CARIACO Ocean Time-Series Program | 249 |
| 5e43a220-a91d-485b-b608-58fd22693be7 | All taxa records for Leicestershire and Rutland | 39 |
| 8138eb72-f762-11e1-a439-00145eb45e9a | RBINS DaRWIN | 8180 |
| 90eae6b8-57d2-4d24-8eb3-1dbde00f1669 | NRW Regional Data: North Wales | 15 |
| f539f857-d9be-4342-a26d-fb6033fb9c1d | Biodiversidad de macroinvertebrados bénticos de la región marina Tijuana-Ensenada Baja California, México | 332 |
| 47b61433-ac6e-4a4f-b81d-bfa798717be5 | Invertebrate Collection of IBIOMAR (CCT CONICET-CENPAT) (CNP-INV) | 120 |
| 7b714568-f762-11e1-a439-00145eb45e9a | RMT Trawl catch from the 1984/85 V5 SIBEX2 voyage | 178 |
| c358edb6-9b46-45eb-a173-b854910500a9 | Fife Nature Records Centre combined dataset | 79 |
| 84cb653a-f762-11e1-a439-00145eb45e9a | Marine Benthic Fauna List, Island of Læsø, Denmark | 25 |
| 62e159de-f762-11e1-a439-00145eb45e9a | Gewässeruntersuchung (Prien) mit Kl. 4b | 1 |
| 8abe750e-f762-11e1-a439-00145eb45e9a | Unna-Mühlhausen, Wiesen | 1 |
| e55a4257-819d-48e2-a0af-83c3448bc0ee | Ecological study of intertidal clay-and peatbanks at Raversijde (Belgium) in 1970 | 18 |
| 80811fba-f762-11e1-a439-00145eb45e9a | Base de datos de fauna batial, abisopelágica y abisal del Golfo de México | 3 |
| 30f2c59d-5818-461b-92b3-9072d705e757 | Three new species of the family Aoridae collected from Sagami Bay, central Japan (Crustacea: Amphipoda) | 10 |
| ad73fa3b-cc93-4709-a41d-dbac4ac3d21d | Manx Biological Recording Partnership UNVERIFIED Isle of Man records between 14/02/2017 and 05/09/2019 | 85 |
| f3053f73-094c-4db7-9273-58978ecf5d77 | Colección de Insectos de la Universidad del Quindío | 2 |
| 8a1e4556-ca01-4c98-8b04-c522cce74f80 | Numérisation des données faune contenues dans les Bulletins de la Société Linnéenne de Bordeaux - Bulletin de la Société Linnéenne de Bordeaux, Tome 136 (N.S) n° 29 (2), 2001 - Données faune | 1 |
| 62ab4c96-a5a9-4090-b99f-c2308600edca | Invertebrados del Parque Nacional Arrecife Puerto Morelos | 176 |
| ceac4558-4a9a-4cb4-917a-f2c99a112f35 | Woodmeadow Invertebrate Survey 2019 | 1 |
| 2dfe7e9e-bc3a-49f6-b0ee-15a64b0388f8 | anfipodos_mopex | 26 |
| 9767ee34-f762-11e1-a439-00145eb45e9a | Abundance of benthos infauna at station GIK23013-2 | 4 |
| 977e9daa-f762-11e1-a439-00145eb45e9a | Abundance of benthos infauna at station POS128/2_267 | 1 |
| 97828cc6-f762-11e1-a439-00145eb45e9a | Abundance of benthos infauna at station POS128/2_284 | 1 |
| 9783d11c-f762-11e1-a439-00145eb45e9a | Abundance of benthos infauna at station PS1239-1 | 2 |
| 978670de-f762-11e1-a439-00145eb45e9a | Abundance of benthos infauna at station PS1241-1 | 1 |
| 7c3fa69c-f762-11e1-a439-00145eb45e9a | (Table 2a and b) Median abundances of macrobenthos in surface sediments | 204 |
| 78d19fa6-f762-11e1-a439-00145eb45e9a | Benthic macrofauna abundance of sediment core 12930-014 | 14 |
| 78d2d2f4-f762-11e1-a439-00145eb45e9a | Benthic macrofauna abundance of sediment core 12930-017 | 14 |
| 78dbb892-f762-11e1-a439-00145eb45e9a | Benthic macrofauna abundance of sediment core 12930-052 | 14 |
| 78e03624-f762-11e1-a439-00145eb45e9a | Benthic macrofauna abundance of sediment core 12930-065 | 14 |
| 081ca37d-8ff2-4168-9970-04d93af3c060 | Another new species of Glossocephalus (Crustacea: Amphipoda: Hyperiidea Oxycephalidae) from the Monterey Bay region, California, USA | 4 |
| 0265ff07-12b4-4926-b38a-0e66e7bc0147 | Réseau de suivi des macro-invertébrés aquatiques - Inventaire et suivi Macroinvertébrés | 574 |
| 78f02dea-f762-11e1-a439-00145eb45e9a | Benthic macrofauna abundance of sediment core 13077-070 | 14 |
| 78f1cf88-f762-11e1-a439-00145eb45e9a | Benthic macrofauna abundance of sediment core 13077-087 | 14 |
| 78f39fb6-f762-11e1-a439-00145eb45e9a | Benthic macrofauna abundance of sediment core 13077-089 | 14 |
| 6e4b215e-9019-4934-8433-65d80a35c230 | New Zealand Arthropod Collection (NZAC) | 278 |
| 07609307-26f9-490f-89ea-4e74497e3c0d | Brecknock Wildlife Trust (Now WTSWW Brecknockhire) records held by BIS | 12 |
| 5a1b62b2-4a1f-4404-b481-87836564d4a5 | Miscellaneous records held by BIS | 61 |
| 6e09f9dc-0bf2-4c20-8ff6-bd898bbc4831 | Radnorshire Wildlife Trust records held by BIS | 2 |
| ecca1a3e-3b7d-4229-8a7a-d106997e6d03 | UAM Insect Observations (Arctos) | 99 |
| 80ebe7f9-00ee-4fe3-80f6-303729cf5bac | UAM Invertebrate Collection (Arctos) | 824 |
| dda871e8-9212-484c-83be-c68d5536e571 | New deep-sea Atlantic and Antarctic species of Abyssorchomene De Broyer, 1984 (Amphipoda, Lysianassoidea, Uristidae) with a redescription of A. abyssorum (Stebbing, 1888) | 33 |
| 9496873c-3760-4f64-af01-c074d5fa312c | Saisie naturaliste opportuniste dans SICEN Occitanie - Données ANA - CEN Ariège opportunistes | 1 |
| 166d2036-e110-47f0-88a5-ef34b085e05a | A new species of the genus Protohyale Bousfield & Hendrycks, 2002 (Crustacea, Amphipoda, Hyalidae) from Covelong, Chennai, India | 3 |
| e0bee920-5c02-4c8d-8ac3-115a95f5a4b1 | Estudio de la comunidad zooplanctónica y fitoplanctónica en los ecosistemas de arrecifes coralinos mesofóticos del Parque Nacional Corales de Profundidad | 1 |
| abd3521c-f459-468e-9039-f989786147d4 | Natural Resources Wales Regional Data : Mid-Wales | 54 |
| b8866a8d-adc7-4a3a-887b-e59f830bb988 | INNS records held by BIS in Powys and Brecon Beacons National Park | 239 |
| c7399c30-eeda-4424-8194-dba370ea380c | FRA-PLANKTON DATASET | 1 |
| 4230db27-6316-44f7-96c9-165972014686 | Kelp Dataset | 10 |
| a00debff-435f-477d-a282-4699631d5f7a | Colección de Artropodos del Museo de Historia Natural Marina de Colombia - Makuriwa | 656 |
| e4fecf61-a790-4b0d-aaa1-6df408b57bd8 | Crustáceos macrobénticos de la plataforma y talud continental del Golfo de México | 204 |
| 7c010e32-f762-11e1-a439-00145eb45e9a | Crustacea in surface sediments off Sylt collected during HEINCKE cruise HE293 | 14256 |
| 7bfbe8b2-f762-11e1-a439-00145eb45e9a | Crustacea in surface sediments off Sylt collected during HEINCKE cruise HE275 | 14256 |
| 44bcde48-ac71-46f2-bf73-24fc3c008b6c | CAS Invertebrate Zoology (IZ) | 4816 |
| 6fbe0a8a-f762-11e1-a439-00145eb45e9a | Distribution of meiobenthos in sediment core M19_217-2 (2) of the northeastern Atlantic (Table 1) | 3 |
| 6fad8c5a-f762-11e1-a439-00145eb45e9a | Distribution of meiobenthos in sediment core M19_238-2 of the northeastern Atlantic (Table 1) | 4 |
| 6fa0bd18-f762-11e1-a439-00145eb45e9a | Distribution of meiobenthos in sediment core M19_193-2 of the northeastern Atlantic (Table 1) | 4 |
| 6fb18436-f762-11e1-a439-00145eb45e9a | Distribution of meiobenthos in sediment core M19_263 of the northeastern Atlantic (Table 1) | 4 |
| 6fb54846-f762-11e1-a439-00145eb45e9a | Distribution of meiobenthos in sediment core M23_123 of the northeastern Atlantic (Table 1) | 4 |
| 0b1150c9-dfa8-4904-8bc5-b79b4e1930e6 | FBIP: SeaKeys_SANBI: Marine images iSpot_2013 | 35 |
| 8bc4f5d2-8e7a-430d-b219-6776c38f401a | Caracterización de fauna y flora para el establecimiento de límites funcionales de humedales en tres ventanas piloto: Ciénaga de la Virgen, Ciénaga Zapatosa y Complejo de humedales Paz de Ariporo - Hato Corozal | 2 |
| 7025c72e-f762-11e1-a439-00145eb45e9a | Endofauna abundance in 2006-10 from an experiment in the Wadden Sea of List, Sylt | 20 |
| 711abdba-f762-11e1-a439-00145eb45e9a | Meiobenthos investigations in the Black Sea during Poseidon cruise POS317/3 | 8 |
| 6fff2df8-f762-11e1-a439-00145eb45e9a | Meiofauna abundances at the Arctic Håkon Mosby Mud Volcano (HMMV) of sediment core PS64/321-1 | 5 |
| 96165cbe-38aa-43fd-a11d-5b5346f05f10 | Inventaire de la réserve naturelle géologique de Saucats - La Brède - Inventaire entomologiques | 11 |
| d70c169f-fd9e-4355-8431-8d1fba91370f | MnhnL various project records, Recorder-Lux database | 70 |
| edd9b710-f3bb-4c49-9fa5-724cd4ecfc7d | Freshwater pelagic invertebrates ecological collection NTNU University Museum | 9 |
| f4004b04-bdcb-467f-9bbf-878b736a72ce | 2015_nsmk_smpl_ms | 150 |
| c8e4dc51-0611-47ec-8000-dea61108ceff | A new deep-sea genus and species of Eriopisidae (Crustacea: Amphipoda: Senticaudata) from the Gulf of Mexico | 1 |
| 82ea2cf6-f762-11e1-a439-00145eb45e9a | Southern Ocean Continuous Zooplankton Recorder (SO-CPR) Survey | 13873 |
| c7a3af38-7fbf-424f-841c-853108ff5f66 | Invertebrados y aves playeras de la Laguna Madre de Tamaulipas, México (Crustáceos) | 103 |
| 6fc88654-f762-11e1-a439-00145eb45e9a | The near-bottom plankton and benthic invertebrate fauna of Josephine and Great Meteor Seamounts (Table 2) | 30 |
| da5f7bf0-c72a-4087-b116-885fbdd10c03 | Bob Merritt invertebrate records for VCs 72-75 | 285 |
| db2cd6fc-a7d5-43ad-b944-8a663d57ae73 | Royal Belgian Institute of Natural Sciences Crustacea collection | 8000 |
| 2f63bb07-dbb6-4f9f-9507-ff75f77a4512 | Clare Biological Records Centre Dataset 2004-2007 | 3 |
| cfb98735-6065-42fa-8613-fce70d39cbbe | BioGIS - Marine snails - HUJ collections | 2 |
| b635be2e-76ea-4600-8f83-549601653c0a | The Belgica 121 expedition to the Western Antarctic Peninsula: a high resolution biodiversity census | 149 |
| 5318e238-18f5-11e2-98fb-00145eb45e9a | Type Collection of the Seto Marine Biological Laboratory, Kyoto University | 13 |
| 7ee7849b-b7d8-429f-a8a6-1f3787ce7262 | A new species of the genus Elasmopus from Miyako Island, Japan (Crustacea: Amphipoda: Maeridae) | 1 |
| ef7e0d45-c59f-4f48-afa3-53d9f545de56 | St Andrews BioBlitz 2015 | 4 |
| 241f9bd4-613e-4050-9fe0-2ff44b526c84 | St Andrews BioBlitz 2014 | 1 |
| 3fc4ed1b-9026-47a9-857f-f6543c444874 | Megafauna at the Haakon Mosby mud volcano (the Barents Sea) based on image analysis. | 34 |
| 37497bf7-31a0-4fd5-9591-2b47004f5ab9 | Deep-sea megabenthos of the Eurasian Central Arctic based on image analysis. | 55 |
| 5d283bb6-64dd-4626-8b3b-a4e8db5415c3 | Essig Museum of Entomology | 18 |
| ffe3fc6c-85c8-48cc-a261-b98773e34052 | Ferme pilote d'éoliennes flottantes de Groix & Belle-Ile et son raccordement au Réseau Public de Transport d'Electricité - Campagne benthos subtidal Drague2015-Raccordement du parc éolien Groix/Belle-île | 29 |
| 2ac4b2f0-9887-4d25-a8fa-f94391e87919 | DASSH Data Archive Centre Academic Surveys | 3287 |
| 686c1b9b-5f29-4e1f-8c28-f9779d264579 | Porcupine Marine Natural History Society Dataset | 901 |
| 11314af8-aad0-4414-a961-e91b88fd4abb | DASSH Data Archive Centre volunteer sightings records | 8 |
| 70b7c7b1-fbea-4771-8b63-ee1c7fae393d | Scottish river macro-invertebrate records from 2007 collected by SEPA | 305 |
| 4d749d70-e2e1-11dd-8102-b8a03c50a862 | Rapid Assessment Program (RAP) Biodiversity Survey Database | 7 |
| ea34233a-9ba5-4022-ad7c-78eb11687693 | Confirmation of Caprella scauroides Mayer, 1903 (Crustacea: Amphipoda) from New Zealand, using integrative techniques | 4 |
| 83ae84cf-88e4-4b5c-80b2-271a15a3e0fc | Auckland Museum Botany Collection | 2 |
| 968dfa3e-3614-4bab-a3b5-e9209bdd6078 | Ocean Genome Legacy Collection | 603 |
| 129704b4-762b-4f0c-8eab-ef3f8aedd478 | Computarización de la Colección Nacional de Crustáceos del Instituto de Biología, UNAM y elaboración de su catálogo | 169 |
| b4804f19-8a8a-49e7-8dc2-79b528635696 | Mesozooplankton Ramfjord | 3 |
| 2bcc960c-aa93-4997-9432-e4945c6345f0 | Regional Museum of Lapland (LMM) | 1 |
| 7b573d12-767c-4d2c-a434-651c4e7764e8 | Base de données faune - DREAL Centre-Val de Loire. Polygones | 2 |
| b4443d83-635f-4dd8-a311-46992c0f17cf | Numérisation des données faune contenues dans les Bulletins de la Société Linnéenne de Bordeaux - Bulletin de la Société Linnéenne de Bordeaux, Tome 146 (N.S) n° 39 (4), 2011 - Données faune | 70 |
| dd620b3c-e193-4767-8452-8541deabd688 | Projet de ferme pilote d'éoliennes flottantes du golfe du Lion et de son raccordement électrique - Etat initial benthique et sédimentaire | 24 |
| d59ae05c-bf84-44aa-9753-d4acf40a9bb1 | Biodiversidad selecta de los humedales de Laguna de Términos - Pantanos de Centla (Crustáceos) | 53 |
| 8670893a-08ad-4735-8c18-eb088443ccd6 | Museu Darder de Banyoles | 1 |
| b96aa772-0701-4b75-a308-f3ff755bb749 | Review of the hyperiidean amphipod family Lycaeidae Claus, 1879 (Crustacea: Amphipoda: Hyperiidea) | 47 |
| 4c0b59ec-84a8-4daa-8467-3ac34e86ecd6 | The Tryphosa group (Crustacea: Amphipoda: Lysianassoidea: Lysianassidae: Tryphosinae) | 62 |
| ca4df07d-4d10-402a-8d2f-3c8a1fdf45c0 | Analysis of macrobenthos in the Southern Bight of the North Sea (1971-1972) | 201 |
| 56687a3d-b48f-44e5-b2b8-d55bafff2f33 | Galiano Island BC Canada Marine Zoology 1893–2021 | 23 |
| fad80260-3167-49ea-b797-87292773a923 | Phylogenetic analyses of a new freshwater amphipod reveal polyphyly within the Holarctic family Crangonyctidae, with revision of the genus Synurella | 5 |
| 577e64c7-be69-4866-906b-343e28880c9d | Hyalella luciae (Crustacea, Amphipoda, Hyalellidae) - a new species of freshwater amphipod from Southern Brazil | 2 |
| 280c1f6a-219f-4994-bb94-35bec5bed4a8 | Fauna críptica del Bajo de las Ánimas, Caribe colombiano | 4 |
| 6f7c2191-a210-4685-98f6-7a90241131df | New genera for species of Jassa Leach (Crustacea: Amphipoda) and their relationship to a revised Ischyrocerini | 21 |
| 7e8caa2f-e2ba-45ba-bc22-357dbbf822d0 | Dorotea gen. nov., a new bathyal genus (Amphipoda, Eusiridae) from the Solomon Sea (Papua New Guinea) | 1 |
| 4e11d715-c5ac-4669-a901-86e3852b11ed | Diversity and spatial distribution of Phyto and Zooplankton in the West Coast of Madagascar. | 5 |
| 8a8a0ee8-f677-41db-9ff5-9296d1c456c3 | Morphological comparison and description of five new species of Hyalella (Crustacea: Amphipoda) from Veracruz and Mexico City | 12 |
| fc228778-e408-49cf-aed3-73078dac29b0 | Metarhachotropis parva, a new genus and species of Eusiridae (Crustacea: Amphipoda) from Sagami Bay, central Japan | 1 |
| 1e39712e-c308-4250-8ad2-663d5932cc9c | Pilbarana, a new subterranean amphipod genus (Hadzioidea: Eriopisidae) of environmental assessment importance from the Pilbara, Western Australia | 6 |
| af7d48d4-492a-4f7d-9ae2-929ae6dbe1dd | First record of the genus Eriopisella Chevreux, 1920 (Crustacea, Amphipoda, Senticaudata, Eriopisidae) from Australia, with the description of a new species, Eriopisella moretoni sp. nov. | 2 |
| 87920495-c1bf-4bdb-b153-50e709c1d6c2 | Epimeria of the Southern Ocean with notes on their relatives (Crustacea, Amphipoda, Eusiroidea) | 26 |
| 7f89b5f5-ce82-436b-9d27-ec9ba6540198 | Données Faune de l'Agence des Espaces Verts (AEV) d'Ile de France | 18 |
| 727232c0-d4a6-4959-b04d-3ae146281fd8 | Thaumatelsonine Stenothoids (Crustacea, Amphipoda). Part 2 | 18 |
| bb72e906-c382-481e-b5ef-0fd361f38777 | A review of Quadrimaera Krapp-Schickel & Ruffo, 2000 (Amphipoda: Senticaudata) from Brazil | 11 |
| 1dcfb37e-2288-4105-80d5-530e2c98d2a4 | Système d'Information sur la Nature et les Paysages d'Ile de France - Données sensibles (ou cachées par les observateurs) de la base de données Île-de-France | 1 |
| 44ef21b3-550b-4f82-950c-3d7db7e04de0 | Species of the Maera - clade collected from Japan. Part 3: genera Maera Leach, 1814, Meximaera Barnard, 1969 and Orientomaera Ariyama, 2018 (addendum), with a key to Japanese species of the clade (Crustacea: Amphipoda: Maeridae) | 3 |
| b2800bf3-5970-45b8-a4fc-d513fc7c111c | Gammaropsis elvirae sp. nov., a widely distributed amphipod (Amphipoda: Photidae) in the Yucatan Shelf, with ecological comments and a key for the genus in tropical America | 4 |
| 9005a5e2-b2dd-42db-aaa4-b17b8077988b | A new species of Ceradocus Costa, 1853 (Senticaudata: Maeridae) from Ceará State, Northeastern Brazil | 4 |
| b5314e83-212b-4985-a80b-8dac61a991a5 | Stygobromus bakeri, a new species of groundwater amphipod (Amphipoda, Crangonyctidae) associated with the Trinity and Edwards aquifers of central Texas, USA | 2 |
| bc28e8d7-56ea-4318-86bc-834a0fdb87c8 | Morphology and zoogeography of the burrower-like gammarid Gammarus koshovi (Bazikalova, 1946) (Crustacea, Amphipoda, Gammaridae) - An overlooked and poorly known species in the Siberian fauna | 8 |
| a94a502d-d301-4b1f-bc9e-4494405eaa96 | A new genus and species of sand-hopper, Mauritiorchestia fayetta gen. nov., sp. nov (Amphipoda, Talitridae) from Mauritius | 3 |
| e35fa951-3db7-4b72-a9a9-f06fc4507577 | Amphipod crustaceans from Chilean Patagonia | 1 |
| 68f31503-d38a-4c39-9774-a7802bb3fe09 | Four new species of Lysianassidae Dana, 1849 (Crustacea: Amphipoda) From Northeastern Brazilian coast | 3 |
| 463ea041-e53f-4bd9-954b-c34f8806fcf5 | Sea-land transition drove terrestrial amphipod diversification in East Asia, with a description of a new species | 3 |
| 368ee07f-340b-47b1-a0d0-de421fe309d1 | New species of Eurythenes from hadal depths of the Mariana Trench, Pacific Ocean (Crustacea: Amphipoda) | 3 |
| 6f0d0430-b719-4a2e-9bb0-20b35ba4bc6c | Data on the biodiversity of macrophyte communities and associated aquatic organisms in lakes of the Vologda Region (North-Western Russia): algae and invertebrates | 52 |
| 9a0b66df-7535-4f28-9f4e-5bc11b8b096c | Waarnemingen.be - Non-native animal occurrences in Flanders and the Brussels Capital Region, Belgium | 297 |
| 3ff96650-b8c7-40cb-a557-3f4e7a9d235d | Species of the Maera-clade collected from Japan. Part 4: addenda to genera Maera Leach, 1814 and Quadrimaera Krapp-Schickel & Ruffo, 2000, with revised keys to Japanese species of the clade (Crustacea: Amphipoda: Maeridae) | 6 |
| dfd5f8fe-abf4-4089-a326-fd97e5669d4b | A new deep-sea species of Vemana J. L. Barnard, 1964 (Amphipoda, Amphilochidea, Vemanidae) from off southern Gulf of Mexico | 1 |
| 96cdea48-b78a-4345-ae6d-b2c7c6e0278a | NCSM Non-molluscan Invertebrates Collection | 30 |
| 468ad787-70bb-4280-9ae7-cf83eaf664b3 | A new species of Pleonexes Spence Bate, 1857 (Amphipoda: Senticaudata: Ampithoidae) from the São Pedro and São Paulo Archipelago, Equatorial Atlantic, Brazil, with comments on the genus | 4 |
| 0c11c0fd-d069-4a1a-9533-202ae884b6e0 | A new amphipod species of the bathyal genus Dautzenbergia Chevreux, 1900 (Amphipoda, Calliopioidea, Pontogeneiidae) associated with cold-water corals off Angola | 4 |
| ecb6492c-9469-4fbf-a42d-97e75d5403c0 | Turcogammarus aralensis (Uljanin, 1875), a relict pontogammarid amphipod crustacean from the Aralo-Caspian paleobasin: redescription, phylogenetic position and biogeography | 2 |
| 84ed08a5-7d5b-4c0c-9de6-5185dd318e1f | Sistema de Monitoreo de Arrecifes Coralinos - SIMAC: Estructura Coralina | 2 |
| 5378e1cf-522d-4469-8776-b709579b4a3e | Recent Invertebrates Specimens | 1 |
| ce990d03-43e2-418e-9b42-4b7ba88535d7 | Maerid amphipods (Crustacea: Amphipoda) from Okinawa, Japan with description of a new species | 1 |
| 4e8e833c-f497-435b-abd1-835cbe5dd94b | A new species of the genus Rhachotropis from off Amamioshima Island northwestern Pacific (Crustacea: Amphipoda: Eusiridae) | 2 |
| 5727b209-20b0-4e6a-906c-0eeff346ea92 | Description of Ptilohyale corinne sp. nov. (Amphipoda: Hyalidae) from the Bahía Blanca estuary, Argentina, including a key to all valid Ptilohyale species | 3 |
| 9f0cd0ac-792a-4566-8880-b61e0125a5ad | Two species of Ceradocus collected from coastal areas in Japan, with description of a new species (Crustacea: Amphipoda: Maeridae) | 4 |
| 0df60ac3-92fe-491e-ba26-40d2db665a4e | Talitrus saltator (Montagu, 1808), a species complex (Amphipoda, Senticaudata Talitroidea, Talitridae) | 3 |
| 30736dc0-0f81-4770-89ee-268c23441052 | A new species of sponge-dwelling amphipod, Polycheria spongoteras sp. nov., from Spirits Bay, Northland, New Zealand | 2 |
| e74c14a1-adcd-492c-8d1f-5208568d14d5 | Maxillipiidae * | 1 |
| 8531183a-86b5-459b-a93e-37198f38f8a6 | Biome of Australia Soil Environments | 171 |
| d6097f75-f99e-4c2a-b8a5-b0fc213ecbd0 | University of California Santa Barbara Invertebrate Zoology Collection | 13 |
| 63f2406a-79b0-42e6-87bd-322f5e8e7285 | Two new Gammarus species and a new name (Crustacea: Amphipoda: Gammaridae) from Northwest China | 2 |
| 005eb8d8-ed94-41be-89cf-e3115a9058e4 | Field Museum of Natural History (Zoology) Invertebrate Collection | 438 |
| d32c5586-3a61-4208-bdd3-bd24f41c90c5 | Eusirus bonnieri sp. nov. (Crustacea: Amphipoda: Eusiridae), a new deep species from the southeastern Bay of Biscay (NE Atlantic Ocean) | 4 |
| 7ad954d2-0a0d-4ad5-934c-62d5437e6c32 | An annotated checklist of the Niphargidae (Crustacea: Amphipoda) of Greece | 4 |
| fc57b226-5665-435b-9e37-5ce9b6d13df5 | Pleonexes Spence Bate, 1857 (Amphipoda: Ampithoidae) in the Persian Gulf and the Gulf of Oman | 4 |
| fd26a7a9-6f28-4c88-9915-a9cc9109f4c9 | Macroinvertebrados bentónicos de la cuenca hidrográfica del río Garagoa, macrocuenca del río Orinoco, Boyacá, Colombia | 4 |
| 4578c19d-be07-4d79-bf26-757a74e247d2 | CardObs : Observations naturalistes issues de l'outil de saisie et gestion CardObs mis en place par le Service du Patrimoine Naturel (MNHN) / UMS PatriNat (OFB - CNRS - MNHN) - Contribution de la Ligue Insulaire Spéléogique de Corse à l'inventaire des arthropodes cavernicoles de Corse, dans le cadre de l'appel à projets INPN 2019 | 3 |
| 1bb9e78a-0f20-4335-8c36-1c4683e48a55 | Projet d'interconnexion électrique France-Irlande "Celtic Interconnector" - Inventaire des peuplements benthiques 2019 sur l’AEI « Celtic Interconnector » | 365 |
| 811bed92-f762-11e1-a439-00145eb45e9a | Ibaraki Nature Museum, Arthropoda collection | 18 |
| c0b05870-a4b6-41a1-ad52-30fa31904e7f | New Antarctic stenothoids sensu lato (Amphipoda, Crustacea) | 1 |
| 16de4a1e-812a-4cd1-bf5b-0962b94094d5 | Complement to the knowledge of the Haploops species (Crustacea, Gammaridea Ampeliscidae), with the description of two new species from North Atlantic Ocean [Contribution to the knowledge of the Haploops genus. 10.] | 15 |
| b74e6677-e056-459b-8781-3f3ade783f8c | A new Gammarus species (Crustacea, Amphipoda, Gammaridae) from Northwestern Islands, South Korea | 3 |
| 86939734-f762-11e1-a439-00145eb45e9a | Marine invertabrate(ARTHROPODA) specimen database of Osaka Museum of Natural History | 89 |
| b1a9a24a-3669-43ea-890b-7da58b7a96a6 | Five species of the family Odiidae (Crustacea: Amphipoda) collected from Japan with descriptions of a new genus and four new species | 11 |
| be859ab2-245b-4477-b770-108671f5bb1b | A novel species of Heterophoxus Shoemaker, 1925 (Crustacea, Amphipoda, Phoxocephalidae) from southeast and southern Brazil, with an identification key to world species of the genus | 8 |
| b18eba44-3193-4436-a6b0-75ee3ea54875 | Community Foundation for Ireland Records | 12 |
| 6ebf9dcb-db2d-4e08-807c-43f5d55392a4 | Two new Amphilochida (Amphipoda: Amphilochidea) associated with the bioinvasive Tubastraea coccinea fromTodos-os-Santos Bay, Bahia State, Brazil | 4 |
| e56bac47-d0c1-4850-9975-75f3c9c55588 | A new superfamily and family of Hadziida (Amphipoda: Senticaudata), with a description of a new genus and new species from the Brazilian continental shelf | 1 |
| 34162c15-e5a8-4085-9bfd-be4dc280d20e | New tryphosine amphipods from Australian waters (Crustacea, Amphipoda, Lysianassoidea, Lysianassidae, Tryphosinae) | 136 |
| 084d8de1-dab3-4d6e-9db0-69e853c39709 | Pelagic amphipods (Crustacea: Amphipoda: Hyperiidea) in western Mexico. 6. Superfamily Vibilioidea. Families Paraphronimidae and Vibiliidae | 73 |
| b3ab1acc-5ff9-4152-9dcc-3b4d127b599b | A new species of Paraproto (Crustacea: Amphipoda) from southern New SouthWales, Australia | 4 |
| 64aa8730-a90c-4c26-aea9-9e7dff068dc1 | The competition of native sponges and the sun coral Tubastraea spp. does not influence the morphological pattern of a new Photis (Photidae: Senticaudata) | 16 |
| 6df87eb2-cc20-4868-ad50-a621a5314bd9 | Niphargus dancaui sp. nov. (Amphipoda, Niphargidae) - a new species thriving in sulfidic groundwaters in southeastern Romania | 14 |
| 482193ad-9476-4aa0-bd03-1001e6156506 | Two new species of Grandidierella (Amphipoda, Corophiida, Aoridea) from Singapore | 6 |
| f2fbdc01-4a1f-4043-b4aa-8458fc490f04 | A new species of Cephaloecetes (Bubocorophiina) from the Iranian coasts of the Gulf of Oman and the Hormuz Strait (Crustacea: Amphipoda: Siphonoecetini) | 2 |
| 6263881d-c36c-403b-a0d8-29c69da782d2 | Numérisation des données faune contenues dans les Bulletins de la Société Linnéenne de Bordeaux - Bulletin de la Société Linnéenne de Bordeaux, Tome 144 (N.S) n° 37 (3), 2009 - Données faune | 12 |
| c3370dbd-320c-40f4-9708-389231e1a1f8 | Caprellidae (Crustacea: Peracarida: Amphipoda) from deep-sea waters off Galicia (NW Iberian Peninsula) with the description of a new genus and three new species | 17 |
| c1ec7d18-049a-493a-91fe-fab34dd2cc4c | A new data of freshwater amphipod of genus Niphargus Schiödte, 1849 from Lorestan Province in Iran | 1 |
| abf29a92-f6c5-4ddd-90f1-6987f11e2914 | Liaison autoroutière concédée entre Machilly et Thonon les Bains et suppression des passages à niveau N° 65 et 66 à Perrignier Haute-Savoie - Crustacés - Inventaire non standardisé - FRAPNA74 - 24HNAT | 2 |
| d7b7d634-c4a3-4e4d-b919-a502df02112f | Exploring the diversity of the deep sea-four new species of the amphipod genus Oedicerina described using morphological and molecular methods | 10 |
| 3ca0710c-d484-4e32-805a-3c6281d1ef6e | Siphonoecetini Just, 1983 (Crustacea, Amphipoda, Ischyroceridae) 13: Western Australian species of Bubocorophiina in Rhinoecetes, Cephaloecetes, Sinoecetes, Borneoecetes and Pararhinoecetes gen. nov. | 42 |
| dede68a0-5291-4c06-b3f7-4f41cf1295e0 | Species of the Maera - clade collected from Japan. Part 2: genera Austromaera Lowry & Springthorpe, 2005 and Quadrimaera Krapp-Schickel & Ruffo, 2000 (Crustacea: Amphipoda: Maeridae) | 7 |
| e1990f2e-89e4-476e-8407-8d736c02f47a | A new species of Quadrivisio (Amphipoda, Maeridae) from coastal tropical lagoons (Benin, West Africa) | 4 |
| e16432db-82d6-4286-9271-5c9902dcc516 | Three new species of Colomastix Grube, 1861 (Amphipoda: Colomastigidea) from Todos-os-Santos Bay, northeastern Brazilian coast, with identification keys to Atlantic Ocean species | 26 |
| 0b0cf968-955f-4872-951e-fab60e0cad10 | Four new species of Pseudharpinia Schellenberg, 1931 (Crustacea: Amphipoda Phoxocephalidae) from southwestern Atlantic and new records of P. tupinamba Senna & Souza-Filho, 2011 | 4 |
| 6cceabcf-a2f2-4b6b-84f8-e6447683e1a1 | Talitrid amphipods from India, East Africa and the Red Sea (Amphipoda, Senticaudata, Talitroidea, Talitridae) | 10 |
| 129e5856-650a-49bf-a46a-0e3c6c0d2011 | Macroinvertebrados acuáticos asociados a la red vial secundaria en Medellín | 1 |
| ffdbb934-3505-49ef-9af9-2619082e4d1c | NHMD Invertebrate Zoology Collection | 1093 |
| 403cea9f-80d5-48f4-ae41-97cc9a157a62 | Programme de rééquilibrage du lit de la Loire entre Les Ponts de Cé et Nantes - Données naturalistes brutes - Programme de rééquilibrage du lit de la Loire | 2 |
| 58fc397b-eee4-4138-8a5f-50f9dfb00216 | Invertebrates excl. Entomology at the Natural History Museum of Denmark | 1001 |
| d731839a-3f1b-4af5-bba9-6495ecade64e | ZFMK Crustacea collection | 131 |
| 6ed43f52-25d1-4d56-a821-63a8564b81f6 | Référentiel taxonomique national (TAXREF) - Inventaire des Collectivités d'outre-mer et de la métropole (ICOM_14) issu des données biogéographiques de TAXREF v14 | 93 |
| fda395aa-63c9-4b98-99e4-8d37651aa4f5 | SILENE-FAUNE-PACA - DREAL_PACA_2017_12_18 | 1031 |
| d0d1b5f8-695a-4b08-a345-439d77b9902e | Lothian Wildlife Information Centre surveys (Invertebrates - general) | 9 |
| fbf97019-92f1-430b-8419-6aaf68343317 | Grupo de Macroinvertebrados de la laguna Cristalina Páramo de la Rusia-Boyacá. | 1 |
| 13de2146-3a79-4c6f-a87c-6ac85a95c5d4 | Comunidades hidrobiológicas de los tributarios principales de la cuenca del lago de Tota, Boyacá | 8 |
| bef19e99-90a7-4b3d-a113-bdf4ea6c88f2 | mabik_cr | 19149 |
| c8cb5bae-0957-4726-9c5a-e1f81219bd7c | Registros de macroinvertebrados acuáticos presentes en la Laguna La Virginia, páramo del Sumapaz | 1 |
| 76ef1883-c32a-49bb-a36c-2752af1b4e95 | Abundance of zooplankton during the polar night (cruise in January 2017) at 13 stations using a 64um-mesh Multinet | 8 |
| 7e8caed5-bf17-43a5-b0f6-bfb71a489336 | Nottinghamshire Wildlife Trust - Records | 2 |
| e49eb802-fd8f-4c6a-9eaf-aec9bcbf45d7 | Ferme pilote d'éoliennes flottantes de Groix & Belle-Ile et son raccordement au Réseau Public de Transport d'Electricité - Campagne benthos Rocheux2015-Raccordement du parc éolien Groix/Belle-île | 21 |
| a8035a1d-e674-4d2a-bb59-b476af6a3d6d | American Museum of Natural History (AMNH) Crustacea Collection | 284 |
| 1cd10854-e401-4822-ae48-adaeeedc647d | Northern Ireland Environment Agency (NIEA) Collated Species Records | 76 |
| 9fad752d-4bf9-4adf-9535-3cf6339a70fb | Department of Agriculture Environment and Rural Affairs (DAERA) Marine and Fisheries Division Marine Survey Data | 23233 |
| f47f6839-9203-44ee-87f6-7ad047d8430d | Northern Ireland Environmental Recorders - Marine Species Records | 19 |
| d0cee8f6-dbf9-4355-a9c2-62ab51fe36fd | Invasive Species and Pests | 162 |
| f3f169bb-3cd8-4a67-b347-f0776c53f3e5 | Coastwatch Marine Species Records from Northern Ireland | 13 |
| 29c713af-3f97-4c0a-bb6d-c09c1cef5f72 | Queens University Belfast Marine Research Data | 2 |
| 11ddb6e5-7bc8-4478-9e80-5c85c1a08ae2 | Consultancy Contract Surveys - Marine Species Records | 378 |
| 69bc8993-33e4-4a34-985b-d73d70a88c6d | Centre for Environmental Data and Recording (CEDaR) Marine Species Data | 13 |
| 60de59a4-c428-4d49-873d-b117bea1fcc9 | Ulster Museum Marine Surveys | 285 |
| 84f58d09-add5-469b-af6e-f3522a8dac7d | CEDaR Online Recording | 30 |
| a2a36824-70bb-431a-a5a4-1f848e5cc9b3 | Agri-Food and Biosciences Institute Marine Surveys | 1134 |
| 70921b14-8ee5-47a0-aea4-679ac21e8196 | Collated IRTU Freshwater Surveys | 8257 |
| 8d7bb962-d8ed-4318-8f45-d4b5248802e2 | BD de la plateforme partagée pour la diffusion des données naturalistes de Normandie - Centralisation des données d'études sur le territoire de la Communauté de Communes de la Côte d'Albâtre | 3 |
| ee9eb6e3-f770-4af9-962a-625a7235a449 | Colección Entomológica de Piedras Blancas | 1 |
| 4fc7684f-d538-4be5-b2ff-848c6bc47905 | Projet de raccordement au réseau public de transport d'électricité du parc éolien en mer de Dieppe - Le Tréport - Campagne Benthos Benne et Drague 2015-2016 - Projet de raccordement du DLT | 197 |
| 6cd1212c-66ba-40ee-9295-14aee7339dd0 | Colección Macroinvertebrados Acuáticos Universidad Católica de Oriente | 4 |
| 716f686c-a13e-4975-9076-7ee9f59bb4a3 | Système d'Information sur la Nature et les Paysages d'Ile de France - Données du naturaliste PROVOST romain provenant de la base de donnée du SINP Île-de-France CETTIA | 1 |
| 61313732-a660-4fdf-9cfb-0d13d4553bbe | iRecord Surveys | 5 |
| b94cbceb-76c9-4a0a-a752-caa92d6613cf | Système d'Information sur la Nature et les Paysages d'Ile de France - Inventaire éclair de Natureparif - Année 2015 | 1 |
| 583d91fe-bbc0-4b4a-afe1-801f88263016 | INSDC Environment Sample Sequences | 7 |
| 4939f5ba-e740-4f16-87fc-e2eac9b26cab | Système d'Information sur la Nature et les Paysages d'Ile de France - Données de la structure Département 77 provenant de la base de donnée du SINP Île-de-France CETTIA | 4 |
| 97d01261-0027-4e89-a0d2-4a7d700f5fdb | Verified marine records from Indicia-based surveys | 175 |
| c249ca5e-ef81-4687-a6fb-80520a048293 | Système d'Information sur la Nature et les Paysages d'Ile de France - Données de la structure Ville de Paris provenant de la base de donnée du SINP Île-de-France CETTIA | 2 |
| e9f5955b-0d3e-4560-b289-d1f26b3875be | Système d'Information sur la Nature et les Paysages d'Ile de France - Données de l'association RENARD provenant de la base de donnée du SINP Île-de-France CETTIA | 9 |
| 1c599438-5abd-484f-8deb-d0263dec510f | AquaInvaders | 9 |
| 68ad7ec2-0f89-4d32-9ad2-650c88899eb8 | Yorkshire Wildlife Trust - Non-sensitive records from all taxonomic groups | 9 |
| 732b8816-1367-4cb2-80f9-343063ed3442 | Système d'Information sur la Nature et les Paysages d'Ile de France - Données de la structure Naturessonne provenant de la base de donnée du SINP Île-de-France CETTIA | 1 |
| fc7897c8-5dcd-48d4-859d-3ea3d3217c0b | Système d'Information sur la Nature et les Paysages d'Ile de France - Données de la structure Fédération 77 de Pêche et Protection du Milieu aquatique provenant de la base de donnée du SINP Île-de-France CETTIA | 2 |
| 8de4fda9-edee-4060-a131-5dd9984c8cb5 | Non Native Species Across Wales (1989 to 2012) | 485 |
| 3dc2a7f6-8d3a-4228-a69c-ec795028fe2f | Miscellaneous records held on the Cofnod database | 54 |
| 9b65cb62-96b8-4809-84f0-511134fa03d9 | Invertebrates (except insects), Outer Hebrides | 14 |
| fe730480-cad3-415e-804c-1c2ad80d3859 | Marine Sightings & Miscellaneous Species Records from Natural Resources Wales (NRW), 2007 onwards | 1 |
| b66c629e-b878-4b26-960d-7c0103b025c7 | Marine Non Native Species records from Natural Resources Wales (NRW) Monitoring Research and Ad-hoc Sightings | 15 |
| e91e4a74-3e9b-4a40-af23-6c7f287b1276 | Zooplankton community of Shirazi bay (Kenya) sampled between April and December 1998 | 18 |
| 0c063e90-7fb2-43d2-b200-e7c68a9ef57d | Two new species of groundwater amphipods of the genus Niphargus Schiödte, 1849 from northwestern Iran | 4 |
| b7def985-a35c-4b2d-9b5c-7e48f9fd8751 | CardObs : Observations naturalistes issues de l'outil de saisie et gestion CardObs mis en place par le Service du Patrimoine Naturel (MNHN) / UMS PatriNat (OFB - CNRS - MNHN) - Données naturalistes de Monsieur Pratz Jean-Louis | 13 |
| 6df4cdef-c55d-4a03-9055-3cbadcdd05aa | DASSH Data Archive Centre volunteer survey data | 34 |
| a8c38b89-47ff-4e0e-b16e-646899d1d3fd | Système d'Information sur la Nature et les Paysages d'Ile de France - Données d'observateurs divers (observateurs transmettant un nombre de données peu élevé) saisies dans Cettia-idf | 3 |
| b596f384-6cd7-4a48-92a5-cf8847877262 | Evaluación hidrobiológica para el proyecto Minera de Cobre Quebradona, municipio de Jericó | 4 |
| dc658318-8a2e-4faa-87e2-7f6a501ff16d | "Biodiversidad en el valle de Cuatro Ciénegas". (Peces) | 1 |
| 7e6df30d-d8af-438d-bb01-5a528f6f4fa3 | Programme CarNET B (Cartographie Nationale des Enjeux Territorialisés de Biodiversité Remarquable) - Carnet B Centre - lot 2012 | 4 |
| 72a17f09-ceaa-4420-aff3-3bb99564774e | Programme CARTHAM: Inventaire biologique dans le cadre de Natura 2000 en Mer | 2754 |
| 378264b9-9622-4dc0-9b18-b39818c8898e | Base BOMBINA du Parc Naturel régional Lorraine - Données bibliographique de la base BOMBINA | 38 |
| 4bfac3ea-8763-4f4b-a71a-76a6f5f243d3 | Museum of Comparative Zoology, Harvard University | 2006 |
| e6300f6f-987b-4b33-983d-cfe1e1b9e002 | Trawl Data from the CCGS Sir John Franklin during the 2022 International Year of the Salmon Pan-Pacific Winter High Seas Expedition | 1 |
| 084feec5-77dc-4fad-9037-42880f2a3214 | Trawl Catch and Species Abundance from the 2020 Gulf of Alaska International Year of the Salmon Expedition | 1 |
| 8050237e-f762-11e1-a439-00145eb45e9a | Biodiversidad de crustáceos dulceacuícolas del centro de Nuevo León y noroeste de Tamaulipas (R53, Río San Juan y Río Pesqueria) | 70 |
| 1a2902fe-ea89-4f91-bfb8-c3c8e4c724ca | RACCORDEMENT ELECTRIQUE DE LA FERME EOLIENNE FLOTTANTE DE GROIX ET BELLE-ILE - Création de la liaison sous-marine et souterraine à 63 000 volts - Campagne benthos Intertidal 2017-Raccordement du parc éolien Groix/Belle-île | 7 |
| 523ffd45-1baf-4f8b-b2a2-7b97e293a27e | Study of the biotic environment in the Sluice Dock in relation to oyster farming between 1960 and 1964 | 7 |
| bdad918c-470b-4c4e-a146-6a39caacf989 | Formación de una base de datos de la biodiversidad de fauna marina y costera en el Golfo de California | 388 |
| ce861efd-402f-4c10-a9fb-30ad0426bd15 | Données de la plateforme régionale SINP DREAL Bourgogne - Données de l'Observatoire de la Faune de Bourgogne | 3 |
| 41c4b3e1-0a28-45f4-9f6c-09a7f77c184a | Algas marinas bentónicas de la Península de Yucatán y uso potencial de especies selectas | 5 |
| 43d13435-60f0-421e-860a-6edaeafd79b2 | Macrobenthos monitoring in function of dredge disposal monitoring in the Belgian part of the North Sea | 1313 |
| ca435754-9190-4a87-9b2c-c21563898294 | CLICNAT- Base de données naturaliste picarde - CLICNAT Base de données naturaliste picarde gérée par Picardie Nature | 7 |
| 16cea338-38b9-47f8-bde1-5bf4a613ea3d | Base de données faune - DREAL Centre-Val de Loire. Points / polylignes | 3 |
| ae16463e-7747-499b-ae73-957c618ed99a | Ferme pilote d'éoliennes flottantes de Groix & Belle-Ile et son raccordement au Réseau Public de Transport d'Electricité - Campagne benthos Intertidal 2017-Raccordement du parc éolien Groix/Belle-île | 7 |
| 02743f84-b52a-433f-8482-f54bc86014b7 | CardObs : Observations naturalistes issues de l'outil de saisie et gestion CardObs mis en place par le Service du Patrimoine Naturel (MNHN) / UMS PatriNat (OFB - CNRS - MNHN) - Données naturalistes de Monsieur DAMOISEAU Sébastien | 1 |
| 8c9ced05-6826-4f05-80b5-f9fa7ceb1833 | Bibliographie de la faune, la flore et la fonge de France métropolitaine et outre-mer - Revues diverses et littérature grise. | 47 |
| 5ed89718-b60a-4e63-868a-537d571b7660 | Ferme pilote d'éoliennes flottantes de Groix & Belle-Ile et son raccordement au Réseau Public de Transport d'Electricité - Campagne benthos Benne2015-2017-Raccordement du parc éolien Groix/Belle-île | 171 |
| d08ea09c-6403-4a2f-bd63-c6906bec5e50 | CardObs : Observations naturalistes issues de l'outil de saisie et gestion CardObs mis en place par le Service du Patrimoine Naturel (MNHN) / UMS PatriNat (OFB - CNRS - MNHN) - Données naturalistes de Benoit LECAPLAIN | 2 |
| 3e697abc-7847-43be-a882-16414ece89a5 | RACCORDEMENT ELECTRIQUE DE LA FERME EOLIENNE FLOTTANTE DE GROIX ET BELLE-ILE - Création de la liaison sous-marine et souterraine à 63 000 volts - Campagne benthos subtidal Drague2015-Raccordement du parc éolien Groix/Belle-île | 29 |
| 71d86de3-76f2-4db4-a448-416e52f484fb | RACCORDEMENT ELECTRIQUE DE LA FERME EOLIENNE FLOTTANTE DE GROIX ET BELLE-ILE - Création de la liaison sous-marine et souterraine à 63 000 volts - Campagne benthos Benne2015-2017-Raccordement du parc éolien Groix/Belle-île | 171 |
| d3130e9c-8587-4545-baea-6e72876bb50c | Programme CarNET B (Cartographie Nationale des Enjeux Territorialisés de Biodiversité Remarquable) - Carnet B Lorraine | 193 |
| 2e409ff6-7714-417e-89ef-678207f66071 | ATBI Parc national du Mercantour / Parco naturale Alpi Marittime - Jeux de données provenant de l'ATBI Mercantour | 17 |
| 663bae95-7134-4cb3-9085-e9c4afbf8fb0 | Données Faune Base SIRFF - FNE Centre-Val de Loire - Système d’Information Régional sur la Faune et la Flore - FNE Centre-Val de Loire - Données saisies entre 01-01-2017 et 31-03-2018 | 18 |
| 4459ca07-9aa7-4c79-8179-0837ce758127 | Projet éolien en mer de Dieppe Le Tréport - Etat initial benthique et sédimentaire | 824 |
| adac482f-a6d8-4873-94fe-acec052b92b7 | Apoyo a las colecciones biológicas de la Facultad de Ciencias de la UNAM: Fase 1 (SISAL) | 128 |
| ae89759c-c8f0-436f-bcc9-ef67874e2e30 | SILENE-FAUNE-PACA - Parc_National_des_Ecrins_2017_12_18 | 2 |
| 36d9b785-0739-4ce9-82e7-cf5001c20104 | Données naturalistes du CEN Auvergne concernant la Faune, la Flore et la Fonge - Données naturalistes faune du Conservatoire des espaces naturels Auvergne saisies avant le 18 février 2019. | 13 |
| 75a45d89-5ab2-470c-90df-b15417bf26dc | CardObs : Observations naturalistes issues de l'outil de saisie et gestion CardObs mis en place par le Service du Patrimoine Naturel (MNHN) / UMS PatriNat (OFB - CNRS - MNHN) - Données naturalistes de BOURRU Rémi | 6 |
| fe100feb-3973-4d88-bdea-f3a216c9fb7d | Community analysis and feeding ecology of the ichthyofauna in Gazi Bay sampled in August 1993 | 91 |
| b84a3711-b4ca-4e4f-adac-80dfaea98d1c | Hatikka.fi observations | 3 |
| 4c3b15bd-c98f-448c-9556-810fd82e8e30 | University of Georgia Collection of Arthropods | 14 |
| db47a906-257f-40a8-8638-4cb1bf092c2b | Kenneth S. Norris Center for Natural History, University of California Santa Cruz, Insect Collection | 1 |
| 0d8d90f4-973a-46a1-9ea1-55b0c7b222ea | San Diego Natural History Museum Entomology Department | 2 |
| ae24fb7b-af3c-478c-a1ed-2215a90793cc | Brigham Young University Arthropod Museum | 1 |
| c6772875-0588-47ef-a3fa-494881fefbce | Données de la réserve naturelle nationale de Saint-Mesmin - Données RNN St Mesmin 2019 | 23 |
| 1560f099-f48d-43ac-8d88-ce329a53aa7e | Saisie naturaliste opportuniste dans SICEN Occitanie - Données opportunistes du CEN Midi-Pyrénées | 13 |
| 9e02f8f5-ae2f-49b9-b896-ffb0260a284c | Museum of Southwestern Biology, Division of Arthropods | 5 |
| 63966993-9ff6-47d8-b59b-152320fb5be6 | Autres etudes et projets menés par les CEN d'Occitanie - Données ANA - CEN Ariège sur diverses études | 1 |
| 266628c1-56a0-46cb-b136-3b77dbc32268 | Megafaunal data from the 2009 BIOFUN trans-Mediterranean deep-sea cruise | 2 |
| f49dd893-1e91-4ce6-9aa7-e1047ffee557 | ZMMU MSU, White Sea Branch | 839 |
| 932e3edf-0bcc-46b7-a60f-3d7d0cc63479 | Epiphytic meiofauna and nematode community from seagrasses in Gazi and Nyali (Kenya) sampled in August 1989 | 35 |
| 2f0949ed-75b5-4f87-9f4a-d9334e626cc7 | Plymouth sound dataset. Soft sediment macrobenthos from the Plymouth Sound from 1995 | 171 |
| 7efa5937-15dd-4b6a-b18e-a830a094455c | Inventaire de la réserve naturelle géologique de Saucats - La Brède - Etude sur les protocoles I2M2 | 6 |
| 4cb11f8e-7132-43e9-a52c-dad325938326 | Données Faune Base SIRFF - FNE Centre-Val de Loire - Système d’Information Régional sur la Faune et la Flore - FNE Centre-Val de Loire - Données 2015 | 16 |
| a0adc8af-d15b-4fa4-ad9b-dc74dfbd6f59 | Meiobenthos of 5 mangrove vegetation types at Gazi Bay (Kenya) in August 1989 | 3 |
| e2980e63-d152-4219-8c1e-0ffdef3ea6aa | Auckland Museum NZ Marine Collection | 315 |
| d6cc311c-c5ab-4f23-9a20-10514f9eb9c4 | USGS Nonindigenous Aquatic Species database | 617 |
| b6054e23-dcdd-4ec6-aac7-94e958d7d0dc | RNF \| Habitats benthiques intertidaux, volet Taxons, des espaces protégés de la LPO France et de l'OFB - RNF Benthos - Relevés taxonomiques \| RNN Marais d'Yves | 315 |
| 36c180bb-01da-49c3-ba46-dfe65801fb20 | A summary of benthic studies in the sluice dock of Ostend during 1976-1981 | 1 |
| 110abe92-6a6a-47bc-9732-e8ab85e5e04a | Projet éolien Les Bruyères - Avifaune | 98 |
| a4305468-d94d-4244-b146-738e52d8a6e9 | Actualización del conocimiento de la diversidad de especies de invertebrados marinos bentónicos de aguas someras (<50m) del Sur del Golfo de México | 633 |
| 806bf7d4-f762-11e1-a439-00145eb45e9a | Diversidad bentónica de la laguna Celestún, Yucatán | 157 |
| 629befd5-fb45-4365-95c4-d07e72479b37 | Observations.be - Non-native species occurrences in Wallonia, Belgium | 8 |
| e8bfbb07-a739-49e9-952f-cd4e9a06603c | Fortalecimiento de las colecciones de ECOSUR. Primera fase (Zooplancton Chetumal) | 3299 |
| 0b81b94a-9761-46e5-a483-df6fcb840cc5 | Códigos de barras de la vida en peces y zooplancton de México | 2 |
| dd1c0ad1-ad9b-49c3-8783-aa5c9b66e75d | Catálogo de las especies de crustáceos anfípodos invasores del Parque Nacional Sistema Arrecifal Veracruzano (PNSAV) y la actualización de la base de datos (CONABIO) de los anfípodos en México | 161 |
| 7fba982c-f762-11e1-a439-00145eb45e9a | Crustáceos estomatópodos, anfípodos, isópodos y decápodos del litoral de Quintana Roo | 27 |
| d8cd16ba-bb74-4420-821e-083f2bac17c2 | INSDC Sequences | 12000 |
| e1ab583f-5629-4f9d-8335-72709b591273 | Two new species of the genus Crangonyx Bate, 1859 (Amphipoda: Crangonyctidae) from the St. Marks River Basin with notes on the “ Crangonyx floridanus complex ” | 2 |
| 8264af7b-43c7-429e-8d8f-d6e3bdf9d67c | A new species of Hyalella Smith, 1874 (Crustacea: Amphipoda: Hyalellidae) from Oklahoma, USA | 3 |
| 7af2903a-662f-47d7-92d8-e2830594aec0 | Cerrorchestia taboukeli sp. nov., a new terrestrial amphipod (Amphipoda, Talitridae) from Martinique Island | 5 |
| 1286a634-cdea-4d61-819c-42412405e464 | A new species of the genus Pseudocrangonyx (Crustacea: Amphipoda Pseudocrangonyctidae) from Simbok Cave, Korea | 2 |
| ba9984d8-d982-4fe6-b81c-a7585790034a | University of Texas, Biodiversity Center, Entomology Collection (UTIC) | 295 |
| 5f734908-8a5f-4858-b058-8e1c8c34051a | Macroinvertebrados asociados a la ciénaga de Guineo, Riosucio, Chocó | 33 |
| ce697b4c-7803-47d8-81bd-5a172f4960b5 | Invertebrate collections, UiB | 818 |
| b6162578-e753-40b4-bd69-0658221aee4c | NORFANZ Biological Survey, Tasman Sea, Australia - New Zealand 2003 | 9 |
| 854e35e6-f762-11e1-a439-00145eb45e9a | Invertebrate Zoology Division, Yale Peabody Museum | 3464 |
| 63c206b8-c610-40fc-b044-020934eef021 | New talitrids from South Africa (Amphipoda, Senticaudata, Talitroidea, Talitridae) with notes on their ecology | 20 |
| 6095b4d5-7e4d-4013-8eed-6ede683d6611 | Review of amphipods of the Melita group (Amphipoda: Melitidae) from the coastal waters of Sakhalin Island (Far East of Russia). III. Genera Abludomelita Karaman, 1981 and Melita Leach, 1814 | 10 |
| 21afe20c-de81-4221-8c1f-476c76d04c2e | On the genus Elasmopus Costa, 1853 from the Northeastern Coast of Brazil with five new species and new records | 17 |
| 8893caae-03b4-4074-9b25-ab834f5e4eb0 | Opisa takafuminakanoi, a new species of Opisidae from Hokkaido, Japan (Crustacea: Amphipoda) | 1 |
| 1bfc2e63-3da5-448a-8ebe-71f9d25c9c5a | Revision of the Oriental genus Horniella Raffray (Coleoptera, Staphylinidae, Pselaphinae) | 3 |
| 3ff76f4c-9b97-401a-ac7f-2181de01b68d | Re-description of Orchestia stephenseni Cecchini, 1928: designation of neotype and senior synonym to Orchestia constricta A. Costa, 1853 (Crustacea: Amphipoda: Talitridae) by Reversal of Precedence | 2 |
| 6f5c3fe7-b35d-4b04-89fd-b881ac6f82eb | Persianorchestia, a new talitrid genus (Crustacea: Amphipoda: Talitridae) from Gulf of Oman, Iran | 3 |
| 9a3a333c-0a76-42f0-a764-aacec714effd | A new caprellid species (Crustacea: Amphipoda: Senticaudata) from Brazil | 8 |
| f6f7eeb4-5b3d-4f99-90de-5f70f00766df | The family Hyalidae (Crustacea: Amphipoda: Talitroidea) from Korean waters. 2. Genus Protohyale Bousfield & Hendrycks, 2002 | 3 |
| 724e0dd4-6194-4e3a-bb22-e5259cb0a130 | A review of the families and genera of the superfamily PLATYSCELOIDEA Bowman & Gruner, 1973 (Crustacea: Amphipoda: Hyperiidea), together with keys to the families, genera and species | 4 |
| 798ad3a2-4340-4802-abb7-1e0116da6f64 | Pseudaeginella telukrimau sp. n., a new species of caprellid (Crustacea: Amphipoda) from Malaysia | 1 |
| 304402ac-32f8-4d5e-a4ae-09e95cb62631 | Spelaeogammarus uai (Bogidielloidea: Artesiidae): a new troglobitic amphipod from Brazil | 1 |
