# Supplementary material for: Exploring latitudinal gradients and environmental drivers of amphipod biodiversity patterns regarding depth and habitat variations
Source: Sci Rep. 2024 Dec 18;14:30547. doi: 10.1038/s41598-024-83314-6 (PMC11655948; doi:10.1038/s41598-024-83314-6)
Supplement: Supplementary file 2 — Supplementary Material 2 [file 41598_2024_83314_MOESM2_ESM.docx]

Supplementary 2

Table of contents

[Results of PCA analysis 1](#_Toc183686686)

[The abstraction of the selection GAM model and GLM models 2](#_Toc183686687)

[Results of distribution patterns for the whole database and total benthic and pelagic groups 5](#_Toc183686688)

[GAMs for the number of species, shallow water 10](#_Toc183686689)

[The results of GAM analysis for the number of species in shallow water. 10](#_Toc183686690)

[Model selection for the number of species based on GAM analysis for shallow water 27](#_Toc183686691)

[The Plots of GMAs analysis of the number of species in the shallow water 28](#_Toc183686692)

[GAMs for ES50, shallow water 32](#_Toc183686693)

[The script and extracted figures of GAM models for ES50 in shallow water. 32](#_Toc183686694)

[Model selection for ES50 shallow water 46](#_Toc183686695)

[Plots of GMAs analysis of ES50 in the shallow water 47](#_Toc183686696)

[GAMs for the number of species, deep sea 51](#_Toc183686697)

[The script and extracted figures of GAM models for the number of species in deep sea. 52](#_Toc183686698)

[Model selection for the number of species, deep water 68](#_Toc183686699)

[Plots of GMAs analysis of the number of species in the deep sea 69](#_Toc183686700)

[GAMs for ES50, deep sea 73](#_Toc183686701)

[Model selection for ES50 deep sea 88](#_Toc183686702)

[Plots of GMAs analysis of ES50 in the deep sea 89](#_Toc183686703)

[GLM for number of species, the whole database 93](#_Toc183686704)

[Model selection for number of species, whole database 99](#_Toc183686705)

[Plots of GLM analysis of the number of species 100](#_Toc183686706)

[GLM for ES50, whole database 105](#_Toc183686707)

[Model selection for ES50, the whole database 110](#_Toc183686708)

[Plots of GLM analysis of ES50 110](#_Toc183686709)

[Reference 116](#_Toc183686710)

# Results of PCA analysis

In this part, the results of PCA analysis were reported in table format

**Table 1 S2.** PCA analysis was used to define how environmental factors and biological variables interact with each other’s.

|  | **PC1** | **PC2** | **PC3** | **PC4** | **PC5** | **PC6** | **PC7** | **PC8** | **PC9** |
| --- | --- | --- | --- | --- | --- | --- | --- | --- | --- |
| Standard deviation | 1.81 | 1.52 | 1.22 | 0.96 | 0.93 | 0.73 | 0.61 | 0.41 | 0.21 |
| Proportion of Variance | 0.3265 | 0.23 | 0.15 | 0.09 | 0.09 | 0.05 | 0.04 | 0.02 | 0.00 |
| Cumulative Proportion | 0.3265 | 0.56 | 0.71 | 0.80 | 0.89 | 0.94 | 0.98 | 0.99 | 1.00 |

# The abstraction of the selection GAM model and GLM models

In this part, the final results of GLM and GAM model selections were reported in table formats to accelerate the comparison of results. The detailed results are reported in the following sections**.**

**Table 2 S2.** AIC analysis was used to compare models for goodness of fit while penalizing for overparameterization in the number of species of shallow benthic amphipod assemblage extracted from GAMs. The delta AIC represents how much the AIC score for each model differs from the top model, and can be used to estimate the relative support for each model. A delta AIC of 2 is potentially a significantly better fit.

| **Model** | **AIC** | **deltaAIC** | **rel.LL** | **weights** | **Cumulative.Weight** |
| --- | --- | --- | --- | --- | --- |
| gam.numsp.chlorophyll | 2630.7010 | 0.0000 | 1.0000 | 0.1111 | 0.1111 |
| gam.numsp.env | 2630.7775 | 0.0765 | 0.9625 | 0.1069 | 0.2180 |
| gam.numsp.latlon | 2630.9557 | 0.2547 | 0.8804 | 0.0978 | 0.3158 |
| gam.numsp.salinity | 2630.9558 | 0.2548 | 0.8804 | 0.0978 | 0.4137 |
| gam.numsp.temp | 2630.9560 | 0.2549 | 0.8803 | 0.0978 | 0.5115 |
| gam.numsp.PO4 | 2630.9565 | 0.2555 | 0.8801 | 0.0978 | 0.6092 |
| gam.numsp.depth | 2630.9566 | 0.2556 | 0.8800 | 0.0978 | 0.7070 |
| gam.numsp.pH | 2630.9584 | 0.2574 | 0.8792 | 0.0977 | 0.8047 |
| gam.numsp.current | 2630.9588 | 0.2578 | 0.8791 | 0.0977 | 0.9023 |
| gam.numsp.dissolved.oxygen | 2630.9590 | 0.2580 | 0.8790 | 0.0977 | 1.0000 |
| gam.numsp.numrec | 2703.6379 | 72.9369 | 0.0000 | 0.0000 | 1.0000 |
| gam.numsp.intercept | 2973.6480 | 342.9470 | 0.0000 | 0.0000 | 1.0000 |

**Table 3 S2**. AIC analysis was used to compare models extracted from GAMs for goodness of fit while penalizing for overparameterization ES50 of shallow benthic species

| **Model** | **AIC** | **deltaAIC** | **rel.LL** | **weights** | **Cumulative.Weight** |
| --- | --- | --- | --- | --- | --- |
| gam.ES50.temp | 1817.728 | 0 | 1 | 0.1242 | 0.1242 |
| gam.ES50.env | 1817.733 | 0.0045 | 0.9978 | 0.1239 | 0.2482 |
| gam.ES50.dissolved.oxygen | 1817.971 | 0.2425 | 0.8858 | 0.11 | 0.3582 |
| gam.ES50.latlon | 1818.323 | 0.5948 | 0.7428 | 0.0923 | 0.4505 |
| gam.ES50.PO4 | 1818.323 | 0.595 | 0.7427 | 0.0923 | 0.5427 |
| gam.ES50.current | 1818.325 | 0.5961 | 0.7423 | 0.0922 | 0.6349 |
| gam.ES50.salinity | 1818.325 | 0.5961 | 0.7423 | 0.0922 | 0.7271 |
| gam.ES50.chlorophyll | 1818.325 | 0.5962 | 0.7422 | 0.0922 | 0.8193 |
| gam.ES50.depth | 1818.332 | 0.6036 | 0.7395 | 0.0919 | 0.9112 |
| gam.ES50.pH | 1818.399 | 0.6705 | 0.7152 | 0.0888 | 1 |
| gam.ES50.intercept | 1891.414 | 73.6856 | 0 | 0 | 1 |

**Table 4 S2.** AIC analysis was used to compare models extracted from GAMs for goodness of fit while penalizing for overparameterization of the number of species of deep benthic amphipod.

| **Model** | **AIC** | **deltaAIC** | **rel.LL** | **weights** | **Cumulative.Weight** |
| --- | --- | --- | --- | --- | --- |
| gam.numsp.env | 2103.437 | 0 | 1 | 0.8646 | 0.8646 |
| gam.numsp.depth | 2109.501 | 6.0638 | 0.0482 | 0.0417 | 0.9062 |
| gam.numsp.temp | 2109.667 | 6.2298 | 0.0444 | 0.0384 | 0.9446 |
| gam.numsp.pH | 2110.416 | 6.979 | 0.0305 | 0.0264 | 0.971 |
| gam.numsp.dissolved.oxygen | 2112.535 | 9.0978 | 0.0106 | 0.0091 | 0.9801 |
| gam.numsp.current | 2112.79 | 9.3523 | 0.0093 | 0.0081 | 0.9882 |
| gam.numsp.salinity | 2113.84 | 10.4026 | 0.0055 | 0.0048 | 0.993 |
| gam.numsp.PO4 | 2114.445 | 11.0072 | 0.0041 | 0.0035 | 0.9965 |
| gam.numsp.chlorophyll | 2114.445 | 11.0072 | 0.0041 | 0.0035 | 1 |
| gam.numsp.numrec | 2136.03 | 32.5927 | 0 | 0 | 1 |
| gam.numsp.latlon | 2227.333 | 123.896 | 0 | 0 | 1 |
| gam.numsp.intercept | 2799.897 | 696.4597 | 0 | 0 | 1 |

**Table 5 S2.** AIC analysis was used to compare models extracted from GAMs for goodness of fit while penalizing for overparameterization ES50 of deep benthic species

| **Model** | **AIC** | **deltaAIC** | **rel.LL** | **weights** | **Cumulative.Weight** |
| --- | --- | --- | --- | --- | --- |
| gam.ES50.env | 1754.045 | 0 | 1 | 0.997 | 0.997 |
| gam.ES50.depth | 1767.187 | 13.1421 | 0.0014 | 0.0014 | 0.9984 |
| gam.ES50.pH | 1768.925 | 14.8802 | 0.0006 | 0.0006 | 0.999 |
| gam.ES50.temp | 1769.677 | 15.6317 | 0.0004 | 0.0004 | 0.9994 |
| gam.ES50.salinity | 1770.024 | 15.979 | 0.0003 | 0.0003 | 0.9997 |
| gam.ES50.dissolved.oxygen | 1772.131 | 18.0865 | 0.0001 | 0.0001 | 0.9998 |
| gam.ES50.current | 1772.276 | 18.231 | 0.0001 | 0.0001 | 0.9999 |
| gam.ES50.latlon | 1775.649 | 21.6037 | 0 | 0 | 1 |
| gam.ES50.PO4 | 1775.65 | 21.6047 | 0 | 0 | 1 |
| gam.ES50.chlorophyll | 1775.65 | 21.6054 | 0 | 0 | 1 |
| gam.ES50.intercept | 1895.476 | 141.4313 | 0 | 0 | 1 |

**Table 6 S2**. AIC analysis was used to compare models extracted from GLM for goodness of fit while penalizing for overparameterization number of species of benthic species.

| **Model** | **AIC** | **deltaAIC** | **rel.LL** | **weights** | **Cumulative.Weight** |
| --- | --- | --- | --- | --- | --- |
| GLM.numsp.lat | 5984.8859 | 0.0000 | 1.0000 | 1.0000 | 1.0000 |
| GLM.numsp.chl | 6020.7991 | 35.9132 | 0.0000 | 0.0000 | 1.0000 |
| GLM.numsp.temp | 6095.4784 | 110.5925 | 0.0000 | 0.0000 | 1.0000 |
| GLM.numsp.PO4 | 6100.3532 | 115.4673 | 0.0000 | 0.0000 | 1.0000 |
| GLM.numsp.salinity | 6413.6485 | 428.7626 | 0.0000 | 0.0000 | 1.0000 |
| GLM.numsp.pp | 6417.3041 | 432.4181 | 0.0000 | 0.0000 | 1.0000 |
| GLM.numsp.DO | 6442.5315 | 457.6456 | 0.0000 | 0.0000 | 1.0000 |
| GLM.numsp.pH | 6476.0047 | 491.1188 | 0.0000 | 0.0000 | 1.0000 |
| GLM.numsp.Area | 6480.2646 | 495.3787 | 0.0000 | 0.0000 | 1.0000 |
| GLM.numsp.depth | 6495.7344 | 510.8485 | 0.0000 | 0.0000 | 1.0000 |
| GLM.numsp.Curvvel | 6501.3640 | 516.4781 | 0.0000 | 0.0000 | 1.0000 |
| GLM.numsp.numrec | 6503.8480 | 518.9621 | 0.0000 | 0.0000 | 1.0000 |
| GLM.numsp.intercept | Inf | Inf | 0.0000 | 0.0000 | 1.0000 |

**Table 7 S2**. AIC analysis was used to compare models extracted from GLM for goodness of fit while penalizing for overparameterization ES50 of benthic species.

| **Model** | **AIC** | **deltaAIC** | **rel.LL** | **weights** | **Cumulative.Weight** |
| --- | --- | --- | --- | --- | --- |
| GLM.ES50.lat | 231.4169 | 0.0000 | 1.0000 | 0.5114 | 0.5114 |
| GLM.ES50.salinity | 234.7531 | 3.3362 | 0.1886 | 0.0965 | 0.6079 |
| GLM.ES50.chl | 235.6104 | 4.1935 | 0.1229 | 0.0628 | 0.6707 |
| GLM.ES50.pp | 235.6924 | 4.2755 | 0.1179 | 0.0603 | 0.7310 |
| GLM.ES50.temp | 235.7059 | 4.2890 | 0.1171 | 0.0599 | 0.7909 |
| GLM.ES50.depth | 235.7800 | 4.3632 | 0.1129 | 0.0577 | 0.8487 |
| GLM.ES50.intercept | 236.4553 | 5.0384 | 0.0805 | 0.0412 | 0.8899 |
| GLM.ES50.PO4 | 236.6674 | 5.2505 | 0.0724 | 0.0370 | 0.9269 |
| GLM.ES50.Area | 237.3266 | 5.9098 | 0.0521 | 0.0266 | 0.9535 |
| GLM.ES50.Curvvel | 238.2635 | 6.8466 | 0.0326 | 0.0167 | 0.9702 |
| GLM.ES50.pH | 238.2833 | 6.8665 | 0.0323 | 0.0165 | 0.9867 |
| GLM.ES50.DO | 238.7179 | 7.3010 | 0.0260 | 0.0133 | 1.0000 |

**Table 8 S2**. The results of the normality test and dip-test for the distribution of gamma (the number of species) and ES50 in each 5° latitudinal band. The p-value less than 0.05 for normality means data has a non-normal distribution and the p-value less than 0.05 for dip test means there is no bimodal or multimodel pattern.

|  | **Anderson-Darling normality test** | | **Hartigans' dip test** | |
| --- | --- | --- | --- | --- |
|  | A | p-value | D | p-value |
| Gamma benthic shallow | 1.2031 | 0.003308 | 0.059217 | 0.4636 |
| Gamma benthic deep | 1.2542 | 0.00246 | 0.053778 | 0.6413 |
| Gamma pelagic deep | 0.56462 | 0.133 | 0.055556 | 0.582 |
| Gamma pelagic shallow | 1.1687 | 0.004039 | 0.033422 | 0.9932 |
| ES50 pelagic shallow | 0.86984 | 0.0229 | 0.069589 | 0.2049 |
| ES50 pelagic deep | 1.0211 | 0.009509 | 0.056963 | 0.5353 |
| ES50 benthic shallow | 1.44 | 0.0008385 | 0.045401 | 0.8843 |
| ES50 benthic deep | 1.413 | 0.0009801 | 0.046583 | 0.8549 |

# Results of distribution patterns for the whole database and total benthic and pelagic groups

In this part, the geographical position of the personal database, total database amphipod records, the number of records and species of benthic and pelagic databases were demonstrated. Additionally, the kernel plots for defining the bimodal patterns were reported. QGIS 3.28.10 was used to create the figures


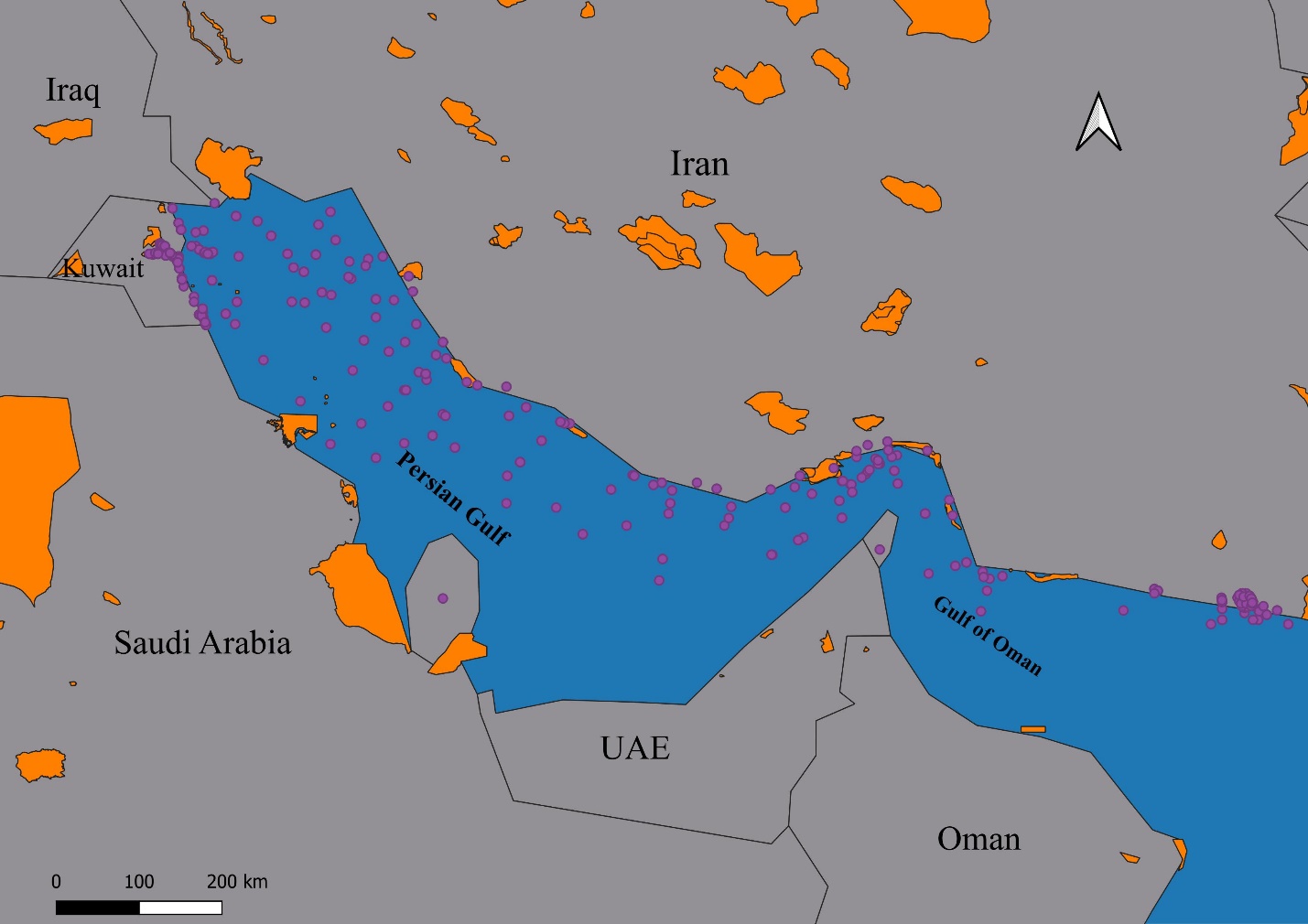


**Figure 1 S2.** The geographical position of amphipod records of personal data in the Persian Gulf and the Gulf of Oman. This region was not protected mostly. The database consisted of 1504 records of 117 species and 53 species were endemic to the region. The protected area’s map (orange color) was extracted from UNEP-WCMC and IUCN^1^, available at: [www.protectedplanet.net](http://protectedplanet.net/)). QGIS 3.28.10 was used to create the figure.


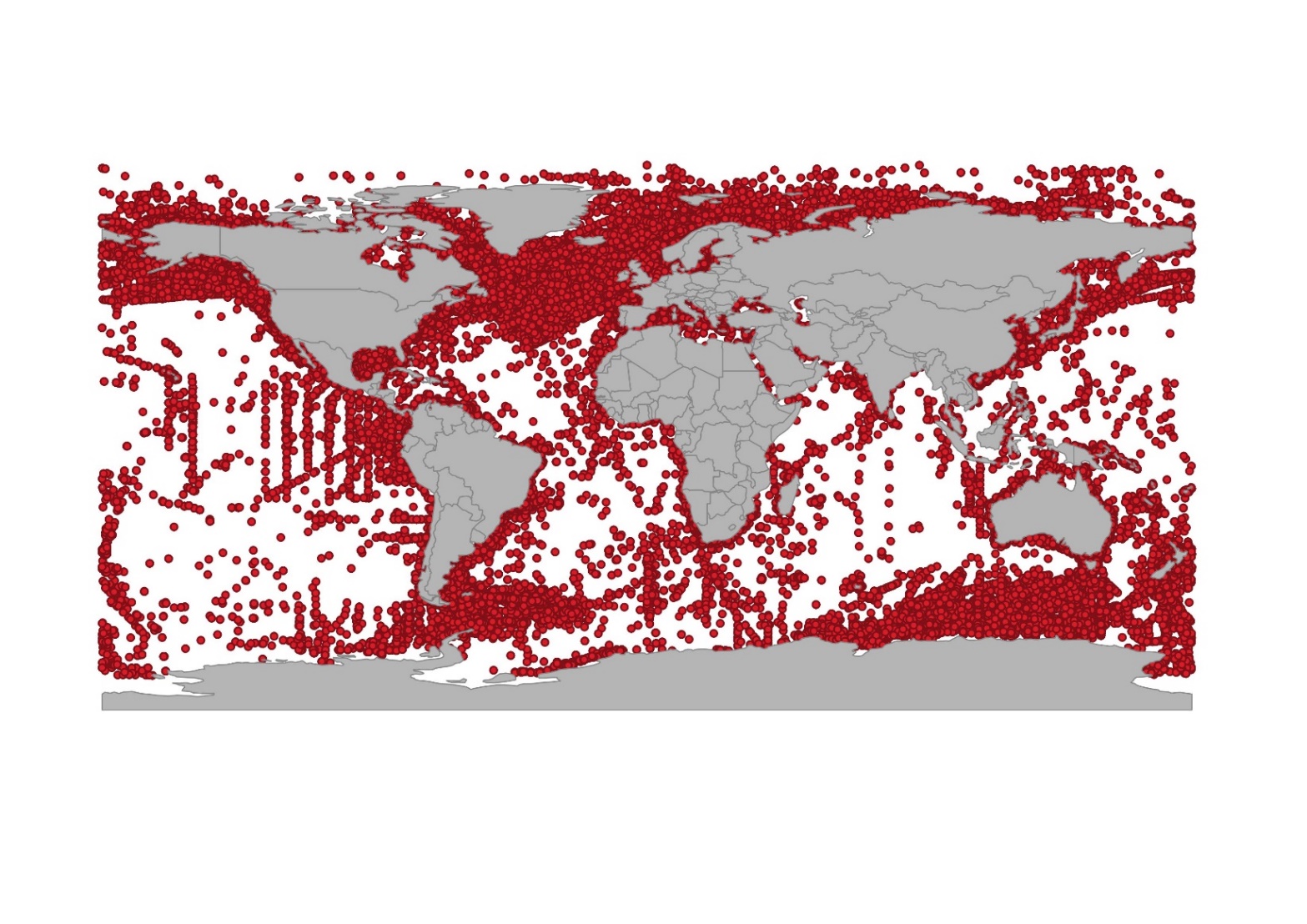


**Figure 2 S2.** The geographical position of amphipod records (after cleaning data) was extracted from all databases containing 1,142,416 records.


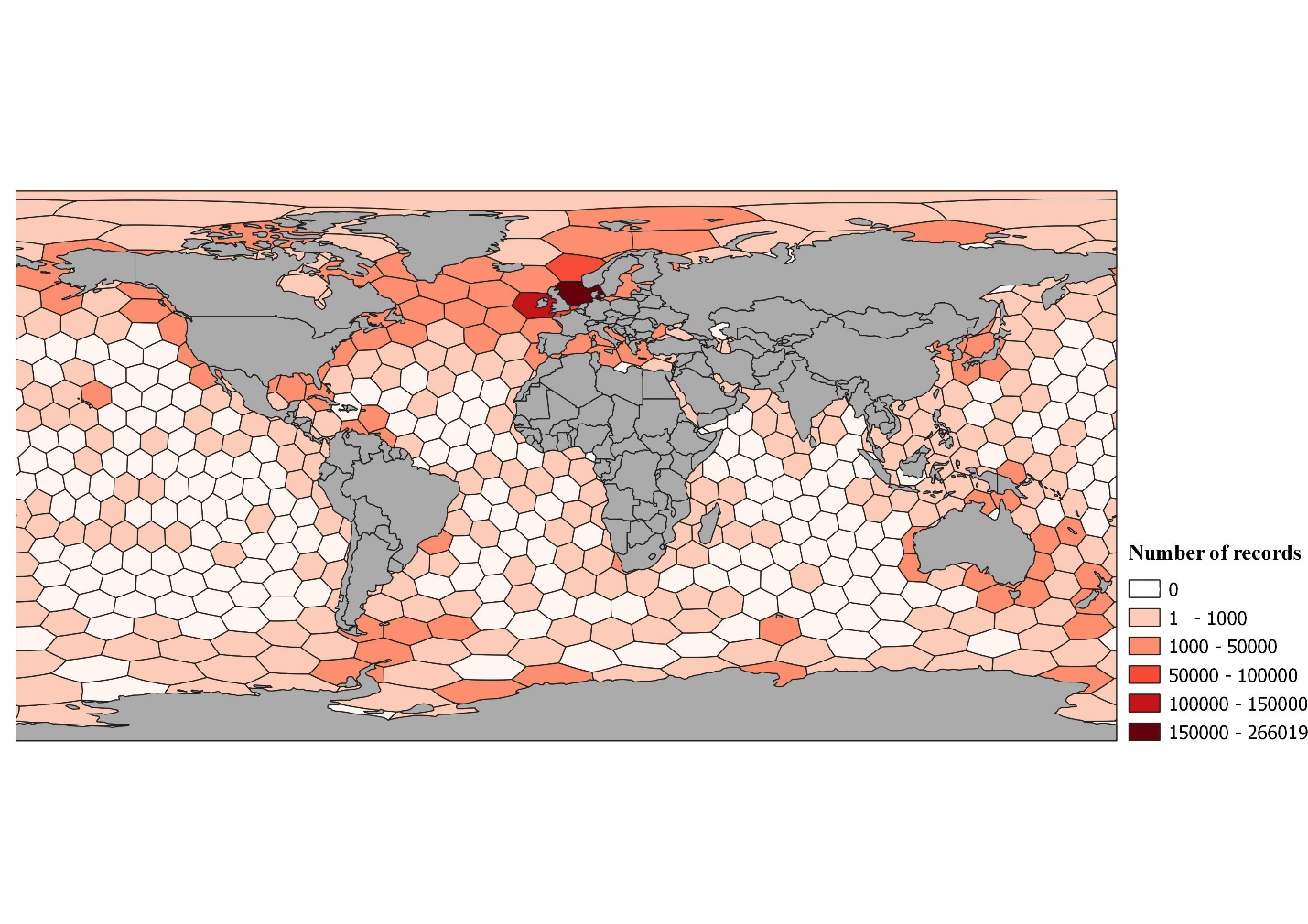


**Figure 3 S2.** The number of occurrence records per c. 50 000 km2 hexagonal cells for benthic amphipods. QGIS 3.28.10 was used to create the figure.


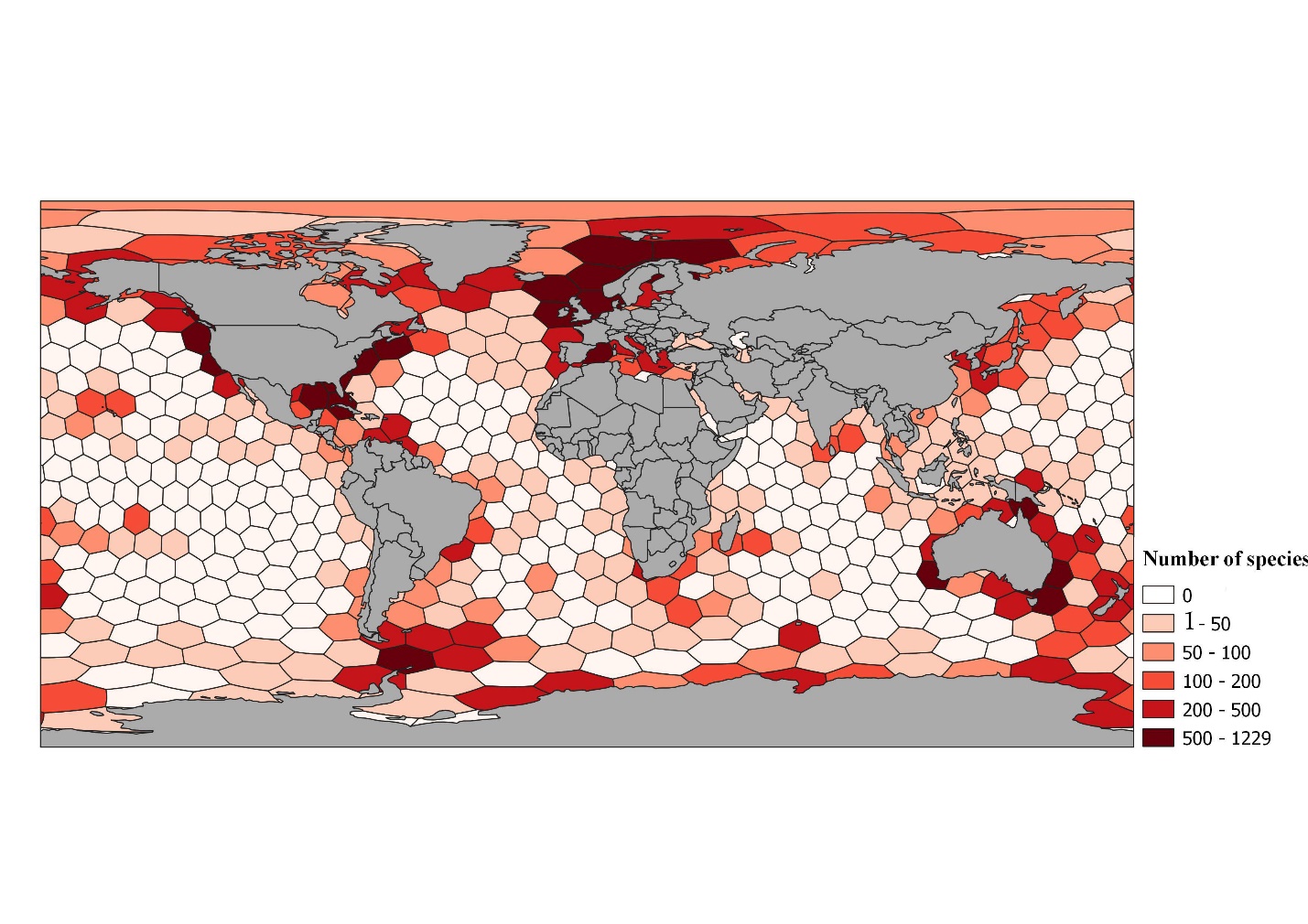


**Figure 4 S2.** The number of species per c. 50 000 km2 (alpha diversity) hexagonal cells for benthic amphipods. QGIS 3.28.10 was used to create the figure.


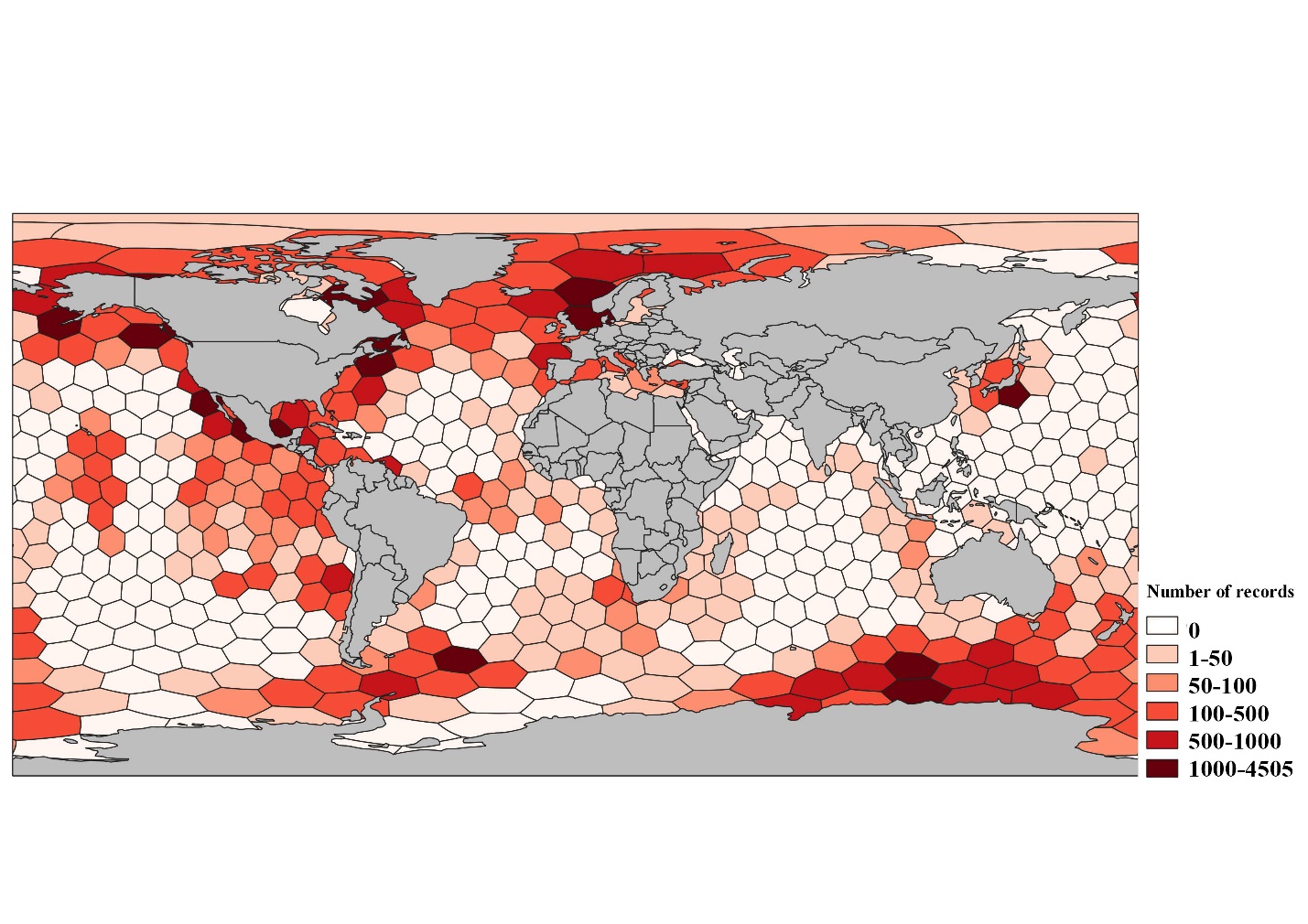


**Figure 5 S2**. The number of occurrence records per c. 50 000 km2 hexagonal cells for pelagic amphipods. QGIS 3.28.10 was used to create the figure.


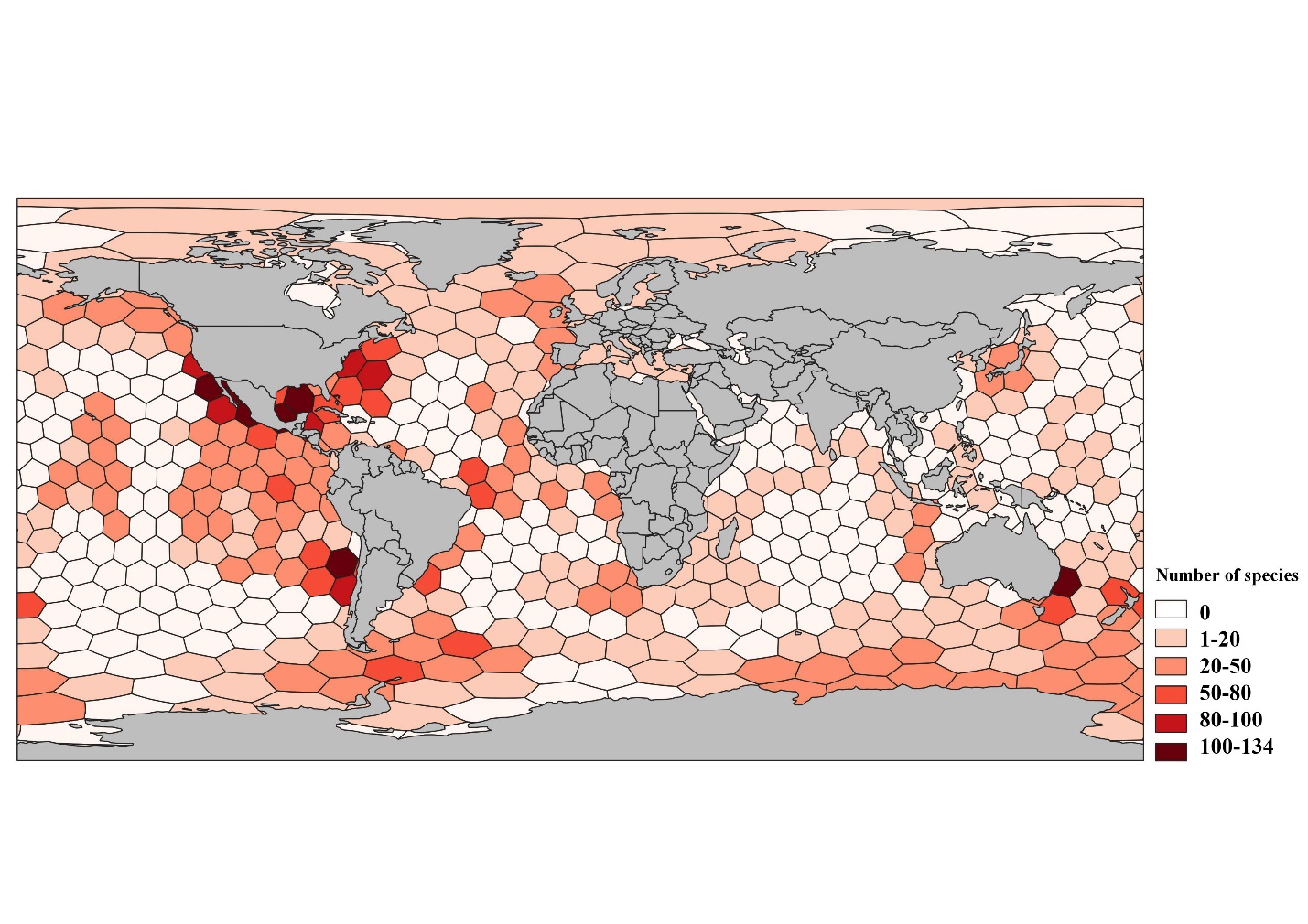


**Figure 6 S2.** The number of species per c. 50 000 km2 (alpha diversity) hexagonal cells for pelagic amphipods. QGIS 3.28.10 was used to create figure.


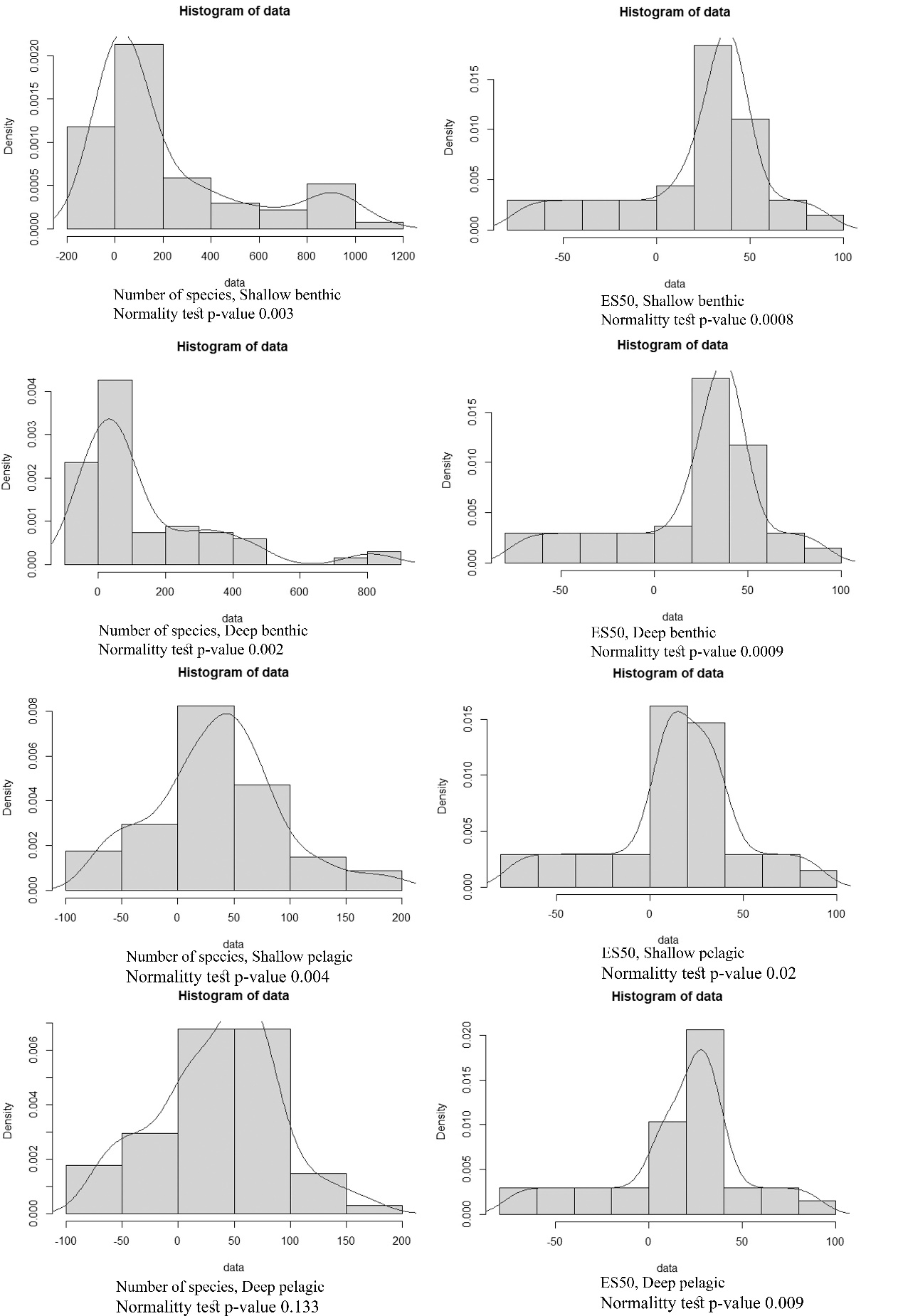


**Figure 7 S2**. The Kernel curves for species richness and ES50 per 5 ° latitude band. The bimodality pattern can be accepted only for the shallow benthic species richness.

# GAMs for the number of species, shallow water

GAMs analysis was used for the whole benthic database and two subgroups; shallow and deep-sea groups. In these analyses, “intercept” represents the fit of a model that assumes no relationship between the species counts and the environment and no spatial autocorrelation. The “latlon” model represents the fit of a model that fits spatial autocorrelation without environmental effects. The “env” model represents the combined effects of all environmental predictors, and the remainder of the models estimate the effects of a single predictor at a time. The total number of records for each locality as an estimate of sampling effort was used in models. The spatial autocorrelation model used a two-dimensional spherical spline on the latitude and longitude of sampling sites. **
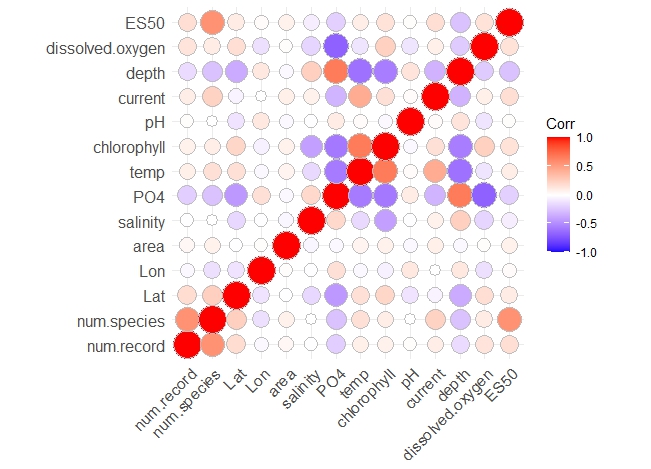
**

**Figure 7 S2.** The correlation between environmental variables and ES50, the number of records, and the number of shallow benthic species.

## The results of GAM analysis for the number of species in shallow water.

1. GAM model for intercept

*gam.numsp.intercept <- gam(num.species ~ 1, data = data, family = "nb", method = "REML", select = TRUE)*

*> gam.check(gam.numsp.intercept)*

Method: REML Optimizer: outer newton

full convergence after 4 iterations.

Gradient range [4.320419e-06,4.320419e-06]

(score 1487.293 & scale 1).

Hessian positive definite, eigenvalue range [235.4627,235.4627].

Model rank = 1 / 1

*> summary(gam.numsp.intercept)*

Family: Negative Binomial(0.367)

Link function: log

Formula:

num.species ~ 1

Parametric coefficients:

Estimate Std. Error z value Pr(>|z|)

(Intercept) 4.02661 0.09181 43.86 <2e-16 ***

Signif. codes: 0 ‘***’ 0.001 ‘**’ 0.01 ‘*’ 0.05 ‘.’ 0.1 ‘ ’ 1

R-sq.(adj) = 2.22e-16 Deviance explained = 1.49e-09%

-REML = 1487.3 Scale est. = 1 n = 325


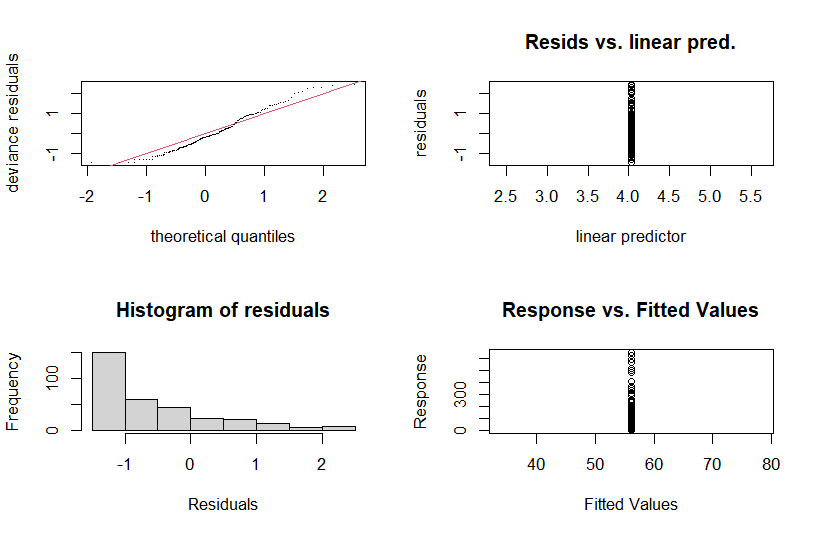


**Figure 8 S2.** The extracted plots of GAM model for intercept.

1. GAM model for the number of records

*gam.numsp.numrec <- gam(num.species ~ s(num.record), data = data, family = "nb", method = "REML", select = TRUE)*

*> gam.check(gam.numsp.numrec)*

Method: REML Optimizer: outer newton

full convergence after 10 iterations.

Gradient range [-4.535265e-07,3.336951e-06]

(score 1373.584 & scale 1).

Hessian positive definite, eigenvalue range [0.4783034,181.5172].

Model rank = 10 / 10

Basis dimension (k) checking results. Low p-value (k-index<1) may

indicate that k is too low, especially if edf is close to k'.

k' edf k-index p-value

s(num.record) 9.00 8.59 0.25 <2e-16 ***

Signif. codes: 0 ‘***’ 0.001 ‘**’ 0.01 ‘*’ 0.05 ‘.’ 0.1 ‘ ’ 1

*> summary(gam.numsp.numrec)*

Family: Negative Binomial(0.7)

Link function: log

Formula:

num.species ~ s(num.record)

Parametric coefficients:

Estimate Std. Error z value Pr(>|z|)

(Intercept) 3.13258 0.06781 46.2 <2e-16 ***

Signif. codes: 0 ‘***’ 0.001 ‘**’ 0.01 ‘*’ 0.05 ‘.’ 0.1 ‘ ’ 1

Approximate significance of smooth terms:

edf Ref.df Chi.sq p-value

s(num.record) 8.587 9 389.3 <2e-16 ***

Signif. codes: 0 ‘***’ 0.001 ‘**’ 0.01 ‘*’ 0.05 ‘.’ 0.1 ‘ ’ 1

R-sq.(adj) = -2.38 Deviance explained = 52.9%

-REML = 1373.6 Scale est. = 1 n = 325


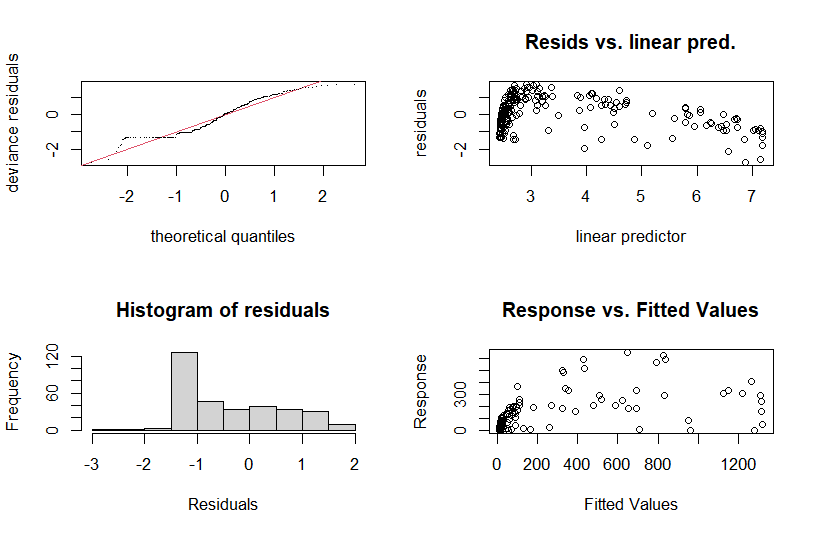


**Figure 9 S2.** The extracted plots of the GAM model for the number of records.

1. GAM model for spatial autocorrelation.

*gam.numsp.latlon <- gam(num.species ~ s(Lat,Lon, bs="sos")+ s(num.record), data = data, family = "nb", method = "REML", select = TRUE)*

*> gam.check(gam.numsp.latlon)*

Method: REML Optimizer: outer newton

full convergence after 11 iterations.

Gradient range [-3.676199e-06,2.285538e-05]

(score 1354.46 & scale 1).

Hessian positive definite, eigenvalue range [0.4785864,144.1709].

Model rank = 59 / 59

Basis dimension (k) checking results. Low p-value (k-index<1) may

indicate that k is too low, especially if edf is close to k'.

k' edf k-index p-value

s(Lat,Lon) 49.00 28.35 0.93 0.75

s(num.record) 9.00 8.55 0.32 <2e-16 ***

Signif. codes: 0 ‘***’ 0.001 ‘**’ 0.01 ‘*’ 0.05 ‘.’ 0.1 ‘ ’ 1

*> summary(gam.numsp.latlon)*

Family: Negative Binomial(0.915)

Link function: log

Formula:

num.species ~ s(Lat, Lon, bs = "sos") + s(num.record)

Parametric coefficients:

Estimate Std. Error z value Pr(>|z|)

(Intercept) 2.88566 0.06069 47.55 <2e-16 ***

Signif. codes: 0 ‘***’ 0.001 ‘**’ 0.01 ‘*’ 0.05 ‘.’ 0.1 ‘ ’ 1

Approximate significance of smooth terms:

edf Ref.df Chi.sq p-value

s(Lat,Lon) 28.35 49 136.8 <2e-16 ***

s(num.record) 8.55 9 234.8 <2e-16 ***

Signif. codes: 0 ‘***’ 0.001 ‘**’ 0.01 ‘*’ 0.05 ‘.’ 0.1 ‘ ’ 1

R-sq.(adj) = -1.17 Deviance explained = 67.8%

-REML = 1354.5 Scale est. = 1 n = 325


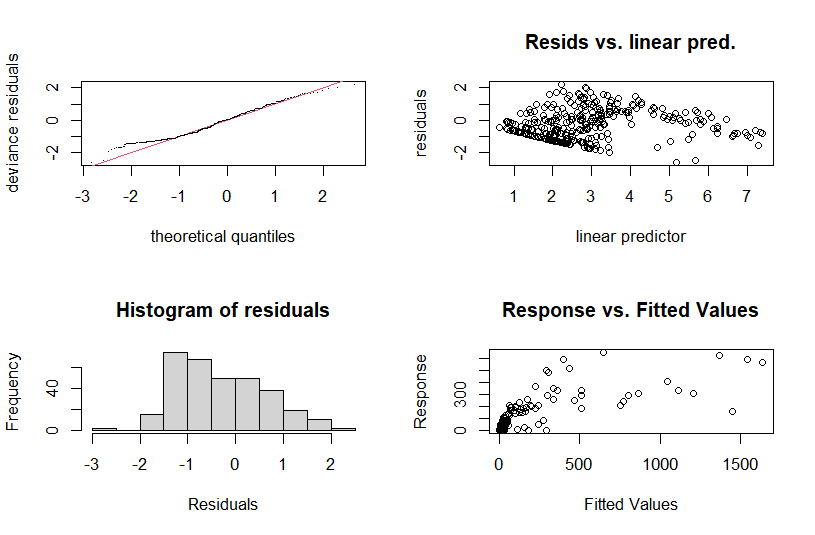


**Figure 10 S2.** The extracted plots of the GAM model for the spatial autocorrelation characters.

1. GAM model for depth

*gam.numsp.depth <- gam(num.species ~ s(Lat,Lon, bs="sos")+ s(num.record)+s(depth), data = data, family = "nb", method = "REML", select = TRUE)*

*> gam.check(gam.numsp.depth)*

Method: REML Optimizer: outer newton

full convergence after 12 iterations.

Gradient range [-0.0001746564,0.0001759364]

(score 1354.46 & scale 1).

Hessian positive definite, eigenvalue range [4.16984e-05,144.1706].

Model rank = 68 / 68

Basis dimension (k) checking results. Low p-value (k-index<1) may

indicate that k is too low, especially if edf is close to k'.

k' edf k-index p-value

s(Lat,Lon) 4.90e+01 2.84e+01 0.93 0.82

s(num.record) 9.00e+00 8.55e+00 0.32 <2e-16 ***

s(depth) 9.00e+00 5.71e-04 0.83 0.11

Signif. codes: 0 ‘***’ 0.001 ‘**’ 0.01 ‘*’ 0.05 ‘.’ 0.1 ‘ ’ 1

*> summary(gam.numsp.depth)*

Family: Negative Binomial(0.915)

Link function: log

Formula:

num.species ~ s(Lat, Lon, bs = "sos") + s(num.record) + s(depth)

Parametric coefficients:

Estimate Std. Error z value Pr(>|z|)

(Intercept) 2.88566 0.06069 47.55 <2e-16 ***

---

Signif. codes: 0 ‘***’ 0.001 ‘**’ 0.01 ‘*’ 0.05 ‘.’ 0.1 ‘ ’ 1

Approximate significance of smooth terms:

edf Ref.df Chi.sq p-value

s(Lat,Lon) 2.835e+01 49 136.8 <2e-16 ***

s(num.record) 8.550e+00 9 234.8 <2e-16 ***

s(depth) 5.706e-04 9 0.0 0.881

---

Signif. codes: 0 ‘***’ 0.001 ‘**’ 0.01 ‘*’ 0.05 ‘.’ 0.1 ‘ ’ 1

R-sq.(adj) = -1.17 Deviance explained = 67.8%

-REML = 1354.5 Scale est. = 1 n = 325


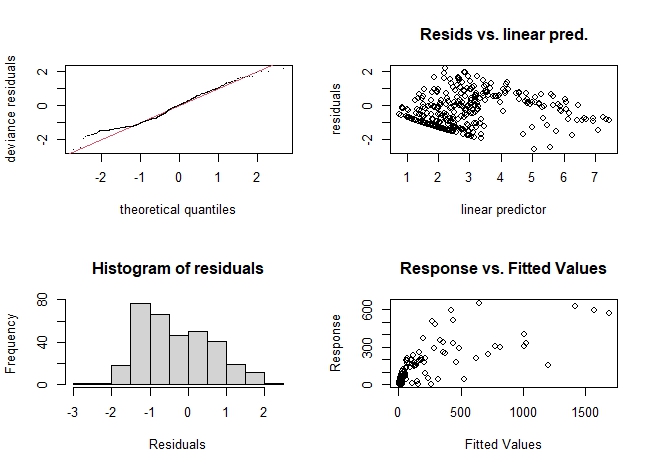


**Figure 11S**. The extracted plots of GAM model for depth.

1. GAM model for chlorophyll

*gam.numsp.chlorophyll <- gam(num.species ~ s(Lat,Lon, bs="sos")+ s(num.record)+s(chlorophyll), data = data, family = "nb", method = "REML", select = TRUE)*

*> gam.check(gam.numsp.chlorophyll)*

Method: REML Optimizer: outer newton

full convergence after 12 iterations.

Gradient range [-0.004313754,0.002968765]

(score 1354.458 & scale 1).

Hessian positive definite, eigenvalue range [1.982887e-05,143.8341].

Model rank = 68 / 68

Basis dimension (k) checking results. Low p-value (k-index<1) may

indicate that k is too low, especially if edf is close to k'.

k' edf k-index p-value

s(Lat,Lon) 49.000 28.458 0.93 0.82

s(num.record) 9.000 8.551 0.32 <2e-16 ***

s(chlorophyll) 9.000 0.118 0.99 0.96

Signif. codes: 0 ‘***’ 0.001 ‘**’ 0.01 ‘*’ 0.05 ‘.’ 0.1 ‘ ’ 1

summary(gam.numsp.chlorophyll)

Family: Negative Binomial(0.916)

Link function: log

Formula:

num.species ~ s(Lat, Lon, bs = "sos") + s(num.record) + s(chlorophyll)

Parametric coefficients:

Estimate Std. Error z value Pr(>|z|)

(Intercept) 2.88450 0.06066 47.55 <2e-16 ***

Signif. codes: 0 ‘***’ 0.001 ‘**’ 0.01 ‘*’ 0.05 ‘.’ 0.1 ‘ ’ 1

Approximate significance of smooth terms:

edf Ref.df Chi.sq p-value

s(Lat,Lon) 28.4581 49 137.197 <2e-16 ***

s(num.record) 8.5510 9 234.751 <2e-16 ***

s(chlorophyll) 0.1179 9 0.109 0.319

Signif. codes: 0 ‘***’ 0.001 ‘**’ 0.01 ‘*’ 0.05 ‘.’ 0.1 ‘ ’ 1

R-sq.(adj) = -1.16 Deviance explained = 67.9%

-REML = 1354.5 Scale est. = 1 n = 325


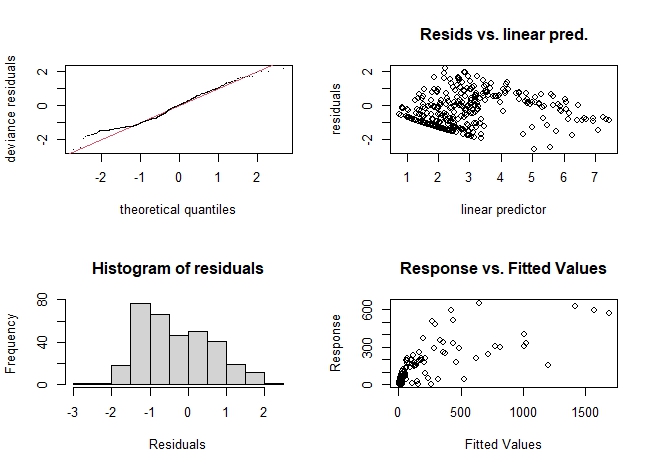


**Figure 12S**. The extracted plots of the GAM model for chlorophyll.

1. GAM model for current

*gam.numsp.current <- gam(num.species ~ s(Lat,Lon, bs="sos")+ s(num.record)+s(current), data = data, family = "nb", method = "REML", select = TRUE)*

*> gam.check(gam.numsp.current)*

Method: REML Optimizer: outer newton

full convergence after 10 iterations.

Gradient range [-0.0002673362,0.0005642008]

(score 1354.46 & scale 1).

Hessian positive definite, eigenvalue range [0.000127229,144.1709].

Model rank = 68 / 68

Basis dimension (k) checking results. Low p-value (k-index<1) may

indicate that k is too low, especially if edf is close to k'.

k' edf k-index p-value

s(Lat,Lon) 49.00000 28.35036 0.93 0.78

s(num.record) 9.00000 8.55001 0.32 <2e-16 ***

s(current) 9.00000 0.00177 0.93 0.69

Signif. codes: 0 ‘***’ 0.001 ‘**’ 0.01 ‘*’ 0.05 ‘.’ 0.1 ‘ ’ 1

*summary(gam.numsp.current)*

Family: Negative Binomial(0.915)

Link function: log

Formula:

num.species ~ s(Lat, Lon, bs = "sos") + s(num.record) + s(current)

Parametric coefficients:

Estimate Std. Error z value Pr(>|z|)

(Intercept) 2.88566 0.06069 47.55 <2e-16 ***

Signif. codes: 0 ‘***’ 0.001 ‘**’ 0.01 ‘*’ 0.05 ‘.’ 0.1 ‘ ’ 1

Approximate significance of smooth terms:

edf Ref.df Chi.sq p-value

s(Lat,Lon) 28.350363 49 136.744 <2e-16 ***

s(num.record) 8.550011 9 234.772 <2e-16 ***

s(current) 0.001768 9 0.001 0.554

Signif. codes: 0 ‘***’ 0.001 ‘**’ 0.01 ‘*’ 0.05 ‘.’ 0.1 ‘ ’ 1

R-sq.(adj) = -1.17 Deviance explained = 67.8%

-REML = 1354.5 Scale est. = 1 n = 325


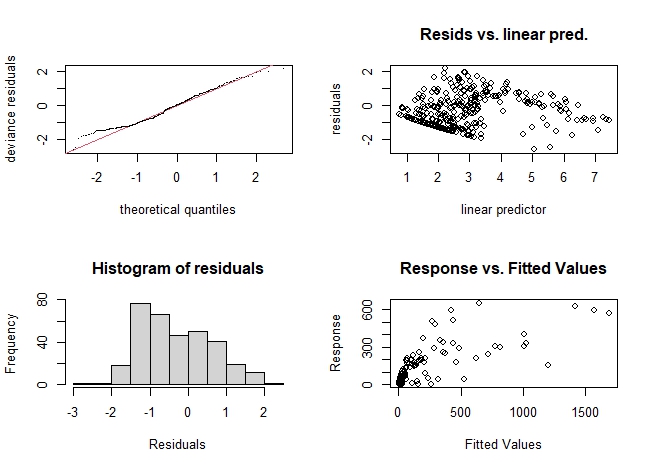


**Figure 13S**. The extracted plots of the GAM model for current.

1. GAM model for pH

*gam.numsp.pH <- gam(num.species ~ s(Lat,Lon, bs="sos")+ s(num.record)+s(pH), data = data, family = "nb", method = "REML", select = TRUE)*

*> gam.check(gam.numsp.pH)*

Method: REML Optimizer: outer newton

full convergence after 12 iterations.

Gradient range [-0.0003657867,0.0001261746]

(score 1354.46 & scale 1).

Hessian positive definite, eigenvalue range [4.489226e-05,144.1706].

Model rank = 68 / 68

Basis dimension (k) checking results. Low p-value (k-index<1) may

indicate that k is too low, especially if edf is close to k'.

k' edf k-index p-value

s(Lat,Lon) 49.00000 28.35053 0.93 0.76

s(num.record) 9.00000 8.55003 0.32 <2e-16 ***

s(pH) 9.00000 0.00229 0.93 0.81

---

Signif. codes: 0 ‘***’ 0.001 ‘**’ 0.01 ‘*’ 0.05 ‘.’ 0.1 ‘ ’ 1

*summary(gam.numsp.pH)*

Family: Negative Binomial(0.915)

Link function: log

Formula:

num.species ~ s(Lat, Lon, bs = "sos") + s(num.record) + s(pH)

Parametric coefficients:

Estimate Std. Error z value Pr(>|z|)

(Intercept) 2.88566 0.06069 47.55 <2e-16 ***

Signif. codes: 0 ‘***’ 0.001 ‘**’ 0.01 ‘*’ 0.05 ‘.’ 0.1 ‘ ’ 1

Approximate significance of smooth terms:

edf Ref.df Chi.sq p-value

s(Lat,Lon) 28.350531 49 136.741 <2e-16 ***

s(num.record) 8.550028 9 234.773 <2e-16 ***

s(pH) 0.002289 9 0.001 0.569

Signif. codes: 0 ‘***’ 0.001 ‘**’ 0.01 ‘*’ 0.05 ‘.’ 0.1 ‘ ’ 1

R-sq.(adj) = -1.17 Deviance explained = 67.8%

-REML = 1354.5 Scale est. = 1 n = 325


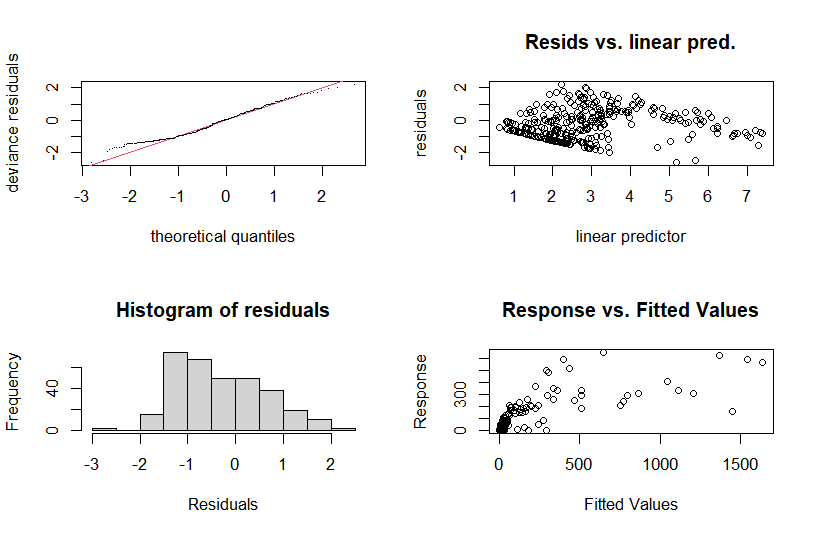


**Figure 14S**. The extracted plots of the GAM model for pH.

1. GAM model for PO4

*gam.numsp.PO4 <- gam(num.species ~ s(Lat,Lon, bs="sos")+ s(num.record)+s(PO4), data = data, family = "nb", method = "REML", select = TRUE)*

*> gam.check(gam.numsp.PO4)*

Method: REML Optimizer: outer newton

full convergence after 11 iterations.

Gradient range [-0.0002262481,-4.181222e-06]

(score 1354.46 & scale 1).

Hessian positive definite, eigenvalue range [2.264207e-05,144.1696].

Model rank = 68 / 68

Basis dimension (k) checking results. Low p-value (k-index<1) may

indicate that k is too low, especially if edf is close to k'.

k' edf k-index p-value

s(Lat,Lon) 49.00000 28.35061 0.93 0.78

s(num.record) 9.00000 8.55003 0.32 <2e-16 ***

s(PO4) 9.00000 0.00116 0.83 0.08 .

Signif. codes: 0 ‘***’ 0.001 ‘**’ 0.01 ‘*’ 0.05 ‘.’ 0.1 ‘ ’ 1

*summary(gam.numsp.PO4)*

Family: Negative Binomial(0.915)

Link function: log

Formula:

num.species ~ s(Lat, Lon, bs = "sos") + s(num.record) + s(PO4)

Parametric coefficients:

Estimate Std. Error z value Pr(>|z|)

(Intercept) 2.88566 0.06069 47.55 <2e-16 ***

Signif. codes: 0 ‘***’ 0.001 ‘**’ 0.01 ‘*’ 0.05 ‘.’ 0.1 ‘ ’ 1

Approximate significance of smooth terms:

edf Ref.df Chi.sq p-value

s(Lat,Lon) 28.350608 49 136.758 <2e-16 ***

s(num.record) 8.550031 9 234.785 <2e-16 ***

s(PO4) 0.001158 9 0.001 0.437

Signif. codes: 0 ‘***’ 0.001 ‘**’ 0.01 ‘*’ 0.05 ‘.’ 0.1 ‘ ’ 1

R-sq.(adj) = -1.17 Deviance explained = 67.8%

-REML = 1354.5 Scale est. = 1 n = 325


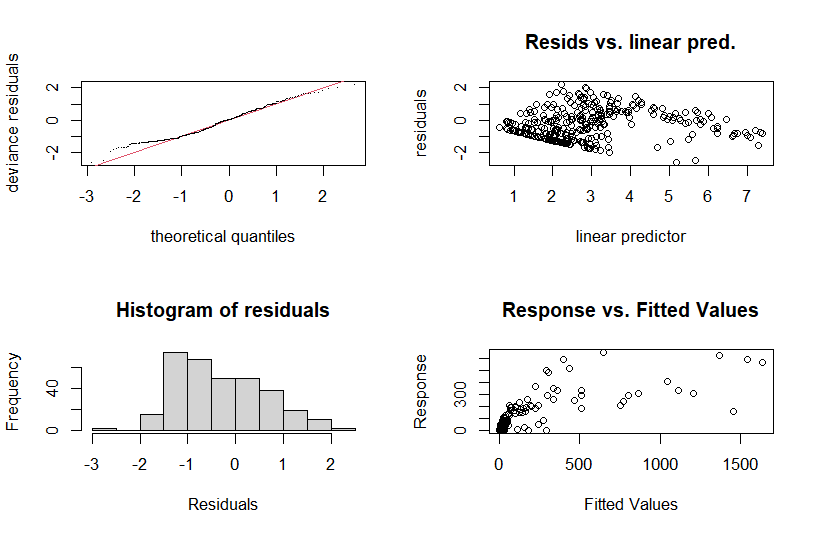


**Figure 15S**. The extracted plots of the GAM model for PO4.

1. GAM model for salinity

*gam.numsp.salinity <- gam(num.species ~ s(Lat,Lon, bs="sos")+ s(num.record)+s(salinity), data = data, family = "nb", method = "REML", select = TRUE)*

*> gam.check(gam.numsp.salinity)*

Method: REML Optimizer: outer newton

full convergence after 15 iterations.

Gradient range [-3.089521e-05,2.070163e-05]

(score 1354.46 & scale 1).

Hessian positive definite, eigenvalue range [2.304689e-05,144.1708].

Model rank = 68 / 68

Basis dimension (k) checking results. Low p-value (k-index<1) may

indicate that k is too low, especially if edf is close to k'.

k' edf k-index p-value

s(Lat,Lon) 4.90e+01 2.84e+01 0.93 0.78

s(num.record) 9.00e+00 8.55e+00 0.32 <2e-16 ***

s(salinity) 9.00e+00 1.27e-04 0.87 0.29

Signif. codes: 0 ‘***’ 0.001 ‘**’ 0.01 ‘*’ 0.05 ‘.’ 0.1 ‘ ’ 1

*summary(gam.numsp.salinity)*

Family: Negative Binomial(0.915)

Link function: log

Formula:

num.species ~ s(Lat, Lon, bs = "sos") + s(num.record) + s(salinity)

Parametric coefficients:

Estimate Std. Error z value Pr(>|z|)

(Intercept) 2.88566 0.06069 47.55 <2e-16 ***

Signif. codes: 0 ‘***’ 0.001 ‘**’ 0.01 ‘*’ 0.05 ‘.’ 0.1 ‘ ’ 1

Approximate significance of smooth terms:

edf Ref.df Chi.sq p-value

s(Lat,Lon) 2.835e+01 49 136.8 <2e-16 ***

s(num.record) 8.550e+00 9 234.8 <2e-16 ***

s(salinity) 1.273e-04 9 0.0 0.663

Signif. codes: 0 ‘***’ 0.001 ‘**’ 0.01 ‘*’ 0.05 ‘.’ 0.1 ‘ ’ 1

R-sq.(adj) = -1.17 Deviance explained = 67.8%

-REML = 1354.5 Scale est. = 1 n = 325


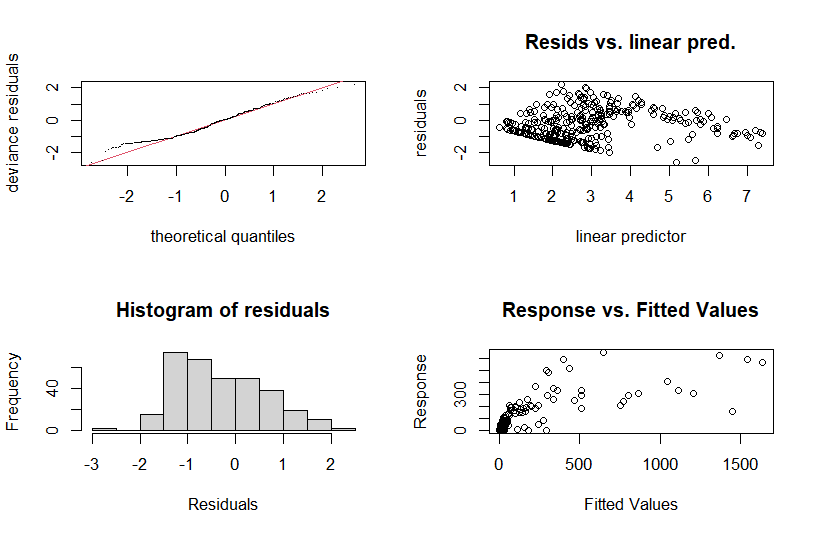


**Figure 16S**. The extracted plots of the GAM model for salinity.

1. GAM model for dissolved oxygen

*gam.numsp.dissolved.oxygen <- gam(num.species ~ s(Lat,Lon, bs="sos")+ s(num.record)+s(dissolved.oxygen), data = data, family = "nb", method = "REML", select = TRUE)*

*> gam.check(gam.numsp.dissolved.oxygen)*

Method: REML Optimizer: outer newton

full convergence after 11 iterations.

Gradient range [-0.0006221328,0.0002205194]

(score 1354.46 & scale 1).

Hessian positive definite, eigenvalue range [4.514023e-05,144.1704].

Model rank = 68 / 68

Basis dimension (k) checking results. Low p-value (k-index<1) may

indicate that k is too low, especially if edf is close to k'.

k' edf k-index p-value

s(Lat,Lon) 49.00000 28.35041 0.93 0.81

s(num.record) 9.00000 8.55003 0.32 <2e-16 ***

s(dissolved.oxygen) 9.00000 0.00253 0.89 0.48

Signif. codes: 0 ‘***’ 0.001 ‘**’ 0.01 ‘*’ 0.05 ‘.’ 0.1 ‘ ’ 1

*summary(gam.numsp.dissolved.oxygen)*

Family: Negative Binomial(0.915)

Link function: log

Formula:

num.species ~ s(Lat, Lon, bs = "sos") + s(num.record) + s(dissolved.oxygen)

Parametric coefficients:

Estimate Std. Error z value Pr(>|z|)

(Intercept) 2.88566 0.06069 47.55 <2e-16 ***

Signif. codes: 0 ‘***’ 0.001 ‘**’ 0.01 ‘*’ 0.05 ‘.’ 0.1 ‘ ’ 1

Approximate significance of smooth terms:

edf Ref.df Chi.sq p-value

s(Lat,Lon) 28.350414 49 136.731 <2e-16 ***

s(num.record) 8.550030 9 234.771 <2e-16 ***

s(dissolved.oxygen) 0.002531 9 0.001 0.694

Signif. codes: 0 ‘***’ 0.001 ‘**’ 0.01 ‘*’ 0.05 ‘.’ 0.1 ‘ ’ 1

R-sq.(adj) = -1.17 Deviance explained = 67.8%

-REML = 1354.5 Scale est. = 1 n = 325


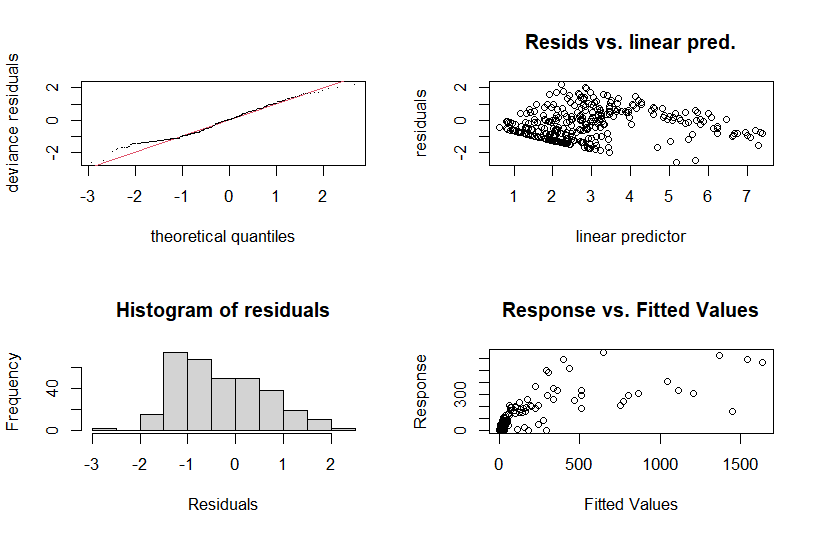


**Figure 17S**. The extracted plots of the GAM model for dissolved oxygen.

1. GAM model for temprature

*gam.numsp.temp <- gam(num.species ~ s(Lat,Lon, bs="sos")+ s(num.record)+s(temp), data = data, family = "nb", method = "REML", select = TRUE)*

*> gam.check(gam.numsp.temp)*

Method: REML Optimizer: outer newton

full convergence after 11 iterations.

Gradient range [-8.101012e-05,5.667138e-05]

(score 1354.46 & scale 1).

Hessian positive definite, eigenvalue range [1.960706e-05,144.1704].

Model rank = 68 / 68

Basis dimension (k) checking results. Low p-value (k-index<1) may

indicate that k is too low, especially if edf is close to k'.

k' edf k-index p-value

s(Lat,Lon) 4.90e+01 2.84e+01 0.93 0.80

s(num.record) 9.00e+00 8.55e+00 0.32 <2e-16 ***

s(temp) 9.00e+00 4.45e-04 0.91 0.64

Signif. codes: 0 ‘***’ 0.001 ‘**’ 0.01 ‘*’ 0.05 ‘.’ 0.1 ‘ ’ 1

*summary(gam.numsp.temp)*

Family: Negative Binomial(0.915)

Link function: log

Formula:

num.species ~ s(Lat, Lon, bs = "sos") + s(num.record) + s(temp)

Parametric coefficients:

Estimate Std. Error z value Pr(>|z|)

(Intercept) 2.88566 0.06069 47.55 <2e-16 ***

Signif. codes: 0 ‘***’ 0.001 ‘**’ 0.01 ‘*’ 0.05 ‘.’ 0.1 ‘ ’ 1

Approximate significance of smooth terms:

edf Ref.df Chi.sq p-value

s(Lat,Lon) 2.835e+01 49 136.8 <2e-16 ***

s(num.record) 8.550e+00 9 234.8 <2e-16 ***

s(temp) 4.449e-04 9 0.0 0.525

Signif. codes: 0 ‘***’ 0.001 ‘**’ 0.01 ‘*’ 0.05 ‘.’ 0.1 ‘ ’ 1

R-sq.(adj) = -1.17 Deviance explained = 67.8%

-REML = 1354.5 Scale est. = 1 n = 325


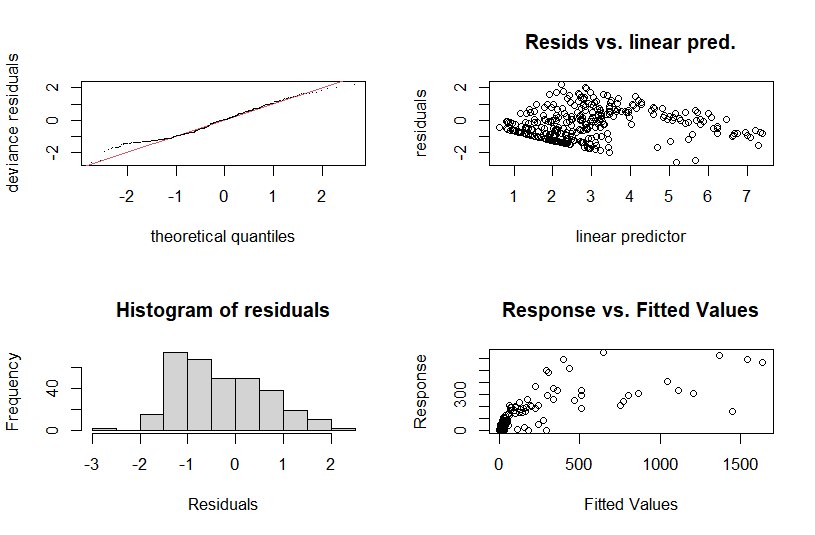


**Figure 18S**. The extracted plots of the GAM model for temperature.

1. GAM model for the combination of environmental variables

*gam.numsp.env <- gam(num.species ~ s(Lat,Lon, bs="sos")+ s(num.record)+ s (temp) + s(dissolved.oxygen) + s(chlorophyll) + s(current) + s(pH) + s(salinity) + s(depth)+ s(PO4) +s (current) , data = data, family = "nb", method= "REML", select = TRUE)*

*> gam.check(gam.numsp.env)*

Method: REML Optimizer: outer newton

full convergence after 17 iterations.

Gradient range [-0.002122123,2.721997e-05]

(score 1354.459 & scale 1).

Hessian positive definite, eigenvalue range [3.724563e-07,143.8939].

Model rank = 131 / 131

Basis dimension (k) checking results. Low p-value (k-index<1) may

indicate that k is too low, especially if edf is close to k'.

k' edf k-index p-value

s(Lat,Lon) 4.90e+01 2.84e+01 0.93 0.860

s(num.record) 9.00e+00 8.55e+00 0.32 <2e-16 ***

s(temp) 9.00e+00 1.37e-03 0.91 0.570

s(dissolved.oxygen) 9.00e+00 4.97e-04 0.89 0.490

s(chlorophyll) 9.00e+00 1.03e-01 0.99 0.960

s(current) 9.00e+00 6.68e-04 0.93 0.765

s(pH) 9.00e+00 7.28e-04 0.93 0.805

s(salinity) 9.00e+00 3.70e-04 0.87 0.330

s(depth) 9.00e+00 5.16e-04 0.83 0.095 .

s(PO4) 9.00e+00 8.80e-03 0.83 0.115

Signif. codes: 0 ‘***’ 0.001 ‘**’ 0.01 ‘*’ 0.05 ‘.’ 0.1 ‘ ’ 1

*summary(gam.numsp.env)*

Family: Negative Binomial(0.916)

Link function: log

Formula:

num.species ~ s(Lat, Lon, bs = "sos") + s(num.record) + s(temp) +

s(dissolved.oxygen) + s(chlorophyll) + s(current) + s(pH) +

s(salinity) + s(depth) + s(PO4) + s(current)

Parametric coefficients:

Estimate Std. Error z value Pr(>|z|)

(Intercept) 2.88473 0.06067 47.55 <2e-16 ***

Signif. codes: 0 ‘***’ 0.001 ‘**’ 0.01 ‘*’ 0.05 ‘.’ 0.1 ‘ ’ 1

Approximate significance of smooth terms:

edf Ref.df Chi.sq p-value

s(Lat,Lon) 2.843e+01 49 136.982 <2e-16 ***

s(num.record) 8.551e+00 9 234.593 <2e-16 ***

s(temp) 1.369e-03 9 0.001 0.550

s(dissolved.oxygen) 4.975e-04 9 0.000 0.787

s(chlorophyll) 1.025e-01 9 0.094 0.337

s(current) 6.681e-04 9 0.000 0.540

s(pH) 7.285e-04 9 0.000 0.574

s(salinity) 3.698e-04 9 0.000 0.664

s(depth) 5.165e-04 9 0.000 0.889

s(PO4) 8.795e-03 9 0.006 0.409

---

Signif. codes: 0 ‘***’ 0.001 ‘**’ 0.01 ‘*’ 0.05 ‘.’ 0.1 ‘ ’ 1

R-sq.(adj) = -1.16 Deviance explained = 67.9%

-REML = 1354.5 Scale est. = 1 n = 325


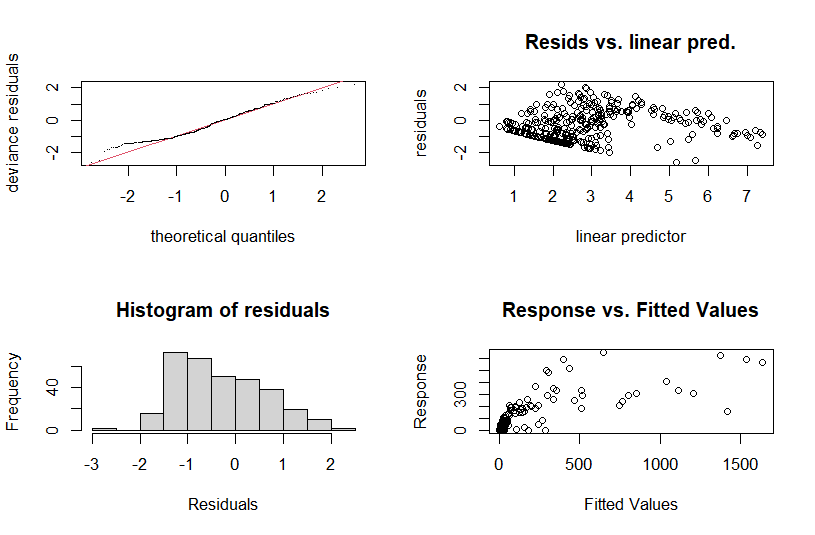


**Figure 19S**. The extracted plots of the GAM model for the combination of environmental variables.

## Model selection for the number of species based on GAM analysis for shallow water

*> gam.numsp.models <- list(gam.numsp.intercept = gam.numsp.intercept,*

*+ gam.numsp.numrec = gam.numsp.numrec,*

*+ gam.numsp.env=gam.numsp.env,*

*+ gam.numsp.latlon = gam.numsp.latlon,*

*+ gam.numsp.depth = gam.numsp.depth,*

*+ gam.numsp.pH = gam.numsp.pH,*

*+ gam.numsp.current = gam.numsp.current,*

*+ gam.numsp.PO4 = gam.numsp.PO4,*

*+ gam.numsp.chlorophyll= gam.numsp.chlorophyll,*

*+ gam.numsp.dissolved.oxygen = gam.numsp.dissolved.oxygen,*

*+ gam.numsp.salinity= gam.numsp.salinity,*

*+ gam.numsp.temp= gam.numsp.temp)*

*> gam.numsp.aic.df <- data.frame(Model = names(gam.numsp.models),*

*+ AIC = sapply(gam.numsp.models, function(x) x$aic),*

*+ akaike.weights(sapply(gam.numsp.models, function(x) x$aic)))*

*> gam.numsp.aic.df <- gam.numsp.aic.df[order(gam.numsp.aic.df$AIC),]*

*> gam.numsp.aic.df$Cumulative.Weight <- cumsum(gam.numsp.aic.df$weights)*

*> kable(gam.numsp.aic.df, row.names = FALSE)*

|  |  |  | | |  | |  | |  | |
| --- | --- | --- | --- | --- | --- | --- | --- | --- | --- | --- |
|  |  |  | | |  | |  | |  | |
| Model | AIC | | deltaAIC | rel.LL | | weights | | Cumulative.  Weight | |  |
| gam.numsp.chlorophyll | 2630.7010 | | 0.0000 | 1.0000 | | 0.1111 | | 0.1111 | |  |
| gam.numsp.env | 2630.7775 | | 0.0765 | 0.9625 | | 0.1069 | | 0.2180 | |  |
| gam.numsp.latlon | 2630.9557 | | 0.2547 | 0.8804 | | 0.0978 | | 0.3158 | |  |
| gam.numsp.salinity | 2630.9558 | | 0.2548 | 0.8804 | | 0.0978 | | 0.4137 | |  |
| gam.numsp.temp | 2630.9560 | | 0.2549 | 0.8803 | | 0.0978 | | 0.5115 | |  |
| gam.numsp.PO4 | 2630.9565 | | 0.2555 | 0.8801 | | 0.0978 | | 0.6092 | |  |
| gam.numsp.depth | 2630.9566 | | 0.2556 | 0.8800 | | 0.0978 | | 0.7070 | |  |
| gam.numsp.pH | 2630.9584 | | 0.2574 | 0.8792 | | 0.0977 | | 0.8047 | |  |
| gam.numsp.current | 2630.9588 | | 0.2578 | 0.8791 | | 0.0977 | | 0.9023 | |  |
| gam.numsp.dissolved.oxygen | 2630.9590 | | 0.2580 | 0.8790 | | 0.0977 | | 1.0000 | |  |
| gam.numsp.numrec | 2703.6379 | | 72.9369 | 0.0000 | | 0.0000 | | 1.0000 | |  |
| gam.numsp.intercept | 2973.6480 | | 342.9470 | 0.0000 | | 0.0000 | | 1.0000 | |  |

The results show that chlorophyll is the best-fitted model. In the following, the combination of environmental parameters and also the parameters including geographical position, salinity, temperature, PO4, depth, pH, current, and dissolved oxygen could be modeled for the distribution of the number of species in shallow waters.

## The Plots of GMAs analysis of the number of species in the shallow water


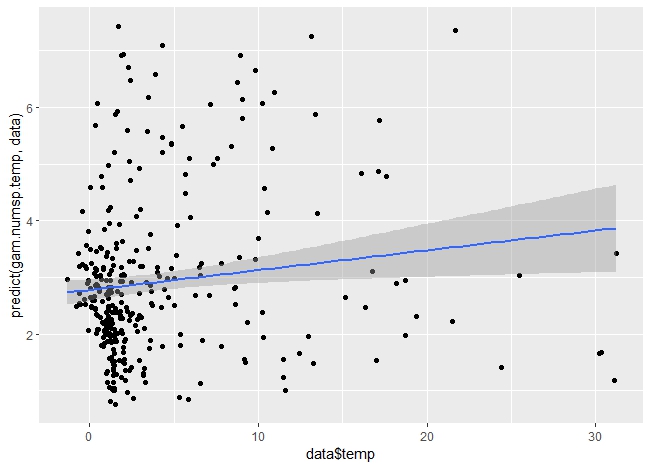


**Figure 20S.** qplot(data$temp, predict(gam.numsp.temp, data)) + geom_smooth(method = "glm")


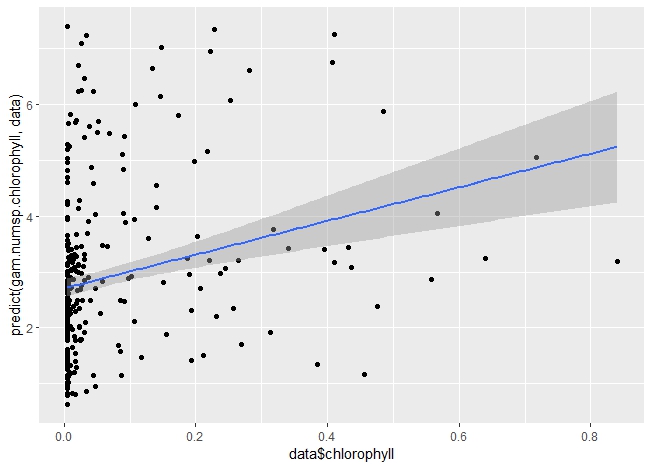


**Figure 21S.** qplot(data$chlorophyll, predict(gam.numsp.chlorophyll, data)) + geom_smooth(method = "glm")


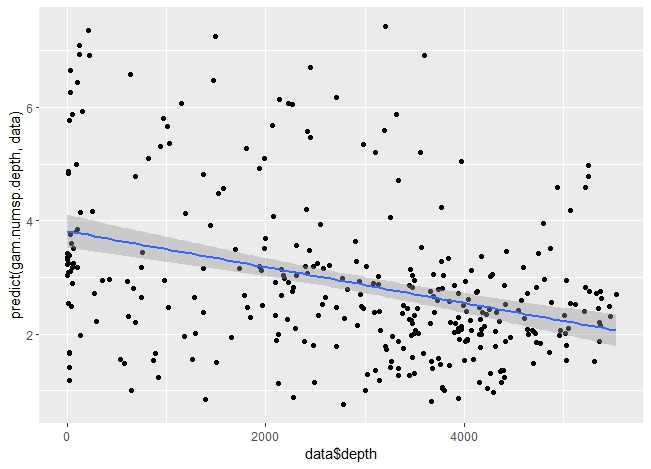


**Figure 22S.** qplot(data$depth, predict(gam.numsp.depth, data)) + geom_smooth(method = "glm")


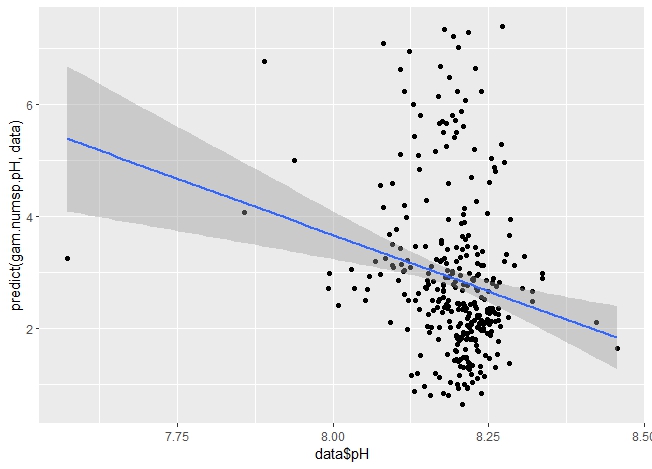


**Figure 23S.** qplot(data$pH, predict(gam.numsp.pH, data)) + geom_smooth(method = "glm")


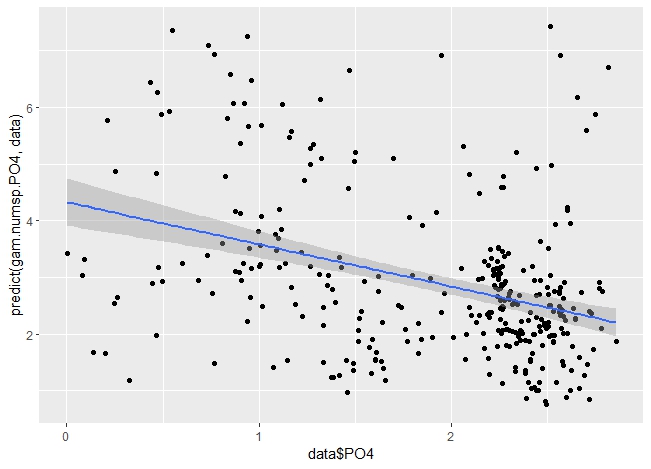


**Figure 24S.** qplot(data$PO4, predict(gam.numsp.PO4, data)) + geom_smooth(method = "glm")


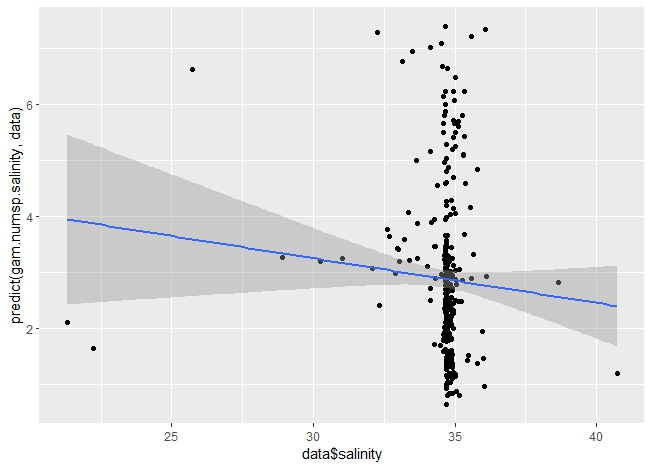


**Figure 25S.** qplot(data$salinity, predict(gam.numsp.salinity, data)) + geom_smooth(method = "glm")


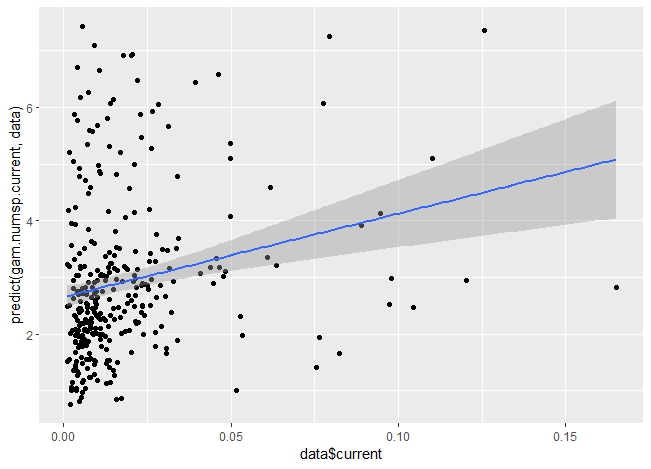


**Figure 26S.** qplot(data$current, predict(gam.numsp.current, data)) + geom_smooth(method = "glm")


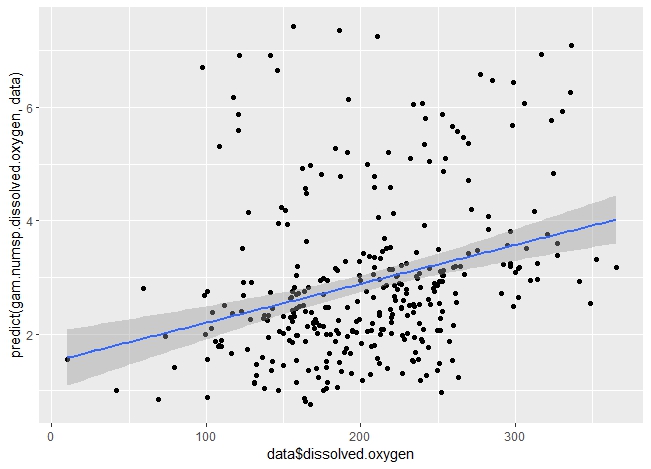


**Figure 27S.** qplot(data$dissolved.oxygen, predict(gam.numsp.dissolved.oxygen, data)) + geom_smooth(method = "glm")

# GAMs for ES50, shallow water

This part presents the script, figures, and plots of results from GAM analysis for the ES50 in shallow water.

## The script and extracted figures of GAM models for ES50 in shallow water.

1. GAM model for intercept

*gam.ES50.intercept <- gam(ES50 ~ 1, data = data, family = "nb", method = "REML", select = TRUE)> gam.check(gam.ES50.intercept)*

Method: REML Optimizer: outer newton

full convergence after 2 iterations.

Gradient range [-9.489043e-05,-9.489043e-05]

(score 946.8012 & scale 1).

Hessian positive definite, eigenvalue range [103.7359,103.7359].

Model rank = 1 / 1

*summary(gam.ES50.intercept)*

Family: Negative Binomial(1.586)

Link function: log

Formula: ES50 ~ 1

Parametric coefficients:

Estimate Std. Error z value Pr(>|z|)

(Intercept) 1.86853 0.04914 38.03 <2e-16 ***

Signif. codes: 0 ‘***’ 0.001 ‘**’ 0.01 ‘*’ 0.05 ‘.’ 0.1 ‘ ’ 1

R-sq.(adj) = 0 Deviance explained = 4.92e-09%

-REML = 946.8 Scale est. = 1 n = 325


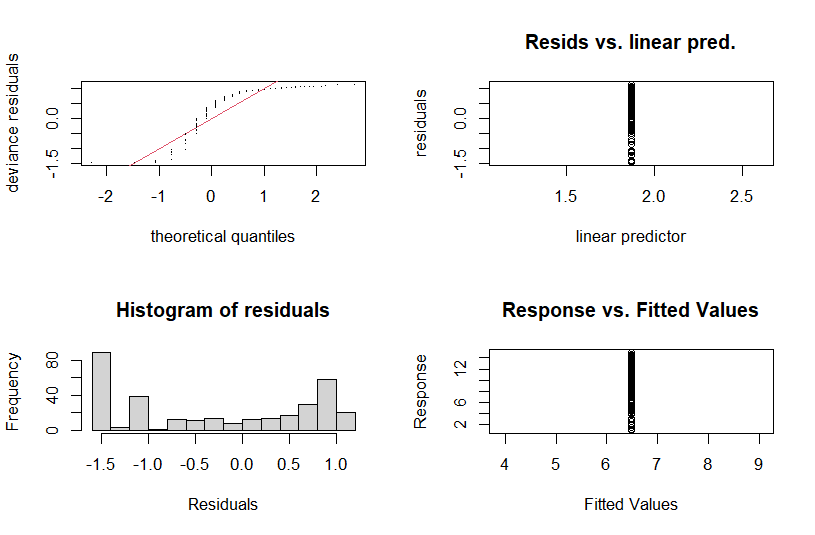


**Figure 28S.** The extracted plots of GAM model for intercept.

1. GAM model for spatial autocorrelation

*gam.ES50.latlon <- gam(ES50 ~ s(Lat, Lon, bs = "sos") , data = data, family= "nb", method = "REML", select = TRUE)*

*> gam.check(gam.ES50.latlon)*

Method: REML Optimizer: outer newton

full convergence after 4 iterations.

Gradient range [-8.086785e-05,7.468807e-05]

(score 928.3212 & scale 1).

Hessian positive definite, eigenvalue range [5.792978,64.95963].

Model rank = 50 / 50

Basis dimension (k) checking results. Low p-value (k-index<1) may

indicate that k is too low, especially if edf is close to k'.

k' edf k-index p-value

s(Lat,Lon) 49.0 28.4 0.97 0.64

summary(gam.ES50.latlon)

Family: Negative Binomial(2.413)

Link function: log

Formula:

ES50 ~ s(Lat, Lon, bs = "sos")

Parametric coefficients:

Estimate Std. Error z value Pr(>|z|)

(Intercept) 1.7503 0.0431 40.62 <2e-16 ***

Signif. codes: 0 ‘***’ 0.001 ‘**’ 0.01 ‘*’ 0.05 ‘.’ 0.1 ‘ ’ 1

Approximate significance of smooth terms:

edf Ref.df Chi.sq p-value

s(Lat,Lon) 28.36 49 124.2 <2e-16 ***

Signif. codes: 0 ‘***’ 0.001 ‘**’ 0.01 ‘*’ 0.05 ‘.’ 0.1 ‘ ’ 1

R-sq.(adj) = 0.298 Deviance explained = 32.4%

-REML = 928.32 Scale est. = 1 n = 325


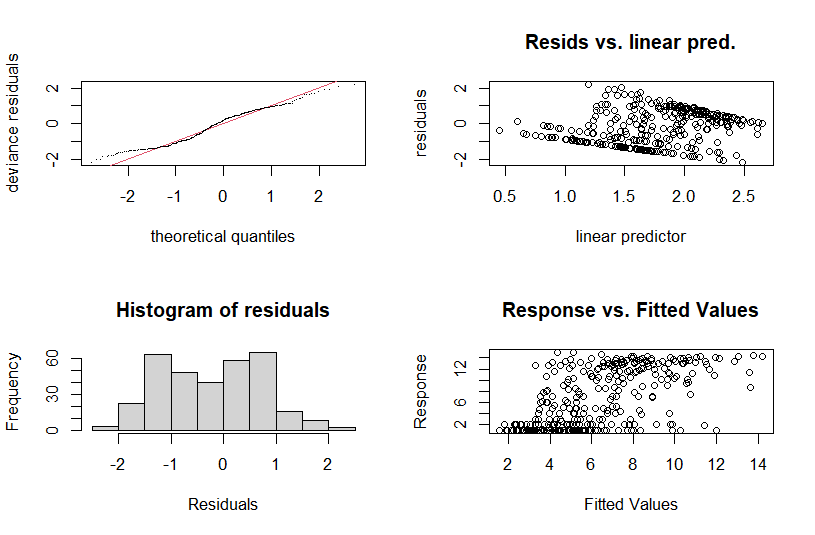


**Figure 29S.** The extracted plots of GAM model for spatial autocorrelation.

1. GAM model for depth

*gam.ES50.depth <- gam(ES50 ~ s(Lat,Lon, bs="sos")+ +s(depth), data = data, family = "nb", method = "REML", select = TRUE)*

*> gam.check(gam.ES50.depth)*

Method: REML Optimizer: outer newton

full convergence after 7 iterations.

Gradient range [-0.002547403,0.0009930553]

(score 928.3218 & scale 1).

Hessian positive definite, eigenvalue range [8.236143e-05,64.96077].

Model rank = 59 / 59

Basis dimension (k) checking results. Low p-value (k-index<1) may

indicate that k is too low, especially if edf is close to k'.

k' edf k-index p-value

s(Lat,Lon) 49.00000 28.35988 0.97 0.64

s(depth) 9.00000 0.00539 0.87 0.06 .

Signif. codes: 0 ‘***’ 0.001 ‘**’ 0.01 ‘*’ 0.05 ‘.’ 0.1 ‘ ’ 1

*summary(gam.ES50.depth)*

Family: Negative Binomial(2.413)

Link function: log

Formula:

ES50 ~ s(Lat, Lon, bs = "sos") + +s(depth)

Parametric coefficients:

Estimate Std. Error z value Pr(>|z|)

(Intercept) 1.7503 0.0431 40.61 <2e-16 ***

Signif. codes: 0 ‘***’ 0.001 ‘**’ 0.01 ‘*’ 0.05 ‘.’ 0.1 ‘ ’ 1

Approximate significance of smooth terms:

edf Ref.df Chi.sq p-value

s(Lat,Lon) 28.359882 49 124.063 <2e-16 ***

s(depth) 0.005395 9 0.004 0.408

Signif. codes: 0 ‘***’ 0.001 ‘**’ 0.01 ‘*’ 0.05 ‘.’ 0.1 ‘ ’ 1

R-sq.(adj) = 0.298 Deviance explained = 32.4%

-REML = 928.32 Scale est. = 1 n = 325


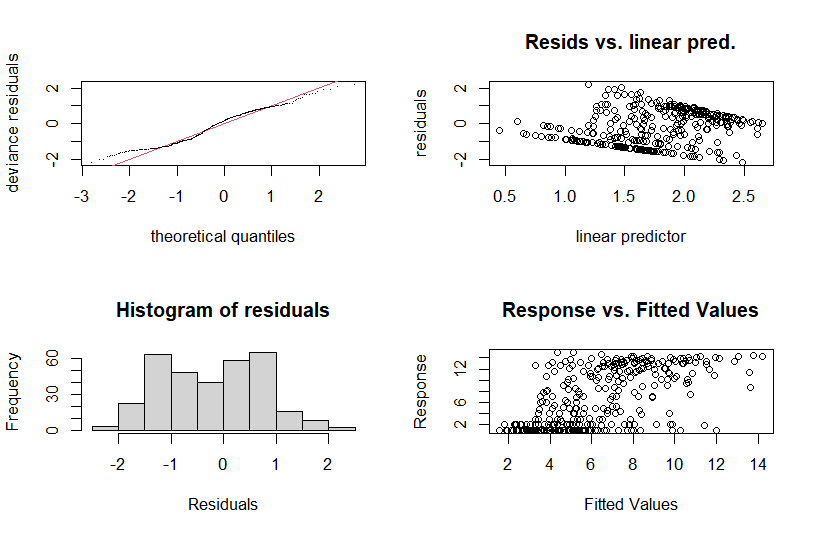


**Figure 30S.** The extracted plots of GAM model for depth

1. GAM model for chlorophyll

*gam.ES50.chlorophyll <- gam(ES50 ~ s(Lat,Lon, bs="sos")+ s(chlorophyll), data = data, family = "nb", method = "REML", select = TRUE)*

*> gam.check(gam.ES50.chlorophyll)*

Method: REML Optimizer: outer newton

full convergence after 8 iterations.

Gradient range [-0.0003131868,0.0002404984]

(score 928.3216 & scale 1).

Hessian positive definite, eigenvalue range [6.511457e-06,64.95974].

Model rank = 59 / 59

Basis dimension (k) checking results. Low p-value (k-index<1) may

indicate that k is too low, especially if edf is close to k'.

k' edf k-index p-value

s(Lat,Lon) 4.90e+01 2.84e+01 0.97 0.62

s(chlorophyll) 9.00e+00 7.06e-04 1.03 0.94

*summary(gam.ES50.chlorophyll)*

Family: Negative Binomial(2.413)

Link function: log

Formula:

ES50 ~ s(Lat, Lon, bs = "sos") + s(chlorophyll)

Parametric coefficients:

Estimate Std. Error z value Pr(>|z|)

(Intercept) 1.7503 0.0431 40.62 <2e-16 ***

---

Signif. codes: 0 ‘***’ 0.001 ‘**’ 0.01 ‘*’ 0.05 ‘.’ 0.1 ‘ ’ 1

Approximate significance of smooth terms:

edf Ref.df Chi.sq p-value

s(Lat,Lon) 28.361042 49 124.2 <2e-16 ***

s(chlorophyll) 0.000706 9 0.0 0.931

---

Signif. codes: 0 ‘***’ 0.001 ‘**’ 0.01 ‘*’ 0.05 ‘.’ 0.1 ‘ ’ 1

R-sq.(adj) = 0.298 Deviance explained = 32.4%

-REML = 928.32 Scale est. = 1 n = 325


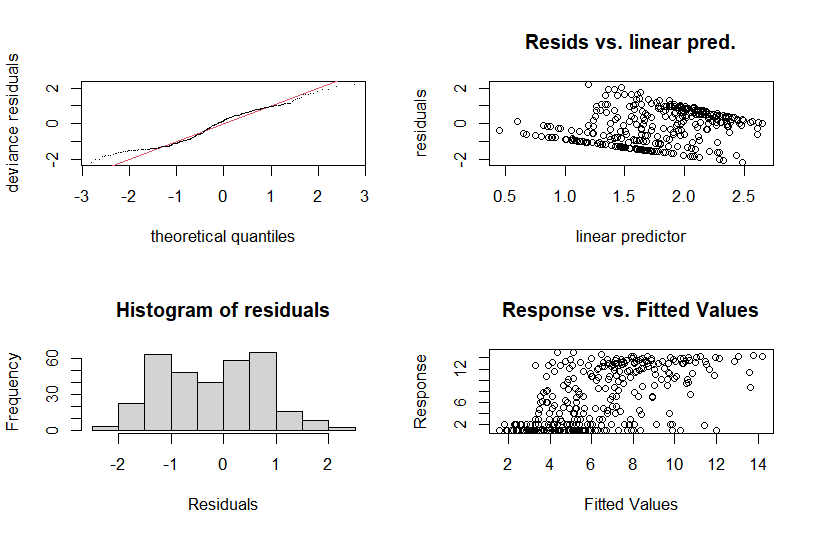


**Figure 31S.** The extracted plots of GAM model for chlorophyll.

1. GAM model for current

*gam.ES50.current <- gam(ES50 ~ s(Lat,Lon, bs="sos")+s(current), data = data, family = "nb", method = "REML", select = TRUE)*

*> gam.check(gam.ES50.current)*

Method: REML Optimizer: outer newton

full convergence after 9 iterations.

Gradient range [-0.0001829774,0.0001939054]

(score 928.3214 & scale 1).

Hessian positive definite, eigenvalue range [3.019701e-05,64.95984].

Model rank = 59 / 59

Basis dimension (k) checking results. Low p-value (k-index<1) may

indicate that k is too low, especially if edf is close to k'.

k' edf k-index p-value

s(Lat,Lon) 4.90e+01 2.84e+01 0.97 0.64

s(current) 9.00e+00 6.68e-04 0.94 0.42

summary(gam. ES50.current)

Family: Negative Binomial(2.413)

Link function: log

Formula:

ES50 ~ s(Lat, Lon, bs = "sos") + s(current)

Parametric coefficients:

Estimate Std. Error z value Pr(>|z|)

(Intercept) 1.7503 0.0431 40.62 <2e-16 ***

Signif. codes: 0 ‘***’ 0.001 ‘**’ 0.01 ‘*’ 0.05 ‘.’ 0.1 ‘ ’ 1

Approximate significance of smooth terms:

edf Ref.df Chi.sq p-value

s(Lat,Lon) 2.836e+01 49 124.2 <2e-16 ***

s(current) 6.681e-04 9 0.0 0.611

Signif. codes: 0 ‘***’ 0.001 ‘**’ 0.01 ‘*’ 0.05 ‘.’ 0.1 ‘ ’ 1

R-sq.(adj) = 0.298 Deviance explained = 32.4%

-REML = 928.32 Scale est. = 1 n = 325


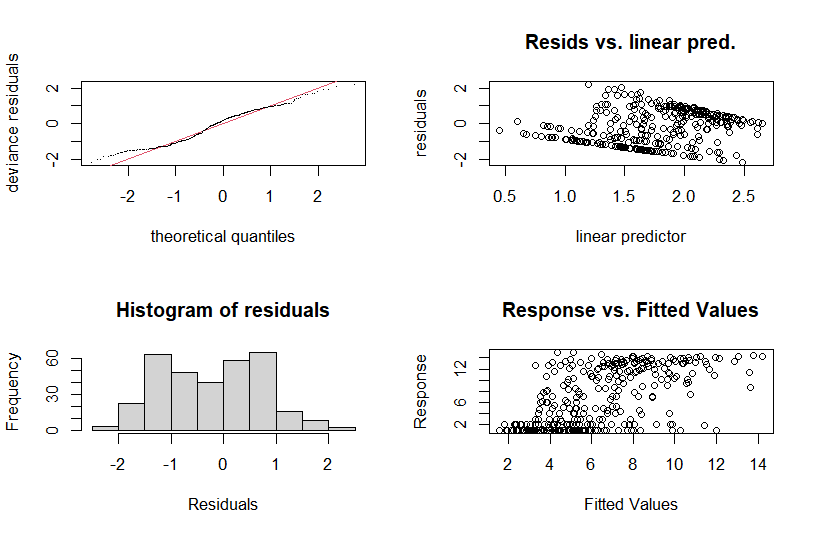


**Figure 32S.** The extracted plots of GAM model for current

1. GAM model for pH

*gam.ES50.pH <- gam(ES50 ~ s(Lat,Lon, bs="sos")+s(pH), data = data, family = "nb", method = "REML", select = TRUE)*

*> gam.check(gam.ES50.pH)*

Method: REML Optimizer: outer newton

full convergence after 5 iterations.

Gradient range [-0.0002716055,0.0002111231]

(score 928.2736 & scale 1).

Hessian positive definite, eigenvalue range [0.0002714663,64.76775].

Model rank = 59 / 59

Basis dimension (k) checking results. Low p-value (k-index<1) may

indicate that k is too low, especially if edf is close to k'.

k' edf k-index p-value

s(Lat,Lon) 49.000 28.228 0.97 0.59

s(pH) 9.000 0.487 0.97 0.64

*summary(gam.ES50.pH)*

Family: Negative Binomial(2.416)

Link function: log

Formula:

ES50 ~ s(Lat, Lon, bs = "sos") + s(pH)

Parametric coefficients:

Estimate Std. Error z value Pr(>|z|)

(Intercept) 1.74994 0.04308 40.62 <2e-16 ***

Signif. codes: 0 ‘***’ 0.001 ‘**’ 0.01 ‘*’ 0.05 ‘.’ 0.1 ‘ ’ 1

Approximate significance of smooth terms:

edf Ref.df Chi.sq p-value

s(Lat,Lon) 28.2275 49 120.471 <2e-16 ***

s(pH) 0.4866 9 0.622 0.236

Signif. codes: 0 ‘***’ 0.001 ‘**’ 0.01 ‘*’ 0.05 ‘.’ 0.1 ‘ ’ 1

R-sq.(adj) = 0.298 Deviance explained = 32.5%

-REML = 928.27 Scale est. = 1 n = 325


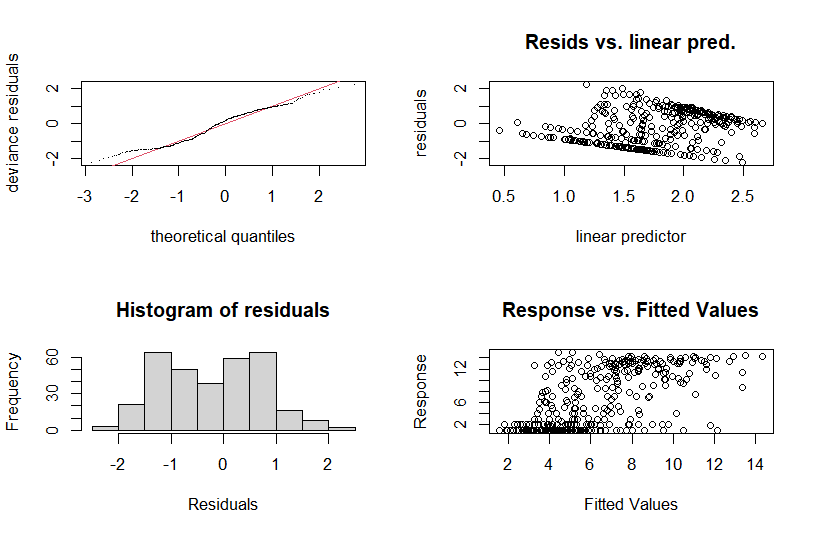


**Figure 33S.** The extracted plots of GAM model for pH.

1. GAM model for PO4

*gam.ES50.PO4 <- gam(ES50 ~ s(Lat,Lon, bs="sos")+s(PO4), data = data, family = "nb", method = "REML", select = TRUE)*

*> gam.check(gam.ES50.PO4)*

Method: REML Optimizer: outer newton

full convergence after 7 iterations.

Gradient range [-1.827103e-05,1.714747e-05]

(score 928.3213 & scale 1).

Hessian positive definite, eigenvalue range [1.272822e-05,64.95968].

Model rank = 59 / 59

Basis dimension (k) checking results. Low p-value (k-index<1) may

indicate that k is too low, especially if edf is close to k'.

k' edf k-index p-value

s(Lat,Lon) 4.90e+01 2.84e+01 0.97 0.60

s(PO4) 9.00e+00 7.84e-05 0.90 0.13

*summary(gam.ES50.PO4)*

Family: Negative Binomial(2.413)

Link function: log

Formula:

ES50 ~ s(Lat, Lon, bs = "sos") + s(PO4)

Parametric coefficients:

Estimate Std. Error z value Pr(>|z|)

(Intercept) 1.7503 0.0431 40.62 <2e-16 ***

Signif. codes: 0 ‘***’ 0.001 ‘**’ 0.01 ‘*’ 0.05 ‘.’ 0.1 ‘ ’ 1

Approximate significance of smooth terms:

edf Ref.df Chi.sq p-value

s(Lat,Lon) 2.836e+01 49 124.2 <2e-16 ***

s(PO4) 7.840e-05 9 0.0 0.874

Signif. codes: 0 ‘***’ 0.001 ‘**’ 0.01 ‘*’ 0.05 ‘.’ 0.1 ‘ ’ 1

R-sq.(adj) = 0.298 Deviance explained = 32.4%

-REML = 928.32 Scale est. = 1 n = 325


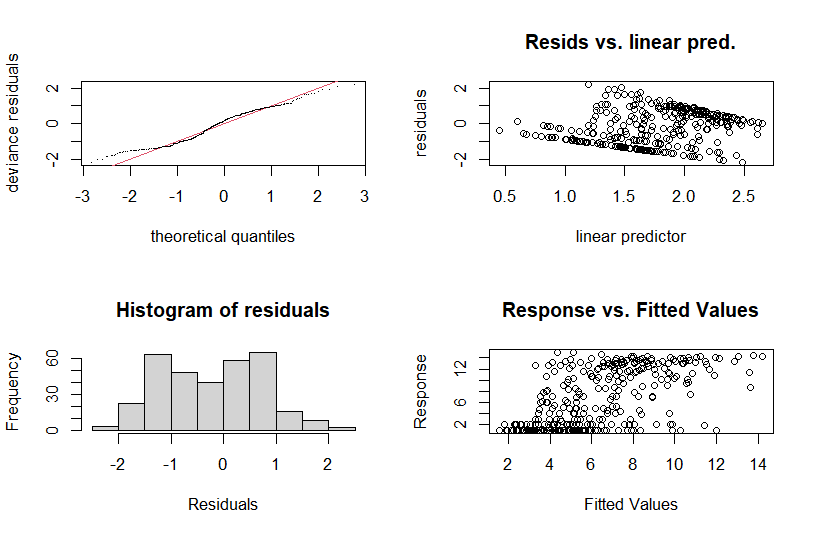


**Figure 341S.** The extracted plots of GAM model for PO4.

1. GAM model for salinity

*gam.ES50.salinity <- gam(ES50 ~ s(Lat,Lon, bs="sos")+ s(salinity), data = data, family = "nb", method = "REML", select = TRUE)*

*> gam.check(gam.ES50.salinity)*

Method: REML Optimizer: outer newton

full convergence after 8 iterations.

Gradient range [-0.0003391053,0.0001757611]

(score 928.3216 & scale 1).

Hessian positive definite, eigenvalue range [7.127976e-06,64.95969].

Model rank = 59 / 59

Basis dimension (k) checking results. Low p-value (k-index<1) may

indicate that k is too low, especially if edf is close to k'.

k' edf k-index p-value

s(Lat,Lon) 49.00000 28.36101 0.97 0.71

s(salinity) 9.00000 0.00074 0.90 0.14

summary(gam.ES50.salinity)

Family: Negative Binomial(2.413)

Link function: log

Formula:

ES50 ~ s(Lat, Lon, bs = "sos") + s(salinity)

Parametric coefficients:

Estimate Std. Error z value Pr(>|z|)

(Intercept) 1.7503 0.0431 40.62 <2e-16 ***

Signif. codes: 0 ‘***’ 0.001 ‘**’ 0.01 ‘*’ 0.05 ‘.’ 0.1 ‘ ’ 1

Approximate significance of smooth terms:

edf Ref.df Chi.sq p-value

s(Lat,Lon) 2.836e+01 49 124.2 <2e-16 ***

s(salinity) 7.398e-04 9 0.0 0.952

Signif. codes: 0 ‘***’ 0.001 ‘**’ 0.01 ‘*’ 0.05 ‘.’ 0.1 ‘ ’ 1

R-sq.(adj) = 0.298 Deviance explained = 32.4%

-REML = 928.32 Scale est. = 1 n = 325


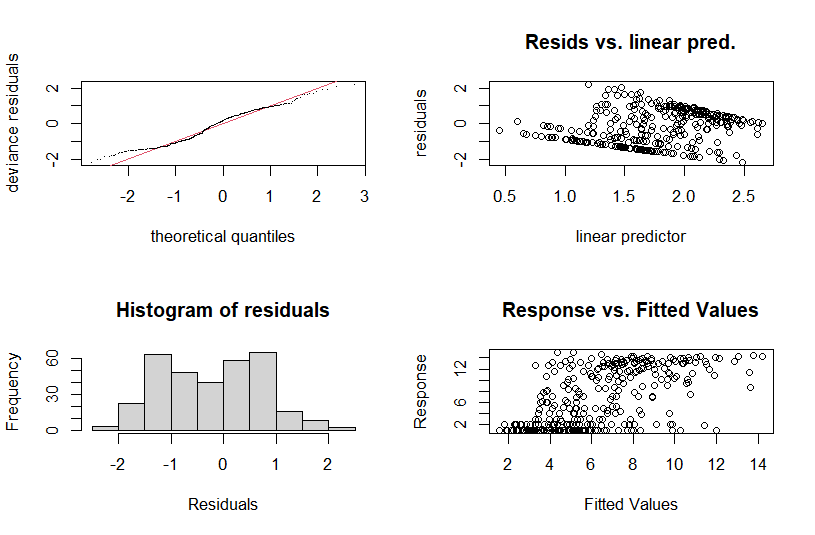


**Figure 35S.** The extracted plots of GAM model for salinity.

1. GAM model for dissolved oxygen

*gam.ES50.dissolved.oxygen <- gam(ES50 ~ s(Lat,Lon, bs="sos")+s(dissolved.oxygen), data = data, family = "nb", method = "REML", select = TRUE)*

*> gam.check(gam.ES50.dissolved.oxygen)*

Method: REML Optimizer: outer newton

full convergence after 6 iterations.

Gradient range [-0.0002070852,0.0002112791]

(score 928.311 & scale 1).

Hessian positive definite, eigenvalue range [7.048402e-08,64.66748].

Model rank = 59 / 59

Basis dimension (k) checking results. Low p-value (k-index<1) may

indicate that k is too low, especially if edf is close to k'.

k' edf k-index p-value

s(Lat,Lon) 49.00 28.48 0.97 0.65

s(dissolved.oxygen) 9.00 0.23 0.93 0.28

summary(gam.ES50.dissolved.oxygen)

Family: Negative Binomial(2.42)

Link function: log

Formula:

ES50 ~ s(Lat, Lon, bs = "sos") + s(dissolved.oxygen)

Parametric coefficients:

Estimate Std. Error z value Pr(>|z|)

(Intercept) 1.74955 0.04306 40.63 <2e-16 ***

Signif. codes: 0 ‘***’ 0.001 ‘**’ 0.01 ‘*’ 0.05 ‘.’ 0.1 ‘ ’ 1

Approximate significance of smooth terms:

edf Ref.df Chi.sq p-value

s(Lat,Lon) 28.4755 49 123.005 <2e-16 ***

s(dissolved.oxygen) 0.2299 9 0.257 0.263

Signif. codes: 0 ‘***’ 0.001 ‘**’ 0.01 ‘*’ 0.05 ‘.’ 0.1 ‘ ’ 1

R-sq.(adj) = 0.299 Deviance explained = 32.6%

-REML = 928.31 Scale est. = 1 n = 325


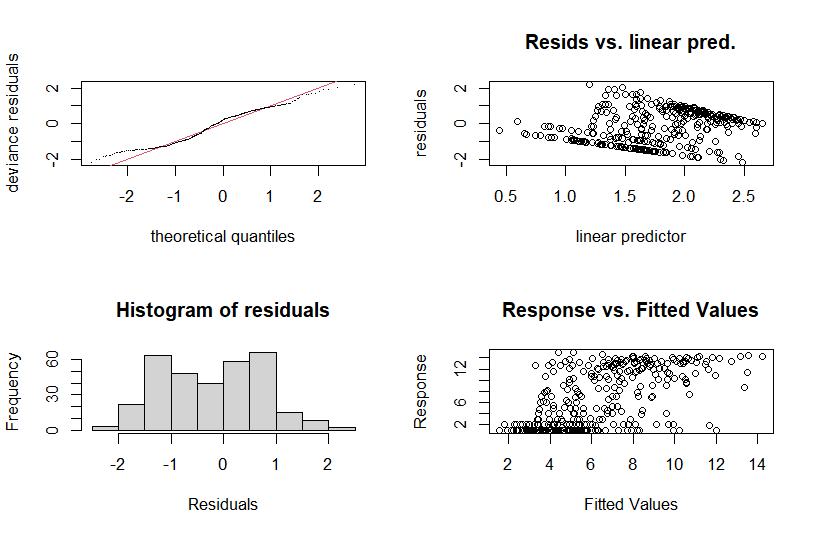


**Figure 36S.** The extracted plots of GAM model for dissolved oxygen.

1. GAM model for temperature

*gam.ES50.temp <- gam(ES50 ~ s(Lat,Lon, bs="sos")+s(temp), data = data, family = "nb", method = "REML", select = TRUE)*

*> gam.check(gam.ES50.temp)*

Method: REML Optimizer: outer newton

full convergence after 6 iterations.

Gradient range [-3.167521e-05,1.71605e-05]

(score 927.7981 & scale 1).

Hessian positive definite, eigenvalue range [3.167207e-05,64.5346].

Model rank = 59 / 59

Basis dimension (k) checking results. Low p-value (k-index<1) may

indicate that k is too low, especially if edf is close to k'.

k' edf k-index p-value

s(Lat,Lon) 49.00 27.42 0.98 0.69

s(temp) 9.00 1.57 1.00 0.72

*summary(gam.ES50.temp)*

Family: Negative Binomial(2.423)

Link function: log

Formula:

ES50 ~ s(Lat, Lon, bs = "sos") + s(temp)

Parametric coefficients:

Estimate Std. Error z value Pr(>|z|)

(Intercept) 1.74905 0.04305 40.63 <2e-16 ***

Signif. codes: 0 ‘***’ 0.001 ‘**’ 0.01 ‘*’ 0.05 ‘.’ 0.1 ‘ ’ 1

Approximate significance of smooth terms:

edf Ref.df Chi.sq p-value

s(Lat,Lon) 27.424 49 119.340 <2e-16 ***

s(temp) 1.572 9 3.816 0.0606 .

Signif. codes: 0 ‘***’ 0.001 ‘**’ 0.01 ‘*’ 0.05 ‘.’ 0.1 ‘ ’ 1

R-sq.(adj) = 0.294 Deviance explained = 32.8%

-REML = 927.8 Scale est. = 1 n = 325


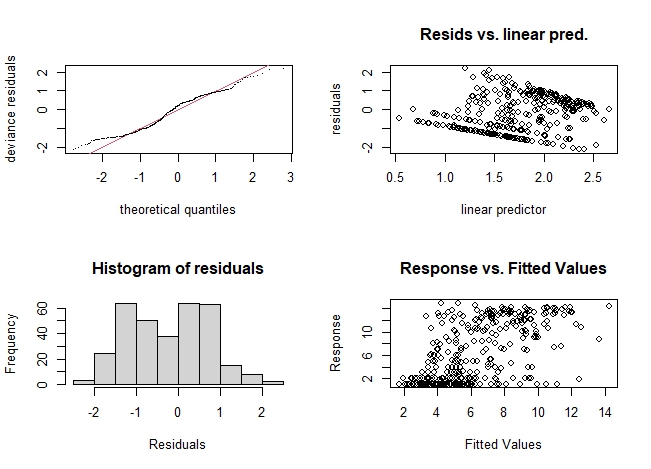


**Figure 37S.** The extracted plots of GAM model for temperature.

1. GAM model for the combination of environmental variables

*gam.ES50.env <- gam(ES50 ~ s(Lat,Lon, bs="sos")+ s (temp) + s(dissolved.oxygen) + s(chlorophyll) + s(current) + s(pH) + s(salinity) + s(depth)+ s(PO4) +s (current) , data = data, family = "nb", method= "REML", select = TRUE)*

*> gam.check(gam.ES50.env)*

Method: REML Optimizer: outer newton

full convergence after 9 iterations.

Gradient range [-9.555819e-05,0.0002586404]

(score 927.7956 & scale 1).

Hessian positive definite, eigenvalue range [2.261591e-06,64.43612].

Model rank = 122 / 122

Basis dimension (k) checking results. Low p-value (k-index<1) may

indicate that k is too low, especially if edf is close to k'.

k' edf k-index p-value

s(Lat,Lon) 4.90e+01 2.74e+01 0.98 0.68

s(temp) 9.00e+00 1.56e+00 1.00 0.81

s(dissolved.oxygen) 9.00e+00 5.90e-04 0.92 0.22

s(chlorophyll) 9.00e+00 1.88e-04 1.05 0.96

s(current) 9.00e+00 2.29e-04 0.94 0.38

s(pH) 9.00e+00 1.61e-01 0.97 0.64

s(salinity) 9.00e+00 2.01e-04 0.91 0.24

s(depth) 9.00e+00 1.96e-04 0.88 0.05 *

s(PO4) 9.00e+00 1.52e-04 0.91 0.20

Signif. codes: 0 ‘***’ 0.001 ‘**’ 0.01 ‘*’ 0.05 ‘.’ 0.1 ‘ ’ 1

summary(gam.ES50.env)

Family: Negative Binomial(2.424)

Link function: log

Formula:

ES50 ~ s(Lat, Lon, bs = "sos") + s(temp) + s(dissolved.oxygen) +

s(chlorophyll) + s(current) + s(pH) + s(salinity) + s(depth) +

s(PO4) + s(current)

Parametric coefficients:

Estimate Std. Error z value Pr(>|z|)

(Intercept) 1.74888 0.04304 40.64 <2e-16 ***

Signif. codes: 0 ‘***’ 0.001 ‘**’ 0.01 ‘*’ 0.05 ‘.’ 0.1 ‘ ’ 1

Approximate significance of smooth terms:

edf Ref.df Chi.sq p-value

s(Lat,Lon) 2.742e+01 49 118.298 <2e-16 ***

s(temp) 1.561e+00 9 3.729 0.0626 .

s(dissolved.oxygen) 5.896e-04 9 0.000 0.4722

s(chlorophyll) 1.880e-04 9 0.000 0.9301

s(current) 2.288e-04 9 0.000 0.7894

s(pH) 1.612e-01 9 0.159 0.3161

s(salinity) 2.013e-04 9 0.000 0.7016

s(depth) 1.958e-04 9 0.000 0.7651

s(PO4) 1.521e-04 9 0.000 0.9810

Signif. codes: 0 ‘***’ 0.001 ‘**’ 0.01 ‘*’ 0.05 ‘.’ 0.1 ‘ ’ 1

R-sq.(adj) = 0.295 Deviance explained = 32.8%

-REML = 927.8 Scale est. = 1 n = 325


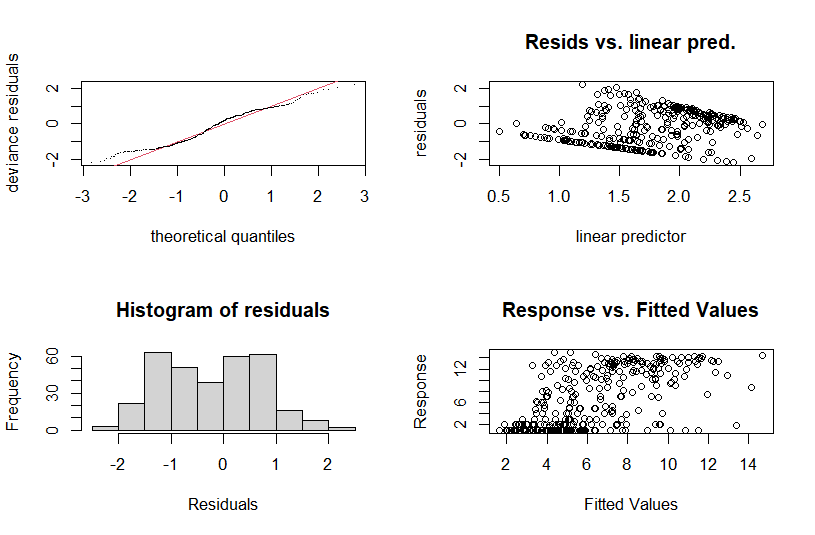


**Figure 38S.** The extracted plots of GAM model for the combination of environmental variables..

## Model selection for ES50 shallow water

In this part, the model selection for ES50 shallow water based on GAM analysis was presented.

Model selection for number of traces

*gam.ES50.models <- list(gam.ES50.intercept = gam.ES50.intercept,*

*gam.ES50.env=gam.ES50.env,*

*gam.ES50.latlon = gam.ES50.latlon,*

*gam.ES50.depth = gam.ES50.depth,*

*gam.ES50.pH = gam.ES50.pH,*

*gam.ES50.current = gam.ES50.current,*

*gam.ES50.PO4 = gam.ES50.PO4,*

*gam.ES50.chlorophyll= gam.ES50.chlorophyll,*

*gam.ES50.dissolved.oxygen = gam.ES50.dissolved.oxygen,*

*gam.ES50.salinity= gam.ES50.salinity,*

*gam.ES50.temp= gam.ES50.temp)*

*gam.ES50.aic.df <- data.frame(Model = names(gam.ES50.models),*

*AIC = sapply(gam.ES50.models, function(x) x$aic),*

*akaike.weights(sapply(gam.ES50.models, function(x) x$aic)))*

*gam.ES50.aic.df <- gam.ES50.aic.df[order(gam.ES50.aic.df$AIC),]*

*gam.ES50.aic.df$Cumulative.Weight <- cumsum(gam.ES50.aic.df$weights)*

*kable(gam.ES50.aic.df, row.names = FALSE*

| Model | AIC | deltaAIC | rel.LL | weights | Cumulative.Weight |
| --- | --- | --- | --- | --- | --- |
| gam.ES50.temp | 1817.7284 | 0.0000 | 1.0000 | 0.1242 | 0.1242 |
| gam.ES50.env | 1817.7329 | 0.0045 | 0.9978 | 0.1239 | 0.2482 |
| gam.ES50.dissolved.oxygen | 1817.9709 | 0.2425 | 0.8858 | 0.1100 | 0.3582 |
| gam.ES50.latlon | 1818.3232 | 0.5948 | 0.7428 | 0.0923 | 0.4505 |
| gam.ES50.PO4 | 1818.3234 | 0.5950 | 0.7427 | 0.0923 | 0.5427 |
| gam.ES50.current | 1818.3245 | 0.5961 | 0.7423 | 0.0922 | 0.6349 |
| gam.ES50.salinity | 1818.3245 | 0.5961 | 0.7423 | 0.0922 | 0.7271 |
| gam.ES50.chlorophyll | 1818.3246 | 0.5962 | 0.7422 | 0.0922 | 0.8193 |
| gam.ES50.depth | 1818.3320 | 0.6036 | 0.7395 | 0.0919 | 0.9112 |
| gam.ES50.pH | 1818.3989 | 0.6705 | 0.7152 | 0.0888 | 1.0000 |
| gam.ES50.intercept | 1891.4140 | 73.6856 | 0.0000 | 0.0000 | 1.0000 |

The results show the most fitted model of ES50 could be found with temperature in shallow waters. The other parameters including env, dissolved oxygen, latlon, PO4, current, salinity, chlorophyll, depth, and pH with less than 2 deltaAIC depicted the distribution pattern of amphipods in shallow waters.

## Plots of GMAs analysis of ES50 in the shallow water

The extracted plots of the relation between environmental variables and the extracted models were presented for GAMs analysis of ES50 in the shallow waters.


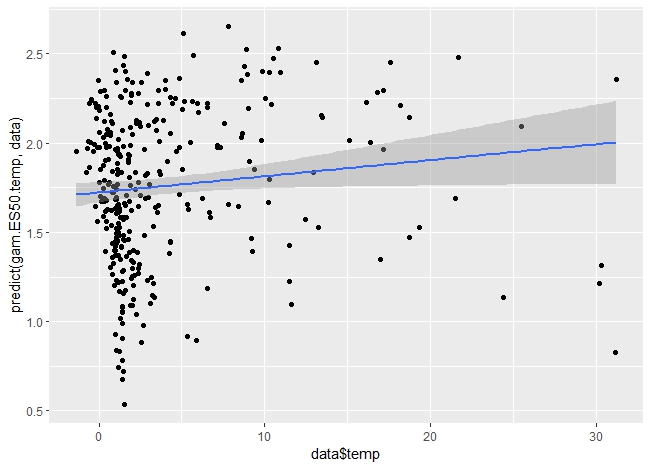


**Figure 39S.** qplot(data$temp, predict(gam.ES50.temp, data)) + geom_smooth(method = "glm")


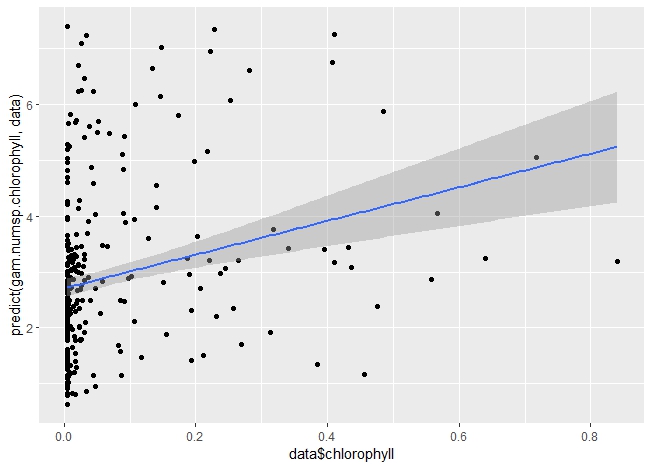


**Figure 40S.** qplot(data$chlorophyll, predict(gam.ES50.chlorophyll, data)) + geom_smooth(method = "glm")


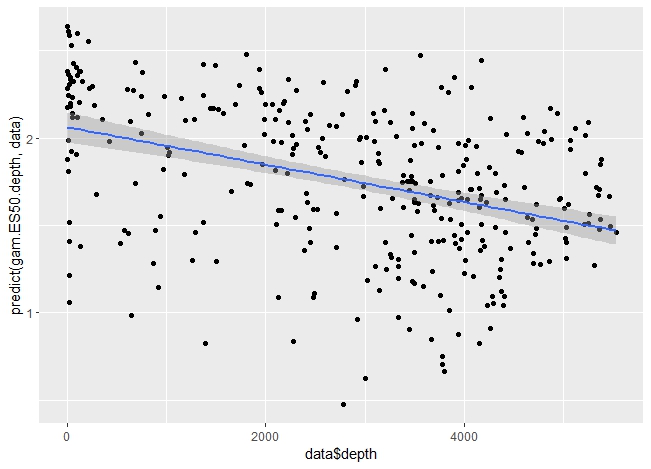


**Figure 41S.** qplot(data$depth, predict(gam.ES50.depth, data)) + geom_smooth(method = "glm")


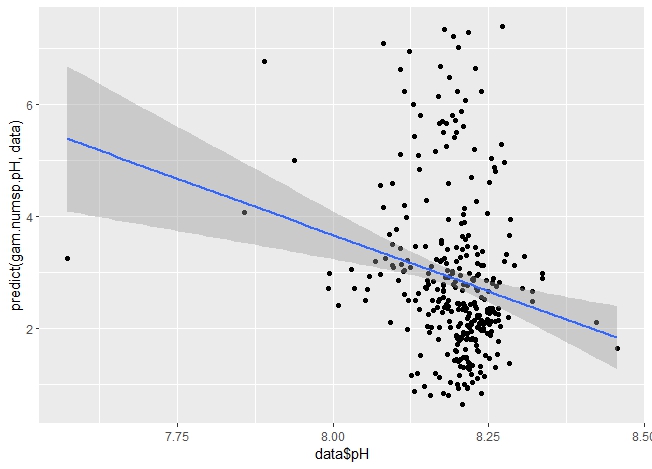


**Figure 42S.** qplot(data$pH, predict(gam.ES50.pH, data)) + geom_smooth(method = "glm")


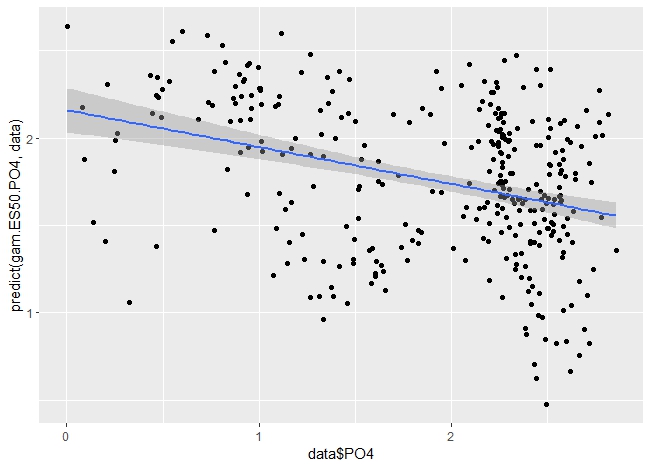


**Figure 43S.** qplot(data$PO4, predict(gam.ES50.PO4, data)) + geom_smooth(method


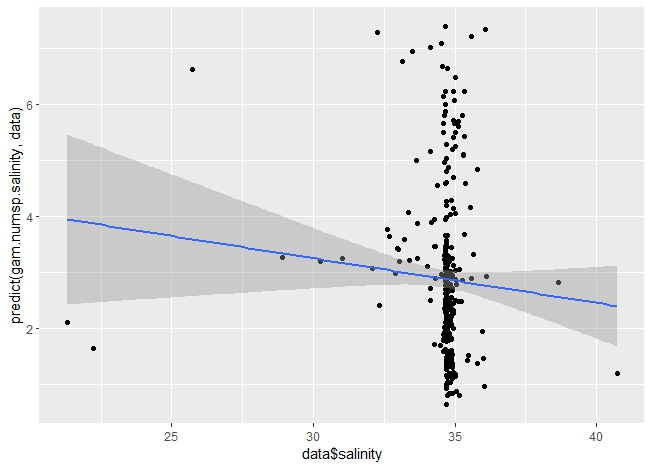


**Figure 44S.** qplot(data$salinity, predict(gam.ES50.salinity, data)) + geom_smooth(method = "glm")"glm")


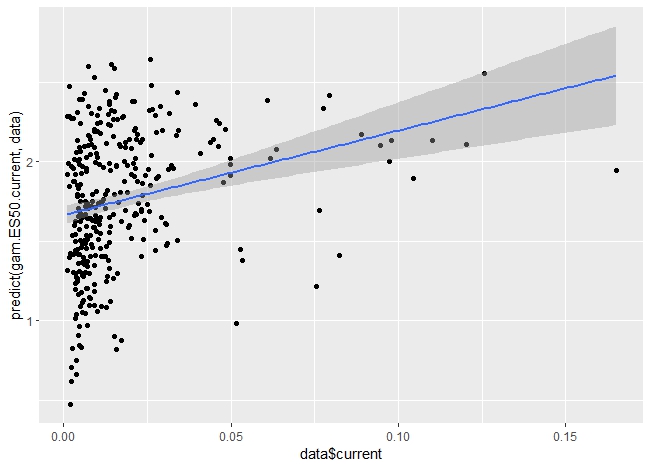


**Figure 45S.** qplot(data$current, predict(gam.ES50.current, data)) + geom_smooth(method = "glm")


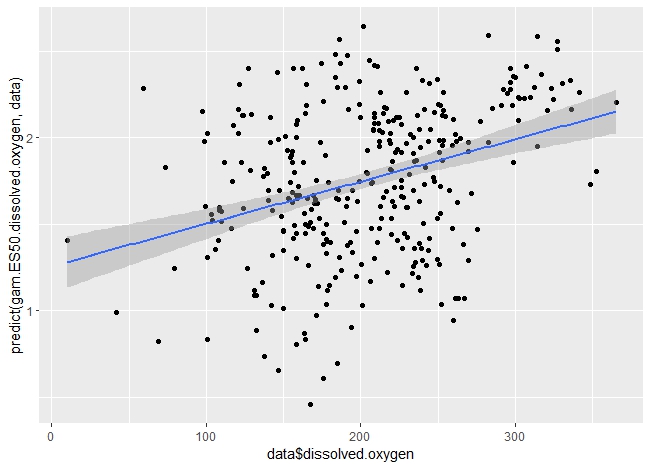


**Figure 46S.** qplot(data$dissolved.oxygen, predict(gam.ES50.dissolved.oxygen, data)) + geom_smooth(method = "glm)

# GAMs for the number of species, deep sea

The correlation between environmental variables, the script, plots and figures of GAMs analysis for the number of species in the deep sea were presented in this part.


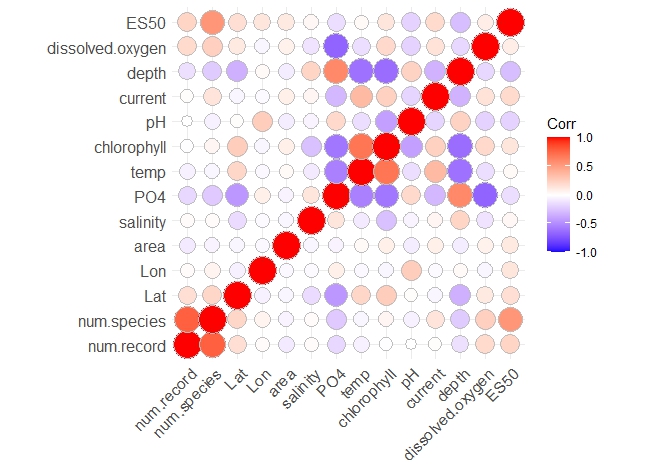


**Figure 47S.** The correlation between environmental variables and ES50, the number of records, and the number of shallow benthic species.

## The script and extracted figures of GAM models for the number of species in deep sea.

1. GAM model for intercept

*gam.numsp.intercept <- gam(num.species ~ 1, data = data, family = "nb", method = "REML", select = TRUE)*

*> gam.check(gam.numsp.intercept)*

Method: REML Optimizer: outer newton

full convergence after 4 iterations.

Gradient range [2.072174e-07,2.072174e-07]

(score 1506.42 & scale 1).

Hessian positive definite, eigenvalue range [249.2453,249.2453].

Model rank = 1 / 1

> summary(gam.numsp.intercept)

Family: Negative Binomial(0.431)

Link function: log

Formula:

num.species ~ 1

Parametric coefficients:

Estimate Std. Error z value Pr(>|z|)

(Intercept) 3.48775 0.08106 43.02 <2e-16 ***

Signif. codes: 0 ‘***’ 0.001 ‘**’ 0.01 ‘*’ 0.05 ‘.’ 0.1 ‘ ’ 1

R-sq.(adj) = 0 Deviance explained = 1.07e-09%

-REML = 1506.4 Scale est. = 1 n = 358


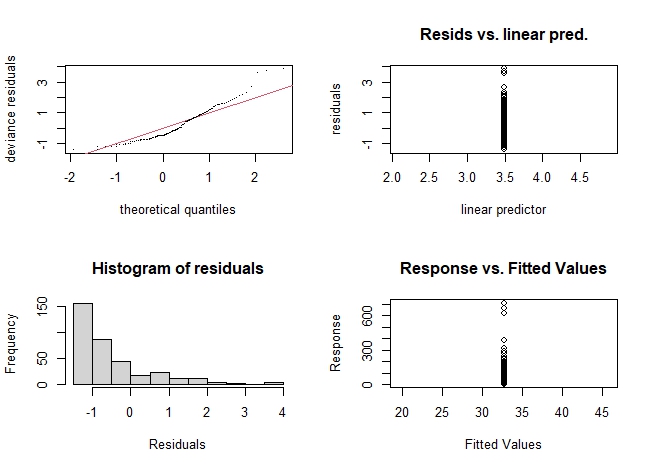


**Figure 48S.** The extracted plots of GAM model for intercept

1. GAM model for the number of records

*gam.numsp.numrec <- gam(num.species ~ s(num.record), data = data, family = "nb", method = "REML", select = TRUE)*

*> gam.check(gam.numsp.numrec)*

Method: REML Optimizer: outer newton

full convergence after 15 iterations.

Gradient range [-9.93025e-06,0.0001580771]

(score 1185.232 & scale 1).

Hessian positive definite, eigenvalue range [0.4965799,92.9349].

Model rank = 10 / 10

Basis dimension (k) checking results. Low p-value (k-index<1) may

indicate that k is too low, especially if edf is close to k'.

k' edf k-index p-value

s(num.record) 9.00 8.82 0.42 <2e-16 ***

Signif. codes: 0 ‘***’ 0.001 ‘**’ 0.01 ‘*’ 0.05 ‘.’ 0.1 ‘ ’ 1

*summary(gam.numsp.numrec)*

Family: Negative Binomial(2.896)

Link function: log

Formula:

num.species ~ s(num.record)

Parametric coefficients:

Estimate Std. Error z value Pr(>|z|)

(Intercept) 2.30920 0.03754 61.52 <2e-16 ***

Signif. codes: 0 ‘***’ 0.001 ‘**’ 0.01 ‘*’ 0.05 ‘.’ 0.1 ‘ ’ 1

Approximate significance of smooth terms:

edf Ref.df Chi.sq p-value

s(num.record) 8.817 9 1619 <2e-16 ***

Signif. codes: 0 ‘***’ 0.001 ‘**’ 0.01 ‘*’ 0.05 ‘.’ 0.1 ‘ ’ 1

R-sq.(adj) = 0.845 Deviance explained = 86.1%

-REML = 1185.2 Scale est. = 1 n = 358


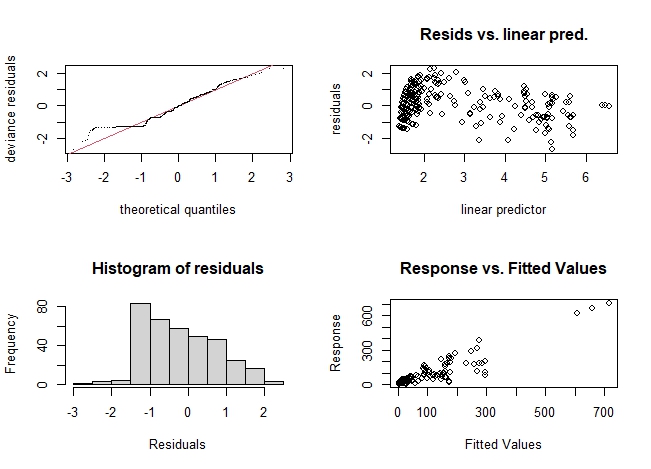


**Figure 49S.** The extracted plots of GAM model for the number of species.

1. GAM model for spatial autocorrelation

*gam.numsp.latlon <- gam(num.species ~ s(Lat,Lon, bs="sos")+ s(num.record), data = data, family = "nb", method = "REML", select = TRUE)*

*> gam.check(gam.numsp.latlon)*

Method: REML Optimizer: outer newton

full convergence after 16 iterations.

Gradient range [-1.271869e-06,1.299328e-05]

(score 1182.22 & scale 1).

Hessian positive definite, eigenvalue range [0.4955471,78.47354].

Model rank = 59 / 59

Basis dimension (k) checking results. Low p-value (k-index<1) may

indicate that k is too low, especially if edf is close to k'.

k' edf k-index p-value

s(Lat,Lon) 49.0 21.0 0.92 0.18

s(num.record) 9.0 8.8 0.45 <2e-16 ***

Signif. codes: 0 ‘***’ 0.001 ‘**’ 0.01 ‘*’ 0.05 ‘.’ 0.1 ‘ ’ 1

*> summary(gam.numsp.latlon)*

Family: Negative Binomial(3.435)

Link function: log

Formula:

num.species ~ s(Lat, Lon, bs = "sos") + s(num.record)

Parametric coefficients:

Estimate Std. Error z value Pr(>|z|)

(Intercept) 2.27189 0.03591 63.27 <2e-16 ***

Signif. codes: 0 ‘***’ 0.001 ‘**’ 0.01 ‘*’ 0.05 ‘.’ 0.1 ‘ ’ 1

Approximate significance of smooth terms:

edf Ref.df Chi.sq p-value

s(Lat,Lon) 20.992 49 51.86 1.24e-05 ***

s(num.record) 8.795 9 1073.08 < 2e-16 ***

Signif. codes: 0 ‘***’ 0.001 ‘**’ 0.01 ‘*’ 0.05 ‘.’ 0.1 ‘ ’ 1

R-sq.(adj) = 0.85 Deviance explained = 89%

-REML = 1182.2 Scale est. = 1 n = 358


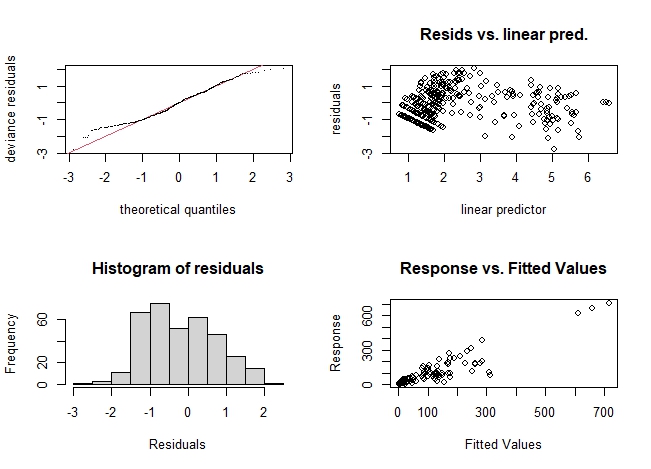


**Figure 50S.** The extracted plots of GAM model for spatial autocorrelation.

1. GAM model for depth

*gam.numsp.depth <- gam(num.species ~ s(Lat,Lon, bs="sos")+ s(num.record)+s(depth), data = data, family = "nb", method = "REML", select = TRUE)*

*> gam.check(gam.numsp.depth)*

Method: REML Optimizer: outer newton

full convergence after 12 iterations.

Gradient range [-1.031691e-05,1.568733e-05]

(score 1177.72 & scale 1).

Hessian positive definite, eigenvalue range [1.031671e-05,77.70622].

Model rank = 68 / 68

Basis dimension (k) checking results. Low p-value (k-index<1) may

indicate that k is too low, especially if edf is close to k'.

k' edf k-index p-value

s(Lat,Lon) 49.00 17.68 0.91 0.11

s(num.record) 9.00 8.79 0.44 <2e-16 ***

s(depth) 9.00 1.84 0.93 0.26

Signif. codes: 0 ‘***’ 0.001 ‘**’ 0.01 ‘*’ 0.05 ‘.’ 0.1 ‘ ’ 1

*> summary(gam.numsp.depth)*

Family: Negative Binomial(3.512)

Link function: log

Formula:

num.species ~ s(Lat, Lon, bs = "sos") + s(num.record) + s(depth)

Parametric coefficients:

Estimate Std. Error z value Pr(>|z|)

(Intercept) 2.27095 0.03565 63.71 <2e-16 ***

Signif. codes: 0 ‘***’ 0.001 ‘**’ 0.01 ‘*’ 0.05 ‘.’ 0.1 ‘ ’ 1

Approximate significance of smooth terms:

edf Ref.df Chi.sq p-value

s(Lat,Lon) 17.685 49 39.93 0.000189 ***

s(num.record) 8.792 9 1113.95 < 2e-16 ***

s(depth) 1.836 9 12.65 0.000276 ***

Signif. codes: 0 ‘***’ 0.001 ‘**’ 0.01 ‘*’ 0.05 ‘.’ 0.1 ‘ ’ 1

R-sq.(adj) = 0.843 Deviance explained = 89.1%

-REML = 1177.7 Scale est. = 1 n = 358


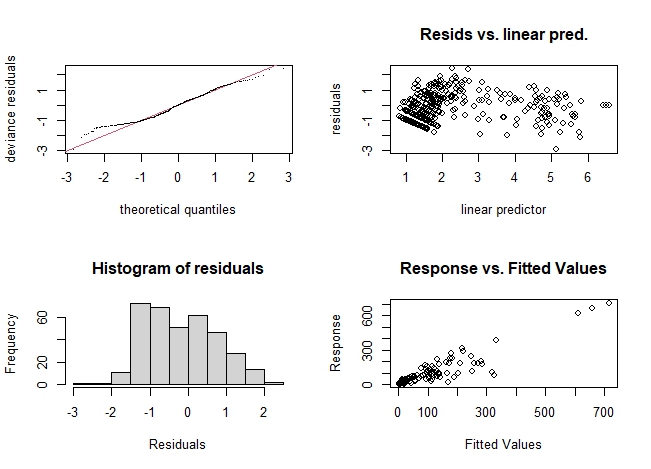


**Figure 51S.** The extracted plots of GAM model for dpth

5) GAM model for chlorphyll

*> gam.numsp.chlorophyll <- gam(num.species ~ s(Lat,Lon, bs="sos")+ s(num.record)+s(chlorophyll), data = data, family = "nb", method = "REML", select = TRUE)*

*> gam.check(gam.numsp.chlorophyll)*

Method: REML Optimizer: outer newton

full convergence after 15 iterations.

Gradient range [-0.0001114954,4.013088e-05]

(score 1185.232 & scale 1).

Hessian positive definite, eigenvalue range [2.529211e-05,92.93221].

Model rank = 68 / 68

Basis dimension (k) checking results. Low p-value (k-index<1) may

indicate that k is too low, especially if edf is close to k'.

k' edf k-index p-value

s(Lat,Lon) 49.00000 0.00370 0.78 <2e-16 ***

s(num.record) 9.00000 8.81654 0.42 <2e-16 ***

s(chlorophyll) 9.00000 0.00118 0.98 0.68

Signif. codes: 0 ‘***’ 0.001 ‘**’ 0.01 ‘*’ 0.05 ‘.’ 0.1 ‘ ’ 1

*> summary(gam.numsp.chlorophyll)*

Family: Negative Binomial(2.896)

Link function: log

Formula:

num.species ~ s(Lat, Lon, bs = "sos") + s(num.record) + s(chlorophyll)

Parametric coefficients:

Estimate Std. Error z value Pr(>|z|)

(Intercept) 2.30919 0.03754 61.52 <2e-16 ***

Signif. codes: 0 ‘***’ 0.001 ‘**’ 0.01 ‘*’ 0.05 ‘.’ 0.1 ‘ ’ 1

Approximate significance of smooth terms:

edf Ref.df Chi.sq p-value

s(Lat,Lon) 0.003700 49 0.004 0.386

s(num.record) 8.816536 9 1619.286 <2e-16 ***

s(chlorophyll) 0.001183 9 0.001 0.304

Signif. codes: 0 ‘***’ 0.001 ‘**’ 0.01 ‘*’ 0.05 ‘.’ 0.1 ‘ ’ 1

R-sq.(adj) = 0.845 Deviance explained = 86.1%

-REML = 1185.2 Scale est. = 1 n = 358


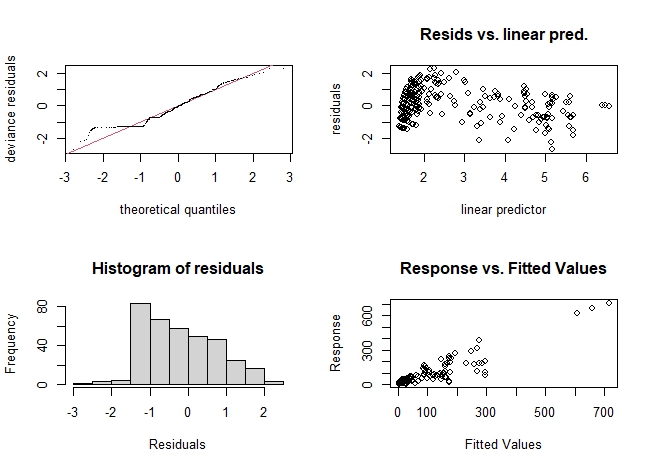


**Figure 52S.** The extracted plots of GAM model for dpth

1. GAM model for current

*gam.numsp.current <- gam(num.species ~ s(Lat,Lon, bs="sos")+ s(num.record)+s(current), data = data, family = "nb", method = "REML", select = TRUE)*

*> gam.check(gam.numsp.current)*

Method: REML Optimizer: outer newton

full convergence after 15 iterations.

Gradient range [-0.000160323,0.0003112152]

(score 1182.122 & scale 1).

Hessian positive definite, eigenvalue range [3.291416e-05,77.91067].

Model rank = 68 / 68

Basis dimension (k) checking results. Low p-value (k-index<1) may

indicate that k is too low, especially if edf is close to k'.

k' edf k-index p-value

s(Lat,Lon) 49.000 20.997 0.92 0.12

s(num.record) 9.000 8.794 0.44 <2e-16 ***

s(current) 9.000 0.691 0.86 0.01 **

Signif. codes: 0 ‘***’ 0.001 ‘**’ 0.01 ‘*’ 0.05 ‘.’ 0.1 ‘ ’ 1

*> summary(gam.numsp.current)*

Family: Negative Binomial(3.45)

Link function: log

Formula:

num.species ~ s(Lat, Lon, bs = "sos") + s(num.record) + s(current)

Parametric coefficients:

Estimate Std. Error z value Pr(>|z|)

(Intercept) 2.27114 0.03586 63.33 <2e-16 ***

Signif. codes: 0 ‘***’ 0.001 ‘**’ 0.01 ‘*’ 0.05 ‘.’ 0.1 ‘ ’ 1

Approximate significance of smooth terms:

edf Ref.df Chi.sq p-value

s(Lat,Lon) 20.997 49 51.733 1.22e-05 ***

s(num.record) 8.794 9 1060.138 < 2e-16 ***

s(current) 0.691 9 1.013 0.201

Signif. codes: 0 ‘***’ 0.001 ‘**’ 0.01 ‘*’ 0.05 ‘.’ 0.1 ‘ ’ 1

R-sq.(adj) = 0.854 Deviance explained = 89%

-REML = 1182.1 Scale est. = 1 n = 358


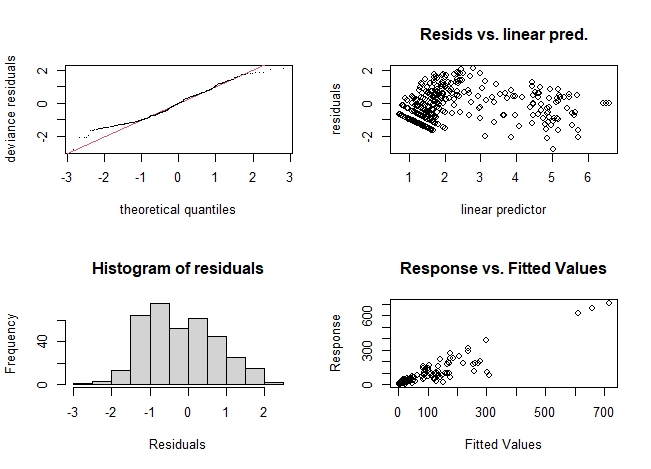


**Figure 53S.** The extracted plots of GAM model for current

7) GAM model for pH

*> gam.numsp.pH <- gam(num.species ~ s(Lat,Lon, bs="sos")+ s(num.record)+s(pH), data = data, family = "nb", method = "REML", select = TRUE)*

*> gam.check(gam.numsp.pH)*

Method: REML Optimizer: outer newton

full convergence after 18 iterations.

Gradient range [-9.98769e-05,0.0001469718]

(score 1181.522 & scale 1).

Hessian positive definite, eigenvalue range [9.986257e-05,78.38483].

Model rank = 68 / 68

Basis dimension (k) checking results. Low p-value (k-index<1) may

indicate that k is too low, especially if edf is close to k'.

k' edf k-index p-value

s(Lat,Lon) 49.000 20.350 0.91 0.12

s(num.record) 9.000 8.796 0.45 <2e-16 ***

s(pH) 9.000 0.725 0.90 0.09 .

Signif. codes: 0 ‘***’ 0.001 ‘**’ 0.01 ‘*’ 0.05 ‘.’ 0.1 ‘ ’ 1

*> summary(gam.numsp.pH)*

Family: Negative Binomial(3.452)

Link function: log

Formula:

num.species ~ s(Lat, Lon, bs = "sos") + s(num.record) + s(pH)

Parametric coefficients:

Estimate Std. Error z value Pr(>|z|)

(Intercept) 2.27138 0.03585 63.36 <2e-16 ***

Signif. codes: 0 ‘***’ 0.001 ‘**’ 0.01 ‘*’ 0.05 ‘.’ 0.1 ‘ ’ 1

Approximate significance of smooth terms:

edf Ref.df Chi.sq p-value

s(Lat,Lon) 20.3499 49 48.486 3.27e-05 ***

s(num.record) 8.7964 9 1087.346 < 2e-16 ***

s(pH) 0.7245 9 2.592 0.0521 .

Signif. codes: 0 ‘***’ 0.001 ‘**’ 0.01 ‘*’ 0.05 ‘.’ 0.1 ‘ ’ 1

R-sq.(adj) = 0.851 Deviance explained = 89%

-REML = 1181.5 Scale est. = 1 n = 358


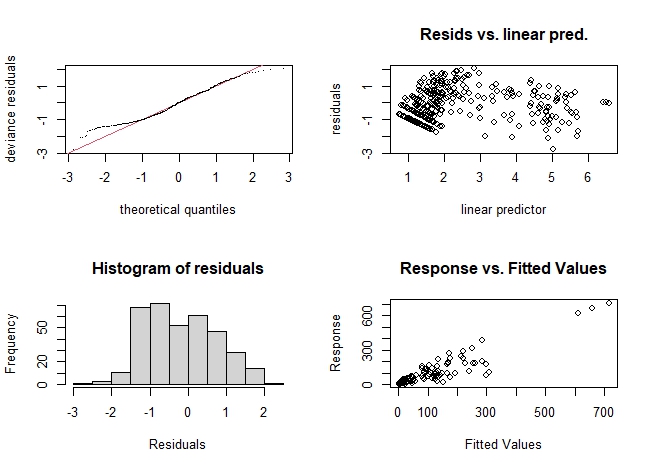


**Figure 54S.** The extracted plots of GAM model for pH

8) GAM model for PO4

*> gam.numsp.PO4 <- gam(num.species ~ s(Lat,Lon, bs="sos")+ s(num.record)+s(PO4), data = data, family = "nb", method = "REML", select = TRUE)*

*> gam.check(gam.numsp.PO4)*

Method: REML Optimizer: outer newton

full convergence after 16 iterations.

Gradient range [-1.879324e-05,9.662876e-05]

(score 1181.767 & scale 1).

Hessian positive definite, eigenvalue range [1.87904e-05,78.39669].

Model rank = 68 / 68

Basis dimension (k) checking results. Low p-value (k-index<1) may

indicate that k is too low, especially if edf is close to k'.

k' edf k-index p-value

s(Lat,Lon) 49.000 20.083 0.91 0.13

s(num.record) 9.000 8.796 0.45 <2e-16 ***

s(PO4) 9.000 0.925 0.93 0.22

Signif. codes: 0 ‘***’ 0.001 ‘**’ 0.01 ‘*’ 0.05 ‘.’ 0.1 ‘ ’ 1

*> summary(gam.numsp.PO4)*

Family: Negative Binomial(3.44)

Link function: log

Formula:num.species ~ s(Lat, Lon, bs = "sos") + s(num.record) + s(PO4)

Parametric coefficients:

Estimate Std. Error z value Pr(>|z|)

(Intercept) 2.27207 0.03588 63.33 <2e-16 ***

Signif. codes: 0 ‘***’ 0.001 ‘**’ 0.01 ‘*’ 0.05 ‘.’ 0.1 ‘ ’ 1

Approximate significance of smooth terms:

edf Ref.df Chi.sq p-value

s(Lat,Lon) 20.0829 49 48.866 1.58e-05 ***

s(num.record) 8.7958 9 1086.571 < 2e-16 ***

s(PO4) 0.9253 9 2.184 0.0748 .

Signif. codes: 0 ‘***’ 0.001 ‘**’ 0.01 ‘*’ 0.05 ‘.’ 0.1 ‘ ’ 1

R-sq.(adj) = 0.845 Deviance explained = 89%

-REML = 1181.8 Scale est. = 1 n = 358


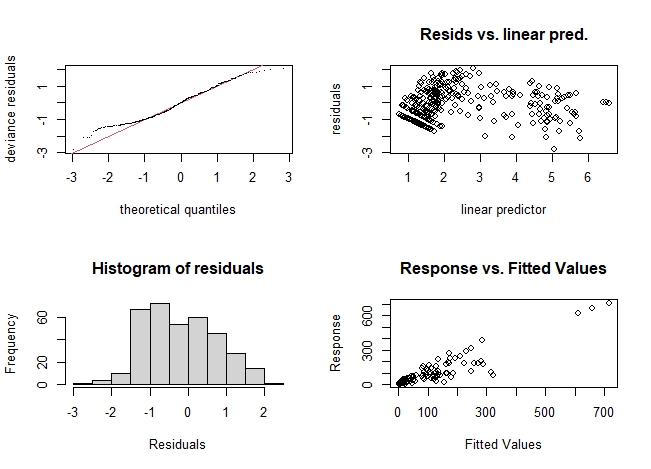


**Figure 55S.** The extracted plots of GAM model for PO4

9) GAM model for salinity

*> gam.numsp.salinity <- gam(num.species ~ s(Lat,Lon, bs="sos")+ s(num.record)+s(salinity), data = data, family = "nb", method = "REML", select = TRUE)*

*> gam.check(gam.numsp.salinity)*

Method: REML Optimizer: outer newton

full convergence after 21 iterations.

Gradient range [-0.000387197,8.325803e-05]

(score 1181.706 & scale 1).

Hessian positive definite, eigenvalue range [0.0002608122,78.33317].

Model rank = 68 / 68

Basis dimension (k) checking results. Low p-value (k-index<1) may

indicate that k is too low, especially if edf is close to k'.

k' edf k-index p-value

s(Lat,Lon) 49.000 20.427 0.91 0.13

s(num.record) 9.000 8.795 0.45 <2e-16 ***

s(salinity) 9.000 0.677 0.94 0.28

Signif. codes: 0 ‘***’ 0.001 ‘**’ 0.01 ‘*’ 0.05 ‘.’ 0.1 ‘ ’ 1

*> summary(gam.numsp.salinity)*

Family: Negative Binomial(3.45)

Link function: log

Formula:

num.species ~ s(Lat, Lon, bs = "sos") + s(num.record) + s(salinity)

Parametric coefficients:

Estimate Std. Error z value Pr(>|z|)

(Intercept) 2.27153 0.03586 63.35 <2e-16 ***

---

Signif. codes: 0 ‘***’ 0.001 ‘**’ 0.01 ‘*’ 0.05 ‘.’ 0.1 ‘ ’ 1

Approximate significance of smooth terms:

edf Ref.df Chi.sq p-value

s(Lat,Lon) 20.4275 49 49.158 2.56e-05 ***

s(num.record) 8.7953 9 1085.234 < 2e-16 ***

s(salinity) 0.6774 9 2.083 0.0696 .

---

Signif. codes: 0 ‘***’ 0.001 ‘**’ 0.01 ‘*’ 0.05 ‘.’ 0.1 ‘ ’ 1

R-sq.(adj) = 0.85 Deviance explained = 89%

-REML = 1181.7 Scale est. = 1 n = 358


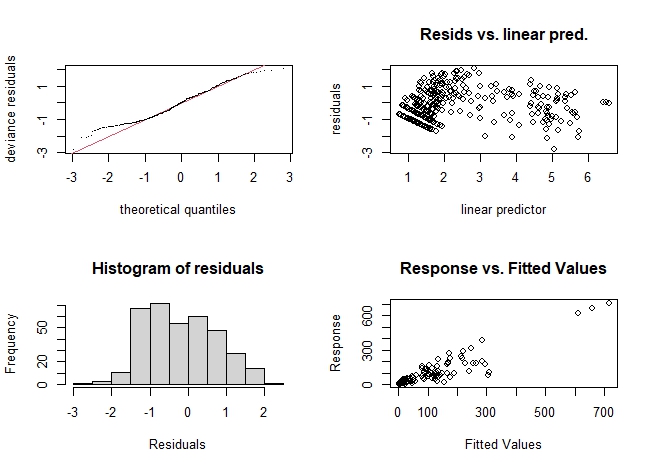


**Figure 56S.** The extracted plots of GAM model for salinity

10) GAMmodel for dissolved oxygen

*> gam.numsp.dissolved.oxygen <- gam(num.species ~ s(Lat,Lon, bs="sos")+ s(num.record)+s(dissolved.oxygen), data = data, family = "nb", method = "REML", select = TRUE)*

*> gam.check(gam.numsp.dissolved.oxygen)*

Method: REML Optimizer: outer newton

full convergence after 12 iterations.

Gradient range [-3.625614e-05,0.0002695791]

(score 1183.371 & scale 1).

eigenvalue range [-6.810567e-05,90.9363].

Model rank = 68 / 68

Basis dimension (k) checking results. Low p-value (k-index<1) may

indicate that k is too low, especially if edf is close to k'.

k' edf k-index p-value

s(Lat,Lon) 4.90e+01 4.55e-04 0.79 <2e-16 ***

s(num.record) 9.00e+00 8.82e+00 0.43 <2e-16 ***

s(dissolved.oxygen) 9.00e+00 1.56e+00 0.91 0.17

Signif. codes: 0 ‘***’ 0.001 ‘**’ 0.01 ‘*’ 0.05 ‘.’ 0.1 ‘ ’ 1

*> summary(gam.numsp.dissolved.oxygen)*

Family: Negative Binomial(2.973)

Link function: log

Formula:

num.species ~ s(Lat, Lon, bs = "sos") + s(num.record) + s(dissolved.oxygen)

Parametric coefficients:

Estimate Std. Error z value Pr(>|z|)

(Intercept) 2.30454 0.03725 61.87 <2e-16 ***

Signif. codes: 0 ‘***’ 0.001 ‘**’ 0.01 ‘*’ 0.05 ‘.’ 0.1 ‘ ’ 1

Approximate significance of smooth terms:

edf Ref.df Chi.sq p-value

s(Lat,Lon) 0.0004549 49 0.000 0.4650

s(num.record) 8.8163952 9 1549.259 <2e-16 ***

s(dissolved.oxygen) 1.5557545 9 6.086 0.0155 *

Signif. codes: 0 ‘***’ 0.001 ‘**’ 0.01 ‘*’ 0.05 ‘.’ 0.1 ‘ ’ 1

R-sq.(adj) = 0.848 Deviance explained = 86.5%

-REML = 1183.4 Scale est. = 1 n = 358


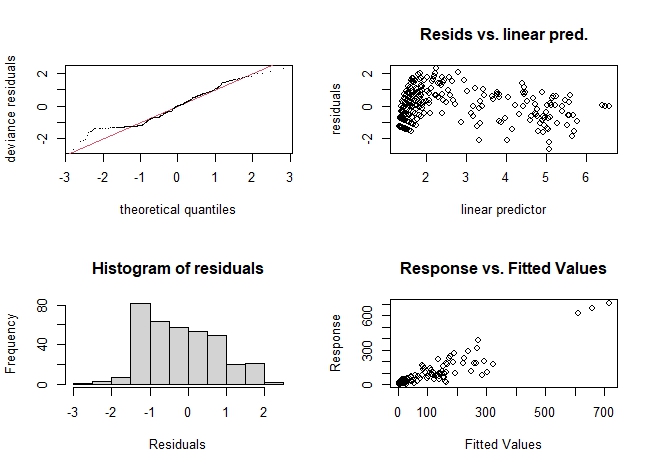


**Figure 57S.** The extracted plots of GAM model for dissolved oxygen

11) GAM model for temperature

*> gam.numsp.temp <- gam(num.species ~ s(Lat,Lon, bs="sos")+ s(num.record)+s(temp), data = data, family = "nb", method = "REML", select = TRUE)*

*> gam.check(gam.numsp.temp)*

Method: REML Optimizer: outer newton

full convergence after 19 iterations.

Gradient range [-8.257339e-05,0.0001520912]

(score 1180.84 & scale 1).

Hessian positive definite, eigenvalue range [8.254961e-05,77.95696].

Model rank = 68 / 68

Basis dimension (k) checking results. Low p-value (k-index<1) may

indicate that k is too low, especially if edf is close to k'.

k' edf k-index p-value

s(Lat,Lon) 49.00 18.77 0.92 0.14

s(num.record) 9.00 8.79 0.45 <2e-16 ***

s(temp) 9.00 1.98 0.95 0.46

Signif. codes: 0 ‘***’ 0.001 ‘**’ 0.01 ‘*’ 0.05 ‘.’ 0.1 ‘ ’ 1

*> summary(gam.numsp.temp)*

Family: Negative Binomial(3.46)

Link function: log

Formula:

num.species ~ s(Lat, Lon, bs = "sos") + s(num.record) + s(temp)

Parametric coefficients:

Estimate Std. Error z value Pr(>|z|)

(Intercept) 2.27160 0.03582 63.42 <2e-16 ***

Signif. codes: 0 ‘***’ 0.001 ‘**’ 0.01 ‘*’ 0.05 ‘.’ 0.1 ‘ ’ 1

Approximate significance of smooth terms:

edf Ref.df Chi.sq p-value

s(Lat,Lon) 18.766 49 45.596 3.46e-05 ***

s(num.record) 8.793 9 1094.994 < 2e-16 ***

s(temp) 1.981 9 6.906 0.0149 *

Signif. codes: 0 ‘***’ 0.001 ‘**’ 0.01 ‘*’ 0.05 ‘.’ 0.1 ‘ ’ 1

R-sq.(adj) = 0.853 Deviance explained = 89%

-REML = 1180.8 Scale est. = 1 n = 358


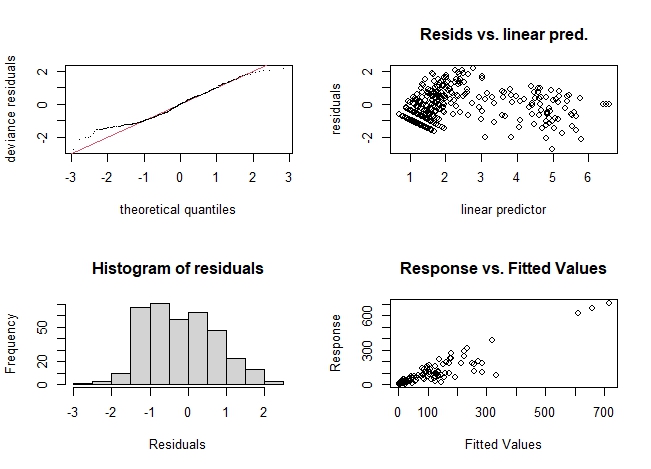


**Figure 58S.** The extracted plots of GAM model for temperature

1. GAM model for the combination of environmental variables

*gam.numsp.env <- gam(num.species ~ s(Lat,Lon, bs="sos")+ s(num.record)+ s (temp) + s(dissolved.oxygen) + s(chlorophyll) + s(current) + s(pH) + s(salinity) + s(depth)+ s(PO4) +s (current) , data = data, family = "nb", method= "REML", select = TRUE)*

*> gam.check(gam.numsp.env)*

Method: REML Optimizer: outer newton

full convergence after 15 iterations.

Gradient range [-0.0002806055,0.0002709344]

(score 1174.929 & scale 1).

Hessian positive definite, eigenvalue range [1.423063e-06,77.17695].

Model rank = 131 / 131

Basis dimension (k) checking results. Low p-value (k-index<1) may

indicate that k is too low, especially if edf is close to k'.

k' edf k-index p-value

s(Lat,Lon) 4.90e+01 1.37e+01 0.89 0.030 *

s(num.record) 9.00e+00 8.80e+00 0.45 <2e-16 ***

s(temp) 9.00e+00 2.16e+00 0.97 0.655

s(dissolved.oxygen) 9.00e+00 3.64e-04 0.94 0.335

s(chlorophyll) 9.00e+00 1.75e+00 1.04 0.960

s(current) 9.00e+00 2.85e-01 0.86 0.005 **

s(pH) 9.00e+00 5.56e-05 0.90 0.085 .

s(salinity) 9.00e+00 2.00e-04 0.96 0.555

s(depth) 9.00e+00 1.86e+00 0.94 0.345

s(PO4) 9.00e+00 1.91e-04 0.94 0.305

Signif. codes: 0 ‘***’ 0.001 ‘**’ 0.01 ‘*’ 0.05 ‘.’ 0.1 ‘ ’ 1

*> summary(gam.numsp.env)*

Family: Negative Binomial(3.591)

Link function: log

Formula:

num.species ~ s(Lat, Lon, bs = "sos") + s(num.record) + s(temp) +

s(dissolved.oxygen) + s(chlorophyll) + s(current) + s(pH) +

s(salinity) + s(depth) + s(PO4) + s(current)

Parametric coefficients:

Estimate Std. Error z value Pr(>|z|)

(Intercept) 2.26742 0.03546 63.95 <2e-16 ***

Signif. codes: 0 ‘***’ 0.001 ‘**’ 0.01 ‘*’ 0.05 ‘.’ 0.1 ‘ ’ 1

Approximate significance of smooth terms:

edf Ref.df Chi.sq p-value

s(Lat,Lon) 1.366e+01 49 28.564 0.001552 **

s(num.record) 8.801e+00 9 1181.743 < 2e-16 ***

s(temp) 2.160e+00 9 8.874 0.002665 **

s(dissolved.oxygen) 3.642e-04 9 0.000 0.437297

s(chlorophyll) 1.746e+00 9 7.717 0.004930 **

s(current) 2.851e-01 9 0.322 0.274917

s(pH) 5.556e-05 9 0.000 0.573268

s(salinity) 1.998e-04 9 0.000 0.748928

s(depth) 1.858e+00 9 13.121 0.000136 ***

s(PO4) 1.908e-04 9 0.000 0.648641

---

Signif. codes: 0 ‘***’ 0.001 ‘**’ 0.01 ‘*’ 0.05 ‘.’ 0.1 ‘ ’ 1

R-sq.(adj) = 0.858 Deviance explained = 89.3%

-REML = 1174.9 Scale est. = 1 n = 358

> # Model selection for number of traces


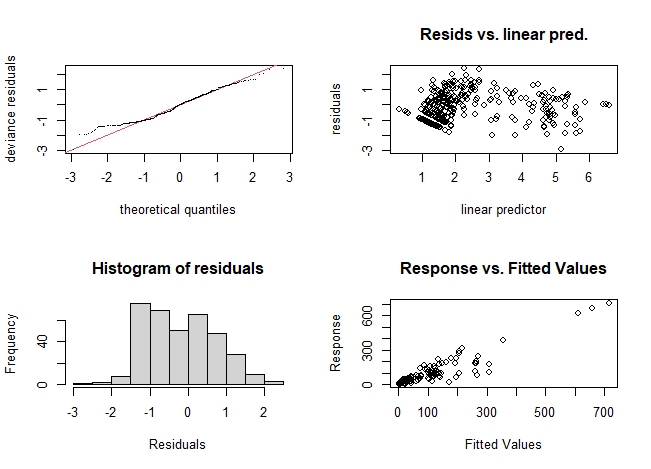


**Figure 59S.** The extracted plots of GAM model for the combination of environmental variables.

## Model selection for the number of species, deep water

Model selection for number of species, deep water based on GAMs analysis was presented.

*> gam.numsp.models <- list(gam.numsp.intercept = gam.numsp.intercept,*

*+ gam.numsp.numrec = gam.numsp.numrec,*

*+ gam.numsp.env=gam.numsp.env,*

*+ gam.numsp.latlon = gam.numsp.latlon,*

*+ gam.numsp.depth = gam.numsp.depth,*

*+ gam.numsp.pH = gam.numsp.pH,*

*+ gam.numsp.current = gam.numsp.current,*

*+ gam.numsp.PO4 = gam.numsp.PO4,*

*+ gam.numsp.chlorophyll= gam.numsp.chlorophyll,*

*+ gam.numsp.dissolved.oxygen = gam.numsp.dissolved.oxygen,*

*+ gam.numsp.salinity= gam.numsp.salinity,*

*+ gam.numsp.temp= gam.numsp.temp)*

*> gam.numsp.aic.df <- data.frame(Model = names(gam.numsp.models),*

*+ AIC = sapply(gam.numsp.models, function(x) x$aic),*

*+ akaike.weights(sapply(gam.numsp.models, function(x) x$aic)))*

*> gam.numsp.aic.df <- gam.numsp.aic.df[order(gam.numsp.aic.df$AIC),]*

*> gam.numsp.aic.df$Cumulative.Weight <- cumsum(gam.numsp.aic.df$weights)*

*> kable(gam.numsp.aic.df, row.names = FALSE)*

| Model | AIC | deltaAIC |  | rel.LL | weights | Cumulative.Weight |
| --- | --- | --- | --- | --- | --- | --- |
| gam.numsp.env | 2103.4373 | 0.0000 |  | 1.0000 | 0.8646 | 0.8646 |
| gam.numsp.depth | 2109.5011 | 6.0638 |  | 0.0482 | 0.0417 | 0.9062 |
| gam.numsp.temp | 2109.6671 | 6.2298 |  | 0.0444 | 0.0384 | 0.9446 |
| gam.numsp.pH | 2110.4163 | 6.9790 |  | 0.0305 | 0.0264 | 0.9710 |
| gam.numsp.dissolved.oxygen | 2112.5351 | 9.0978 |  | 0.0106 | 0.0091 | 0.9801 |
| gam.numsp.current | 2112.7896 | 9.3523 |  | 0.0093 | 0.0081 | 0.9882 |
| gam.numsp.salinity | 2113.8399 | 10.4026 |  | 0.0055 | 0.0048 | 0.9930 |
| gam.numsp.PO4 | 2114.4445 | 11.0072 |  | 0.0041 | 0.0035 | 0.9965 |
| gam.numsp.chlorophyll | 2114.4445 | 11.0072 |  | 0.0041 | 0.0035 | 1.0000 |
| gam.numsp.numrec | 2136.0300 | 32.5927 |  | 0.0000 | 0.0000 | 1.0000 |
| gam.numsp.latlon | 2227.3333 | 123.8960 |  | 0.0000 | 0.0000 | 1.0000 |
| gam.numsp.intercept | 2799.8970 | 696.4597 |  | 0.0000 | 0.0000 | 1.0000 |

The results show the combination of environmental parameter could be modeled the distribution of the number of species in deep sea.

## Plots of GMAs analysis of the number of species in the deep sea

The extracted plots of the relation between environmental variables and the extracted models were presented for GAMs analysis of the number of species in deep sea.


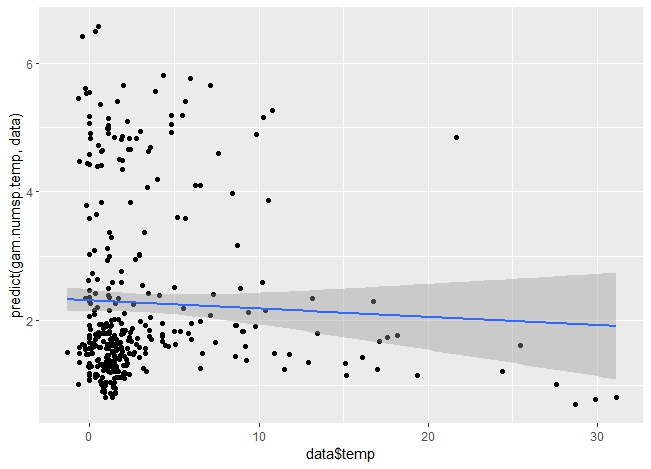


**Figure 60S.** qplot(data$temp, predict(gam.numsp.temp, data)) + geom_smooth(method = "glm")


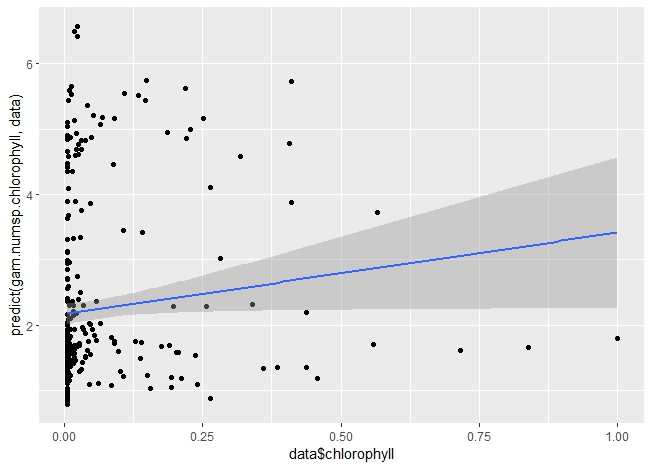


**Figure 61S.** qplot(data$chlorophyll, predict(gam.numsp.chlorophyll, data)) + geom_smooth(method = "glm")


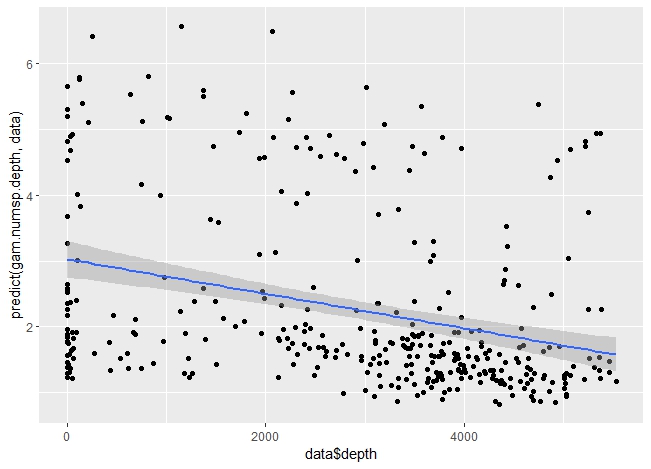


**Figure 62S.**qplot(data$depth, predict(gam.numsp.depth, data)) + geom_smooth(method = "glm")


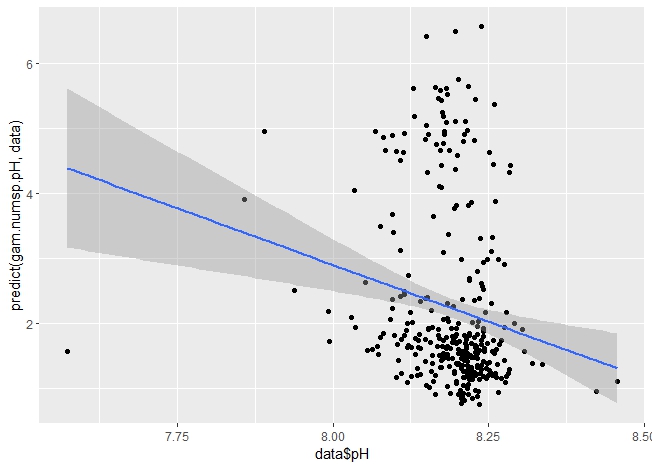


**Figure 63S.**qplot(data$pH, predict(gam.numsp.pH, data)) + geom_smooth(method = "glm")


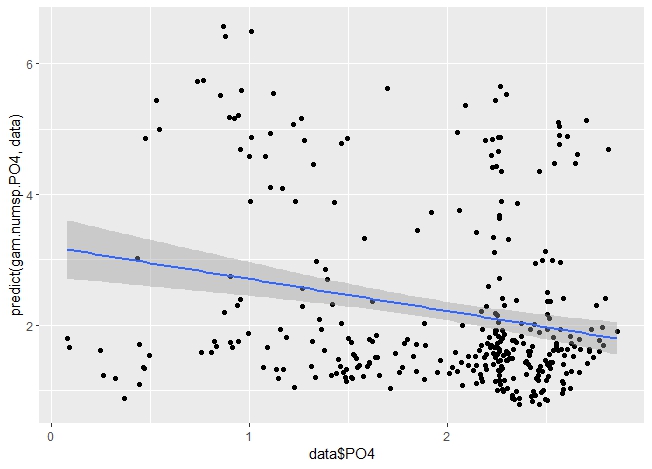


**Figure 64S.**qplot(data$PO4, predict(gam.numsp.PO4, data)) + geom_smooth(method = "glm")


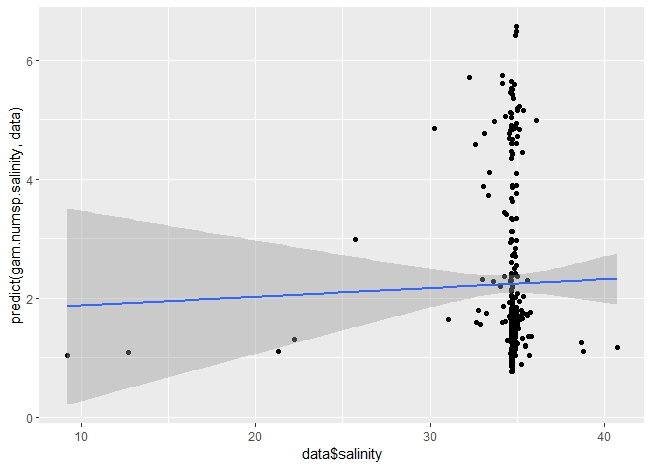


**Figure 65S.**qplot(data$salinity.clay, predict(gam.numsp.salinity, data)) + geom_smooth(method = "glm")


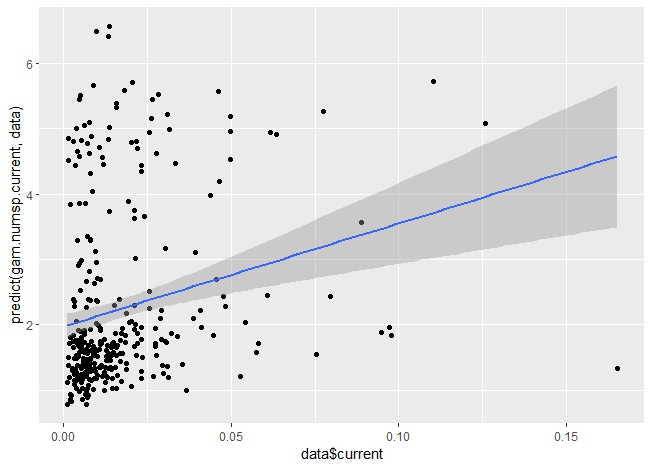


**Figure 66S.**qplot(GLM$current, predict(gam.numsp.current, data)) + geom_smooth(method = "glm")


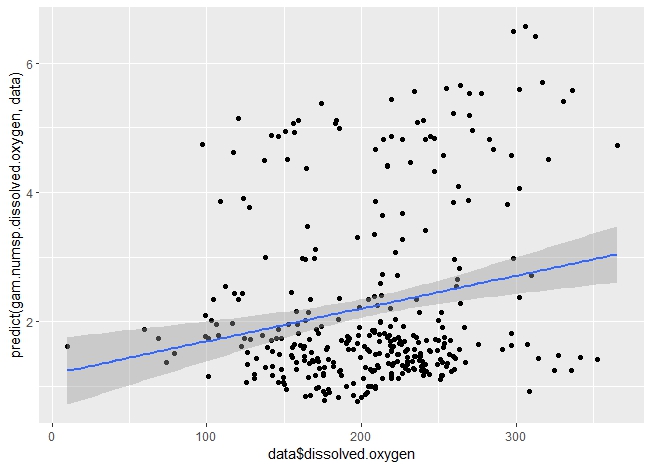


**Figure 67S.**qplot(GLM$dissolved.oxygen, predict(gam.numsp.dissolved.oxygen, data)) + geom_smooth(method = "glm")

# GAMs for ES50, deep sea

The scripts and extracted plots and figures of the GAMs analysis for ES50 in deep sea were presented.

1) GAM model for intercept

*> gam.ES50.intercept <- gam(ES50 ~ 1, data = data, family = "nb", method = "REML", select = TRUE)*

*> gam.check(gam.ES50.intercept)*

Method: REML Optimizer: outer newton

full convergence after 3 iterations.

Gradient range [-4.22982e-08,-4.22982e-08]

(score 1020.329 & scale 1).

Hessian positive definite, eigenvalue range [95.2136,95.2136].

Model rank = 1 / 1

*summary(gam.ES50.intercept)*

Family: Negative Binomial(2.162)

Link function: log

Formula:

ES50 ~ 1 Parametric coefficients:

Estimate Std. Error z value Pr(>|z|)

(Intercept) 1.85387 0.04159 44.58 <2e-16 ***

Signif. codes: 0 ‘***’ 0.001 ‘**’ 0.01 ‘*’ 0.05 ‘.’ 0.1 ‘ ’ 1

R-sq.(adj) = 0 Deviance explained = 1.82e-09%

-REML = 1020.3 Scale est. = 1 n = 358


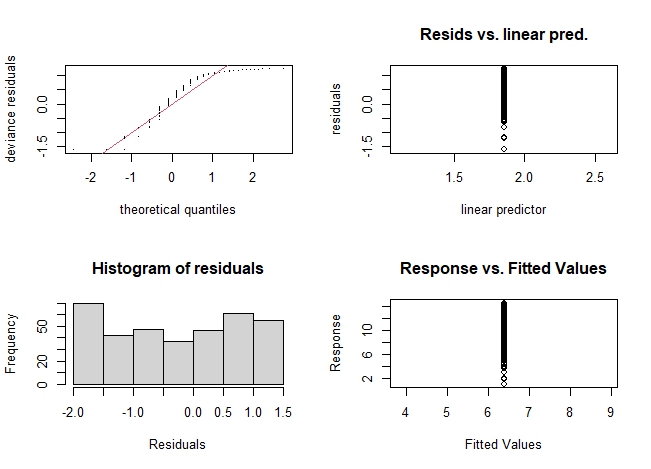


**Figure 68S.** The extracted plots of GLAM model for intercept

2) GAM mmodel for spatial autocorellation

*gam.ES50.latlon <- gam(ES50 ~ s(Lat, Lon, bs = "sos") , data = data, family= "nb", method = "REML", select = TRUE)*

*> gam.check(gam.ES50.latlon)*

Method: REML Optimizer: outer newton

full convergence after 5 iterations.

Gradient range [-8.213703e-08,-8.09273e-09]

(score 977.9552 & scale 1).

Hessian positive definite, eigenvalue range [9.190182,42.13718].

Model rank = 50 / 50

Basis dimension (k) checking results. Low p-value (k-index<1) may

indicate that k is too low, especially if edf is close to k'.

k' edf k-index p-value

s(Lat,Lon) 49.0 32.9 1 0.8

*summary(gam.ES50.latlon)*

Family: Negative Binomial(4.572)

Link function: log

Formula:

ES50 ~ s(Lat, Lon, bs = "sos")

Parametric coefficients:

Estimate Std. Error z value Pr(>|z|)

(Intercept) 1.73220 0.03407 50.84 <2e-16 ***

Signif. codes: 0 ‘***’ 0.001 ‘**’ 0.01 ‘*’ 0.05 ‘.’ 0.1 ‘ ’ 1

Approximate significance of smooth terms:

edf Ref.df Chi.sq p-value

s(Lat,Lon) 32.92 49 207.2 <2e-16 ***

Signif. codes: 0 ‘***’ 0.001 ‘**’ 0.01 ‘*’ 0.05 ‘.’ 0.1 ‘ ’ 1

R-sq.(adj) = 0.388 Deviance explained = 41.9%

-REML = 977.96 Scale est. = 1 n = 358


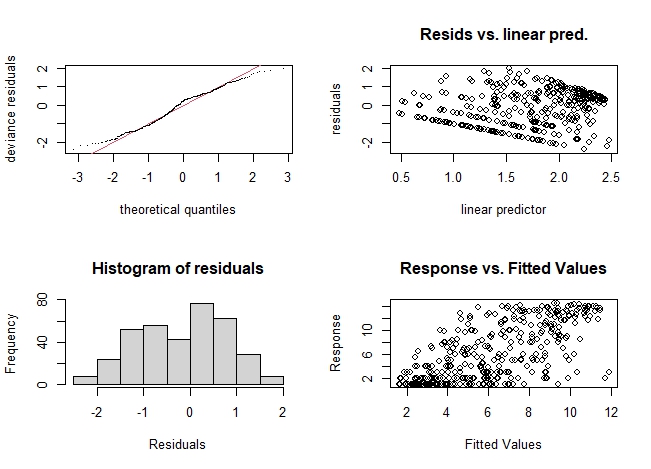


**Figure 69S.** The extracted plots of GLAM model for spatial autocorrelation.

3) GAM model for depth

*gam.ES50.depth <- gam(ES50 ~ s(Lat,Lon, bs="sos")+ +s(depth), data = data, family = "nb", method = "REML", select = TRUE)*

*> gam.check(gam.ES50.depth)*

Method: REML Optimizer: outer newton

full convergence after 5 iterations.

Gradient range [-0.0001537147,6.445491e-05]

(score 974.6166 & scale 1).

Hessian positive definite, eigenvalue range [0.000153662,40.5324].

Model rank = 59 / 59

Basis dimension (k) checking results. Low p-value (k-index<1) may

indicate that k is too low, especially if edf is close to k'.

k' edf k-index p-value

s(Lat,Lon) 49.00 31.81 1.01 0.75

s(depth) 9.00 1.97 0.89 0.05 *

Signif. codes: 0 ‘***’ 0.001 ‘**’ 0.01 ‘*’ 0.05 ‘.’ 0.1 ‘ ’ 1

*summary(gam.ES50.depth)*

Family: Negative Binomial(4.754)

Link function: log

Formula:

ES50 ~ s(Lat, Lon, bs = "sos") + +s(depth)

Parametric coefficients:

Estimate Std. Error z value Pr(>|z|)

(Intercept) 1.72930 0.03375 51.24 <2e-16 ***

Signif. codes: 0 ‘***’ 0.001 ‘**’ 0.01 ‘*’ 0.05 ‘.’ 0.1 ‘ ’ 1

Approximate significance of smooth terms:

edf Ref.df Chi.sq p-value

s(Lat,Lon) 31.815 49 172.45 < 2e-16 ***

s(depth) 1.967 9 10.93 0.00106 **

Signif. codes: 0 ‘***’ 0.001 ‘**’ 0.01 ‘*’ 0.05 ‘.’ 0.1 ‘ ’ 1

R-sq.(adj) = 0.397 Deviance explained = 43.2%

-REML = 974.62 Scale est. = 1 n = 358


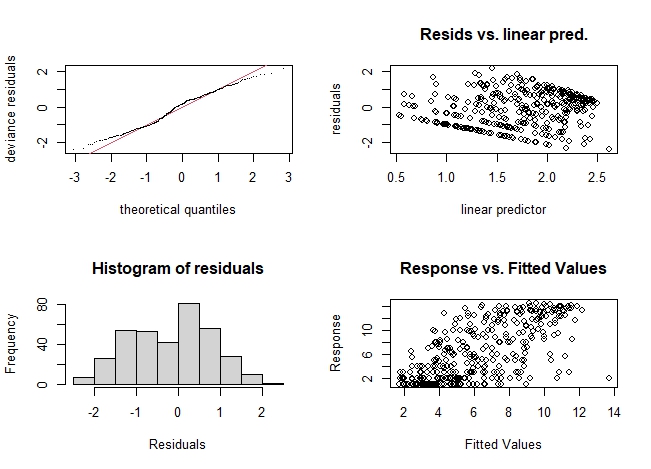


**Figure 70S.** The extracted plots of GLAM model for depth

1. GAM model for chlorophyll

*gam.ES50.chlorophyll <- gam(ES50 ~ s(Lat,Lon, bs="sos")+ s(chlorophyll), data = data, family = "nb", method = "REML", select = TRUE)*

*> gam.check(gam.ES50.chlorophyll)*

Method: REML Optimizer: outer newton

full convergence after 10 iterations.

Gradient range [-0.0009479507,0.0008383627]

(score 977.9557 & scale 1).

Hessian positive definite, eigenvalue range [0.0001600974,42.13529].

Model rank = 59 / 59

Basis dimension (k) checking results. Low p-value (k-index<1) may

indicate that k is too low, especially if edf is close to k'.

k' edf k-index p-value

s(Lat,Lon) 49.00000 32.92102 1.00 0.80

s(chlorophyll) 9.00000 0.00486 0.94 0.28

summary(gam.ES50.chlorophyll)

Family: Negative Binomial(4.572)

Link function: log

Formula:

ES50 ~ s(Lat, Lon, bs = "sos") + s(chlorophyll)

Parametric coefficients:

Estimate Std. Error z value Pr(>|z|)

(Intercept) 1.73220 0.03407 50.84 <2e-16 ***

Signif. codes: 0 ‘***’ 0.001 ‘**’ 0.01 ‘*’ 0.05 ‘.’ 0.1 ‘ ’ 1

Approximate significance of smooth terms:

edf Ref.df Chi.sq p-value

s(Lat,Lon) 32.921023 49 207.221 <2e-16 ***

s(chlorophyll) 0.004863 9 0.004 0.4

Signif. codes: 0 ‘***’ 0.001 ‘**’ 0.01 ‘*’ 0.05 ‘.’ 0.1 ‘ ’ 1

R-sq.(adj) = 0.388 Deviance explained = 41.9%

-REML = 977.96 Scale est. = 1 n = 358


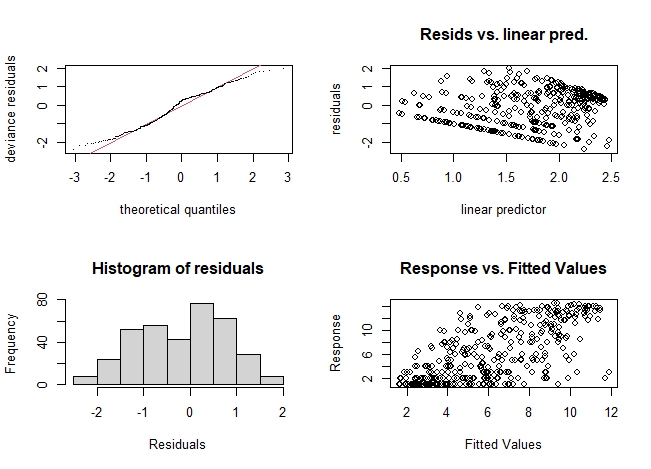


**Figure 71S.** The extracted plots of GLAM model for chlorophyll

1. GAM model for current

*gam.ES50.current <- gam(ES50 ~ s(Lat,Lon, bs="sos")+s(current), data = data, family = "nb", method = "REML", select = TRUE)*

*> gam.check(gam.ES50.current)*

Method: REML Optimizer: outer newton

full convergence after 6 iterations.

Gradient range [-0.0002724523,0.0002143798]

(score 977.1664 & scale 1).

Hessian positive definite, eigenvalue range [0.0002721562,41.24989].

Model rank = 59 / 59

Basis dimension (k) checking results. Low p-value (k-index<1) may

indicate that k is too low, especially if edf is close to k'.

k' edf k-index p-value

s(Lat,Lon) 49.00 32.76 1.01 0.835

s(current) 9.00 1.36 0.87 0.015 *

Signif. codes: 0 ‘***’ 0.001 ‘**’ 0.01 ‘*’ 0.05 ‘.’ 0.1 ‘ ’ 1

summary(gam.ES50.current)

Family: Negative Binomial(4.654)

Link function: log

Formula:

ES50 ~ s(Lat, Lon, bs = "sos") + s(current)

Parametric coefficients:

Estimate Std. Error z value Pr(>|z|)

(Intercept) 1.73047 0.03393 51 <2e-16 ***

Signif. codes: 0 ‘***’ 0.001 ‘**’ 0.01 ‘*’ 0.05 ‘.’ 0.1 ‘ ’ 1

Approximate significance of smooth terms:

edf Ref.df Chi.sq p-value

s(Lat,Lon) 32.764 49 199.20 <2e-16 ***

s(current) 1.363 9 3.77 0.0459 *

---

Signif. codes: 0 ‘***’ 0.001 ‘**’ 0.01 ‘*’ 0.05 ‘.’ 0.1 ‘ ’ 1

R-sq.(adj) = 0.393 Deviance explained = 42.7%

-REML = 977.17 Scale est. = 1 n = 358


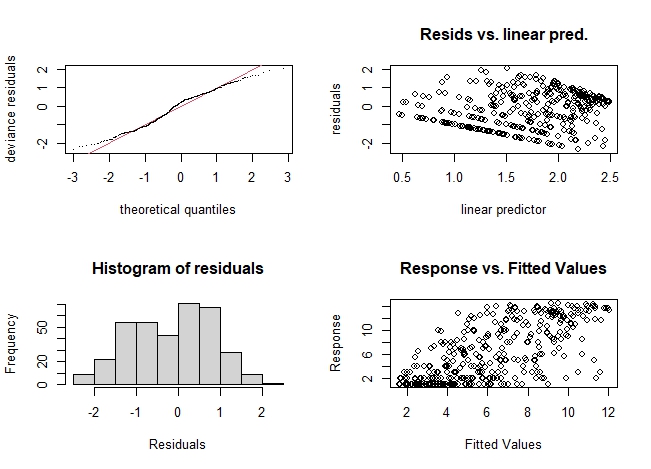


**Figure 72S.** The extracted plots of GLAM model for current

1. GAM model for pH

*gam.ES50.pH <- gam(ES50 ~ s(Lat,Lon, bs="sos")+s(pH), data = data, family = "nb", method = "REML", select = TRUE)*

*> gam.check(gam.ES50.pH)*

Method: REML Optimizer: outer newton

full convergence after 13 iterations.

Gradient range [-0.0005061342,6.183449e-05]

(score 977.8799 & scale 1).

Hessian positive definite, eigenvalue range [0.0004452891,41.95094].

Model rank = 59 / 59

Basis dimension (k) checking results. Low p-value (k-index<1) may

indicate that k is too low, especially if edf is close to k'.

k' edf k-index p-value

s(Lat,Lon) 49.000 32.897 1.00 0.8

s(pH) 9.000 0.398 0.84 <2e-16 ***

Signif. codes: 0 ‘***’ 0.001 ‘**’ 0.01 ‘*’ 0.05 ‘.’ 0.1 ‘ ’ 1

*summary(gam.ES50.pH)*

Family: Negative Binomial(4.587)

Link function: log

Formula:

ES50 ~ s(Lat, Lon, bs = "sos") + s(pH)

Parametric coefficients:

Estimate Std. Error z value Pr(>|z|)

(Intercept) 1.73184 0.03404 50.87 <2e-16 ***

Signif. codes: 0 ‘***’ 0.001 ‘**’ 0.01 ‘*’ 0.05 ‘.’ 0.1 ‘ ’ 1

Approximate significance of smooth terms:

edf Ref.df Chi.sq p-value

s(Lat,Lon) 32.8966 49 205.225 <2e-16 ***

s(pH) 0.3977 9 0.657 0.186

Signif. codes: 0 ‘***’ 0.001 ‘**’ 0.01 ‘*’ 0.05 ‘.’ 0.1 ‘ ’ 1

R-sq.(adj) = 0.389 Deviance explained = 42.1%

-REML = 977.88 Scale est. = 1 n = 358


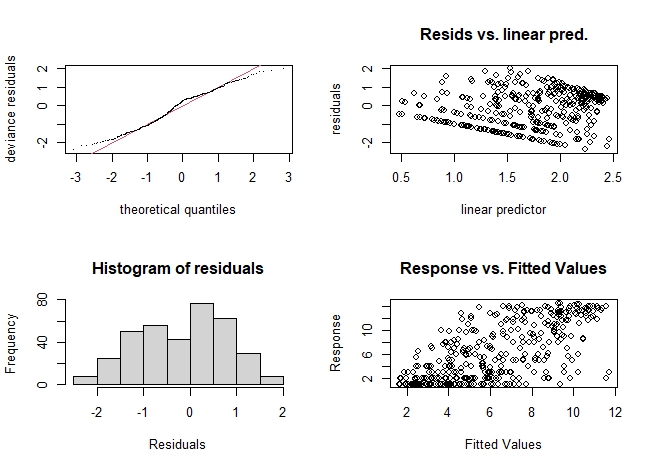


**Figure 73S.** The extracted plots of GLAM model for pH

1. GAM model for PO4

*gam.ES50.PO4 <- gam(ES50 ~ s(Lat,Lon, bs="sos")+s(PO4), data = data, family = "nb", method = "REML", select = TRUE)*

*> gam.check(gam.ES50.PO4)*

Method: REML Optimizer: outer newton

full convergence after 7 iterations.

Gradient range [-0.0004403149,0.0001190003]

(score 977.9557 & scale 1).

Hessian positive definite, eigenvalue range [6.62143e-05,42.1371].

Model rank = 59 / 59

Basis dimension (k) checking results. Low p-value (k-index<1) may

indicate that k is too low, especially if edf is close to k'.

k' edf k-index p-value

s(Lat,Lon) 49.00000 32.92093 1.00 0.77

s(PO4) 9.00000 0.00238 0.97 0.46

summary(gam.ES50.PO4)

Family: Negative Binomial(4.572)

Link function: log

Formula:

ES50 ~ s(Lat, Lon, bs = "sos") + s(PO4)

Parametric coefficients:

Estimate Std. Error z value Pr(>|z|)

(Intercept) 1.73220 0.03407 50.84 <2e-16 ***

Signif. codes: 0 ‘***’ 0.001 ‘**’ 0.01 ‘*’ 0.05 ‘.’ 0.1 ‘ ’ 1

Approximate significance of smooth terms:

edf Ref.df Chi.sq p-value

s(Lat,Lon) 32.920929 49 207.166 <2e-16 ***

s(PO4) 0.002382 9 0.001 0.462

Signif. codes: 0 ‘***’ 0.001 ‘**’ 0.01 ‘*’ 0.05 ‘.’ 0.1 ‘ ’ 1

R-sq.(adj) = 0.388 Deviance explained = 41.9%

-REML = 977.96 Scale est. = 1 n = 358


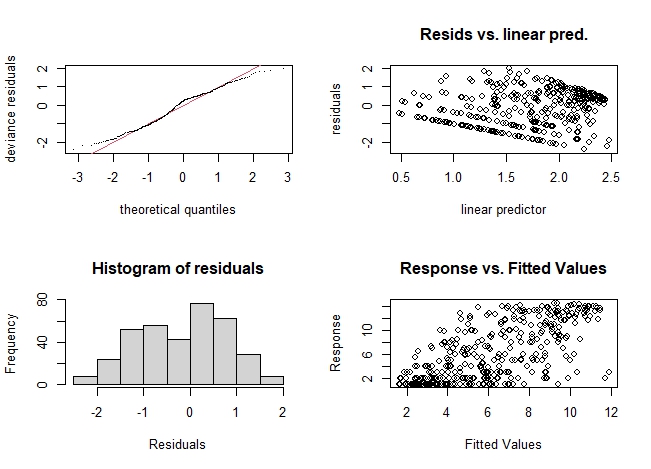


**Figure 74S.** The extracted plots of GLAM model for PO4

1. GAM model for salinity

*gam.ES50.salinity <- gam(ES50 ~ s(Lat,Lon, bs="sos")+ s(salinity), data = data, family = "nb", method = "REML", select = TRUE)*

*> gam.check(gam.ES50.salinity)*

Method: REML Optimizer: outer newton

full convergence after 9 iterations.

Gradient range [-0.0005132574,-7.245252e-05]

(score 977.9337 & scale 1).

Hessian positive definite, eigenvalue range [0.000513015,42.01138].

Model rank = 59 / 59

Basis dimension (k) checking results. Low p-value (k-index<1) may

indicate that k is too low, especially if edf is close to k'.

k' edf k-index p-value

s(Lat,Lon) 49.000 32.908 1.00 0.79

s(salinity) 9.000 0.245 0.93 0.20

*summary(gam.ES50.salinity)*

Family: Negative Binomial(4.581)

Link function: log

Formula:

ES50 ~ s(Lat, Lon, bs = "sos") + s(salinity)

Parametric coefficients:

Estimate Std. Error z value Pr(>|z|)

(Intercept) 1.73200 0.03406 50.86 <2e-16 ***

---

Signif. codes: 0 ‘***’ 0.001 ‘**’ 0.01 ‘*’ 0.05 ‘.’ 0.1 ‘ ’ 1

Approximate significance of smooth terms:

edf Ref.df Chi.sq p-value

s(Lat,Lon) 32.9080 49 206.09 <2e-16 ***

s(salinity) 0.2446 9 0.33 0.228

---

Signif. codes: 0 ‘***’ 0.001 ‘**’ 0.01 ‘*’ 0.05 ‘.’ 0.1 ‘ ’ 1

R-sq.(adj) = 0.389 Deviance explained = 42%

-REML = 977.93 Scale est. = 1 n = 358


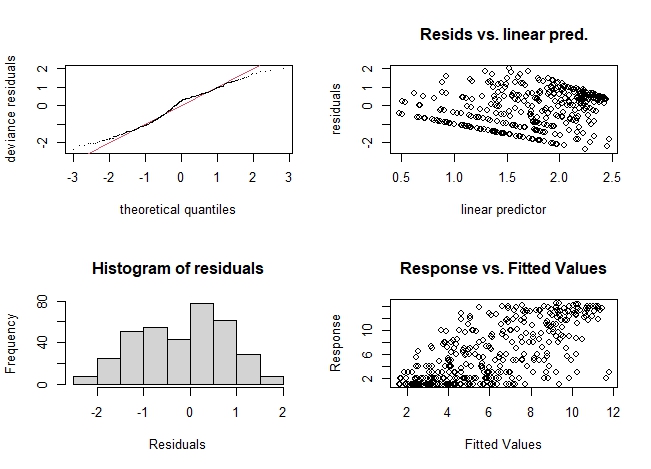


**Figure 75S.** The extracted plots of GLAM model for salinity

1. GAM model for dissolved oxygen

*gam.ES50.dissolved.oxygen <- gam(ES50 ~ s(Lat,Lon, bs="sos")+s(dissolved.oxygen), data = data, family = "nb", method = "REML", select = TRUE)*

*> gam.check(gam.ES50.dissolved.oxygen)*

Method: REML Optimizer: outer newton

full convergence after 12 iterations.

Gradient range [-0.0004767256,0.0002658906]

(score 976.4947 & scale 1).

Hessian positive definite, eigenvalue range [0.0004763086,41.08875].

Model rank = 59 / 59

Basis dimension (k) checking results. Low p-value (k-index<1) may

indicate that k is too low, especially if edf is close to k'.

k' edf k-index p-value

s(Lat,Lon) 49.000 32.827 1.00 0.74

s(dissolved.oxygen) 9.000 0.834 0.95 0.29

*summary(gam.ES50.dissolved.oxygen)*

Family: Negative Binomial(4.685)

Link function: log

Formula:

ES50 ~ s(Lat, Lon, bs = "sos") + s(dissolved.oxygen)

Parametric coefficients:

Estimate Std. Error z value Pr(>|z|)

(Intercept) 1.73008 0.03388 51.06 <2e-16 ***

Signif. codes: 0 ‘***’ 0.001 ‘**’ 0.01 ‘*’ 0.05 ‘.’ 0.1 ‘ ’ 1

Approximate significance of smooth terms:

edf Ref.df Chi.sq p-value

s(Lat,Lon) 32.8271 49 214.25 <2e-16 ***

s(dissolved.oxygen) 0.8343 9 4.84 0.0127 *

Signif. codes: 0 ‘***’ 0.001 ‘**’ 0.01 ‘*’ 0.05 ‘.’ 0.1 ‘ ’ 1

R-sq.(adj) = 0.392 Deviance explained = 42.7%

-REML = 976.49 Scale est. = 1 n = 358


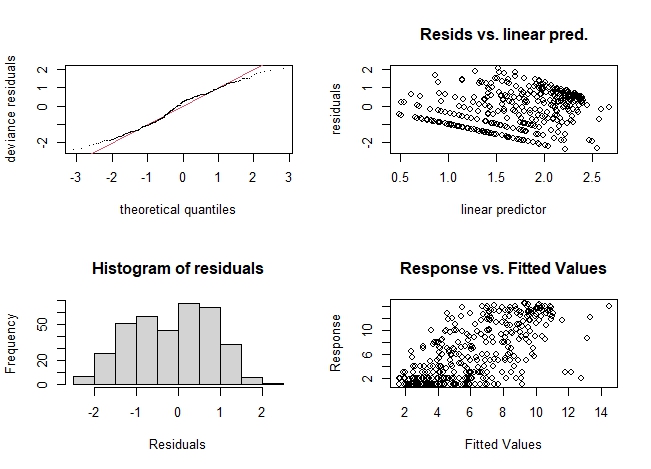


**Figure 76S.** The extracted plots of GLAM model for dissolved oxygen

1. GAM model for temperature

*gam.ES50.temp <- gam(ES50 ~ s(Lat,Lon, bs="sos")+s(temp), data = data, family = "nb", method = "REML", select = TRUE)*

*> gam.check(gam.ES50.temp)*

Method: REML Optimizer: outer newton

full convergence after 5 iterations.

Gradient range [-0.00010305,7.692359e-05]

(score 976.5435 & scale 1).

Hessian positive definite, eigenvalue range [0.0001030117,40.74932].

Model rank = 59 / 59

Basis dimension (k) checking results. Low p-value (k-index<1) may

indicate that k is too low, especially if edf is close to k'.

k' edf k-index p-value

s(Lat,Lon) 49.00 32.08 1.01 0.78

s(temp) 9.00 2.21 0.95 0.28

*summary(gam.ES50.temp)*

Family: Negative Binomial(4.697)

Link function: log

Formula:

ES50 ~ s(Lat, Lon, bs = "sos") + s(temp)

Parametric coefficients:

Estimate Std. Error z value Pr(>|z|)

(Intercept) 1.72968 0.03386 51.08 <2e-16 ***

Signif. codes: 0 ‘***’ 0.001 ‘**’ 0.01 ‘*’ 0.05 ‘.’ 0.1 ‘ ’ 1

Approximate significance of smooth terms:

edf Ref.df Chi.sq p-value

s(Lat,Lon) 32.084 49 199.075 <2e-16 ***

s(temp) 2.209 9 7.686 0.012 *

Signif. codes: 0 ‘***’ 0.001 ‘**’ 0.01 ‘*’ 0.05 ‘.’ 0.1 ‘ ’ 1

R-sq.(adj) = 0.396 Deviance explained = 43%

-REML = 976.54 Scale est. = 1 n = 358


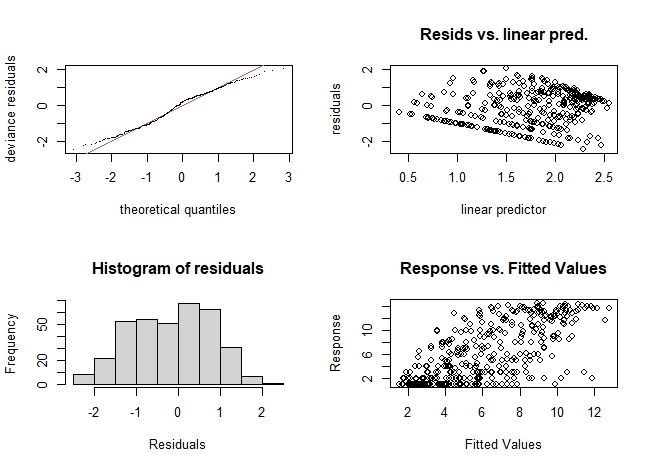


**Figure 77S.** The extracted plots of GLAM model for temprature

1. GAM model for the combination of environmental variables

*gam.ES50.env <- gam(ES50 ~ s(Lat,Lon, bs="sos")+ s (temp) + s(dissolved.oxygen) + s(chlorophyll) + s(current) + s(pH) + s(salinity) + s(depth)+ s(PO4) +s (current) , data = data, family = "nb", method= "REML", select = TRUE)*

*> gam.check(gam.ES50.env)*

Method: REML Optimizer: outer newton

full convergence after 11 iterations.

Gradient range [-0.0001040095,5.772563e-05]

(score 971.9786 & scale 1).

Hessian positive definite, eigenvalue range [1.919002e-06,36.96011].

Model rank = 122 / 122

Basis dimension (k) checking results. Low p-value (k-index<1) may

indicate that k is too low, especially if edf is close to k'.

k' edf k-index p-value

s(Lat,Lon) 4.90e+01 3.17e+01 1.00 0.750

s(temp) 9.00e+00 1.70e+00 0.96 0.435

s(dissolved.oxygen) 9.00e+00 7.46e-01 0.95 0.230

s(chlorophyll) 9.00e+00 1.37e+00 0.98 0.585

s(current) 9.00e+00 1.40e+00 0.86 0.005 **

s(pH) 9.00e+00 2.71e-05 0.87 0.020 *

s(salinity) 9.00e+00 1.87e-04 0.97 0.460

s(depth) 9.00e+00 1.56e+00 0.90 0.075 .

s(PO4) 9.00e+00 2.74e-04 0.99 0.675

Signif. codes: 0 ‘***’ 0.001 ‘**’ 0.01 ‘*’ 0.05 ‘.’ 0.1 ‘ ’ 1

summary(gam.ES50.env)

Family: Negative Binomial(5.128)

Link function: log

Formula:

ES50 ~ s(Lat, Lon, bs = "sos") + s(temp) + s(dissolved.oxygen) +

s(chlorophyll) + s(current) + s(pH) + s(salinity) + s(depth) +

s(PO4) + s(current)

Parametric coefficients:

Estimate Std. Error z value Pr(>|z|)

(Intercept) 1.72158 0.03323 51.81 <2e-16 ***

---

Signif. codes: 0 ‘***’ 0.001 ‘**’ 0.01 ‘*’ 0.05 ‘.’ 0.1 ‘ ’ 1

Approximate significance of smooth terms:

edf Ref.df Chi.sq p-value

s(Lat,Lon) 3.172e+01 49 164.233 <2e-16 ***

s(temp) 1.704e+00 9 4.323 0.0296 *

s(dissolved.oxygen) 7.455e-01 9 2.841 0.0320 *

s(chlorophyll) 1.368e+00 9 3.545 0.0417 *

s(current) 1.399e+00 9 4.418 0.0249 *

s(pH) 2.707e-05 9 0.000 0.7984

s(salinity) 1.874e-04 9 0.000 0.6247

s(depth) 1.560e+00 9 5.403 0.0116 *

s(PO4) 2.745e-04 9 0.000 0.7131

---

Signif. codes: 0 ‘***’ 0.001 ‘**’ 0.01 ‘*’ 0.05 ‘.’ 0.1 ‘ ’ 1

R-sq.(adj) = 0.419 Deviance explained = 46.2%

-REML = 971.98 Scale est. = 1 n = 358


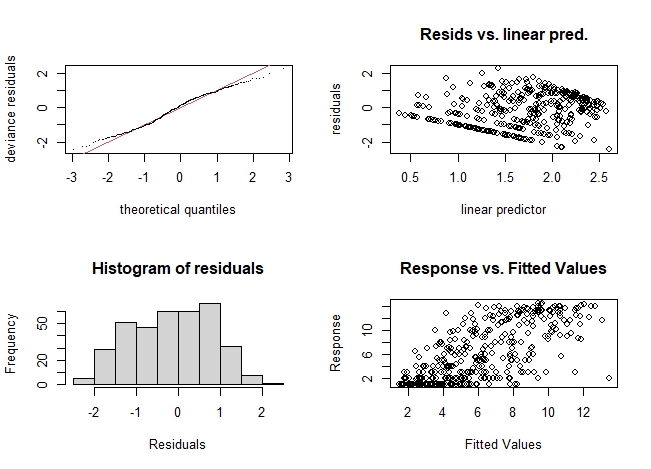


**Figure 78S.** The extracted plots of GLAM model for the combination of environmental variables

## Model selection for ES50 deep sea

*Model selection for number of traces*

*gam.ES50.models <- list(gam.ES50.intercept = gam.ES50.intercept,*

*gam.ES50.env=gam.ES50.env,*

*gam.ES50.latlon = gam.ES50.latlon,*

*gam.ES50.depth = gam.ES50.depth,*

*gam.ES50.pH = gam.ES50.pH,*

*gam.ES50.current = gam.ES50.current,*

*gam.ES50.PO4 = gam.ES50.PO4,*

*gam.ES50.chlorophyll= gam.ES50.chlorophyll,*

*gam.ES50.dissolved.oxygen = gam.ES50.dissolved.oxygen,*

*gam.ES50.salinity= gam.ES50.salinity,*

*gam.ES50.temp= gam.ES50.temp)*

*gam.ES50.aic.df <- data.frame(Model = names(gam.ES50.models),*

*AIC = sapply(gam.ES50.models, function(x) x$aic),*

*akaike.weights(sapply(gam.ES50.models, function(x) x$aic)))*

*gam.ES50.aic.df <- gam.ES50.aic.df[order(gam.ES50.aic.df$AIC),]*

*gam.ES50.aic.df$Cumulative.Weight <- cumsum(gam.ES50.aic.df$weights)*

*kable(gam.ES50.aic.df, row.names = FALSE)*

| Model | AIC | deltaAIC | rel.LL | weights | Cumulative.Weight |
| --- | --- | --- | --- | --- | --- |
| gam.ES50.env | 1754.0449 | 0.0000 | 1.0000 | 0.9970 | 0.9970 |
| gam.ES50.depth | 1767.1870 | 13.1421 | 0.0014 | 0.0014 | 0.9984 |
| gam.ES50.pH | 1768.9251 | 14.8802 | 0.0006 | 0.0006 | 0.9990 |
| gam.ES50.temp | 1769.6766 | 15.6317 | 0.0004 | 0.0004 | 0.9994 |
| gam.ES50.salinity | 1770.0239 | 15.9790 | 0.0003 | 0.0003 | 0.9997 |
| gam.ES50.dissolved.oxygen | 1772.1314 | 18.0865 | 0.0001 | 0.0001 | 0.9998 |
| gam.ES50.current | 1772.2759 | 18.2310 | 0.0001 | 0.0001 | 0.9999 |
| gam.ES50.latlon | 1775.6486 | 21.6037 | 0.0000 | 0.0000 | 1.0000 |
| gam.ES50.PO4 | 1775.6496 | 21.6047 | 0.0000 | 0.0000 | 1.0000 |
| gam.ES50.chlorophyll | 1775.6504 | 21.6054 | 0.0000 | 0.0000 | 1.0000 |
| gam.ES50.intercept | 1895.4762 | 141.4313 | 0.0000 | 0.0000 | 1.0000 |

The results show the combination of all environmental factors fit modeled ES50 in deep-sea regions.

## Plots of GMAs analysis of ES50 in the deep sea

The extracted plots of the relation between environmental variables and the extracted models were presented for GAMs analysis of ES50 in deep sea.


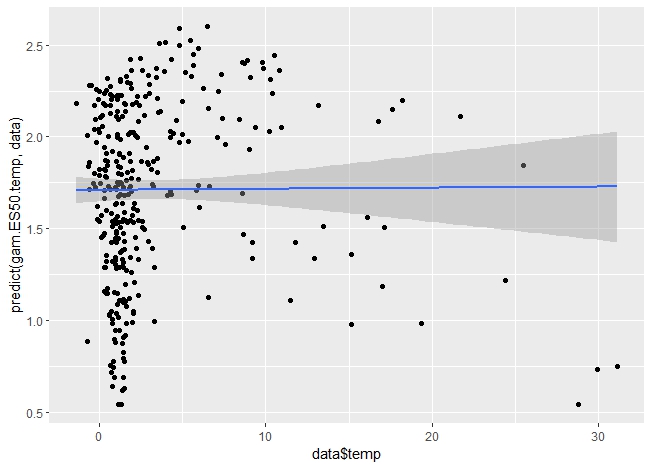


**Figure 79S.** qplot(data$temp, predict(gam.ES50.temp, data)) + geom_smooth(method = "glm")


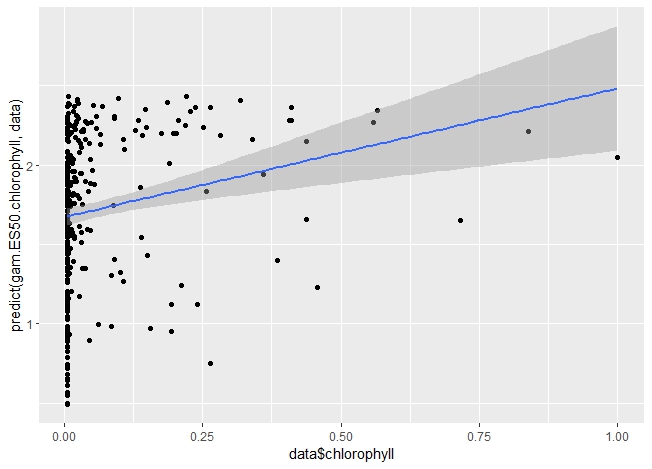


**Figure 80S.**qplot(data$chlorophyll, predict(gam.ES50.chlorophyll, data)) + geom_smooth(method = "glm")


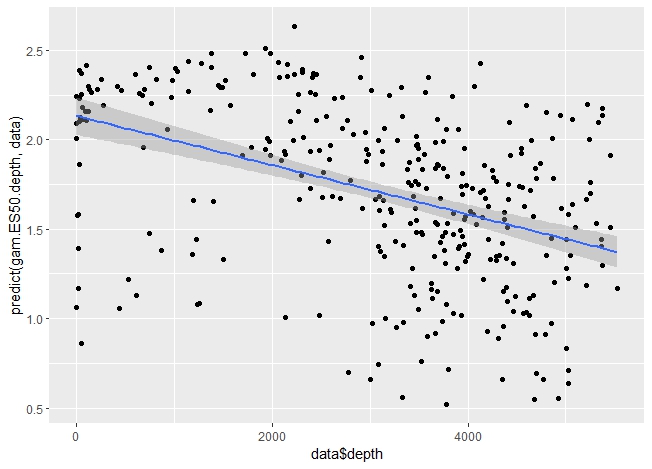


**Figure 81S.**qplot(data$depth, predict(gam.ES50.depth, data)) + geom_smooth(method = "glm")


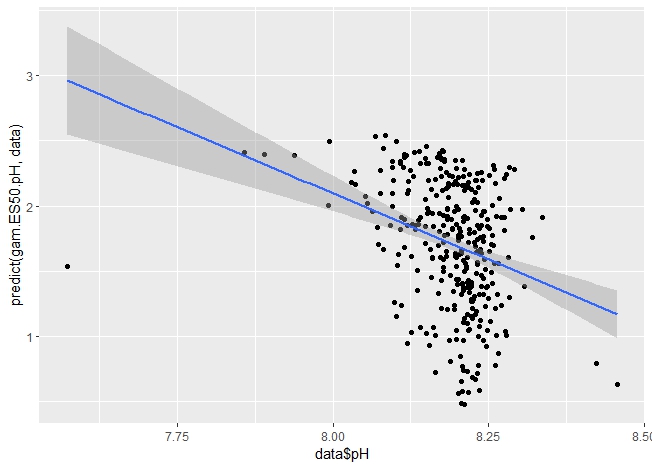


**Figure 82S.** qplot(data$pH, predict(gam.ES50.pH, data)) + geom_smooth(method = "glm")


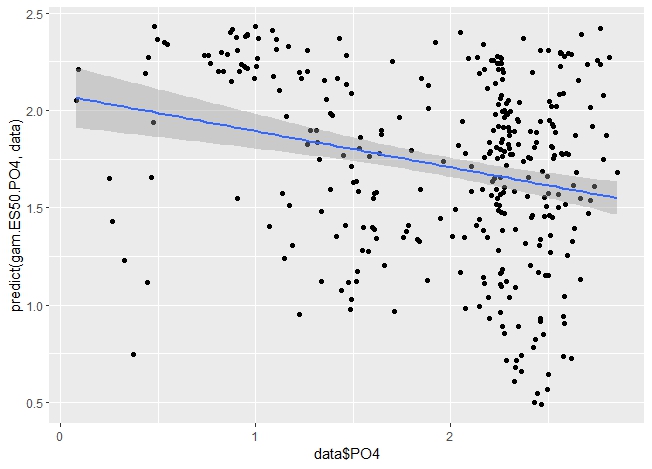


**Figure 83S.** qplot(data$PO4, predict(gam.ES50.PO4, data)) + geom_smooth(method = "glm")


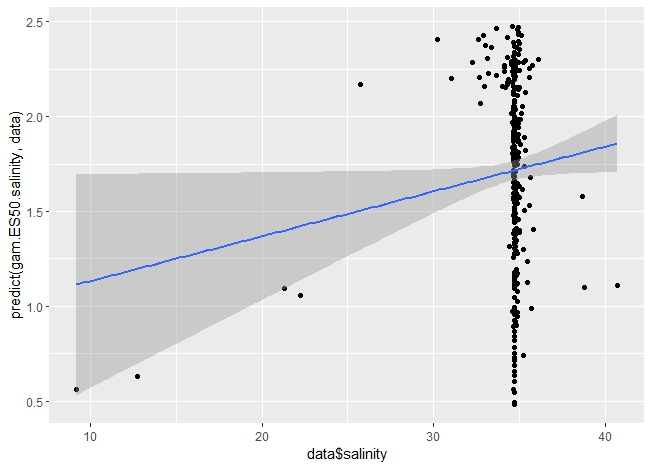


**Figure 84S.**qplot(data$salinity, predict(gam.ES50.salinity, data)) + geom_smooth(method = "glm")


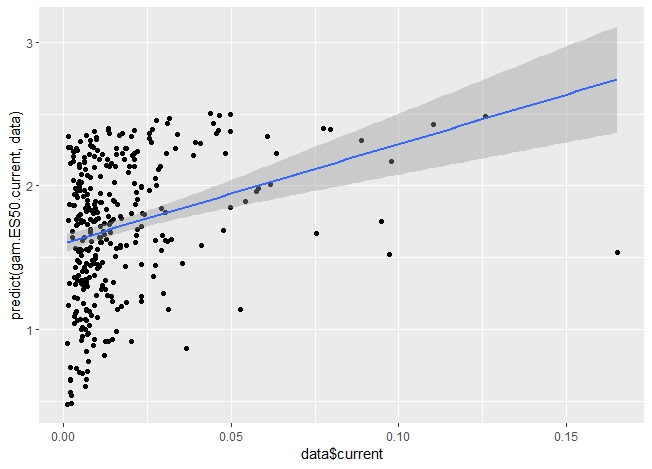


**Figure 85S.** qplot(data$current, predict(gam.ES50.current, data)) + geom_smooth(method = "glm")


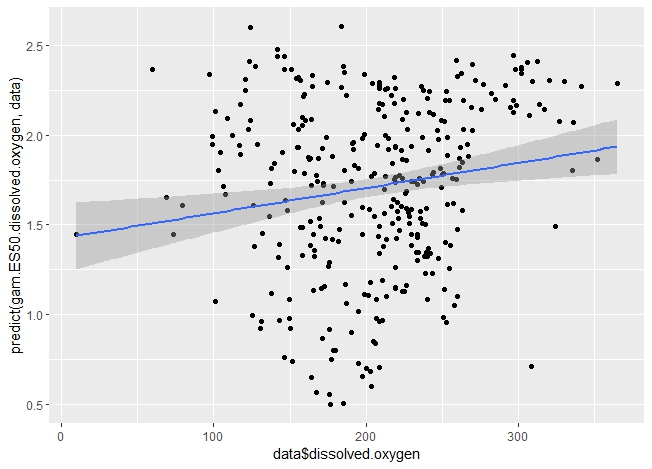


**Figure 86S.** qplot(data$dissolved.oxygen, predict(gam.ES50.dissolved.oxygen, data)) + geom_smooth(method = "glm")

# GLM for number of species, the whole database

The scripts, plots, and figures of GLM analysis for the number of species of total benthic species are presented here.

1. GLM model for intercept

*GLM.numsp.intercept <- glm(No.species ~ 1, family = "poisson", data = GLM)*

*summary(GLM.numsp.intercept)*

Call:

glm(formula = No.species ~ 1, family = "poisson", data = GLM)

Coefficients:

Estimate Std. Error z value Pr(>|z|)

(Intercept) 6.393542 0.007013 911.6 <2e-16 ***

Signif. codes: 0 ‘***’ 0.001 ‘**’ 0.01 ‘*’ 0.05 ‘.’ 0.1 ‘ ’ 1

(Dispersion parameter for poisson family taken to be 1)

Null deviance: 9271.9 on 33 degrees of freedom

Residual deviance: 9271.9 on 33 degrees of freedom

AIC: 9543.4

Number of Fisher Scoring iterations: 5

1. GLM moel for the number of records

*GLM.numsp.numrec <- glm(No.species ~ No.records, family = "poisson", data = GLM)*

*summary(GLM.numsp.numrec)*

Call:

glm(formula = No.species ~ No.records, family = "poisson", data = GLM)

Coefficients:

Estimate Std. Error z value Pr(>|z|)

(Intercept) 6.218e+00 8.272e-03 751.67 <2e-16 ***

No.records 7.375e-06 1.398e-07 52.76 <2e-16 ***

Signif. codes: 0 ‘***’ 0.001 ‘**’ 0.01 ‘*’ 0.05 ‘.’ 0.1 ‘ ’ 1

(Dispersion parameter for poisson family taken to be 1)

Null deviance: 9271.9 on 33 degrees of freedom

Residual deviance: 7009.7 on 32 degrees of freedom

AIC: 7283.1

Number of Fisher Scoring iterations: 5

1. GLM model for depth

*GLM.numsp.depth <- glm(No.species ~ No.records + depth, family = "poisson", data = GLM)*

*summary(GLM.numsp.depth)*

Call:

glm(formula = No.species ~ No.records + depth, family = "poisson",

data = GLM)

Coefficients:

Estimate Std. Error z value Pr(>|z|)

(Intercept) 5.979e+00 2.195e-02 272.38 <2e-16 ***

No.records 8.072e-06 1.516e-07 53.24 <2e-16 ***

depth -7.538e-05 6.316e-06 -11.94 <2e-16 ***

Signif. codes: 0 ‘***’ 0.001 ‘**’ 0.01 ‘*’ 0.05 ‘.’ 0.1 ‘ ’ 1

(Dispersion parameter for poisson family taken to be 1)

Null deviance: 9271.9 on 33 degrees of freedom

Residual deviance: 6861.3 on 31 degrees of freedom

AIC: 7136.8

Number of Fisher Scoring iterations: 5

1. GLM for the area

*GLM.numsp.Area <- glm(No.species ~ No.records + Area, family = "poisson", data = GLM)*

*summary(GLM.numsp.depth)*

Call:

glm(formula = No.species ~ No.records + depth, family = "poisson",

data = GLM)

Coefficients:

Estimate Std. Error z value Pr(>|z|)

(Intercept) 5.979e+00 2.195e-02 272.38 <2e-16 ***

No.records 8.072e-06 1.516e-07 53.24 <2e-16 ***

depth -7.538e-05 6.316e-06 -11.94 <2e-16 ***

Signif. codes: 0 ‘***’ 0.001 ‘**’ 0.01 ‘*’ 0.05 ‘.’ 0.1 ‘ ’ 1

(Dispersion parameter for poisson family taken to be 1)

Null deviance: 9271.9 on 33 degrees of freedom

Residual deviance: 6861.3 on 31 degrees of freedom

AIC: 7136.8

Number of Fisher Scoring iterations: 5

1. GLM model for spatial autocorrelation

*GLM.numsp.lat <- glm(No.species ~ No.records + lat_5, family = "poisson", data = GLM)s*

*summary(GLM.numsp.lat)*

Call:

glm(formula = No.species ~ No.records + lat_5, family = "poisson",

data = GLM)

Coefficients:

Estimate Std. Error z value Pr(>|z|)

(Intercept) 6.212e+00 8.342e-03 744.75 <2e-16 ***

No.records 6.272e-06 1.591e-07 39.42 <2e-16 ***

lat_5 2.394e-03 1.644e-04 14.56 <2e-16 ***

Signif. codes: 0 ‘***’ 0.001 ‘**’ 0.01 ‘*’ 0.05 ‘.’ 0.1 ‘ ’ 1

(Dispersion parameter for poisson family taken to be 1)

Null deviance: 9271.9 on 33 degrees of freedom

Residual deviance: 6798.7 on 31 degrees of freedom

AIC: 7074.2

Number of Fisher Scoring iterations: 5

1. GLM model for chlorophyll

*GLM.numsp.chlorophyll <- glm(No.species ~ No.records + chlorophyll family = "poisson", data = GLM)*

*summary(GLM.numsp.chlorophyll)*

Call:

glm(formula = No.species ~ No.records + chlorophyll family = "poisson",

data = GLM)

Coefficients:

Estimate Std. Error z value Pr(>|z|)

(Intercept) 6.099e+00 9.825e-03 620.8 <2e-16 ***

No.records 5.104e-06 1.685e-07 30.3 <2e-16 ***

chlorophyll 3.604e+00 1.408e-01 25.6 <2e-16 ***

Signif. codes: 0 ‘***’ 0.001 ‘**’ 0.01 ‘*’ 0.05 ‘.’ 0.1 ‘ ’ 1

(Dispersion parameter for poisson family taken to be 1)

Null deviance: 9271.9 on 33 degrees of freedom

Residual deviance: 6433.4 on 31 degrees of freedom

AIC: 6708.9

Number of Fisher Scoring iterations: 5

1. GLM model for current

*GLM.numsp.Curvvel <- glm(No.species ~ No.records + current, family = "poisson", data = GLM)*

*summary(GLM.numsp.Curvvel)*

Call:

glm(formula = No.species ~ No.records + current family = "poisson",

data = GLM)

Coefficients:

Estimate Std. Error z value Pr(>|z|)

(Intercept) 6.064e+00 1.819e-02 333.38 <2e-16 ***

No.records 7.007e-06 1.459e-07 48.02 <2e-16 ***

current 1.060e+01 1.103e+00 9.61 <2e-16 ***

Signif. codes: 0 ‘***’ 0.001 ‘**’ 0.01 ‘*’ 0.05 ‘.’ 0.1 ‘ ’ 1

(Dispersion parameter for poisson family taken to be 1)

Null deviance: 9271.9 on 33 degrees of freedom

Residual deviance: 6919.5 on 31 degrees of freedom

AIC: 7195

Number of Fisher Scoring iterations: 5

1. GLM model for pH

*GLM.numsp.pH <- glm(No.species ~ No.records + pH, family = "poisson", data = GLM)*

*summary(GLM.numsp.pH)*

Call:

glm(formula = No.species ~ No.records + pH, family = "poisson",

data = GLM)

Coefficients:

Estimate Std. Error z value Pr(>|z|)

(Intercept) 3.429e+00 1.791e-01 19.14 <2e-16 ***

No.records 7.082e-06 1.405e-07 50.40 <2e-16 ***

pH 3.445e-01 2.189e-02 15.74 <2e-16 ***

Signif. codes: 0 ‘***’ 0.001 ‘**’ 0.01 ‘*’ 0.05 ‘.’ 0.1 ‘ ’ 1

(Dispersion parameter for poisson family taken to be 1)

Null deviance: 9271.9 on 33 degrees of freedom

Residual deviance: 6221.3 on 31 degrees of freedom

AIC: 6496.7

Number of Fisher Scoring iterations: 5

1. GLM model for PO4

*GLM.numsp.PO4 <- glm(No.species ~ No.records + PO4, family = "poisson", data = GLM)*

*summary(GLM.numsp.PO4)*

Call:

glm(formula = No.species ~ No.records + PO4, family = "poisson",

data = GLM)

Coefficients:

Estimate Std. Error z value Pr(>|z|)

(Intercept) 6.576e+00 2.792e-02 235.53 <2e-16 ***

No.records 6.991e-06 1.440e-07 48.56 <2e-16 ***

PO4 -1.862e-01 1.403e-02 -13.28 <2e-16 ***

Signif. codes: 0 ‘***’ 0.001 ‘**’ 0.01 ‘*’ 0.05 ‘.’ 0.1 ‘ ’ 1

(Dispersion parameter for poisson family taken to be 1)

Null deviance: 9271.9 on 33 degrees of freedom

Residual deviance: 6840.4 on 31 degrees of freedom

AIC: 7115.9

Number of Fisher Scoring iterations: 5

1. GLM model for dissolved oxygen

*GLM.numsp.DISSOLVED.OXYGEN<- glm(No.species ~ No.records + DISSOLVED.OXYGEN, family = "poisson", data = GLM)*

*summary(GLM.numsp.dissolved.oxygen)*

Call:

glm(formula = No.species ~ No.records + DISSOLVED.OXYGEN, family = "poisson",

data = GLM)

Coefficients:

Estimate Std. Error z value Pr(>|z|)

(Intercept) 6.155e+00 3.560e-02 172.883 <2e-16 ***

No.records 7.395e-06 1.403e-07 52.698 <2e-16 ***

DISSOLVED.OXYGEN 2.851e-04 1.573e-04 1.813 0.0699 .

Signif. codes: 0 ‘***’ 0.001 ‘**’ 0.01 ‘*’ 0.05 ‘.’ 0.1 ‘ ’ 1

(Dispersion parameter for poisson family taken to be 1)

Null deviance: 9271.9 on 33 degrees of freedom

Residual deviance: 7006.4 on 31 degrees of freedom

AIC: 7281.9

Number of Fisher Scoring iterations: 5

1. GLM model for primary productivity

*GLM.numsp.primary productivity <- glm(No.species ~ No.records + p, family = "poisson", data = GLM)*

*summary(GLM.numsp.primary productivity,)*

Call:

glm(formula = No.species ~ No.records + p, family = "poisson",

data = GLM)

Coefficients:

Estimate Std. Error z value Pr(>|z|)

(Intercept) 6.110e+00 1.185e-02 515.44 <2e-16 ***

No.records 6.806e-06 1.462e-07 46.54 <2e-16 ***

p 3.487e+02 2.607e+01 13.38 <2e-16 ***

Signif. codes: 0 ‘***’ 0.001 ‘**’ 0.01 ‘*’ 0.05 ‘.’ 0.1 ‘ ’ 1

(Dispersion parameter for poisson family taken to be 1)

Null deviance: 9271.9 on 33 degrees of freedom

Residual deviance: 6835.2 on 31 degrees of freedom

AIC: 7110.7

Number of Fisher Scoring iterations: 5

1. GLM model for salinity

*GLM.numsp.salinity <- glm(No.species ~ No.records + salinity,family = "poisson", data = GLM)*

*summary(GLM.numsp.salinity)*

Call:

glm(formula = No.species ~ No.records + salinity,family = "poisson",

data = GLM)

Coefficients:

Estimate Std. Error z value Pr(>|z|)

(Intercept) 1.289e+01 5.714e-01 22.57 <2e-16 ***

No.records 5.947e-06 1.891e-07 31.44 <2e-16 ***

salinity -1.924e-01 1.647e-02 -11.68 <2e-16 ***

---

Signif. codes: 0 ‘***’ 0.001 ‘**’ 0.01 ‘*’ 0.05 ‘.’ 0.1 ‘ ’ 1

(Dispersion parameter for poisson family taken to be 1)

Null deviance: 9271.9 on 33 degrees of freedom

Residual deviance: 6876.6 on 31 degrees of freedom

AIC: 7152

Number of Fisher Scoring iterations: 5

1. GLM model for tempreture

*GLM.numsp.temp <- glm(No.species ~ No.records + T, family = "poisson", data = GLM)*

*summary(GLM.numsp.temp)*

Call:

glm(formula = No.species ~ No.records + T, family = "poisson",

data = GLM)

Coefficients:

Estimate Std. Error z value Pr(>|z|)

(Intercept) 5.915e+00 1.495e-02 395.54 <2e-16 ***

No.records 5.017e-06 1.664e-07 30.16 <2e-16 ***

T 1.436e-01 5.501e-03 26.11 <2e-16 ***

Signif. codes: 0 ‘***’ 0.001 ‘**’ 0.01 ‘*’ 0.05 ‘.’ 0.1 ‘ ’ 1

(Dispersion parameter for poisson family taken to be 1)

Null deviance: 9271.9 on 33 degrees of freedom

Residual deviance: 6305.2 on 31 degrees of freedom

AIC: 6580.7

Number of Fisher Scoring iterations: 5

## Model selection for number of species, whole database

Here we use AIC to compare models for goodness of fit while penalizing for overparameterization. Models are ranked in order of AIC score, with the lowest scoring model (model with the best compromise between fit and complexity) first in the list. AIC score for each model differs from the top model and can be used as an estimate of the relative support for each model. A delta AIC of 2 is potentially a significantly better fit, with higher delta AICs between models indicating increasingly larger differences in model fit while correcting for the number of parameters.

*GLM.numsp.models <- list(GLM.numsp.intercept = GLM.numsp.intercept,*

*GLM.numsp.numrec = GLM.numsp.numrec,*

*GLM.numsp.primary productivity = GLM.numsp.primary productivity,,*

*GLM.numsp.chlorophyll=GLM.numsp.chlorophyll*

*GLM.numsp.depth = GLM.numsp.depth,*

*GLM.numsp.lat = GLM.numsp.lat,*

*Curvvel = GLM.numsp.Curvvel,*

*GLM.numsp.DISSOLVED.OXYGEN= GLM.numsp.DISSOLVED.OXYGEN,*

*GLM.numsp.PO4= GLM.numsp.PO4,*

*GLM.numsp.pH = GLM.numsp.pH,*

*GLM.numsp.salinity = GLM.numsp.salinity,*

*GLM.numsp.Area = GLM.numsp.Area,*

*GLM.numsp.temp = GLM.numsp.temp)*

*GLM.numsp.aic.df <- data.frame(Model = names(GLM.numsp.models),*

*AIC = sapply(GLM.numsp.models, function(x) AICc(x)),*

*akaike.weights(sapply(GLM.numsp.models, function(x) AICc(x))))*

*GLM.numsp.aic.df <- GLM.numsp.aic.df[order(GLM.numsp.aic.df$AIC),]*

*GLM.numsp.aic.df$Cumulative.Weight <- cumsum(GLM.numsp.aic.df$weights)*

*kable(GLM.numsp.aic.df, row.names = FALSE)*

Model AIC deltaAIC rel.LL weights Cumulative.Weight

GLM.numsp.lat 5984.8859 0.0000 1.0000 1.0000 1.0000

GLM.numsp.chl 6020.7991 35.9132 0.0000 0.0000 1.0000

GLM.numsp.temp 6095.4784 110.5925 0.0000 0.0000 1.0000

GLM.numsp.PO4 6100.3532 115.4673 0.0000 0.0000 1.0000

GLM.numsp.salinity 6413.6485 428.7626 0.0000 0.0000 1.0000

GLM.numsp.pp 6417.3041 432.4181 0.0000 0.0000 1.0000

GLM.numsp.DO 6442.5315 457.6456 0.0000 0.0000 1.0000

GLM.numsp.pH 6476.0047 491.1188 0.0000 0.0000 1.0000

GLM.numsp.Area 6480.2646 495.3787 0.0000 0.0000 1.0000

GLM.numsp.depth 6495.7344 510.8485 0.0000 0.0000 1.0000

GLM.numsp.Curvvel 6501.3640 516.4781 0.0000 0.0000 1.0000

GLM.numsp.numrec 6503.8480 518.9621 0.0000 0.0000 1.0000

GLM.numsp.intercept Inf Inf 0.0000 0.0000 1.0000

As it could be seen, only geographical position (spatial autocorrelation) is a fit predictor of distribution amphipods along 5-degree latitudal band.

## Plots of GLM analysis of the number of species

The extracted plots of the relation between environmental variables and the extracted models were presented for LMs analysis of the number of species.


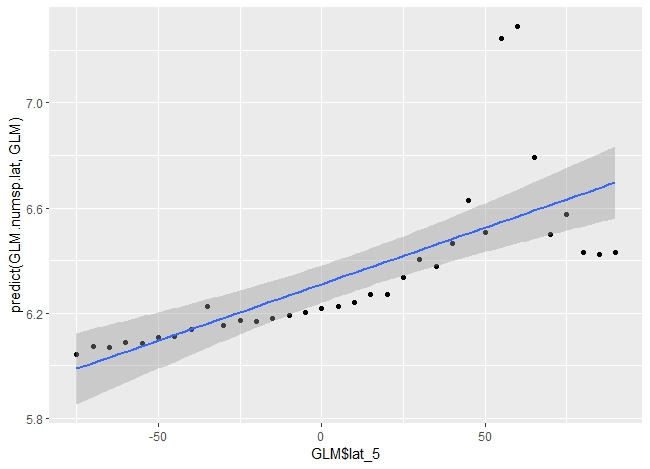


**Figure 87S.** qplot(GLM$lat_5, predict(GLM.numsp.lat, GLM)) + geom_smooth(method = "glm")


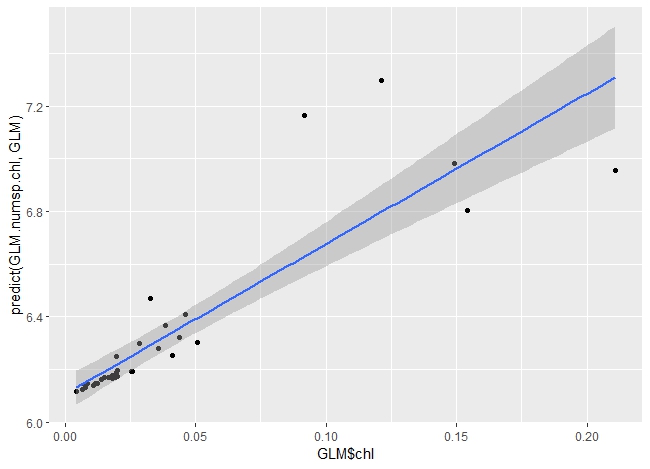


**Figure 88S.** qplot(GLM$chlorophyll predict(GLM.numsp.chlorophyll GLM)) + geom_smooth(method = "glm")


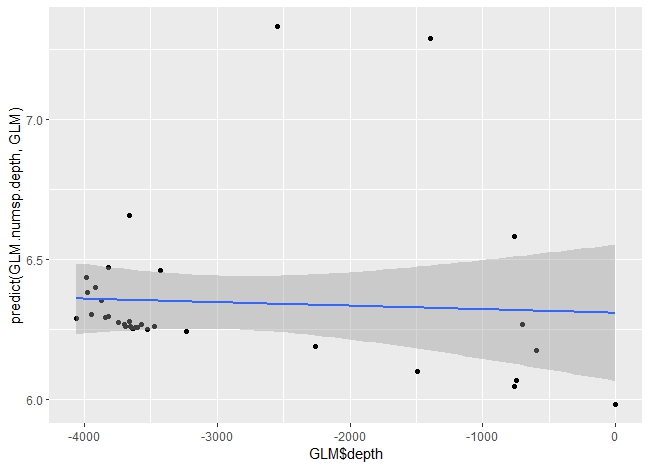


**Figure 89S.**qplot(GLM$depth, predict(GLM.numsp.depth, GLM)) + geom_smooth(method = "glm")


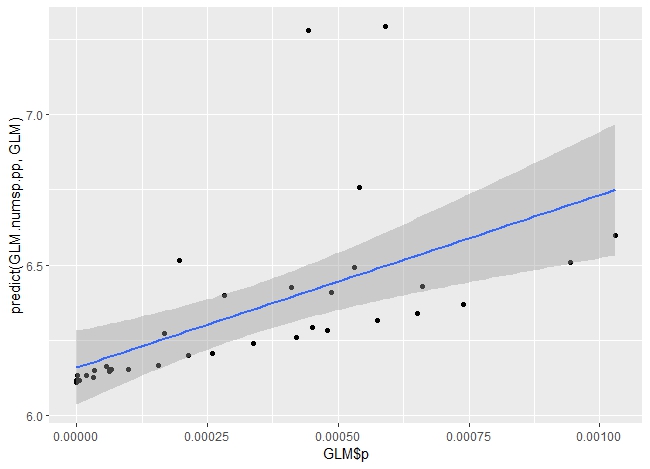

**Figure 90S.** qplot(GLM$pH, predict(GLM.numsp.pH, GLM)) + geom_smooth(method = glm")

"
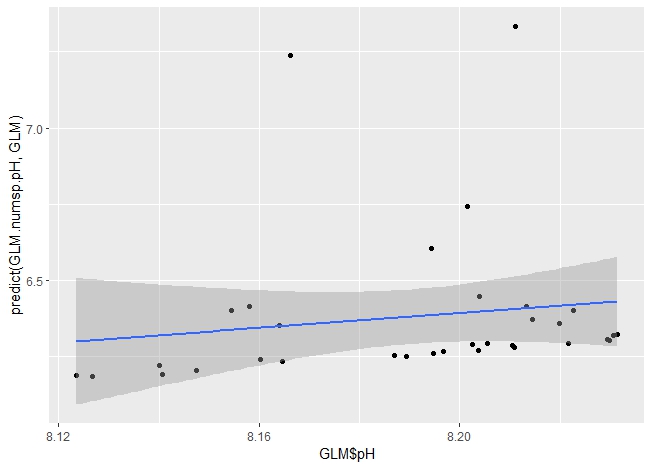


**Figure 91S.** qplot(GLM$p, predict(GLM.numsp.primary productivity,GLM)) + geom_smooth(method="glm")


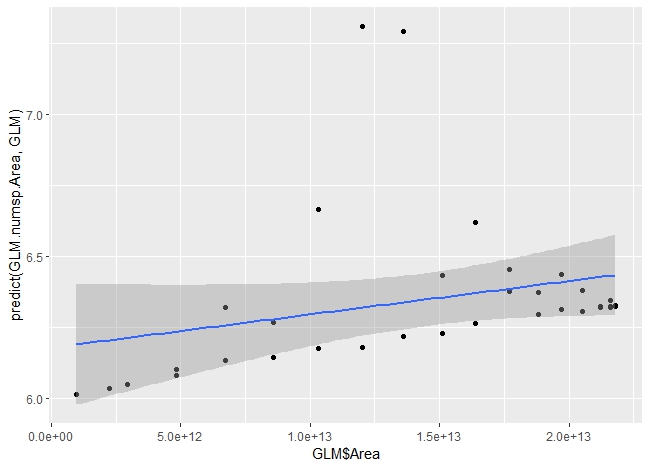


**Figure 92S.**qplot(GLM$Area, predict(GLM.numsp.Area, GLM)) + geom_smooth(method = "glm")


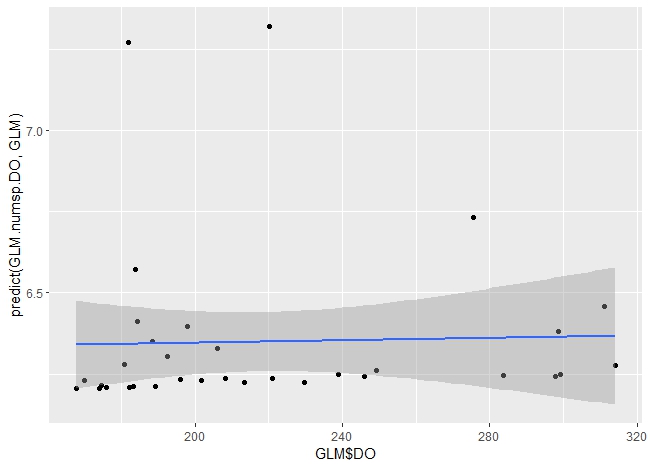


**Figure 93S.**qplot(GLM$DISSOLVED.OXYGEN, predict(GLM.numsp.DISSOLVED.OXYGEN, GLM)) + geom_smooth(method = "glm")


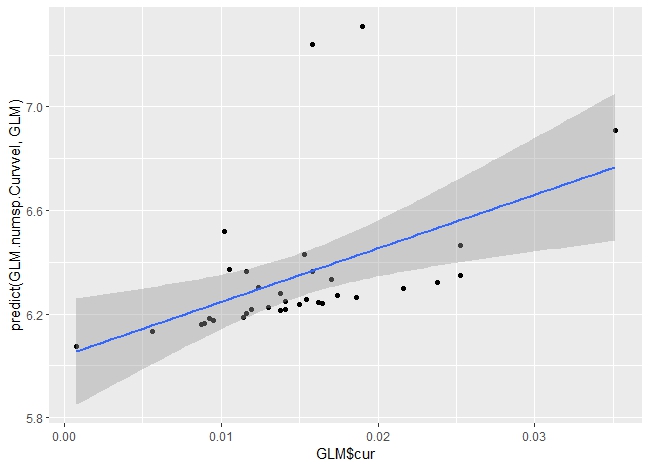


**Figure 94S.** qplot(GLM$current predict(GLM.numsp.Curvvel, GLM)) + geom_smooth(method = "glm")


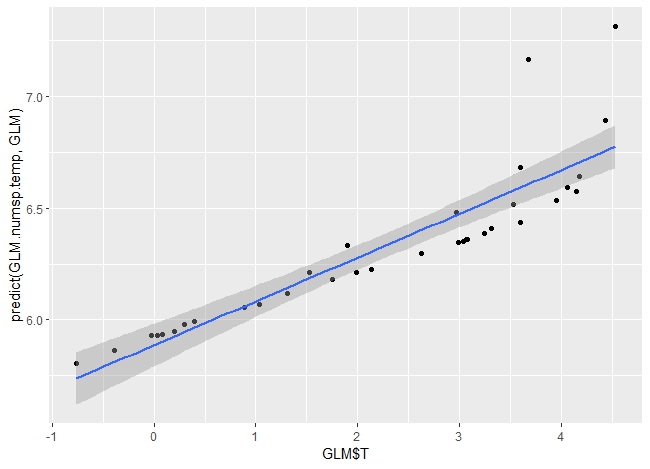


**Figure 95S.** qplot(GLM$T, predict(GLM.numsp.temp, GLM)) + geom_smooth(method = "glm")


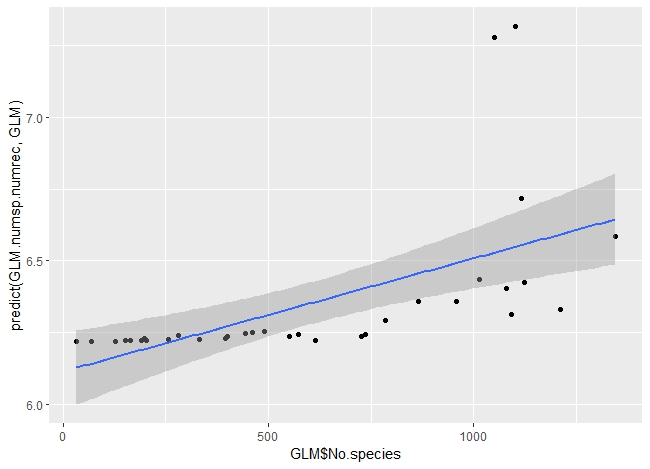


**Figure 96S.**qplot(GLM$No.species, predict(GLM.numsp.numrec, GLM)) + geom_smooth(method = "glm")


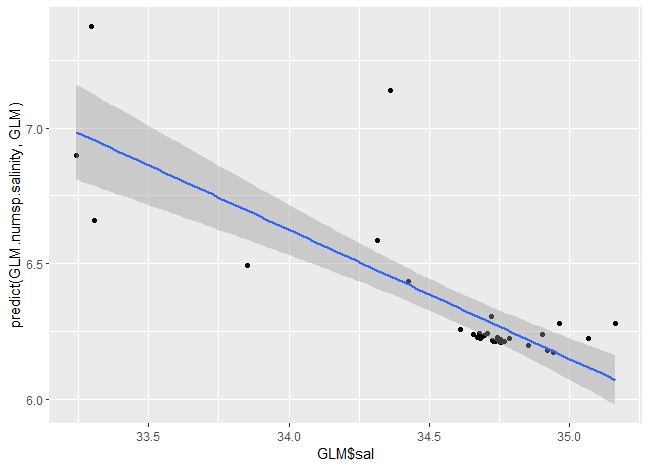


**Figure 97S.** qplot(GLM$salinity,predict(GLM.numsp.salinity, GLM)) + geom_smooth(method = "glm")

# GLM for ES50, whole database

The scripts, plots, and figures of GLM analysis for ES50 of total benthic species were presented here.

1. GAM model for intercept

*GLM.ES50.intercept <- glm(ES50 ~ 1, family = "poisson", data = GLM)*

*summary(GLM.ES50.intercept)*

Call:

glm(formula = ES50 ~ 1, family = "poisson", data = GLM)

Coefficients:

Estimate Std. Error z value Pr(>|z|)

(Intercept) 4.32918 0.01969 219.9 <2e-16 ***

Signif. codes: 0 ‘***’ 0.001 ‘**’ 0.01 ‘*’ 0.05 ‘.’ 0.1 ‘ ’ 1

(Dispersion parameter for poisson family taken to be 1)

Null deviance: 55.646 on 33 degrees of freedom

Residual deviance: 55.646 on 33 degrees of freedom

AIC: 266.99

Number of Fisher Scoring iterations: 4

1. GAM model for depth

*GLM.ES50.depth <- glm(ES50 ~ depth, family = "poisson", data = GLM)*

*summary(GLM.ES50.depth)*

Call:

glm(formula = ES50 ~ depth, family = "poisson", data = GLM)

Coefficients:

Estimate Std. Error z value Pr(>|z|)

(Intercept) 4.162e+00 5.336e-02 78.007 < 2e-16 ***

depth -5.581e-05 1.633e-05 -3.417 0.000634 ***

Signif. codes: 0 ‘***’ 0.001 ‘**’ 0.01 ‘*’ 0.05 ‘.’ 0.1 ‘ ’ 1

(Dispersion parameter for poisson family taken to be 1)

Null deviance: 55.646 on 33 degrees of freedom

Residual deviance: 43.633 on 32 degrees of freedom

AIC: 256.98

Number of Fisher Scoring iterations: 4

1. GLM model for area

*GLM.ES50.Area <- glm(ES50 ~ Area, family = "poisson", data = GLM)*

*summary(GLM.ES50.depth)*

glm(formula = ES50 ~ depth, family = "poisson", data = GLM)

Coefficients:

Estimate Std. Error z value Pr(>|z|)

(Intercept) 4.162e+00 5.336e-02 78.007 < 2e-16 ***

depth -5.581e-05 1.633e-05 -3.417 0.000634 ***

Signif. codes: 0 ‘***’ 0.001 ‘**’ 0.01 ‘*’ 0.05 ‘.’ 0.1 ‘ ’ 1

(Dispersion parameter for poisson family taken to be 1)

Null deviance: 55.646 on 33 degrees of freedom

Residual deviance: 43.633 on 32 degrees of freedom

AIC: 256.98

Number of Fisher Scoring iterations: 4

GLM.ES50.lat <- glm(ES50 ~ lat_5, family = "poisson", data = GLM)

summary(GLM.ES50.lat)

Call:

glm(formula = ES50 ~ lat_5, family = "poisson", data = GLM)

Coefficients:

Estimate Std. Error z value Pr(>|z|)

(Intercept) 4.3380312 0.0197437 219.717 < 2e-16 ***

lat_5 -0.0015797 0.0004021 -3.929 8.54e-05 ***

---

Signif. codes: 0 ‘***’ 0.001 ‘**’ 0.01 ‘*’ 0.05 ‘.’ 0.1 ‘ ’ 1

(Dispersion parameter for poisson family taken to be 1)

Null deviance: 55.646 on 33 degrees of freedom

Residual deviance: 40.183 on 32 degrees of freedom

AIC: 253.53

Number of Fisher Scoring iterations:4

1. GLM model for chlorophyll

*GLM.ES50.chlorophyll <- glm(ES50 ~ chlorophyll family = "poisson", data = GLM)*

*summary(GLM.ES50.chlorophyll)*

Call:

glm(formula = ES50 ~ chlorophyll family = "poisson", data = GLM)

Coefficients:

Estimate Std. Error z value Pr(>|z|)

(Intercept) 4.34844 0.02568 169.306 <2e-16 ***

chlorophyll -0.48692 0.42285 -1.152 0.25

Signif. codes: 0 ‘***’ 0.001 ‘**’ 0.01 ‘*’ 0.05 ‘.’ 0.1 ‘ ’ 1

(Dispersion parameter for poisson family taken to be 1)

Null deviance: 55.646 on 33 degrees of freedom

Residual deviance: 54.297 on 32 degrees of freedom

AIC: 267.64

Number of Fisher Scoring iterations: 4

1. GLM model for current

*GLM.ES50.Curvvel <- glm(ES50 ~ current family = "poisson", data = GLM)*

*summary(GLM.ES50.Curvvel)*

Call:

glm(formula = ES50 ~ current family = "poisson", data = GLM)

Coefficients:

Estimate Std. Error z value Pr(>|z|)

(Intercept) 4.27516 0.05092 83.960 <2e-16 ***

current 3.62429 3.13523 1.156 0.248

Signif. codes: 0 ‘***’ 0.001 ‘**’ 0.01 ‘*’ 0.05 ‘.’ 0.1 ‘ ’ 1

(Dispersion parameter for poisson family taken to be 1)

Null deviance: 55.646 on 33 degrees of freedom

Residual deviance: 54.319 on 32 degrees of freedom

AIC: 267.67

Number of Fisher Scoring iterations: 4

1. GLM model for pH

*GLM.ES50.pH <- glm(ES50 ~ pH, family = "poisson", data = GLM)*

*summary(GLM.ES50.pH)*

Call:

glm(formula = ES50 ~ pH, family = "poisson", data = GLM)

Coefficients:

Estimate Std. Error z value Pr(>|z|)

(Intercept) 3.60817 0.16454 21.929 < 2e-16 ***

pH 0.08992 0.02024 4.444 8.84e-06 ***

Signif. codes: 0 ‘***’ 0.001 ‘**’ 0.01 ‘*’ 0.05 ‘.’ 0.1 ‘ ’ 1

(Dispersion parameter for poisson family taken to be 1)

Null deviance: 55.646 on 33 degrees of freedom

Residual deviance: 30.262 on 32 degrees of freedom

AIC: 243.61

Number of Fisher Scoring iterations: 4

1. GLm model for PO4

*GLM.ES50.PO4 <- glm(ES50 ~ PO4, family = "poisson", data = GLM)*

*summary(GLM.ES50.PO4)*

Call:

glm(formula = ES50 ~ PO4, family = "poisson", data = GLM)

Coefficients:

Estimate Std. Error z value Pr(>|z|)

(Intercept) 4.10176 0.08387 48.905 < 2e-16 ***

PO4 0.11777 0.04193 2.809 0.00497 **

Signif. codes: 0 ‘***’ 0.001 ‘**’ 0.01 ‘*’ 0.05 ‘.’ 0.1 ‘ ’ 1

(Dispersion parameter for poisson family taken to be 1)

Null deviance: 55.646 on 33 degrees of freedom

Residual deviance: 47.524 on 32 degrees of freedom

AIC: 260.87

Number of Fisher Scoring iterations: 4

1. GLM model for dissolved oxygen

*GLM.ES50.DISSOLVED.OXYGEN<- glm(ES50 ~ DISSOLVED.OXYGEN, family = "poisson", data = GLM)*

*summary(GLM.ES50.dissolved.oxygen)*

Call:

glm(formula = ES50 ~ DISSOLVED.OXYGEN, family = "poisson", data = GLM)

Coefficients:

Estimate Std. Error z value Pr(>|z|)

(Intercept) 4.4685489 0.0967807 46.172 <2e-16 ***

DISSOLVED.OXYGEN -0.0006412 0.0004373 -1.466 0.143

Signif. codes: 0 ‘***’ 0.001 ‘**’ 0.01 ‘*’ 0.05 ‘.’ 0.1 ‘ ’ 1

(Dispersion parameter for poisson family taken to be 1)

Null deviance: 55.646 on 33 degrees of freedom

Residual deviance: 53.477 on 32 degrees of freedom

AIC: 266.82

Number of Fisher Scoring iterations: 4

1. GLM model for primary productivity

*GLM.ES50.primary productivity, <- glm(ES50 ~ p, family = "poisson", data = GLM)*

*summary(GLM.ES50.primary productivity,)*

Call:

glm(formula = ES50 ~ p, family = "poisson", data = GLM)

Coefficients:

Estimate Std. Error z value Pr(>|z|)

(Intercept) 4.34741 0.02988 145.487 <2e-16 ***

p -56.67110 70.39940 -0.805 0.421

Signif. codes: 0 ‘***’ 0.001 ‘**’ 0.01 ‘*’ 0.05 ‘.’ 0.1 ‘ ’ 1

(Dispersion parameter for poisson family taken to be 1)

Null deviance: 55.646 on 33 degrees of freedom

Residual deviance: 54.996 on 32 degrees of freedom

AIC: 268.34

Number of Fisher Scoring iterations: 4

1. GLM model for salinity

*GLM.ES50.salinity <- glm(ES50 ~ salinity,family = "poisson", data = GLM)*

*summary(GLM.ES50.salinity)*

Call:

glm(formula = ES50 ~ salinity,family = "poisson", data = GLM)

Coefficients:

Estimate Std. Error z value Pr(>|z|)

(Intercept) 2.27424 1.51048 1.506 0.132

salinity 0.05940 0.04365 1.361 0.174

(Dispersion parameter for poisson family taken to be 1)

Null deviance: 55.646 on 33 degrees of freedom

Residual deviance: 53.761 on 32 degrees of freedom

AIC: 267.11

Number of Fisher Scoring iterations: 4

1. GLM model for tempretaure

*GLM.ES50.temp <- glm(ES50 ~ T, family = "poisson", data = GLM)*

*summary(GLM.ES50.temp)*

Call:

glm(formula = ES50 ~ T, family = "poisson", data = GLM)

Coefficients:

Estimate Std. Error z value Pr(>|z|)

(Intercept) 4.333141 0.034356 126.13 <2e-16 ***

T -0.001762 0.012547 -0.14 0.888

Signif. codes: 0 ‘***’ 0.001 ‘**’ 0.01 ‘*’ 0.05 ‘.’ 0.1 ‘ ’ 1

(Dispersion parameter for poisson family taken to be 1)

Null deviance: 55.646 on 33 degrees of freedom

Residual deviance: 55.626 on 32 degrees of freedom

AIC: 268.97

Number of Fisher Scoring iterations: 4

## Model selection for ES50, the whole database

*GLM.ES50.models <- list(GLM.ES50.intercept = GLM.ES50.intercept,*

*GLM.ES50.primary productivity = GLM.ES50.primary productivity,,*

*GLM.ES50.chlorophyll=GLM.ES50.chlorophyll*

*GLM.ES50.depth = GLM.ES50.depth,*

*GLM.ES50.lat = GLM.ES50.lat,*

*GLM.ES50.Curvvel = GLM.ES50.Curvvel,*

*GLM.ES50.DISSOLVED.OXYGEN= GLM.ES50.DISSOLVED.OXYGEN,*

*GLM.ES50.PO4= GLM.ES50.PO4,*

*GLM.ES50.pH = GLM.ES50.pH,*

*GLM.ES50.salinity = GLM.ES50.salinity,*

*GLM.ES50.Area = GLM.ES50.Area,*

*GLM.ES50.temp = GLM.ES50.temp)*

*GLM.ES50.aic.df <- data.frame(Model = names(GLM.ES50.models),*

*AIC = sapply(GLM.ES50.models, function(x) AICc(x)),*

*akaike.weights(sapply(GLM.ES50.models, function(x) AICc(x))))*

*GLM.ES50.aic.df <- GLM.ES50.aic.df[order(GLM.ES50.aic.df$AIC),]*

*GLM.ES50.aic.df$Cumulative.Weight <- cumsum(GLM.ES50.aic.df$weights)*

*kable(GLM.ES50.aic.df, row.names = FALSE)*

| Model | AIC | deltaAIC | rel.LL | weights | Cumulative.Weight |
| --- | --- | --- | --- | --- | --- |
| GLM.ES50.lat | 231.4169 | 0.0000 | 1.0000 | 0.5114 | 0.5114 |
| GLM.ES50.salinity | 234.7531 | 3.3362 | 0.1886 | 0.0965 | 0.6079 |
| GLM.ES50.chl | 235.6104 | 4.1935 | 0.1229 | 0.0628 | 0.6707 |
| GLM.ES50.pp | 235.6924 | 4.2755 | 0.1179 | 0.0603 | 0.7310 |
| GLM.ES50.temp | 235.7059 | 4.2890 | 0.1171 | 0.0599 | 0.7909 |
| GLM.ES50.depth | 235.7800 | 4.3632 | 0.1129 | 0.0577 | 0.8487 |
| GLM.ES50.intercept | 236.4553 | 5.0384 | 0.0805 | 0.0412 | 0.8899 |
| GLM.ES50.PO4 | 236.6674 | 5.2505 | 0.0724 | 0.0370 | 0.9269 |
| GLM.ES50.Area | 237.3266 | 5.9098 | 0.0521 | 0.0266 | 0.9535 |
| GLM.ES50.Curvvel | 238.2635 | 6.8466 | 0.0326 | 0.0167 | 0.9702 |
| GLM.ES50.pH | 238.2833 | 6.8665 | 0.0323 | 0.0165 | 0.9867 |
| GLM.ES50.DO | 238.7179 | 7.3010 | 0.0260 | 0.0133 | 1.0000 |

The geographical position is the only predictor of ES50 of amphipod distribution at the global scale.

## Plots of GLM analysis of ES50

The extracted plots of the relation between environmental variables and the extracted models were presented for LMs analysis of ES50.


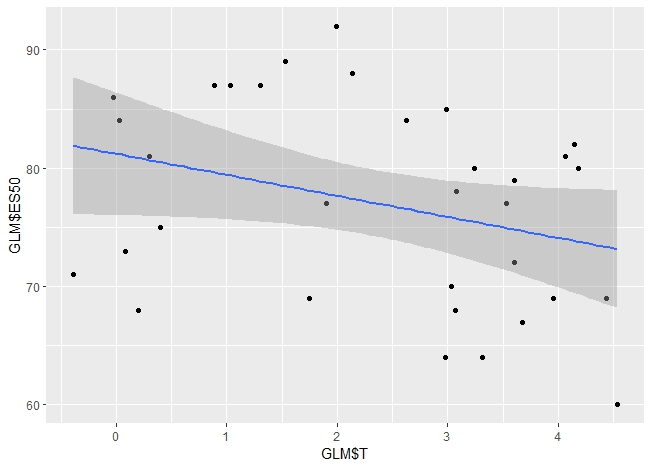


**Figure 98S.**qplot(GLM$T, GLM$ES50) + geom_smooth(method = "glm")


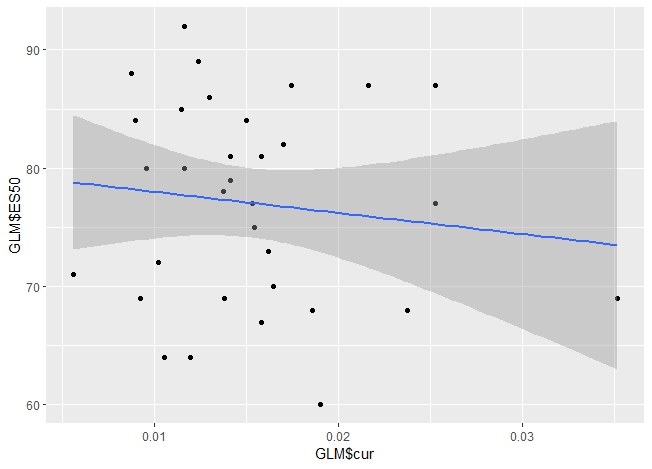


**Figure 99S.**qplot(GLM$current GLM$ES50) + geom_smooth(method = "glm")

**Figure 100S.**qplot(GLM$salinity,GLM$ES50) + geom_smooth(method = "glm")

**Figure 101S.**qplot(GLM$DISSOLVED.OXYGEN, GLM$ES50) + geom_smooth(method = "glm")

**Figure 102S.**qplot(GLM$Area, GLM$ES50) + geom_smooth(method = "glm")

**Figure 103S.**qplot(GLM$PO4, GLM$ES50) + geom_smooth(method = "glm")

**Figure 104S.**qplot(GLM$p, GLM$ES50) + geom_smooth(method = "glm")

**Figure 105S.**qplot(GLM$pH, GLM$ES50) + geom_smooth(method = "glm")

**Figure 106S.**qplot(GLM$lat_5, GLM$ES50) + geom_smooth(method = "glm")

**Figure 107S.**qplot(GLM$chlorophyll GLM$ES50) + geom_smooth(method = "glm")

**Figure 108S.**qplot(GLM$depth, GLM$ES50) + geom_smooth(method = "glm")

## Reference

1. Unep-Wcmc, I. Protected planet: the world database on protected areas (WDPA). *UNEP-WCMC and IUCN, Cambridge, UK Available at: http://wwwprotectedplanetnet, Accessed date* **21**, (2018).
